# Retro‐age: A unique epigenetic biomarker of aging captured by DNA methylation states of retroelements

**DOI:** 10.1111/acel.14288

**Published:** 2024-08-02

**Authors:** Lishomwa C. Ndhlovu, Matthew L. Bendall, Varun Dwaraka, Alina P. S. Pang, Nicholas Dopkins, Natalia Carreras, Ryan Smith, Douglas F. Nixon, Michael J. Corley

**Affiliations:** ^1^ Department of Medicine, Division of Infectious Diseases Weill Cornell Medicine New York New York City USA; ^2^ TruDiagnostic Lexington Kentucky USA

**Keywords:** aging, biomarker, DNA methylation, endogenous retrovirus, epigenetic clock, epigenetics, retroelement

## Abstract

Reactivation of retroelements in the human genome has been linked to aging. However, whether the epigenetic state of specific retroelements can predict chronological age remains unknown. We provide evidence that locus‐specific retroelement DNA methylation can be used to create retroelement‐based epigenetic clocks that accurately measure chronological age in the immune system, across human tissues, and pan‐mammalian species. We also developed a highly accurate retroelement epigenetic clock compatible with EPICv.2.0 data that was constructed from CpGs that did not overlap with existing first‐ and second‐generation epigenetic clocks, suggesting a unique signal for epigenetic clocks not previously captured. We found retroelement‐based epigenetic clocks were reversed during transient epigenetic reprogramming, accelerated in people living with HIV‐1, and responsive to antiretroviral therapy. Our findings highlight the utility of retroelement‐based biomarkers of aging and support a renewed emphasis on the role of retroelements in geroscience.

## Graphical abstract

This study reports that locus‐specific retroelement DNA methylation states can be used to create unique retroelement‐based epigenetic clocks that accurately measure chronological age in the immune system, across human tissues, and pan‐mammalian species. We found retroelement‐based epigenetic clocks minimally overlapped with prior epigenetic clocks and were reversed during transient epigenetic reprogramming, accelerated in people living with HIV‐1, and responsive to antiretroviral therapy.

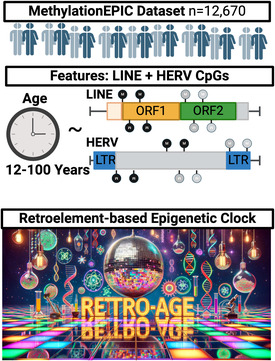

**Abbreviations**
ART: antiretroviral therapy
CpG: cytosine‐phosphate‐Guanine
CTCF: CCCTC‐binding factor
DNA: deoxyribonucleic acid
DNAmAge: DNA methylation age
ENCODE: encyclopedia of DNA elements
ERV: endogenous retrovirus
GTEx: genotype‐tissue expression
HERV: human endogenous retrovirus
HIV: human immunodeficiency virus
HIV‐1: human immunodeficiency virus Type 1
IPSC: induced pluripotent stem cell
LINE: long interspersed nuclear element
LTR: long terminal repeat
MAE: median absolute error
PBMC: peripheral blood mononuclear cell
PLWH: people living with HIV
PrEP: pre‐exposure prophylaxis
RNA: ribonucleic acid
RNA‐Seq: RNA sequencing
TFBS: transcription factor binding site
TSS: transcription start site
ZNF: zinc finger protein

## 1 INTRODUCTION

Retroelements such as human endogenous retroviruses (HERVs) and long interspersed nuclear elements (LINEs) constitute a significant portion of the human genome (Lander et al., 2001; Nurk et al., 2022). While the majority of retroelements embedded within the human genome are typically repressed by epigenetic mechanisms that include DNA methylation and chromatin modifications, the activity of specific HERVs and LINEs in the human genome have been shown to impact gene regulation, gene expression, genomic stability, development, and the pathogenesis of various human diseases (Beck et al., 2011; Dopkins & Nixon, 2023; Fueyo et al., 2022). Moreover, the resurrection of particular HERVs and LINEs have been linked to the aging process (De Cecco et al., 2019; Liu et al., 2023; Zhang et al., 2023). Together, evidence supports a key role of retroelement activity in biological hallmarks of aging. However, the interplay between the preferential insertion and reactivation of certain retroelements in the human genome and aging remains largely unexplored.

Epigenetic clocks are highly accurate biological markers of aging based on patterns of DNA methylation at specific regions of the human genome. They offer a way to measure biological age, which can be distinct from chronological age (Bell et al., 2019; Bocklandt et al., 2011; Hannum et al., 2013; Horvath, 2013; Horvath & Raj, 2018). However, current first‐, second‐, and third‐generation epigenetic clocks have been constructed from underlying DNA methylation features that have not focused on retroelements (Belsky et al., 2022; Hannum et al., 2013; Horvath, 2013; Levine et al., 2018; Lu, Quach, et al., 2019; Ying et al., 2024). Yet, context‐dependent DNA methylation changes have been shown to occur with age at repetitive DNA sequences, introns, and intergenic regions of the genome, which are enriched in retroelements (Jones et al., 2015). The reactivation of certain HERVs and LINEs have been observed to increase with age (De Cecco et al., 2019; Liu et al., 2023; Zhang et al., 2023); however, the potential of using the DNA methylation states of specific HERVs and LINEs in epigenetic clocks for estimating chronological age remains unclear.

Here, we investigate DNA methylation dynamics of retroelements as chronological age predictors. We enhanced the annotation of Illumina's MethylationEPIC v1.0 platform to identify CpGs located within manually curated locus‐specific HERVs and LINEs (Bendall et al., 2019), which were previously unaccounted for in standard and custom add on annotations. Using these Telescope annotation‐based retroelement CpGs and a DNA methylation dataset from 12,670 individuals across the lifespan, we developed a highly accurate chronological age composite Retroelement‐Age clock based on DNA methylation states of HERVs and LINEs. We further constructed a composite Retroelement‐Age V2 clock compatible with the MethylationEPIC v2.0 platform and demonstrate that retroelement clocks extend to diverse human tissues and across mammalian species using DNA methylation from GTEx (Oliva et al., 2023) and from the Mammalian Methylation Consortium (Lu et al., 2023). Our results substantiate the hypothesis that the dysregulation of retroelements may play a role in the distinctive biological features associated with aging.

## 2 RESULTS

### 2.1 Development of epigenetic clocks based on HERVs and LINEs: Retroelement‐Age

**FIGURE 1:**
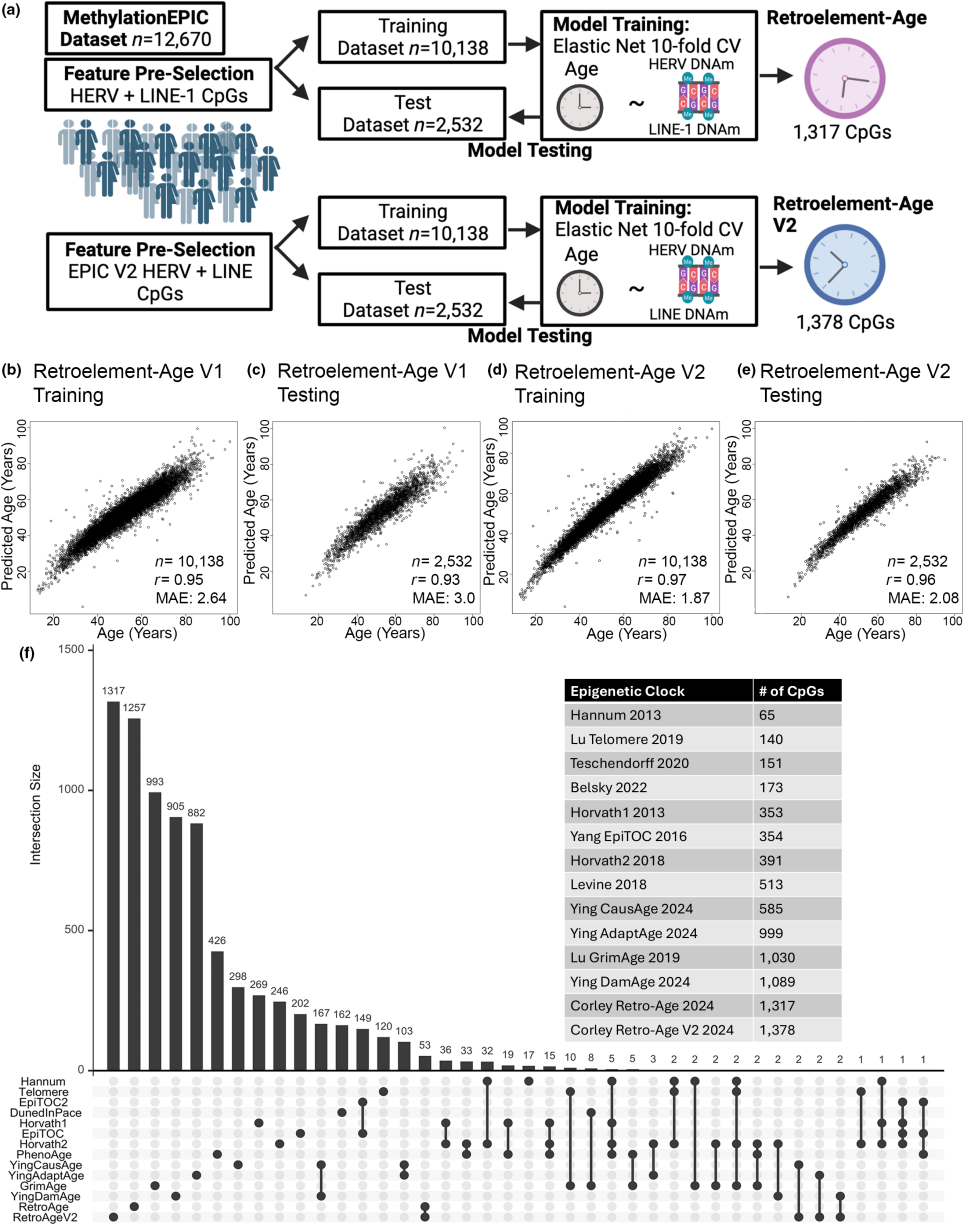
Construction of Composite Retroelement‐Age and Retroelement‐Age V2 Epigenetic Clocks. (a) Diagram of workflow utilized to construct Retroelement‐Age and Retroelement‐Age V2. (b) Age estimation 10‐fold cross validation in training and (c) test datasets for retroelement‐age. (d, e) Retroelement‐Age V2 clocks. Panels report the sample size (*n*), the median absolute error (MAE) and Pearson's correlation coefficient (*r*). (f) Intersection plot of existing first‐, second‐, and third‐generation epigenetic clocks CpGs, retroelement‐age and retroelement‐age V2.

Analysis of MethylationEPIC data relies on a reference annotation of CpGs typically utilizing Illumina's default annotation that does not identify whether a CpG is located within a locus‐specific HERV or LINE (Pidsley et al., 2016). Moreover, manually curated locus‐specific HERV CpGs are not available in enhanced annotations of the MethylationEPIC (Zhou et al., 2017, 2018). Hence, we leveraged a manually curated locus‐specific HERV and LINE annotation including 60 HERV families and 13,545 loci derived from L1Base (Bendall et al., 2019) to annotate probes included on the Illumina Infinium MethylationEPIC (EPIC) v.1.0 array. Telescope annotates HERVs based on a prototypical transcriptional unit containing an internal protein‐coding region flanked by long terminal repeat (LTR) regulatory regions. Utilizing the Telescope database, we identified that 10,917 probes (1.26% of EPIC v.1.0) assessed DNA methylation at a CpG within a HERV transcriptional unit or within an active LINE element (Bendall et al., 2019) (Data S1). We sought to develop a composite retroelement epigenetic clock (Retroelement‐Age) by considering both the DNA methylation states of HERVs and LINEs that predicted chronological age. We leveraged an EPIC v.1.0 dataset of blood DNA methylation EPIC v.1.0 array data from 12,670 people (40.89% female) with chronological ages ranging from 12 to 100 years old. The database was quality controlled, normalized, and filtered to 10,917 CpGs based on our HERV and LINE annotation. The dataset was preprocessed by randomly splitting into an 80% training and 20% test data set. A generalized linear elastic net model was fit with 10‐fold cross‐validation using glmnet on the 80% training dataset of 10,138 samples (Figure 1a). Retroelement‐Age V1 consisted of 1317 CpG sites (Table S1) and was highly predictive of chronological age in both training (Pearson's *r* = 0.95, median absolute error (MAE) = 2.57) and test datasets (Pearson's *r* = 0.89, MAE = 3.81). Since the majority of existing DNA methylation data is on the older 450 K array, we also built a compatible Retroelement‐Age‐450K clock that was constructed from retroelement CpGs common to the 450 K and EPIC v.1.0 arrays consisting of 1317 CpG sites predictive of chronological age in both training (Pearson's *r* = 0.90, MAE = 3.67) and test datasets (Pearson's *r* = 0.88, MAE = 4.02) (Table S2). Given the development of a new HumanMethylation EPIC v.2.0 DNA methylation array likely to be utilized in future geroscience research (Noguera‐Castells et al., 2023), we sought to enhance the utility of a composite Retroelement‐Clock for compatibility with both EPIC v.1.0 and v.2.0 data. Hence, we extended our manually curated annotation of Telescope‐based EPIC v.1.0 CpGs to overlapping probes on the EPIC v2.0 platform. We also expanded our retroelement CpGs for EPIC v2.0 to include LTR and LINE elements identified by RepeatMasker. Utilizing this expanded annotation of compatible retroelement CpGs for MethylationEPIC v2.0, we constructed a EPIC v.1.0 and v.2.0 compatible retroelement‐based epigenetic clock (Retroelement‐Age V2) based on a generalized linear elastic net model fit with 10‐fold cross‐validation on an 80% training dataset of 10,138 samples (Figure 1a). The composite Retroelement‐Age V2 epigenetic clock consisted of 1378 CpG sites (Table S3) and was the most predictive of chronological age (Figure 1d,e) compared to our prior retroelement‐based clocks in both training (Pearson's *r* = 0.97, MAE = 1.87) and test datasets (Pearson's *r* = 0.96, MAE = 2.08). This was likely due to MethylationEPIC v2.0 containing more reliable probes (Kaur et al., 2023; Noguera‐Castells et al., 2023).

### 2.2 Retroelement‐age clocks are constructed from unique CpGs when compared to existing epigenetic clocks

We compared whether any of the CpGs utilized in our Retroelement‐Age clocks predicting chronological age overlapped with CpGs utilized in the construction of 12 existing first, second, and third generation epigenetic clocks that were not constructed based on retroelement CpGs. We examined the intersection of our Retroelement‐Age V1 and Retroelement‐Age V2 clocks with Horvath's multi‐tissue predictor DNAmAge clock based on 353 CpG sites (Horvath, 2013), the Horvath skin‐and‐blood clock based on 391 CpG sites (Horvath, Oshima, et al., 2018), Levine's DNAmPhenoAge clock based on 513 CpG sites (Levine et al., 2018), Hannum's clock based on 71 CpG sites (Hannum et al., 2013), Lu's telomere length predictor based on 140 CpGs (Lu, Seeboth, et al., 2019), DNA methylation based mortality risk assessment GrimAge based on 1030 CpGs (Lu, Quach, et al., 2019), DunedinPace of aging based on 173 CpGs (Belsky et al., 2022), EpiTOC/EpiTOC2 mitotic clocks based on 354 and 151 CpGs (Teschendorff, 2020), and causality‐enriched epigenetic clocks AdaptAge, DamAge, and CausAge (Ying et al., 2024). We found that all CpGs utilized in our Retroelement‐Age V1 clock were unique and did not overlap with any existing epigenetic clocks, suggesting that retroelement‐based epigenetic clocks may capture novel biological DNA methylation features of aging not previously recognized (Figure 1f). As shown by prior studies (Liu et al., 2020), a subset of shared CpGs were observed among Horvath's first‐generation, second‐generation clocks, and DunedInPace of aging suggesting the construction of these clocks captured some similar DNA methylation features of aging (Figure 1f). Notably, we observed our Retroelement‐Age V2 clock overlapped with 9 CpGs (cg06672696, cg07286682, cg08822136, cg16936289, cg16810279, cg22277154, cg13261390, cg22277154, and cg24251135) used in AdaptAge, CausAge, and DamAge causality‐enriched epigenetic clocks recently developed using Mendelian randomization (Ying et al., 2024). This observation suggest some of the signal from our Retroelement‐Age V2 may include sites that contribute and/or protect against aging.

### 2.3 Retroelement‐age clocks are enriched in quiescent, active, and poised enhancer regions

**FIGURE 2:**
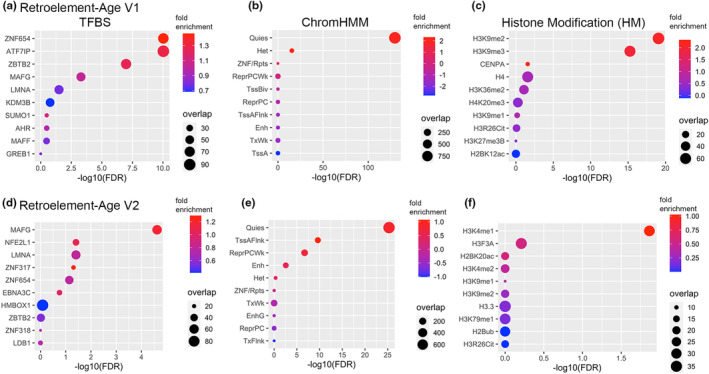
Enrichment plot of retroelement‐based epigenetic clocks in consensus transcription factor binding sites (TFBS), ENCODE ChromHMM chromatin states, and histone modifications for (a–c). Retroelement‐Age V1, (d–f). Retroelement‐Age V2. The SeSAMe R package tool KnowYourCG was utilized (Zhou et al., 2018). Fisher's exact test with false discovery rate (FDR), estimate represents fold enrichment, and overlap the number of CpGs.

We examined the overlap of CpGs in our retroelement clocks in specific regions of the epigenome based on consensus TFBSs, chromatin states, and consensus histone modifications. Consensus ChromHMM derived from 833 ENCODE ChromHMM calls from ENCODE version 2 were used (ENCODE Project Consortium, 2012). Retroelement‐Age was enriched in KRAB zinc finger protein ZNF654, ATF7IP, ZBTB2, MAFG, quiescent/heterochromatin states, and regions containing H3K9me2 and H3K9me3 (Figure 2a–c). H3K9me3 deposition has been shown to be mediated by ATF7IP and SETDB1, which also are required for silencing retroelements (Hu et al., 2021). ZBTB2‐binding dynamics in vivo are sensitive to differential DNA methylation and has been shown to repress the retrovirus HIV‐1 (Bruce et al., 2021; Karemaker & Vermeulen, 2018) and MAFG as a bidirectional regulator of transcription (Motohashi et al., 2006). Retroelement‐Age V2 CpGs were enriched in MAFG and interaction partner NFE2L1, ZNF317, and ZNF654 (Figure 2d). ZNF317 has been recently suggested to primarily target eutheria‐specific ERVs (Otsuka et al., 2023). In addition to being enriched in quiescent chromatin states, Retroelement‐Age V2 was enriched in active transcription start site (TSS)‐proximal promoter states, enhancer states, and repressed Polycomb states (Figure 2e). Polycomb group proteins are hypothesized as an evolutionarily conserved mechanism to silence transposable elements (Déléris et al., 2021). Retroelement‐Age V2 CpGs were enriched in H3K4me1 regions, a chromatin mark of poised and active enhancers (Figure 2f). These findings suggest our retroelement‐age clocks contain CpGs involved in the regulation and silencing of retroelements.

### 2.4 Independent validation and reliability of blood‐based retroelement‐based epigenetic clocks

**FIGURE 3:**
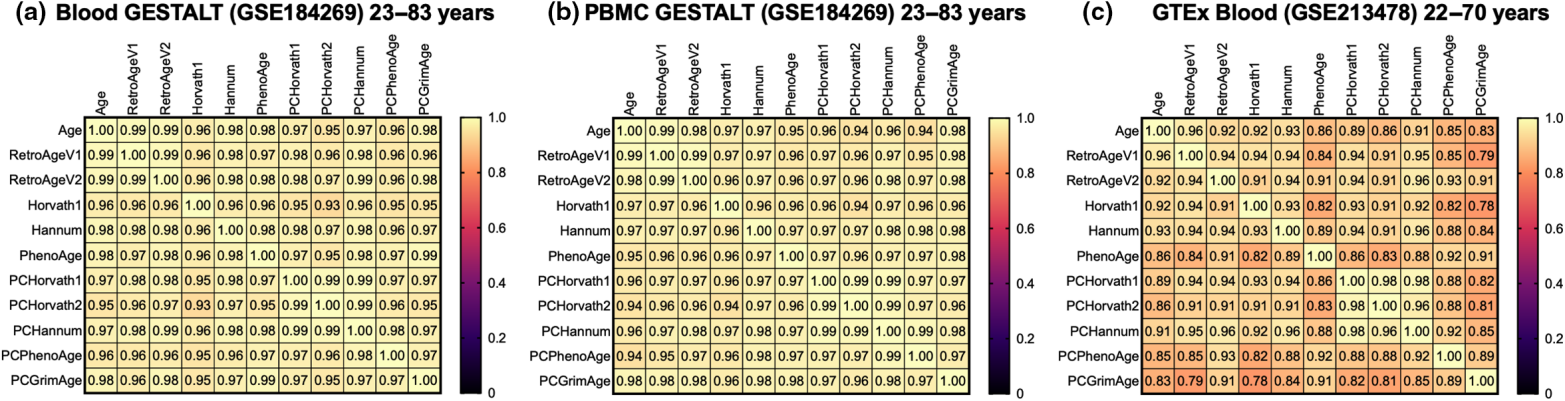
Correlograms of chronological age, retroelement‐based epigenetic clocks, first‐generation epigenetic clocks, second‐generation epigenetic clocks, and PC‐based epigenetic clocks in two external DNA methylation datasets (GSE184269 and GSE213478). Pearson correlation coefficient displayed.

We sought to evaluate the performance and validate our retroelement‐based epigenetic clocks in completely independent and external DNA methylation datasets. First, we utilized a DNA methylation EPIC v.1.0 dataset (GSE184269) of blood and peripheral blood mononuclear cells (PBMC) from healthy individuals interspersed over a wide chronological age range (22–83 years) from the NIH Genetic and Epigenetic Signatures of Translational Aging Laboratory Testing (GESTALT) study (Roy et al., 2023) to examine the relationships between chronological age, predicted epigenetic age based on our new retroelement clocks (Retroelement‐Age V1, Retroelement‐Age V2), and predicted epigenetic ages from existing epigenetic clocks. Despite being constructed from largely unique CpGs compared to previous epigenetic clocks, we found that Retroelement‐Age V1 (Pearson's *r* = 0.99) and Retroelement‐Age V2 (Pearson's *r* = 0.99) significantly associated with chronological age in blood to a similar or better degree than existing epigenetic clock algorithms such as Horvath1 (Pearson's *r* = 0.96), Hannum (Pearson's *r* = 0.98), and PhenoAge (Pearson's *r* = 0.98) (Figure 3a). We also observed this relationship was similar or better than new principal component‐based versions of epigenetic clocks (Higgins‐Chen et al., 2022) including PCHorvath1 (Pearson's *r* = 0.97), PCHorvath2 (Pearson's *r* = 0.95), PCHannum (Pearson's *r* = 0.97), PCPhenoAge (Pearson's *r* = 0.96), and PCGrimAge (Pearson's *r* = 0.98) (Figure 3a). We extended our analysis to DNA methylation data of peripheral blood mononuclear cells (PBMC) from the GESTALT study dataset and observed similar robust relationships between Retroelement‐Age V1 (Pearson's *r* = 0.99) and Retroelement‐Age V2 (Pearson's *r* = 0.98) with chronological age (Figure 3b). Second, we evaluated Retroelement‐Age V1 and V2 in another external DNA methylation EPIC v1.0 dataset generated from whole blood from Genotype‐Tissue Expression (GTEx) for participants ranging in age from 22 to 70 years old (Oliva et al., 2023) and observed that Retroelement‐Age V1 (Pearson's *r* = 0.96) and Retroelement‐Age V2 (Pearson's *r* = 0.92) significantly associated with chronological age (Figure 3c). Additionally, we assessed Retroelement‐Age V1 and V2 in a blood DNA methylation EPIC v1.0 dataset of 790 people generated by the Alzheimer's Disease Neuroimaging Initiative study and found Retroelement‐Age V1 (Pearson's *r* = 0.72) and Retroelement‐Age V2 (Pearson's *r* = 0.83) significantly associated with chronological age (Figure S1). Next, we utilized 30 replicate blood MethylationEPIC v1.0 samples obtained from DNA methylation EPIC v.1.0 data from the ADNI cohort (Vasanthakumar et al., 2020) to evaluate test–retest reliability of Retroelement‐Age V2 based on the intraclass correlation coefficient (ICC) using a linear mixed‐effects model. We found that reliability was high with an ICC of 0.996 (Figure S2).

### 2.5 Effect of HIV‐1 infection and antiretroviral therapy on retroelement‐based epigenetic clocks

**FIGURE 4:**
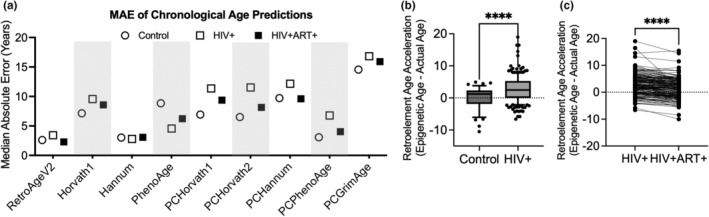
Application of retroelement‐based epigenetic clock to accelerated aging in HIV and antiretroviral therapy. (a) Median absolute error in chronological age prediction for Retroelement‐Age V2, first‐generation epigenetic clocks (Horvath1 and Hannum), second‐generation epigenetic clock (PhenoAge), and PC‐based epigenetic clocks for control, people living with HIV‐1 who are ART naive (HIV+), and people living with HIV on ART (HIV+ART+). (b) Age acceleration detected utilizing Retroelement‐Age V2 in external data from people living with HIV‐1 compared to controls. (c) Retroelement‐Age V2 responsive to antiretroviral therapy (ART) in people living with HIV followed longitudinally before and after 96 weeks of ART. Student's T tests and paired T tests, **** *p* < 0.001.

Prior studies have reported accelerated epigenetic aging in people living with infectious diseases such as HIV‐1 (Gross et al., 2016; Horvath & Levine, 2015). We tested whether our Retroelement‐Age V2 clock would capture accelerated epigenetic aging in DNA methylation dataset from a cohort of 185 people living without HIV‐1 (with pre‐ART and post‐ART longitudinal samples) and 44 demographically matched controls (GSE217633) (Esteban‐Cantos et al., 2021). We replicated evidence of epigenetic age acceleration in people living with HIV‐1 pre‐ART (Figure 4a,b). We found a significant epigenetic age acceleration of mean average 2.8 years in people living without HIV‐1 compared to epigenetic age difference from chronological age of −0.19 years in healthy controls (Figure 4b). Prior work has shown several FDA approved antiretroviral drugs can effectively inhibit a family of endogenous retroviruses found in the human genome (HERV‐K) (Tyagi et al., 2017). Hence, we hypothesized that antiretroviral therapy utilized to treat HIV‐1 would reduce retroelement‐based epigenetic age. We found a significant reduction in retroelement‐based epigenetic age following 96 weeks of antiretroviral therapy comparing longitudinal samples of people living with HIV‐1 at pre‐ and post‐ART timepoints (Figure 4c).

### 2.6 Cell‐type specific transient reprogramming‐induced rejuvenation strategies utilizing Yamanaka factors reverse Retroelement‐based epigenetic clocks

**FIGURE 5:**
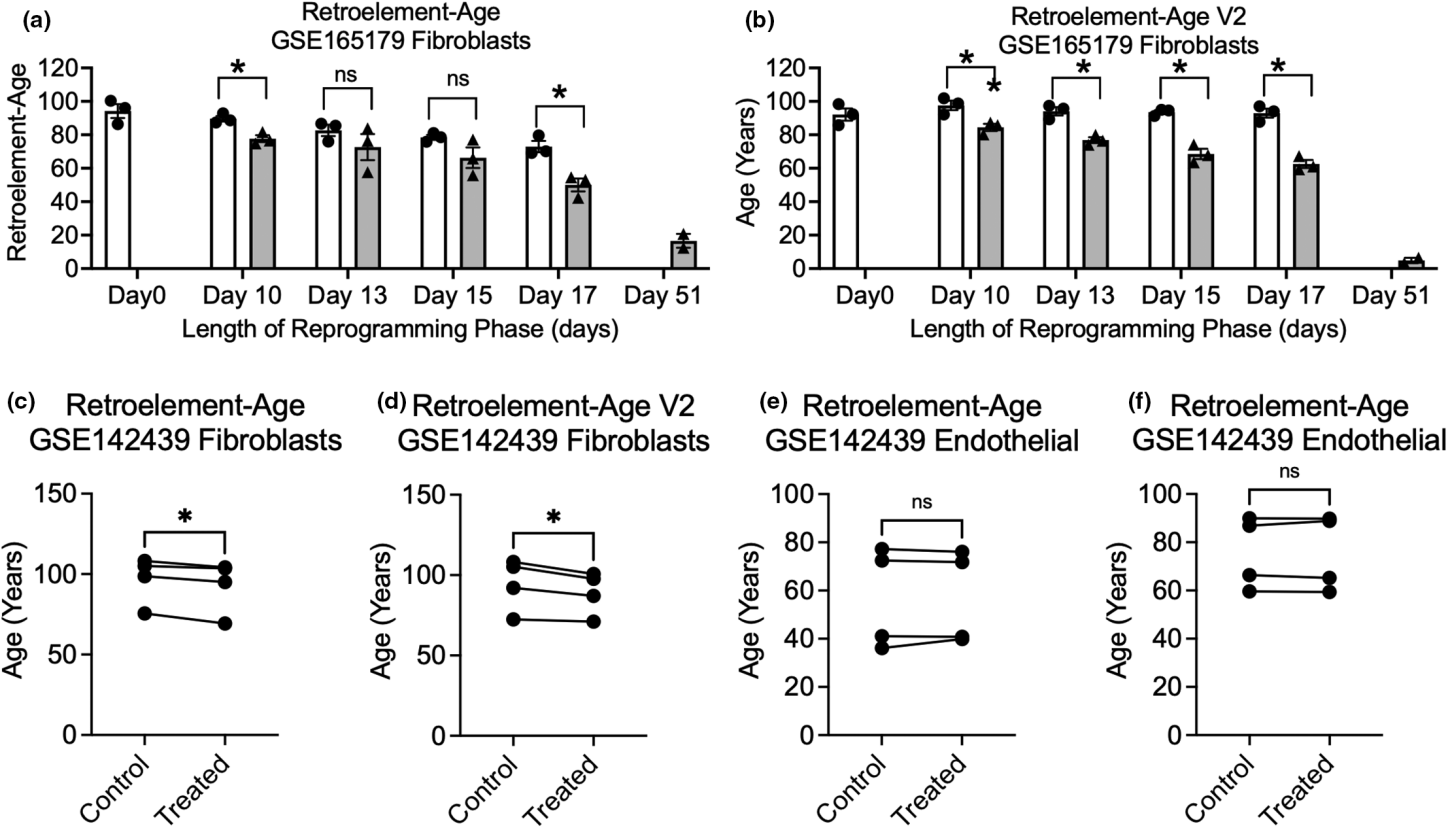
Application of retroelement‐based epigenetic clocks to transient reprogramming‐induced rejuvenation. (a) Retroelement‐Age V1 estimates of fibroblasts transiently reprogrammed at Day 0–51. (b) Retroelement‐Age V2 estimates of fibroblasts transiently reprogrammed at Day 0–51. (c, d) Retroelement‐Age V1 and V2 estimates of fibroblasts transiently reprogrammed. (e, f) Retroelement‐Age V1 and V2 estimates of endothelial cells transiently reprogrammed. Student's T tests and paired T tests, * *p* < 0.05.

Transient reprogramming has emerged as a controversial strategy to epigenetically rejuvenate cells as an anti‐aging strategy (Gill et al., 2022; Levine et al., 2022). A key outcome measures utilized to assess epigenetic rejuvenation through transient reprogramming has been epigenetic clocks. However, findings suggests some clocks may not have utility in assessing epigenetic rejuvenation achieved by transient reprogramming (Gill et al., 2022; Levine et al., 2022). Based on prior work showing HERVs are a key mechanism involved in human iPSC generation and re‐establishment of differentiation potential of induced pluripotent stem cells (iPSCs) (Ohnuki et al., 2014), we sought to examine whether our retroelement‐based epigenetic clocks would inform transient reprogramming‐induced rejuvenation strategies. We leveraged a published epigenetic rejuvenation DNA methylation dataset (Gill et al., 2022) GSE165179 and calculated epigenetic ages for control and transiently reprogrammed fibroblasts at Day 0, 10, 13, 15, and 17. Retroelement‐Age was significantly reversed in reprogrammed cells compared to control cells at Day 10 and Day 17 of the dataset (Figure 5a). Retroelement‐Age V2 was significantly reversed in reprogrammed cells compared to control cells at Day 10, 13, 15, and 17 (Figure 5b). Next, we applied our retroelement‐age clocks to GSE142439 that included transient expression of reprogramming factors in fibroblasts and endothelial cells from aged and young human participants (Sarkar et al., 2020). We found Retroelement‐Age and Retroelement‐Age V2 was significantly reversed in fibroblasts (Figure 5c,d). However, according to our retroelement‐based epigenetic clocks we did not observe a rejuvenation effect in endothelial cells suggesting the importance of considering cell‐type specific responses in transient reprogramming‐induced rejuvenation strategies (Figure 5e,f). Our findings suggest retroelement‐based epigenetic clocks as an additional outcome measure to consider in ongoing transient reprogramming efforts and anti‐aging strategies (Gill et al., 2022). Whether a new transient‐native‐treatment (TNT) reprogramming strategy shown to correct aberrant transposable element expression impacts retroelement‐based epigenetic clocks remains unclear (Buckberry et al., 2023).

### 2.7 Immune transcriptome telescope‐age

Evidence suggest that age‐related repetitive element transcript accumulation and changes in DNA methylation states may play a role in inflammaging (Smith et al., 2024) and transcriptomic age signatures have been shown to offer a complementary predictor of biological aging (Bulteau & Francesconi, 2022). Hence, we expanded upon our DNA methylation retroelement‐based epigenetic clocks and evaluated whether age‐related RNA expression of locus‐specific retroelements in the immune system could be utilized to develop a transcriptome retroelement‐based predictor of chronological age. We utilized the Telescope computational pipeline (Bendall et al., 2019) to estimate HERV and LINE element expression resolved to specific genomic locations in an independent RNA‐Seq dataset of blood from 157 human donors spanning chronological ages 20–74 year (GEO: GSE193141) (Morandini et al., 2024) (Figure S3). Using a generalized linear elastic net model fit with 10‐fold cross‐validation on an 80% training and 20% test dataset using both HERV and LINEs expression in blood, we found that the expression levels of 95 retroelements (Table S4) were predictive of chronological age in both training (Pearson's *r* = 0.98, MAE = 4.22) and test datasets (Pearson's *r* = 0.62, MAE = 9.99) (Figure S3). Among the elements, we observed 41 LINEs, supporting hypotheses regarding the resurrection of particular LINEs in the aging process (De Cecco et al., 2019; Liu et al., 2023; Zhang et al., 2023). Integrating loci from our retroelement‐based epigenetic clocks, we found overlap of age‐associated DNA methylation and RNA changes in ERVLE_1q25.3d, L1FLnI_11p13w, and L1FLnI_6q12d (Figure S3). These findings suggest that distinct retroelement transcriptome profiles can be utilized as a predictor of chronological age and that a subset of the loci identified in retroelement‐based epigenetic DNA methylation clocks may relate to transcription of specific retroelements.

### 2.8 Evidence of human multi‐tissue Retroelement clock

**FIGURE 6:**
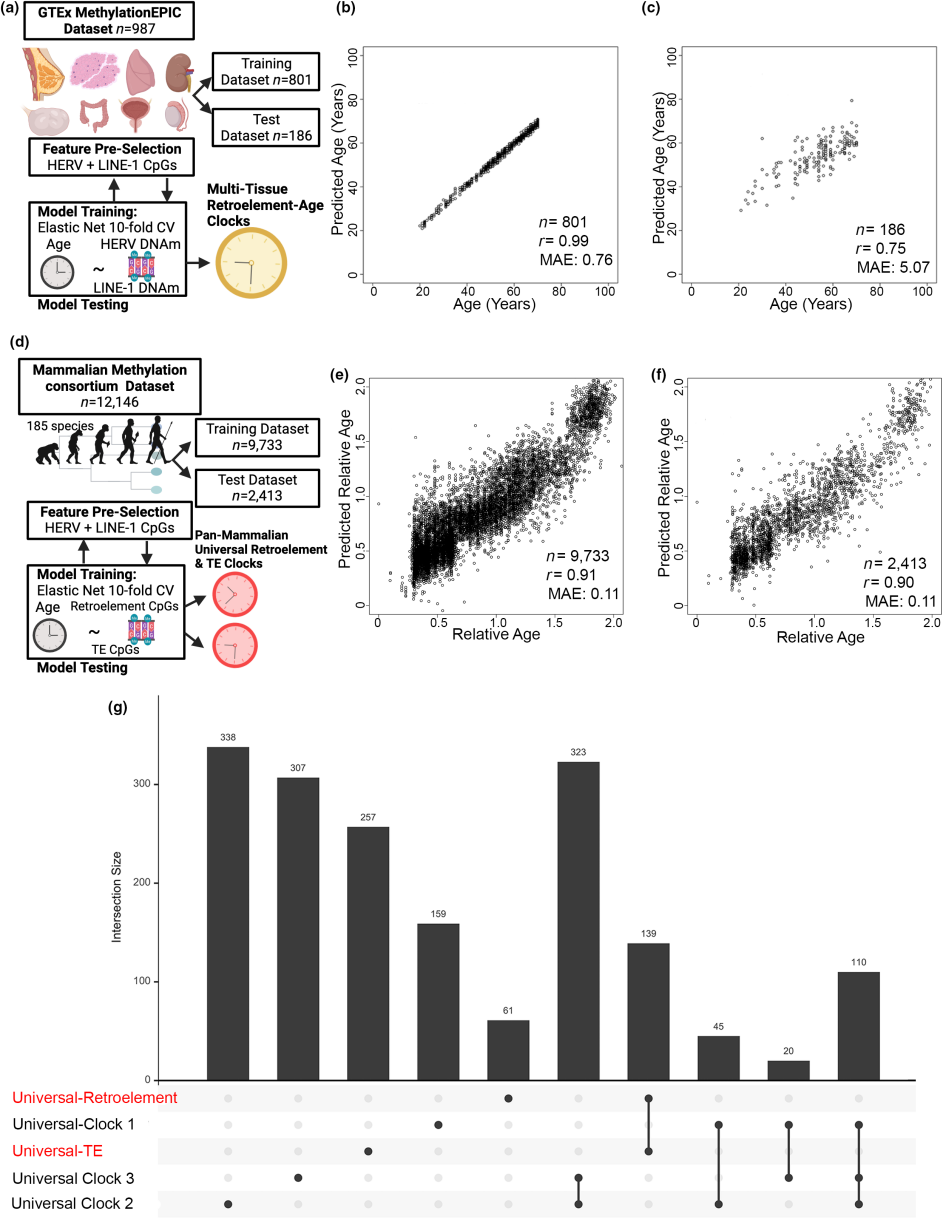
Construction of Multi‐Tissue and Pan‐Mammalian Retroelement‐based Epigenetic Clocks. (a) Multi‐Tissue RetroAge GTEx project data GSE213478. (b) Age estimation 10 fold cross validation in training and (c) test datasets for Multi‐Tissue RetroAge GTEx project data GSE213478. (d) Construction of a unique Pan‐Mammalian Retroelement‐based Epigenetic Clock based on data from the international Mammalian Methylation consortium (Lu et al., 2023). (e) Age estimation 10‐fold cross validation in training and (f) test datasets. Panels report the sample size (*n*), the median absolute error (MAE), and Pearson's correlation coefficient (*r*). (g) Intersection plot of published Pan‐mammalian Universal epigenetic clocks CpGs, Universal‐Retroelement Age, and Universal Transposable Element (TE) Age.

To demonstrate DNA methylation states of retroelements could be utilized beyond the immune system to capture human aging, we utilized Genotype‐Tissue Expression (GTEx) DNA methylation data for 987 human samples from nine tissue types spanning breast mammary tissue, muscle skeletal, lung, ovary, kidney, testis, prostate, colon, and whole blood (Oliva et al., 2023). To test whether there was evidence of a human multi‐tissue retroelement clock, we utilized an expanded list of 74,577 retroelement CpGs based on HERV and LINE‐1 annotations and constructed a new composite retroelement‐based epigenetic clock (Retroelement‐TissueAge) based on a generalized linear elastic net model fit with 10‐fold cross‐validation on an 80% training and 20% validation dataset (Figure 6a). Since the GTEx DNA methylation dataset consisted of multiple tissues from the same donor, we sought to minimize data leakage and ensured each donor was only in one of the sets (either training or validation). We developed a Multi‐Tissue Retroelement‐based epigenetic clock that consisted of 734 CpG sites (Table S5) and was highly predictive of chronological age in both training (Pearson's *r* = 0.99, MAE = 0.76) and test datasets (Pearson's *r* = 0.75, MAE = 5.07) (Figure 6b,c).

### 2.9 Evidence of pan‐mammalian species multi‐tissue Retroelement clock

Last, we sought evidence of an evolutionarily conserved universal retroelement‐based epigenetic clock predicting chronological age. Recent data from the international Mammalian Methylation consortium demonstrated the development of universal pan‐mammalian clocks using DNA methylation data from over 11,000 samples spanning 59 tissue types and across 185 mammalian species using the HorvathMammalMethylChip40 (Mammal40) array, a mammalian array that targets over 36,000 conserved CpGs across mammalian species (Arneson et al., 2022; Lu et al., 2023). However, the specific CpGs utilized to construct these pan‐mammalian clocks did not focus on CpGs located within retroelements or other transposable elements. Hence, we first sought to evaluate whether the Mammal40 platform contained probes overlapping with transposable elements. We used RepeatMasker annotated probes in the Mammal40 platform and identified 563 loci in the class of DNA, LINE, LTR, SINE, and Unknown. Next, we utilized the pan‐mammalian DNA methylation dataset consisting of 12,146 samples and relative ages calculated as log transformed chronological ages with an offset of 2 years added as utilized in the construction of the original development of universal pan‐mammalian clocks (Lu et al., 2023). First, we utilized all 563 DNA, LINE, LTR, SINE, and unknown CpGs identified by RepeatMasker annotated probes and developed a pan‐mammalian species clock consisting of 398 CpG sites (Table S6) that was highly predictive of chronological age in both training (Pearson's *r* = 0.94) and test datasets (Pearson's *r* = 0.94). Next, we only used the 211 CpGs in LINEs and LTR and found we could also construct a highly predictive pan‐mammalian species clock based on only 201 CpGs in HERVs and LINEs (Table S7) covered in the dataset (Figure 6d–f). We examined the intersection of the CpGs utilized in our pan‐mammalian TransAge and RetroAge clocks with the published three universal pan‐mammalian clocks (Lu et al., 2023). Notably, we found no overlap with CpGs utilized to construct the currently available pan‐mammalian epigenetic clocks (Figure 6g), suggesting additional insights into the evolutionary components of aging‐associated processes not previously detected.

## 3 DISCUSSION

The development of a myriad of epigenetic clocks based on distinct DNA methylation patterns have emerged as promising candidate biomarkers warranting further validation to quantify chronological and biological age for the geroscience field (Moqri et al., 2024). A subset of epigenetic clocks have also been shown to have utility in predicting aging‐related health outcomes and proposed as complementary outcome measures for geroscience clinical trials (Moqri et al., 2023). While epigenetic clock‐based measures have been linked to specific hallmarks of aging (Kabacik et al., 2022), the role of epigenetic clocks in mechanisms of aging linked to retroelements has not been a major focus. Here, we show the potential of DNA methylation states in HERVs and LINEs as highly accurate epigenetic clocks predicting chronological age, supporting the hypothesis that a subset of specific retroelements in the human genome may be involved in aging. Using only HERV and LINE‐1 DNA methylation states, we developed highly accurate retroelement‐based epigenetic clocks that minimally overlapped with preexisting first and second‐generation epigenetic clocks. We find evidence of clock CpGs located in HERV elements previously linked to aging and immune function, such as HERV‐K and MER41. We also captured retroelement‐based epigenetic clock CpGs in novel retroelements not previously linked to aging, suggesting a broader role of locus‐specific retroelements in biological aging. Applying these retroelement‐based epigenetic clocks to DNA methylation datasets, we find that retroelement clocks are reversed during transient epigenetic reprogramming (Gill et al., 2022), accelerated in people living with HIV (Esteban‐Cantos et al., 2023), and responsive to antiretroviral therapy (Esteban‐Cantos et al., 2023). Finally, we demonstrate that retroelement clocks extend to diverse human tissues and across mammalian species using DNA methylation from GTEx (Oliva et al., 2023) and from the Mammalian Methylation Consortium (Lu et al., 2023). Together, these findings support the hypothesis of dysregulation of endogenous retroelements as a potential contributor to the biological hallmarks of aging and suggest that therapeutic interventions modifying the epigenetic states of specific retroelements in the human genome could have beneficial effects against a root cause of aging and disease. Additionally, these studies suggest that retroelement‐based epigenetic clocks are evolutionarily conserved throughout mammals, and that further study of which throughout post‐speciation events could provide valuable insight into the evolutionary components of aging‐associated processes and the impact of Paleovirology on each species' lifespan.

Emerging evidence suggests a correlation between aging, chronic diseases, and the reactivation of specific retroelements, primarily LINEs and HERV‐K‐derived retrovirus like particles (RVLPs). Prior work has shown that the transcriptional derepression of LINEs in immune cells triggers interferon production to contribute to inflammaging (Buttler et al., 2023; De Cecco et al., 2019; Della Valle et al., 2022; Marasca et al., 2022). Recent findings have suggested a key role of the endogenous retrovirus HERV‐K (HML‐2) activation in cellular senescence and tissue aging (Liu et al., 2023). Higher expression of transposable elements and HERVs has been shown to trigger renal firbroinflammation and found to be associated with diseased human kidneys (Dhillon et al., 2023). Data from Drosophila models of aging have revealed that stimulating retrotransposon activity increases mortality and accelerates a subset of aging phenotypes (Rigal et al., 2022). Our data extend these findings and suggest DNA methylation changes of locus‐specific retroelements may play a key role in immune aging and potentially eliciting biological hallmarks of aging in the immune system.

A notable observation was that the set of CpGs that were utilized to construct our retroelement‐based epigenetic clocks did not show a significant overlap with pre‐existing first and second‐generation epigenetic clock algorithms (Belsky et al., 2022; Horvath, 2013; Levine et al., 2018; Lu, Quach, et al., 2019). Differences between clocks have arisen from the focus on DNA methylation data compatibility, considerations of probe reliability, datasets utilized, and being trained to predict different aging‐related variables, such as chronological age, composite biomarkers of aging, mortality risk, or mitotic divisions. Our observation of minimal overlap between our retroelement‐based clocks and prior epigenetic clocks may be due to our retroelement‐based clocks being developed on CpGs found on the EPIC platform. However, we observed that examination of all 4466 retroelement related CpGs common to the EPIC and 450 K arrays did not show high overlap with prior first‐, second‐, and third‐generation epigenetic clocks developed largely on 450 K CpGs, suggesting that retroelement‐based epigenetic clocks capture previously undetected facets of biological aging to complement current epigenetic clocks. Epigenetic clocks should be applied to the proper context and appear to capture distinct aspects of aging and associate with different biological hallmarks of aging, environmental exposures, traits, and disease patterns (Higgins‐Chen et al., 2021; Horvath & Raj, 2018; Liu et al., 2020; Oblak et al., 2021).

We found that using a limited set of CpGs from the HorvathMammalMethylChip40 (Mammal40) array that only contained 643 loci based on RepeatMasker in the class of DNA, LINE, LTR, SINE, and Unknown, we were able to construct a highly accurate universal mammalian retroelement methylation clock based on 220 CpGs. Less than 3% (6 CpGs) overlapped with CpGs utilized to construct the 3 Universal Pan‐Mammalian methylation clocks (Lu et al., 2023). Our additional findings to those reported by Lu et al. suggest an evolutionarily conserved dysregulation of DNA methylation states within retroelements may play a role beyond mammalian development into aging (Garcia‐Perez et al., 2016). Interestingly, beyond our universal retroelement‐based epigenetic clocks, we were able to construct an accurate retroelement methylation clock for naked mole‐rats, which have long lifespans and reduced transposon‐derived sequences (Kim et al., 2011). The differences and interactions between retroelements that promote aging and those that confer anti‐aging benefits merit further investigation. Models with extended lifespans could prove useful in exploring this hypothesis.

Due to the likely increase in EPIC v.2.0 data for the geroscience field, we developed a EPIC v.1.0 and v.2.0 compatible retroelement‐based epigenetic clock (Retroelement‐Age V2) that contains CpGs covered on Illumina's MethylationEPIC v2.0 kit. An interesting feature of Retroelement‐Age V2 is that none of the CpGs utilized to construct this clock overlapped with nine existing first and second‐generation epigenetic clocks. We found a significant enrichment of 80 CpGs in this clock at CCCTC‐binding factor (CTCF) sites of the genome. These findings suggest that DNA methylation changes in retroelements, particularly at CTCF (CCCTC‐binding factor) binding sites, may potentially play a role in aging. CTCF is a critical protein involved in the organization of chromatin structure and the regulation of gene expression. It acts as an insulator, helping to define boundaries between chromatin domains and regulate the accessibility of genes to the transcriptional machinery. Our findings support prior DNA methylation work showing changes with aging observed in CTCF binding sites (Reynolds et al., 2014) and a pan‐tissue DNA methylation epigenetic clock based on deep learning that found that the most important CpG sites were proximal to CTCF binding sites (de Lima Camillo et al., 2022). Future work will need to examine whether modifying DNA methylation states at retroelements overlapping with CTCF binding impacts immune aging by changes in transcriptional regulation, insulation of chromatin domains, and the organization of higher‐order chromatin structure.

Prior epigenetic clocks detect accelerated aging effects related to infection with the exogenous retrovirus HIV‐1 (Gross et al., 2016; Horvath & Levine, 2015; Horvath, Stein, et al., 2018; Leung et al., 2017; Shiau et al., 2021). People living with HIV‐1 are a population who exhibit increased features of biological aging (Cole et al., 2017; Guaraldi et al., 2009; Justice, 2010), likely due to infection from the exogenous retrovirus HIV‐1, chronic inflammation, antiretroviral therapy, and lifestyle effects (Deeks et al., 2013; Goulet et al., 2007; High et al., 2012). Prior work has shown that HIV‐1 infection activates certain HERVs including HERV‐K. This activation may be an additional factor contributing to accelerated/attenuated aging in people living with HIV related to immune dysfunction, inflammation, and immunosenescence. Prior epigenetic clocks did not provide insights into whether altered DNA methylation states of HERVs or LINEs related to HIV‐1 infection. By applying our retroelement‐based epigenetic clocks, we find evidence that suggest an increase in the epigenetic age compared to chronological age related to HIV, supporting the hypothesis that retroviruses may accelerate biological aging. Additionally, examining longitudinal data from people living with HIV‐1 (PLWH) receiving antiretroviral therapy (Esteban‐Cantos et al., 2021), we find that antiretroviral therapy treatment significantly reverses HIV‐1‐related increased retroelement‐based epigenetic age. A portion of this effect due to antiretroviral therapy may reflect CD^4+^ T cell immune restoration in people living with HIV‐1. Yet, whether antiretroviral therapy can be used as therapeutic to improve health and increase lifespan by reversing retroelement‐based epigenetic age in the absence of exogenous retroviral infections therefore warrants further investigation. Analysis of the effects of pre‐exposure prophylaxis (PrEP), which delivers antiretroviral drugs to reduce the risk of HIV infection, on retroelement‐based epigenetic age can uncover potential effects of antiretroviral drugs in HIV‐negative people on aging.

A limitation of our findings is that our study primarily focused on development of retroelement‐based epigenetic clocks from a large DNA methylation dataset from the human immune system. While we included DNA methylation data from GTEx samples across nine tissues, this dataset was limited and did not permit the construction of systems‐specific retroelement‐based epigenetic clocks. Our application of retroelement‐based epigenetic clocks across mammalian species was also limited to highly conserved CpG DNA sequences and could benefit from a species‐specific analysis that considers uniquely arisen CpG sites in respective species genomes. We suspect future work will improve upon retroelement‐based epigenetic clocks and include cell‐type and organ‐specific clocks.

In summary, these findings highlight the potential of DNA methylation states of specific retroelements as reliable predictors of chronical and potentially biological aging, complementing existing epigenetic clocks and offering an additional mechanism to consider in epigenetic clock signals. Collectively, our results suggest a renewed emphasis on the role of retroelements in human aging and warrants further study on their undefined roles in geroscience.

## 4 MATERIALS AND METHODS

### 4.1 Discovery cohort

The TruDiagnostic Biobank cohort, previously described in (Chen et al., 2023), included 13,109 individuals who took the commercial TruDiagnostic TruAge test and had their DNA methylation data generated from whole blood. The participants were recruited between October 2020 and April 2023 and were predominantly from the United States.

### 4.2 MethylationEPIC V1.0 DNA methylation pre‐processing and analysis

Peripheral blood samples were collected using a lancet and capillary method and placed in a lysis buffer for DNA extraction. Then, 500 ng of DNA was treated with bisulfite using the EZ DNA Methylation kit from Zymo Research following the manufacturer's instructions. The bisulfite‐treated DNA samples were randomly assigned to a well on the Infinium HumanMethylationEPIC BeadChip, which was then amplified, hybridized, stained, washed, and imaged with the Illumina iScan SQ instrument to obtain raw image intensities. To pre‐process the TruDiagnostic methylation data, we used the *minfi* pipeline (Aryee et al., 2014), and low quality samples were identified using the *qcfilter()* function from the ENmix package (Xu et al., 2016), using default parameters. A total of 12,670 individuals, representing 96.7% of the original samples, passed the QA/QC (*p* < 0.05) and were deemed to be high quality samples.

### 4.3 Composite Retroelement clocks construction

CpGs covered on the Illumina Infininium MethylationEPIC (EPIC) V1 array were filtered using a manually curated locus‐specific HERV annotation of 60 HERV families (Bendall et al., 2019). Using the Telescope database, we identified 10,917 probes, accounting for 1.26% of the EPIC v1.0 array, that assess DNA methylation at CpG sites located within HERV transcriptional units or active LINEs (Bendall et al., 2019). The locus specific HERV and LINE‐1 annotations were then used to filter two beta matrixes of CpGs for all 12,670 samples. The caret R package was used to load metadata for chronological age of all 12,670 samples. The composite HERV and LINE‐1 filtered beta matrix was then split into 80% training and 20% validation datasets. The glmnet R package was then used to train models for Retroelement‐Age V1 clock with a 10‐fold validation utilizing an elastic net. Retroelement‐Age V2 extended our manually curated annotation of Telescope‐based EPIC v.1.0 CpGs to overlapping probes on the EPIC v2.0 platform. We also expanded our retroelement CpGs for EPIC v2.0 to include LTR and LINE elements identified by RepeatMasker. Retroelement CpG datasets utilized for Retroelement‐Age V1 and V2 are available at Zenodo at DOI: 10.5281/zenodo.11099870.

### 4.4 Multi‐tissue and pan‐mammalian species Retroelement clocks construction

Retroelement filtered beta matrixes were for a GTEx dataset of 987 samples (GSE213478) and Pan‐Mammalian Species dataset of 12,146 samples (GSE223748) were split into 80% training and 20% validation datasets. Chronological ages and log transformed chronological ages with an offset of 2 years added were used. The glmnet R package was then used to train models for clocks with a 10‐fold validation utilizing an elastic net.

### 4.5 CpG Feature Enrichment

The knowYourCG tool was utilized for examining CpG feature enrichment using Illumina probe IDs and databases associated with certain CpGs available at https://github.com/zhou‐lab/KYCG_knowledgebase_EPIC/tree/main/Studies. Fisher's exact test was utilized to test CpG enrichments. Enrichment results were visualized using the KYCG_plotDot function in SeSAMe.

### 4.6 Public datasets

Data used for validation and testing of retroelement clocks was publicly available via Gene Expression Omnibus (GEO). MethylationEPIC V1 array IDATs were downloaded from GEO using the following accession numbers: GSE165179, GSE184269, GSE213478, GSE223748, GSE142439. All GTEx protected data was accessed via the GTEx Portal.

### 4.7 Epigenetic clock and cell type deconvolution

Published epigenetic clocks were calculated according to published methods from processed DNA methylation data. To calculate the principal component‐based epigenetic clock for the Horvath multi‐tissue clock, Hannum clock, DNAmPhenoAge clock, GrimAge clock, and telomere length we used the custom R script available via GitHub (https://github.com/MorganLevineLab/PC‐Clocks). Non‐principal component‐based (non‐PC) Horvath, Hannum, and DNAmPhenoAge epigenetic metrics were calculated using the *methyAge* function in the ENMix R package. The pace of aging clock, DunedinPACE, was calculated using the *PACEProjector* function from the DunedinPACE package available via GitHub (https://github.com/danbelsky/DunedinPACE). We used a 12 cell immune deconvolution method to estimate cell type proportions (Zheng et al., 2018).

## AUTHOR CONTRIBUTIONS

Conceptualization: LCN, RS, MJC. Methodology: MB, APP, MJC, NC, VD. Investigation: LCN, MB, VD, APP, RS, MJC, NC, VD. Supervision: LCN, MJC. Writing—original draft: MJC. Writing—review & editing: LCN, MJC, ND, DN.

## FUNDING INFORMATION

National Institutes of Health grant UM1AI164559 (LCN, DN, MC). National Institutes of Health grant R56 AG078970 (DN). National Institutes of Health grant R01DA052027 (LCN, DN).

## CONFLICT OF INTEREST STATEMENT

LCN has served as a scientific advisor for Abbvie, ViiV and Cytodyn for work unrelated to this project. MJC and LCN are listed co‐inventors on pending patents relating to work disclosed in this manuscript. VD, NC, and RS are employees of TruDiagnostic. All other authors declare no other competing interests.

## Supporting information


Data S1.

Figure S1.

## Data Availability

The retroelement DNA methylation data utilized to construct retroelement‐based epigenetic clocks can be found on Zenodo at DOI: 10.5281/zenodo.11099870. The code, retroelement‐based clock coefficients, and script to calculate retroelement epigenetic ages from any DNA methylation EPIC v.1.0 or 2.0 dataset can be found on Zenodo at DOI: 10.5281/zenodo.11099870. The complete discovery cohort data can be provided by TruDiagnostic pending scientific review and a completed material transfer agreement. Requests for the discovery cohort data should be submitted to: Ryan Smith, ryan@trudiagnostic.com
