# Supplementary material for: Retro‐age: A unique epigenetic biomarker of aging captured by DNA methylation states of retroelements
Source: Aging Cell. 2024 Aug 2;23(10):e14288. doi: 10.1111/acel.14288 (PMC11464121; doi:10.1111/acel.14288)
Supplement: Supplementary file 2 — Figure S1. [file ACEL-23-e14288-s002.docx]

Supplementary Materials for

**Retro-Age: A Unique Epigenetic Biomarker of Aging Captured by DNA methylation States of Retroelements**

Lishomwa Ndhlovu *et al.*

*Corresponding author. Michael J. Corley; Email: [mjc4002@med.cornell.edu](mailto:mjc4002@med.cornell.edu)

**This PDF file includes:**

**Supplemental Figure 1. External validation of retroelement-based epigenetic clocks.**

**Supplemental Figure 2. Reliability of retroelement-based epigenetic clock.**

**Supplemental Figure 3. Integration of Age-related transposable element expression and retroelement-based DNA methylation epigenetic clocks**

Table S1.

**Composite Retroelement-Age CpGs**

Table S2.

**Composite Retroelement-Age-450k CpGs**

Table S3.

**Composite Retroelement-Age V2 CpGs**

Table S4.

**Immune Transcriptome Retroelement-Age**

Table S5.

**Human Multi-Tissue Retroelement clock CpGs**

Table S6.

**Pan-Mammalian Species Multi-Tissue Transposable Element Clock CpGs**

Table S7.

**Pan-Mammalian Species Multi-Tissue Retroelement Clock CpGs**

**
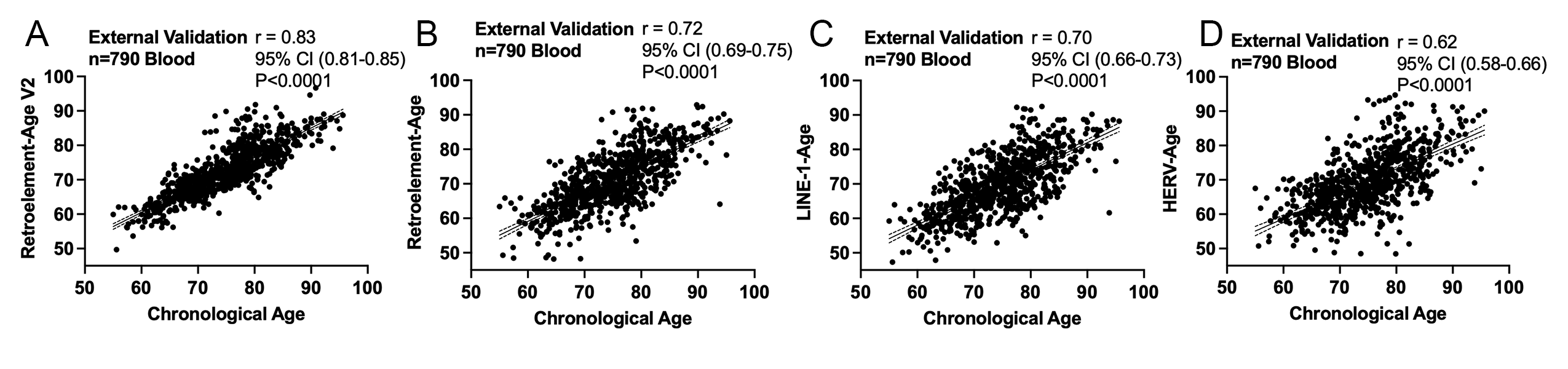
 Supplemental Figure 1. External validation of retroelement-based epigenetic clocks**. Scatterplots of **A.** Retroelement-Age V2, **B.** Retroelement-Age, and chronological age in 790 people. Independent data generated by the Alzheimer's Disease Neuroimaging Initiative study.

**Supplemental Figure 2. Reliability of retroelement-based epigenetic clock.** Intra-class correlation coefficient (ICC) of Retroelement-Age V2 on 30 replicate blood sample MethylationEPIC v1.0 samples from Independent data generated by the Alzheimer's Disease Neuroimaging Initiative study.


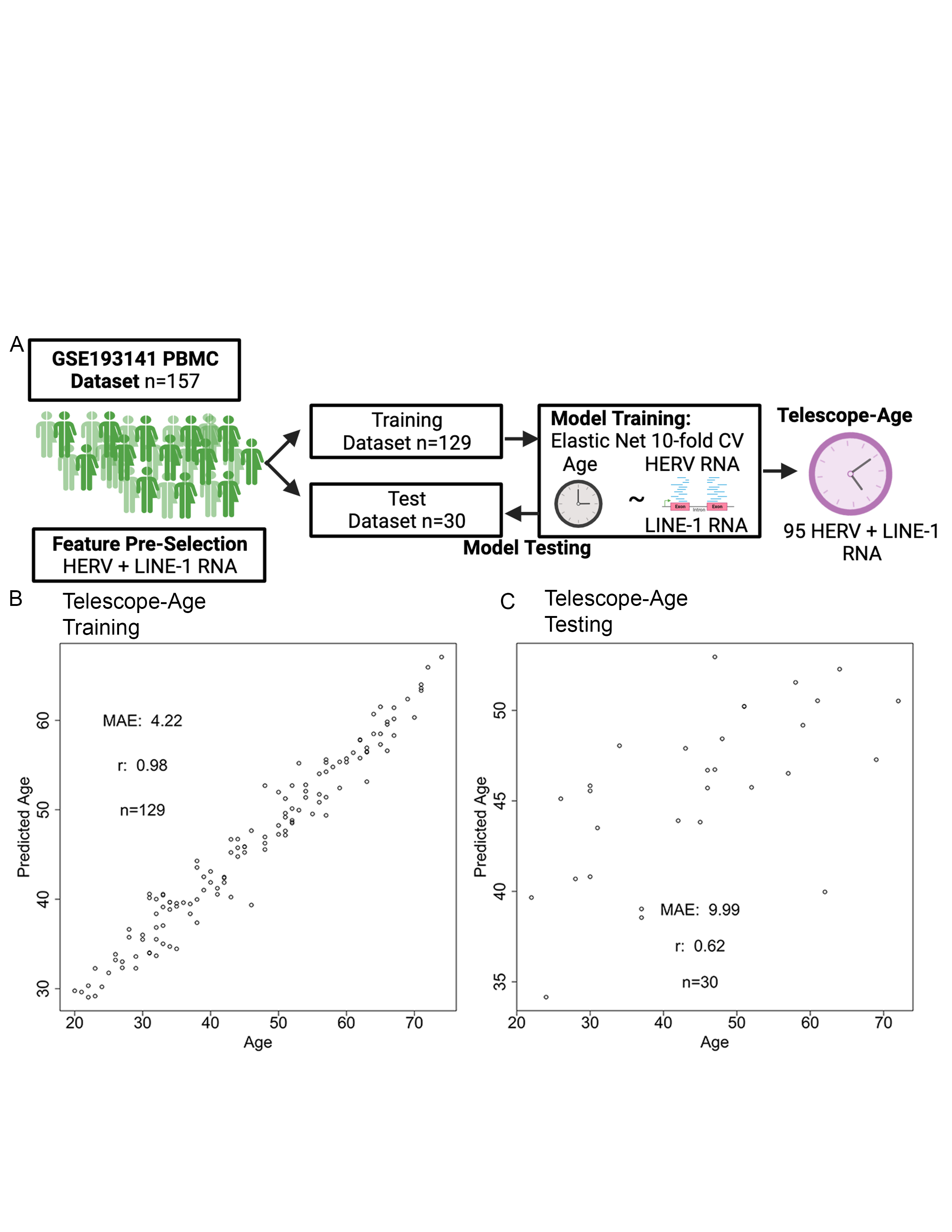


**Supplemental Figure 3. Integration of Age-related transposable element expression and retroelement-based DNA methylation epigenetic clocks. A.** Diagram of workflow utilized to construct immune transcriptome Telescope-Age. **B**. Age estimation 10-fold cross validation in training and **C.** test datasets for Telescope-Age.

Table S1.

**Composite Retroelement-Age CpGs**

| **ProbeID** | **Chr** | **Start** | **Stop** | **Retroelement** | **Coefficient** |
| --- | --- | --- | --- | --- | --- |
| Intercept |  |  |  |  | 78.13616296 |
| cg12576854 | chr8 | 72874544 | 72882593 | gene_id "L1FLI_8q21.11b" | 0.617954593 |
| cg05095427 | chr10 | 36892456 | 36902574 | gene_id "L1FLnI_10p11.21e" | -0.271662896 |
| cg10255679 | chr10 | 36892456 | 36902574 | gene_id "L1FLnI_10p11.21e" | -0.711053466 |
| cg08381168 | chr10 | 37991945 | 38001951 | gene_id "L1FLnI_10p11.21l" | -0.163047982 |
| cg10833451 | chr10 | 30145843 | 30155913 | gene_id "L1FLnI_10p11.23a" | 2.496919244 |
| cg12508651 | chr10 | 27414773 | 27424890 | gene_id "L1FLnI_10p12.1k" | -0.615195504 |
| cg21319613 | chr10 | 23506001 | 23515573 | gene_id "L1FLnI_10p12.2g" | -0.153724846 |
| cg02025136 | chr10 | 14742566 | 14751344 | gene_id "L1FLnI_10p13c" | 0.164893054 |
| cg17797731 | chr10 | 5124254 | 5132838 | gene_id "L1FLnI_10p15.1a" | 2.990713163 |
| cg14373685 | chr10 | 5230999 | 5241377 | gene_id "L1FLnI_10p15.1d" | 2.733604725 |
| cg19681266 | chr10 | 1220918 | 1231055 | gene_id "L1FLnI_10p15.3c" | -1.810046917 |
| cg15257565 | chr10 | 42139505 | 42149644 | gene_id "L1FLnI_10q11.21d" | 0.969134773 |
| cg05985455 | chr10 | 43914306 | 43924400 | gene_id "L1FLnI_10q11.21n" | -3.318284532 |
| cg24387129 | chr10 | 45296392 | 45305497 | gene_id "L1FLnI_10q11.21z" | 1.485796179 |
| cg05468077 | chr10 | 50781976 | 50792087 | gene_id "L1FLnI_10q11.23e" | 0.464607569 |
| cg27608610 | chr10 | 58530883 | 58540043 | gene_id "L1FLnI_10q21.1ra" | 1.44318294 |
| cg05812299 | chr10 | 55598377 | 55608410 | gene_id "L1FLnI_10q21.1y" | -0.440272451 |
| cg00132141 | chr10 | 67696134 | 67706291 | gene_id "L1FLnI_10q21.3ga" | -2.657046416 |
| cg03668579 | chr10 | 67696134 | 67706291 | gene_id "L1FLnI_10q21.3ga" | -0.580580114 |
| cg04141648 | chr10 | 67696134 | 67706291 | gene_id "L1FLnI_10q21.3ga" | -2.14489708 |
| cg07816205 | chr10 | 66582833 | 66591953 | gene_id "L1FLnI_10q21.3w" | -0.342857849 |
| cg06154432 | chr10 | 75558032 | 75568186 | gene_id "L1FLnI_10q22.2f" | -0.358117277 |
| cg10386292 | chr10 | 84276469 | 84286506 | gene_id "L1FLnI_10q23.1ga" | 1.067379632 |
| cg16105938 | chr10 | 84485245 | 84495376 | gene_id "L1FLnI_10q23.1ha" | -0.686601307 |
| cg09873703 | chr10 | 88299367 | 88309797 | gene_id "L1FLnI_10q23.31b" | 1.2468431 |
| cg13342026 | chr10 | 88314177 | 88322860 | gene_id "L1FLnI_10q23.31d" | 0.538603631 |
| cg19877430 | chr10 | 98659911 | 98670397 | gene_id "L1FLnI_10q24.2c" | 1.954737364 |
| cg16238635 | chr10 | 99171324 | 99181102 | gene_id "L1FLnI_10q24.2i" | -1.883773949 |
| cg19534837 | chr10 | 99409020 | 99418562 | gene_id "L1FLnI_10q24.2k" | -0.987788975 |
| cg16591000 | chr10 | 1.12E+08 | 112068902 | gene_id "L1FLnI_10q25.2f" | 0.455025718 |
| cg13412213 | chr10 | 1.18E+08 | 117842387 | gene_id "L1FLnI_10q26.11a" | 0.307233618 |
| cg18650367 | chr10 | 1.32E+08 | 132097374 | gene_id "L1FLnI_10q26.3d" | -0.836772414 |
| cg03230038 | chr10 | 1.33E+08 | 133151249 | gene_id "L1FLnI_10q26.3e" | -1.536786258 |
| cg09292354 | chr10 | 1.33E+08 | 133151249 | gene_id "L1FLnI_10q26.3e" | -10.70056605 |
| cg09699225 | chr10 | 1.33E+08 | 133151249 | gene_id "L1FLnI_10q26.3e" | -0.158933074 |
| cg13754569 | chr10 | 1.33E+08 | 133151249 | gene_id "L1FLnI_10q26.3e" | 0.094028359 |
| cg18635896 | chr10 | 1.33E+08 | 133151249 | gene_id "L1FLnI_10q26.3e" | -0.320412334 |
| cg20654619 | chr10 | 1.33E+08 | 133151249 | gene_id "L1FLnI_10q26.3e" | -0.08136447 |
| cg25120459 | chr10 | 1.33E+08 | 133151249 | gene_id "L1FLnI_10q26.3e" | -0.27278781 |
| cg06842946 | chr11 | 49039708 | 49049829 | gene_id "L1FLnI_11p11.12b" | -0.191921974 |
| cg02791145 | chr11 | 50252669 | 50261393 | gene_id "L1FLnI_11p11.12j" | 1.664423268 |
| cg07643616 | chr11 | 38015237 | 38024968 | gene_id "L1FLnI_11p12h" | 0.06939235 |
| cg02230376 | chr11 | 30050709 | 30060734 | gene_id "L1FLnI_11p14.1v" | 2.17597138 |
| cg08837192 | chr11 | 23728096 | 23738545 | gene_id "L1FLnI_11p14.3r" | 1.007603625 |
| cg14051890 | chr11 | 24151357 | 24160518 | gene_id "L1FLnI_11p14.3u" | -0.110686738 |
| cg01277177 | chr11 | 18655299 | 18665412 | gene_id "L1FLnI_11p15.1c" | 0.318920875 |
| cg02608602 | chr11 | 18939444 | 18949153 | gene_id "L1FLnI_11p15.1g" | -0.253100662 |
| cg00517605 | chr11 | 20338880 | 20349268 | gene_id "L1FLnI_11p15.1p" | 1.580653933 |
| cg24444810 | chr11 | 14083657 | 14093873 | gene_id "L1FLnI_11p15.2c" | -2.011799529 |
| cg11778480 | chr11 | 11710797 | 11720931 | gene_id "L1FLnI_11p15.3a" | 0.159725496 |
| cg16372767 | chr11 | 5474000 | 5484020 | gene_id "L1FLnI_11p15.4r" | 0.540396521 |
| cg14569837 | chr11 | 56029325 | 56038650 | gene_id "L1FLnI_11q12.1b" | 1.291941196 |
| cg21257081 | chr11 | 59886592 | 59895983 | gene_id "L1FLnI_11q12.1ba" | -0.935893995 |
| cg21822149 | chr11 | 57993664 | 58003687 | gene_id "L1FLnI_11q12.1m" | 4.688756998 |
| cg04424945 | chr11 | 63198345 | 63208073 | gene_id "L1FLnI_11q12.3i" | -2.539001223 |
| cg00289431 | chr11 | 75681658 | 75692138 | gene_id "L1FLnI_11q13.5a" | -2.733083491 |
| cg10314270 | chr11 | 76960335 | 76970455 | gene_id "L1FLnI_11q13.5f" | -2.883850941 |
| cg23450586 | chr11 | 86308061 | 86316573 | gene_id "L1FLnI_11q14.2a" | 1.125949866 |
| cg02703695 | chr11 | 89740689 | 89750714 | gene_id "L1FLnI_11q14.3j" | -0.249851533 |
| cg10054980 | chr11 | 89740689 | 89750714 | gene_id "L1FLnI_11q14.3j" | -0.111178956 |
| cg13007967 | chr11 | 94093512 | 94103660 | gene_id "L1FLnI_11q21g" | 0.518753886 |
| cg01627181 | chr11 | 1.02E+08 | 102378692 | gene_id "L1FLnI_11q22.2a" | 1.258553309 |
| cg11420764 | chr11 | 1.07E+08 | 107470420 | gene_id "L1FLnI_11q22.3y" | -2.810774382 |
| cg04297660 | chr11 | 1.11E+08 | 110757269 | gene_id "L1FLnI_11q23.1a" | -0.297059864 |
| cg10488006 | chr11 | 1.12E+08 | 112334651 | gene_id "L1FLnI_11q23.1c" | -4.158052646 |
| cg19400821 | chr11 | 1.16E+08 | 115973616 | gene_id "L1FLnI_11q23.3e" | -0.767960671 |
| cg06873345 | chr12 | 26309854 | 26320262 | gene_id "L1FLnI_12p11.23a" | 0.433386484 |
| cg20398005 | chr12 | 11905651 | 11915816 | gene_id "L1FLnI_12p13.2m" | 1.398488333 |
| cg06005133 | chr12 | 8148453 | 8157111 | gene_id "L1FLnI_12p13.31h" | 1.783246492 |
| cg21411014 | chr12 | 4505397 | 4515401 | gene_id "L1FLnI_12p13.32f" | 1.423650941 |
| cg22875510 | chr12 | 39204252 | 39214392 | gene_id "L1FLnI_12q12k" | -0.030304582 |
| cg00044501 | chr12 | 48319695 | 48329675 | gene_id "L1FLnI_12q13.11z" | -0.899708488 |
| cg04252562 | chr12 | 54882387 | 54892534 | gene_id "L1FLnI_12q13.2e" | -0.094828814 |
| cg02629586 | chr12 | 56943249 | 56953372 | gene_id "L1FLnI_12q13.3b" | 0.206414767 |
| cg09041389 | chr12 | 58470908 | 58481054 | gene_id "L1FLnI_12q14.1h" | -0.214745062 |
| cg26803963 | chr12 | 62286873 | 62296865 | gene_id "L1FLnI_12q14.1la" | 0.307015589 |
| cg00458268 | chr12 | 65548922 | 65558607 | gene_id "L1FLnI_12q14.3c" | -0.414674216 |
| cg00903534 | chr12 | 65548922 | 65558607 | gene_id "L1FLnI_12q14.3c" | -2.391438641 |
| cg09717936 | chr12 | 65548922 | 65558607 | gene_id "L1FLnI_12q14.3c" | 0.719610679 |
| cg18030084 | chr12 | 67896710 | 67906724 | gene_id "L1FLnI_12q15e" | 0.196378097 |
| cg08975197 | chr12 | 69772912 | 69782941 | gene_id "L1FLnI_12q15i" | 1.629353327 |
| cg13318506 | chr12 | 69772912 | 69782941 | gene_id "L1FLnI_12q15i" | 0.434338165 |
| cg14125530 | chr12 | 69935202 | 69945197 | gene_id "L1FLnI_12q15j" | -5.20383688 |
| cg03674826 | chr12 | 75516932 | 75527082 | gene_id "L1FLnI_12q21.2b" | 0.333335878 |
| cg15391813 | chr12 | 80283365 | 80293848 | gene_id "L1FLnI_12q21.31f" | 1.623789674 |
| cg04961225 | chr12 | 90930865 | 90940963 | gene_id "L1FLnI_12q21.33j" | 1.470234662 |
| cg13608630 | chr12 | 90930865 | 90940963 | gene_id "L1FLnI_12q21.33j" | 0.692599063 |
| cg18577326 | chr12 | 90930865 | 90940963 | gene_id "L1FLnI_12q21.33j" | 5.363317538 |
| cg19569093 | chr12 | 97720341 | 97730372 | gene_id "L1FLnI_12q23.1m" | 0.70826945 |
| cg13302587 | chr12 | 99614866 | 99625907 | gene_id "L1FLnI_12q23.1z" | 3.650887061 |
| cg01904977 | chr12 | 1.03E+08 | 103431606 | gene_id "L1FLnI_12q23.2g" | 0.68073648 |
| cg23180171 | chr12 | 1.13E+08 | 112627728 | gene_id "L1FLnI_12q24.13a" | -0.375554564 |
| cg16497431 | chr13 | 19001928 | 19011038 | gene_id "L1FLnI_13q12.11c" | 1.044351275 |
| cg17843665 | chr13 | 23775971 | 23786137 | gene_id "L1FLnI_13q12.12c" | -0.180094168 |
| cg15491131 | chr13 | 24338992 | 24348480 | gene_id "L1FLnI_13q12.12e" | -1.38417508 |
| cg17094718 | chr13 | 24338992 | 24348480 | gene_id "L1FLnI_13q12.12e" | 1.854085167 |
| cg04464316 | chr13 | 34741552 | 34751599 | gene_id "L1FLnI_13q13.2h" | -0.903862893 |
| cg25991569 | chr13 | 34741552 | 34751599 | gene_id "L1FLnI_13q13.2h" | 1.078105071 |
| cg03667040 | chr13 | 36007731 | 36017737 | gene_id "L1FLnI_13q13.3g" | -0.953650451 |
| cg18860329 | chr13 | 42770417 | 42780905 | gene_id "L1FLnI_13q14.11m" | -0.412374108 |
| cg22984446 | chr13 | 42770417 | 42780905 | gene_id "L1FLnI_13q14.11m" | -1.142032024 |
| ch.13.654879R | chr13 | 48197317 | 48207453 | gene_id "L1FLnI_13q14.2n" | -6.161262703 |
| cg15088912 | chr13 | 48412631 | 48423015 | gene_id "L1FLnI_13q14.2o" | 5.087544706 |
| cg24674269 | chr13 | 51509219 | 51519338 | gene_id "L1FLnI_13q14.3e" | 1.912566394 |
| cg14420000 | chr13 | 54280882 | 54291256 | gene_id "L1FLnI_13q14.3v" | -3.683062386 |
| cg24001856 | chr13 | 74235446 | 74245470 | gene_id "L1FLnI_13q22.1c" | 1.700316557 |
| cg20889904 | chr13 | 80087992 | 80097805 | gene_id "L1FLnI_13q31.1m" | -0.134288853 |
| cg09122588 | chr13 | 96769372 | 96779815 | gene_id "L1FLnI_13q32.1l" | -0.002825228 |
| cg07348854 | chr13 | 1.12E+08 | 111895385 | gene_id "L1FLnI_13q34e" | -1.60771168 |
| cg17291325 | chr13 | 1.12E+08 | 111895385 | gene_id "L1FLnI_13q34e" | -0.071750037 |
| cg23153481 | chr13 | 1.12E+08 | 111895385 | gene_id "L1FLnI_13q34e" | 1.727829598 |
| cg09998151 | chr14 | 32456844 | 32467254 | gene_id "L1FLnI_14q12ab" | -0.079415875 |
| cg10005490 | chr14 | 32850662 | 32860808 | gene_id "L1FLnI_14q12eb" | 0.598144222 |
| cg20971956 | chr14 | 27669561 | 27679615 | gene_id "L1FLnI_14q12y" | 0.670173889 |
| cg00625902 | chr14 | 38596508 | 38605792 | gene_id "L1FLnI_14q21.1m" | -0.372505646 |
| cg07320684 | chr14 | 39013673 | 39023827 | gene_id "L1FLnI_14q21.1q" | -1.918738266 |
| cg04147689 | chr14 | 39570176 | 39580655 | gene_id "L1FLnI_14q21.1x" | 0.000982545 |
| cg18494563 | chr14 | 47897682 | 47907704 | gene_id "L1FLnI_14q21.3f" | 0.043173524 |
| cg13349726 | chr14 | 51412675 | 51422810 | gene_id "L1FLnI_14q22.1a" | -2.155347312 |
| cg27455021 | chr14 | 61054620 | 61064648 | gene_id "L1FLnI_14q23.1ba" | 5.906506731 |
| cg13240109 | chr14 | 58920099 | 58929538 | gene_id "L1FLnI_14q23.1j" | 0.737314068 |
| cg18158739 | chr14 | 63299861 | 63308530 | gene_id "L1FLnI_14q23.2l" | -3.383406062 |
| cg11731525 | chr14 | 65854369 | 65863786 | gene_id "L1FLnI_14q23.3b" | -0.072277572 |
| cg12326919 | chr14 | 66524007 | 66534421 | gene_id "L1FLnI_14q23.3g" | 0.597587788 |
| cg11721026 | chr14 | 66657140 | 66665791 | gene_id "L1FLnI_14q23.3i" | 4.651582041 |
| cg25613541 | chr14 | 94550189 | 94560760 | gene_id "L1FLnI_14q32.13b" | -1.314140354 |
| cg27082659 | chr14 | 95998060 | 96007472 | gene_id "L1FLnI_14q32.2a" | -3.82851687 |
| cg23948391 | chr15 | 19944720 | 19954869 | gene_id "L1FLnI_15q11.1a" | 0.1591198 |
| cg09096424 | chr15 | 21619841 | 21628515 | gene_id "L1FLnI_15q11.2c" | 0.007819216 |
| cg06021910 | chr15 | 37404353 | 37413526 | gene_id "L1FLnI_15q14k" | -5.148994258 |
| cg25550003 | chr15 | 39417072 | 39427088 | gene_id "L1FLnI_15q14z" | -4.753553096 |
| cg04726310 | chr15 | 44035059 | 44045419 | gene_id "L1FLnI_15q15.3b" | -0.995574817 |
| cg06336871 | chr15 | 44248536 | 44258549 | gene_id "L1FLnI_15q15.3c" | -0.270172996 |
| cg03278795 | chr15 | 44987645 | 44996475 | gene_id "L1FLnI_15q21.1b" | 0.970633903 |
| cg27073142 | chr15 | 45015780 | 45024723 | gene_id "L1FLnI_15q21.1c" | 0.227681232 |
| cg06103618 | chr15 | 69765935 | 69775695 | gene_id "L1FLnI_15q23c" | -7.528687859 |
| cg18272518 | chr15 | 71366794 | 71376497 | gene_id "L1FLnI_15q23l" | -0.889842637 |
| cg02096507 | chr15 | 71792524 | 71802056 | gene_id "L1FLnI_15q23m" | 0.388592925 |
| cg27528032 | chr15 | 73507947 | 73517970 | gene_id "L1FLnI_15q24.1c" | -2.52191294 |
| cg13848981 | chr15 | 81991668 | 82001696 | gene_id "L1FLnI_15q25.2h" | -0.719681422 |
| cg17700087 | chr15 | 85446879 | 85458130 | gene_id "L1FLnI_15q25.3d" | -0.069954529 |
| cg12956117 | chr15 | 86205333 | 86215477 | gene_id "L1FLnI_15q25.3e" | -2.328619173 |
| cg13570284 | chr15 | 87256286 | 87266206 | gene_id "L1FLnI_15q25.3t" | 1.284354035 |
| cg09884419 | chr15 | 89043405 | 89053407 | gene_id "L1FLnI_15q26.1b" | 1.336801552 |
| cg01704290 | chr15 | 94278386 | 94287293 | gene_id "L1FLnI_15q26.2c" | 1.465996893 |
| cg07694221 | chr15 | 94848420 | 94858438 | gene_id "L1FLnI_15q26.2e" | -1.124570439 |
| cg09239582 | chr15 | 94848420 | 94858438 | gene_id "L1FLnI_15q26.2e" | -0.306293939 |
| cg16668549 | chr15 | 94848420 | 94858438 | gene_id "L1FLnI_15q26.2e" | -2.478078615 |
| cg13097987 | chr15 | 1E+08 | 100292450 | gene_id "L1FLnI_15q26.3i" | 0.741194323 |
| cg02531193 | chr16 | 31567118 | 31575696 | gene_id "L1FLnI_16p11.2c" | 1.180238561 |
| cg06301178 | chr16 | 31567118 | 31575696 | gene_id "L1FLnI_16p11.2c" | 2.526104887 |
| cg07350501 | chr16 | 31567118 | 31575696 | gene_id "L1FLnI_16p11.2c" | 2.111029424 |
| cg08212545 | chr16 | 31567118 | 31575696 | gene_id "L1FLnI_16p11.2c" | -0.307544618 |
| cg24165048 | chr16 | 31783982 | 31792564 | gene_id "L1FLnI_16p11.2i" | -1.958087178 |
| cg16639172 | chr16 | 31821942 | 31831094 | gene_id "L1FLnI_16p11.2j" | -0.617566542 |
| cg06781853 | chr16 | 32837091 | 32846330 | gene_id "L1FLnI_16p11.2p" | 0.856761579 |
| cg26046150 | chr16 | 33645864 | 33655340 | gene_id "L1FLnI_16p11.2q" | 0.115007619 |
| cg01242044 | chr16 | 25552341 | 25562792 | gene_id "L1FLnI_16p12.1b" | 0.100931647 |
| cg15794017 | chr16 | 13086352 | 13096455 | gene_id "L1FLnI_16p13.12c" | -0.678606459 |
| cg00459119 | chr16 | 12069685 | 12079803 | gene_id "L1FLnI_16p13.13" | 8.11833139 |
| cg22101903 | chr16 | 47434212 | 47444407 | gene_id "L1FLnI_16q12.1c" | -1.564408347 |
| cg16519151 | chr16 | 48014967 | 48024989 | gene_id "L1FLnI_16q12.1h" | 1.220899133 |
| cg16418183 | chr16 | 51326311 | 51336454 | gene_id "L1FLnI_16q12.1p" | 0.293102173 |
| cg09458615 | chr16 | 55611953 | 55620729 | gene_id "L1FLnI_16q12.2f" | -0.121423404 |
| cg16752024 | chr16 | 64618851 | 64629569 | gene_id "L1FLnI_16q21ea" | 0.000983343 |
| cg07185006 | chr16 | 68582978 | 68593005 | gene_id "L1FLnI_16q22.1" | 1.41091956 |
| cg10949561 | chr16 | 72577300 | 72587432 | gene_id "L1FLnI_16q22.2g" | -1.176792894 |
| cg22705589 | chr16 | 73823902 | 73833931 | gene_id "L1FLnI_16q22.3" | -0.36844397 |
| cg21146765 | chr16 | 76626547 | 76636611 | gene_id "L1FLnI_16q23.1g" | -0.367615838 |
| cg00938771 | chr16 | 86229797 | 86239810 | gene_id "L1FLnI_16q24.1a" | 0.296038712 |
| cg16441627 | chr17 | 6856384 | 6864960 | gene_id "L1FLnI_17p13.1c" | 0.035498408 |
| cg13739541 | chr17 | 60483194 | 60491826 | gene_id "L1FLnI_17q23.2a" | 0.196353453 |
| cg13152584 | chr18 | 11054623 | 11063943 | gene_id "L1FLnI_18p11.21a" | -0.355349188 |
| cg00527908 | chr18 | 13893444 | 13902787 | gene_id "L1FLnI_18p11.21o" | -0.310641503 |
| cg12343754 | chr18 | 13893444 | 13902787 | gene_id "L1FLnI_18p11.21o" | -0.903101678 |
| cg11970037 | chr18 | 8883562 | 8893120 | gene_id "L1FLnI_18p11.22d" | 1.343306055 |
| cg20190746 | chr18 | 6160199 | 6170323 | gene_id "L1FLnI_18p11.31p" | 4.498285788 |
| cg01248520 | chr18 | 45690974 | 45701093 | gene_id "L1FLnI_18q12.3x" | -4.497122857 |
| cg03671431 | chr18 | 63936874 | 63947271 | gene_id "L1FLnI_18q22.1a" | 1.268718996 |
| cg19766763 | chr18 | 63936874 | 63947271 | gene_id "L1FLnI_18q22.1a" | 2.442469034 |
| cg02372905 | chr18 | 70232927 | 70242396 | gene_id "L1FLnI_18q22.2d" | 1.027184952 |
| cg21192698 | chr18 | 79494432 | 79503735 | gene_id "L1FLnI_18q23l" | 0.133380507 |
| cg27106643 | chr18 | 79494432 | 79503735 | gene_id "L1FLnI_18q23l" | -1.799641251 |
| cg16485140 | chr19 | 21973785 | 21982575 | gene_id "L1FLnI_19p12i" | 0.612903635 |
| cg10395758 | chr19 | 39645714 | 39655743 | gene_id "L1FLnI_19q13.2b" | 1.327243032 |
| cg27497255 | chr19 | 40909209 | 40918497 | gene_id "L1FLnI_19q13.2e" | 1.8173502 |
| cg09418601 | chr19 | 42768604 | 42778611 | gene_id "L1FLnI_19q13.2j" | -1.556055897 |
| cg25870663 | chr19 | 55818903 | 55828929 | gene_id "L1FLnI_19q13.43a" | 1.361576515 |
| cg02912379 | chr1 | 1.21E+08 | 120679929 | gene_id "L1FLnI_1p11.2b" | 0.973687125 |
| cg07831837 | chr1 | 1.21E+08 | 120679929 | gene_id "L1FLnI_1p11.2b" | -0.760108134 |
| cg22945471 | chr1 | 1.21E+08 | 121407969 | gene_id "L1FLnI_1p11.2d" | 0.032201991 |
| cg01558040 | chr1 | 1.16E+08 | 115643429 | gene_id "L1FLnI_1p13.1a" | 0.99242653 |
| cg04425506 | chr1 | 1.16E+08 | 115643429 | gene_id "L1FLnI_1p13.1a" | 0.253832993 |
| cg04548815 | chr1 | 1.16E+08 | 115643429 | gene_id "L1FLnI_1p13.1a" | 9.948338584 |
| cg12336269 | chr1 | 1.16E+08 | 115643429 | gene_id "L1FLnI_1p13.1a" | 2.058810545 |
| cg22063172 | chr1 | 1.16E+08 | 115643429 | gene_id "L1FLnI_1p13.1a" | 3.537288306 |
| cg25350269 | chr1 | 1.17E+08 | 116980025 | gene_id "L1FLnI_1p13.1f" | 3.427766176 |
| cg10989634 | chr1 | 1.11E+08 | 111272080 | gene_id "L1FLnI_1p13.2a" | 4.21050541 |
| cg13469595 | chr1 | 1.11E+08 | 111272080 | gene_id "L1FLnI_1p13.2a" | 1.898909779 |
| cg26834680 | chr1 | 1.11E+08 | 111272080 | gene_id "L1FLnI_1p13.2a" | 2.742761592 |
| cg09049701 | chr1 | 1.11E+08 | 111400395 | gene_id "L1FLnI_1p13.2d" | -1.653295354 |
| cg27525948 | chr1 | 1.11E+08 | 111400395 | gene_id "L1FLnI_1p13.2d" | -3.894787442 |
| cg00388838 | chr1 | 1.1E+08 | 109752441 | gene_id "L1FLnI_1p13.3j" | 0.455872249 |
| cg09344439 | chr1 | 1.07E+08 | 106559811 | gene_id "L1FLnI_1p21.1da" | 0.598776516 |
| cg26063904 | chr1 | 1.01E+08 | 100849599 | gene_id "L1FLnI_1p21.2l" | -2.069672726 |
| cg03072568 | chr1 | 1.02E+08 | 101657754 | gene_id "L1FLnI_1p21.2q" | 1.579064606 |
| cg18770231 | chr1 | 1.02E+08 | 101657754 | gene_id "L1FLnI_1p21.2q" | 0.308107309 |
| cg11235691 | chr1 | 74346509 | 74356601 | gene_id "L1FLnI_1p31.1aa" | 4.055459985 |
| cg15311773 | chr1 | 69904776 | 69914848 | gene_id "L1FLnI_1p31.1f" | -3.090060927 |
| cg06525611 | chr1 | 69922315 | 69932452 | gene_id "L1FLnI_1p31.1g" | -0.630831262 |
| cg05241796 | chr1 | 70739518 | 70749376 | gene_id "L1FLnI_1p31.1k" | -3.700291059 |
| cg07474678 | chr1 | 84157835 | 84167305 | gene_id "L1FLnI_1p31.1qc" | 0.86078668 |
| cg16528638 | chr1 | 84157835 | 84167305 | gene_id "L1FLnI_1p31.1qc" | 2.315740888 |
| cg22499652 | chr1 | 84157835 | 84167305 | gene_id "L1FLnI_1p31.1qc" | 1.063539349 |
| cg07517358 | chr1 | 80148899 | 80157931 | gene_id "L1FLnI_1p31.1vb" | -4.002310224 |
| cg05045403 | chr1 | 65461504 | 65471672 | gene_id "L1FLnI_1p31.3h" | 2.834364204 |
| cg13366057 | chr1 | 65461504 | 65471672 | gene_id "L1FLnI_1p31.3h" | 0.994315573 |
| cg00318766 | chr1 | 68495859 | 68505163 | gene_id "L1FLnI_1p31.3z" | 8.337830482 |
| cg08413469 | chr1 | 68495859 | 68505163 | gene_id "L1FLnI_1p31.3z" | 2.286258113 |
| cg08474403 | chr1 | 68495859 | 68505163 | gene_id "L1FLnI_1p31.3z" | 1.032464119 |
| cg08825216 | chr1 | 68495859 | 68505163 | gene_id "L1FLnI_1p31.3z" | -0.962697384 |
| cg16166651 | chr1 | 68495859 | 68505163 | gene_id "L1FLnI_1p31.3z" | 1.162691354 |
| cg18167921 | chr1 | 68495859 | 68505163 | gene_id "L1FLnI_1p31.3z" | -1.794392609 |
| cg19634693 | chr1 | 68495859 | 68505163 | gene_id "L1FLnI_1p31.3z" | 2.174588746 |
| cg26064634 | chr1 | 68495859 | 68505163 | gene_id "L1FLnI_1p31.3z" | 0.174690998 |
| cg08482190 | chr1 | 59787437 | 59796732 | gene_id "L1FLnI_1p32.1i" | 1.039633702 |
| ch.1.56581040R | chr1 | 56339717 | 56348769 | gene_id "L1FLnI_1p32.2a" | -8.719436736 |
| cg16182491 | chr1 | 40362115 | 40371369 | gene_id "L1FLnI_1p34.2a" | 2.118432611 |
| cg27068760 | chr1 | 40362115 | 40371369 | gene_id "L1FLnI_1p34.2a" | 0.290071902 |
| cg02355112 | chr1 | 24268893 | 24278714 | gene_id "L1FLnI_1p36.11" | 0.574641577 |
| cg22719314 | chr1 | 1.52E+08 | 152324437 | gene_id "L1FLnI_1q21.3d" | -0.646934042 |
| cg17924795 | chr1 | 1.53E+08 | 153192615 | gene_id "L1FLnI_1q21.3j" | -0.011657618 |
| cg12546793 | chr1 | 1.58E+08 | 158342736 | gene_id "L1FLnI_1q23.1n" | -2.237877449 |
| cg10194503 | chr1 | 1.59E+08 | 158969563 | gene_id "L1FLnI_1q23.1t" | 0.597141231 |
| cg00359421 | chr1 | 1.59E+08 | 159459476 | gene_id "L1FLnI_1q23.2c" | 7.153000364 |
| cg02477165 | chr1 | 1.61E+08 | 160812320 | gene_id "L1FLnI_1q23.3d" | 1.551781833 |
| cg01923690 | chr1 | 1.67E+08 | 166735808 | gene_id "L1FLnI_1q24.1i" | -0.567161348 |
| cg16641861 | chr1 | 1.69E+08 | 168688123 | gene_id "L1FLnI_1q24.2b" | 0.409418983 |
| cg19394080 | chr1 | 1.71E+08 | 170575870 | gene_id "L1FLnI_1q24.2s" | 1.133154458 |
| cg14220427 | chr1 | 1.73E+08 | 172990806 | gene_id "L1FLnI_1q24.3m" | 0.634282937 |
| cg00236991 | chr1 | 1.78E+08 | 177992695 | gene_id "L1FLnI_1q25.2n" | -0.51382169 |
| cg24706980 | chr1 | 1.84E+08 | 183664723 | gene_id "L1FLnI_1q25.3h" | -9.857053906 |
| cg04654210 | chr1 | 1.85E+08 | 185008055 | gene_id "L1FLnI_1q25.3m" | -0.290117865 |
| cg12812868 | chr1 | 1.88E+08 | 188075991 | gene_id "L1FLnI_1q31.1m" | 0.645841304 |
| cg19575580 | chr1 | 1.97E+08 | 196737575 | gene_id "L1FLnI_1q31.3h" | 0.02558797 |
| cg24804643 | chr1 | 1.97E+08 | 197494174 | gene_id "L1FLnI_1q31.3m" | 2.347280926 |
| cg12510723 | chr1 | 2.08E+08 | 208448428 | gene_id "L1FLnI_1q32.2h" | -1.342769039 |
| cg01679364 | chr1 | 2.09E+08 | 208621842 | gene_id "L1FLnI_1q32.2j" | -1.129229281 |
| cg08008573 | chr1 | 2.09E+08 | 208621842 | gene_id "L1FLnI_1q32.2j" | -1.655903671 |
| cg01966974 | chr1 | 2.13E+08 | 212664683 | gene_id "L1FLnI_1q32.3f" | 2.199922415 |
| cg23560250 | chr1 | 2.13E+08 | 212664683 | gene_id "L1FLnI_1q32.3f" | -1.3810748 |
| cg07696670 | chr1 | 2.15E+08 | 214731605 | gene_id "L1FLnI_1q41a" | 0.108720983 |
| cg01258940 | chr1 | 2.21E+08 | 221267115 | gene_id "L1FLnI_1q41ea" | -6.409812884 |
| cg08968773 | chr1 | 2.21E+08 | 221404363 | gene_id "L1FLnI_1q41ga" | -2.742675037 |
| cg00983697 | chr1 | 2.17E+08 | 217151250 | gene_id "L1FLnI_1q41j" | 2.523280011 |
| cg02057343 | chr1 | 2.36E+08 | 235852063 | gene_id "L1FLnI_1q42.3b" | -0.049387737 |
| cg07489842 | chr1 | 2.36E+08 | 235852063 | gene_id "L1FLnI_1q42.3b" | -0.615123238 |
| cg13974715 | chr1 | 2.36E+08 | 235852063 | gene_id "L1FLnI_1q42.3b" | -1.41332867 |
| cg24739596 | chr1 | 2.44E+08 | 243813690 | gene_id "L1FLnI_1q44b" | 3.944159225 |
| cg26145632 | chr1 | 2.44E+08 | 243825905 | gene_id "L1FLnI_1q44c" | 4.682619027 |
| cg05943574 | chr1 | 2.49E+08 | 248522766 | gene_id "L1FLnI_1q44x" | 3.528189125 |
| cg16892443 | chr20 | 24918225 | 24928409 | gene_id "L1FLnI_20p11.21p" | -3.725569986 |
| cg09852918 | chr20 | 24940590 | 24949408 | gene_id "L1FLnI_20p11.21q" | 0.042423075 |
| cg23726512 | chr20 | 21745193 | 21754591 | gene_id "L1FLnI_20p11.22c" | -0.22215979 |
| cg07265477 | chr20 | 16817949 | 16826625 | gene_id "L1FLnI_20p12.1s" | 2.841139218 |
| cg09285534 | chr20 | 2260949 | 2271106 | gene_id "L1FLnI_20p13h" | -2.334148063 |
| cg23710764 | chr20 | 30882378 | 30892527 | gene_id "L1FLnI_20q11.21b" | 1.402411335 |
| cg16173730 | chr20 | 42202771 | 42212817 | gene_id "L1FLnI_20q12g" | -1.243936812 |
| cg02928509 | chr20 | 44054688 | 44064618 | gene_id "L1FLnI_20q13.12d" | 1.760309475 |
| cg17035109 | chr21 | 10627591 | 10637720 | gene_id "L1FLnI_21p11.2n" | 0.592673008 |
| cg21769096 | chr21 | 17684808 | 17693460 | gene_id "L1FLnI_21q21.1v" | -0.591939421 |
| cg25906247 | chr21 | 17769255 | 17779704 | gene_id "L1FLnI_21q21.1w" | -4.676815678 |
| cg26475740 | chr21 | 25514803 | 25523754 | gene_id "L1FLnI_21q21.3a" | 1.281232488 |
| cg08334000 | chr22 | 28386349 | 28396369 | gene_id "L1FLnI_22q12.1g" | 3.113328425 |
| cg08149617 | chr22 | 47345287 | 47355153 | gene_id "L1FLnI_22q13.31b" | 0.105025728 |
| cg02775404 | chr22 | 49315838 | 49324351 | gene_id "L1FLnI_22q13.33" | 0.111578095 |
| cg05550339 | chr22 | 49315838 | 49324351 | gene_id "L1FLnI_22q13.33" | 0.108987466 |
| cg11650235 | chr2 | 84734393 | 84743791 | gene_id "L1FLnI_2p11.2o" | 2.245832183 |
| cg02533491 | chr2 | 71410976 | 71421001 | gene_id "L1FLnI_2p13.2a" | 0.623543562 |
| cg08694858 | chr2 | 71410976 | 71421001 | gene_id "L1FLnI_2p13.2a" | -1.584987046 |
| cg11852086 | chr2 | 72015451 | 72025460 | gene_id "L1FLnI_2p13.2b" | 0.178445461 |
| cg06295063 | chr2 | 68613150 | 68622413 | gene_id "L1FLnI_2p13.3b" | -0.097908972 |
| cg23153661 | chr2 | 70872415 | 70882584 | gene_id "L1FLnI_2p13.3e" | -0.576790387 |
| cg02738617 | chr2 | 57789102 | 57798496 | gene_id "L1FLnI_2p16.1v" | 0.593755407 |
| cg02348726 | chr2 | 49239914 | 49248826 | gene_id "L1FLnI_2p16.3i" | -0.72965913 |
| cg00861295 | chr2 | 46471560 | 46481604 | gene_id "L1FLnI_2p21h" | -1.98244944 |
| cg05897350 | chr2 | 37593699 | 37603827 | gene_id "L1FLnI_2p22.2b" | 8.919291057 |
| cg17878237 | chr2 | 31668611 | 31680312 | gene_id "L1FLnI_2p23.1h" | -4.207977234 |
| cg27508055 | chr2 | 19085768 | 19095888 | gene_id "L1FLnI_2p24.1b" | 3.255688285 |
| cg08479629 | chr2 | 19178903 | 19188921 | gene_id "L1FLnI_2p24.1c" | 0.877457409 |
| cg22141650 | chr2 | 12133602 | 12143111 | gene_id "L1FLnI_2p24.3a" | -3.809851311 |
| cg09973771 | chr2 | 1041867 | 1051251 | gene_id "L1FLnI_2p25.3e" | -0.700113484 |
| cg19432688 | chr2 | 1756465 | 1766542 | gene_id "L1FLnI_2p25.3g" | 0.564313797 |
| cg25372596 | chr2 | 3078666 | 3089024 | gene_id "L1FLnI_2p25.3i" | 0.956269279 |
| cg12081267 | chr2 | 97868972 | 97879356 | gene_id "L1FLnI_2q11.2h" | -0.154995258 |
| cg23256822 | chr2 | 1.02E+08 | 102435816 | gene_id "L1FLnI_2q12.1c" | 0.169770678 |
| cg23988182 | chr2 | 1.18E+08 | 118146520 | gene_id "L1FLnI_2q14.2a" | -2.768725521 |
| cg06333870 | chr2 | 1.19E+08 | 118905292 | gene_id "L1FLnI_2q14.2f" | -7.894459984 |
| cg18712968 | chr2 | 1.19E+08 | 119501555 | gene_id "L1FLnI_2q14.2j" | -2.37630518 |
| cg07668206 | chr2 | 1.22E+08 | 122022208 | gene_id "L1FLnI_2q14.3a" | -1.93392716 |
| cg10884612 | chr2 | 1.29E+08 | 128952931 | gene_id "L1FLnI_2q14.3sa" | 0.419737987 |
| cg22448304 | chr2 | 1.3E+08 | 129575447 | gene_id "L1FLnI_2q21.1c" | 0.288372027 |
| cg15928974 | chr2 | 1.33E+08 | 133392684 | gene_id "L1FLnI_2q21.2j" | -0.687677044 |
| cg16498879 | chr2 | 1.33E+08 | 133392684 | gene_id "L1FLnI_2q21.2j" | -0.201048371 |
| cg00294718 | chr2 | 1.49E+08 | 149261602 | gene_id "L1FLnI_2q23.2a" | 0.18648561 |
| cg12026329 | chr2 | 1.5E+08 | 149800253 | gene_id "L1FLnI_2q23.3b" | -4.605314131 |
| cg07779808 | chr2 | 1.54E+08 | 153623625 | gene_id "L1FLnI_2q23.3w" | 1.43694245 |
| cg24497582 | chr2 | 1.68E+08 | 167841689 | gene_id "L1FLnI_2q24.3r" | -1.500685724 |
| cg06843147 | chr2 | 1.69E+08 | 169126662 | gene_id "L1FLnI_2q31.1a" | -0.027170228 |
| cg20801278 | chr2 | 1.69E+08 | 169405799 | gene_id "L1FLnI_2q31.1d" | -9.2761949 |
| cg23726945 | chr2 | 1.74E+08 | 173536817 | gene_id "L1FLnI_2q31.1q" | -0.311468744 |
| cg16023497 | chr2 | 1.76E+08 | 175715773 | gene_id "L1FLnI_2q31.1w" | -0.397097424 |
| cg24252907 | chr2 | 1.78E+08 | 177792049 | gene_id "L1FLnI_2q31.2c" | -0.499687089 |
| cg15699121 | chr2 | 1.87E+08 | 186905775 | gene_id "L1FLnI_2q32.1la" | -0.709237412 |
| cg05256276 | chr2 | 1.88E+08 | 188400736 | gene_id "L1FLnI_2q32.1qa" | -1.593991268 |
| cg07974890 | chr2 | 1.92E+08 | 191768376 | gene_id "L1FLnI_2q32.3a" | -0.240585309 |
| cg06915453 | chr2 | 1.96E+08 | 195750492 | gene_id "L1FLnI_2q32.3ca" | -0.020307223 |
| cg03020006 | chr2 | 2.02E+08 | 201619221 | gene_id "L1FLnI_2q33.1aa" | -0.080225474 |
| cg04411541 | chr2 | 2.02E+08 | 201619221 | gene_id "L1FLnI_2q33.1aa" | 3.09735173 |
| cg26355357 | chr2 | 2.02E+08 | 201619221 | gene_id "L1FLnI_2q33.1aa" | 5.690003469 |
| cg09337141 | chr2 | 1.97E+08 | 196761836 | gene_id "L1FLnI_2q33.1b" | 3.317073758 |
| cg07227024 | chr2 | 2.01E+08 | 201306598 | gene_id "L1FLnI_2q33.1u" | 0.048203738 |
| cg21745710 | chr2 | 2.01E+08 | 201376274 | gene_id "L1FLnI_2q33.1x" | 2.076683827 |
| cg13861099 | chr2 | 2.11E+08 | 210512044 | gene_id "L1FLnI_2q34ca" | 4.682286729 |
| cg12662260 | chr2 | 2.14E+08 | 214313288 | gene_id "L1FLnI_2q34sa" | 0.456240146 |
| cg25471980 | chr2 | 2.14E+08 | 214313288 | gene_id "L1FLnI_2q34sa" | -0.983549581 |
| cg01331064 | chr2 | 2.15E+08 | 215226831 | gene_id "L1FLnI_2q35c" | -0.006212682 |
| cg04262505 | chr2 | 2.17E+08 | 216986654 | gene_id "L1FLnI_2q35j" | -2.669006427 |
| cg20568285 | chr2 | 2.32E+08 | 232185109 | gene_id "L1FLnI_2q37.1h" | 2.145519299 |
| cg19183395 | chr2 | 2.32E+08 | 232361745 | gene_id "L1FLnI_2q37.1i" | -0.006325488 |
| cg10596609 | chr2 | 2.34E+08 | 233620114 | gene_id "L1FLnI_2q37.1k" | -1.433704788 |
| cg18526870 | chr2 | 2.34E+08 | 233638898 | gene_id "L1FLnI_2q37.1l" | 1.85889584 |
| cg09690215 | chr2 | 2.4E+08 | 240284789 | gene_id "L1FLnI_2q37.3c" | 0.347977728 |
| cg21990299 | chr3 | 58275145 | 58285229 | gene_id "L1FLnI_3p14.3q" | 0.140532906 |
| cg00220419 | chr3 | 53364798 | 53374825 | gene_id "L1FLnI_3p21.1a" | 1.151849956 |
| cg05286501 | chr3 | 53921249 | 53931305 | gene_id "L1FLnI_3p21.1f" | 1.683216037 |
| cg18489244 | chr3 | 45272763 | 45282209 | gene_id "L1FLnI_3p21.31a" | -6.65396607 |
| cg25956276 | chr3 | 43061276 | 43071298 | gene_id "L1FLnI_3p22.1ca" | -1.736045522 |
| cg14741811 | chr3 | 40901906 | 40911932 | gene_id "L1FLnI_3p22.1m" | -0.271205741 |
| cg15064667 | chr3 | 40901906 | 40911932 | gene_id "L1FLnI_3p22.1m" | -0.11419449 |
| cg24228058 | chr3 | 37612464 | 37622602 | gene_id "L1FLnI_3p22.2c" | -2.29427815 |
| cg11281103 | chr3 | 36185924 | 36195342 | gene_id "L1FLnI_3p22.3da" | -0.087240015 |
| cg25954358 | chr3 | 21011364 | 21021592 | gene_id "L1FLnI_3p24.3ca" | 0.456652357 |
| cg00090282 | chr3 | 21719590 | 21728789 | gene_id "L1FLnI_3p24.3ka" | 0.329853697 |
| cg25039219 | chr3 | 8712578 | 8722597 | gene_id "L1FLnI_3p25.3e" | -3.23063014 |
| cg14055195 | chr3 | 99288097 | 99297802 | gene_id "L1FLnI_3q12.1h" | -0.578134535 |
| cg13709487 | chr3 | 1.01E+08 | 100604997 | gene_id "L1FLnI_3q12.2c" | -6.107894107 |
| cg08161364 | chr3 | 1.02E+08 | 102014855 | gene_id "L1FLnI_3q12.3f" | 0.292285055 |
| cg08230969 | chr3 | 1.02E+08 | 102046553 | gene_id "L1FLnI_3q12.3g" | 0.181501442 |
| cg14840439 | chr3 | 1.1E+08 | 109517900 | gene_id "L1FLnI_3q13.13f" | -0.0731558 |
| cg20386784 | chr3 | 1.1E+08 | 109517900 | gene_id "L1FLnI_3q13.13f" | -0.688540622 |
| cg15421872 | chr3 | 1.12E+08 | 111762249 | gene_id "L1FLnI_3q13.2b" | -5.042508787 |
| cg27222463 | chr3 | 1.12E+08 | 112258847 | gene_id "L1FLnI_3q13.2g" | 0.469582049 |
| cg23826961 | chr3 | 1.14E+08 | 114283406 | gene_id "L1FLnI_3q13.31a" | 4.536652077 |
| cg08580126 | chr3 | 1.15E+08 | 115321970 | gene_id "L1FLnI_3q13.31c" | 0.937227097 |
| cg11235882 | chr3 | 1.15E+08 | 115321970 | gene_id "L1FLnI_3q13.31c" | 0.621688409 |
| cg08875971 | chr3 | 1.18E+08 | 118418928 | gene_id "L1FLnI_3q13.32c" | 2.579033645 |
| cg19569053 | chr3 | 1.2E+08 | 119572817 | gene_id "L1FLnI_3q13.33a" | -1.945736973 |
| cg06798350 | chr3 | 1.26E+08 | 125943169 | gene_id "L1FLnI_3q21.2b" | -4.824087716 |
| cg18082362 | chr3 | 1.32E+08 | 132142253 | gene_id "L1FLnI_3q22.1p" | -0.546855365 |
| cg21531775 | chr3 | 1.32E+08 | 132380761 | gene_id "L1FLnI_3q22.1q" | 0.042521908 |
| cg07149179 | chr3 | 1.35E+08 | 135261730 | gene_id "L1FLnI_3q22.2g" | 0.102678435 |
| cg01165403 | chr3 | 1.36E+08 | 136120799 | gene_id "L1FLnI_3q22.3a" | -1.00138515 |
| cg03290530 | chr3 | 1.38E+08 | 137643232 | gene_id "L1FLnI_3q22.3q" | 0.126502212 |
| cg09535707 | chr3 | 1.38E+08 | 137643232 | gene_id "L1FLnI_3q22.3q" | 0.363767178 |
| cg02785222 | chr3 | 1.39E+08 | 139176099 | gene_id "L1FLnI_3q23b" | -2.469866701 |
| cg02456782 | chr3 | 1.55E+08 | 155244196 | gene_id "L1FLnI_3q25.2v" | -2.855070293 |
| cg14033737 | chr3 | 1.62E+08 | 162415393 | gene_id "L1FLnI_3q26.1e" | 0.011240453 |
| cg24874998 | chr3 | 1.63E+08 | 163311204 | gene_id "L1FLnI_3q26.1i" | 1.597289073 |
| cg05410708 | chr3 | 1.71E+08 | 170979236 | gene_id "L1FLnI_3q26.2m" | 1.063849723 |
| cg19408207 | chr3 | 1.79E+08 | 179044862 | gene_id "L1FLnI_3q26.32v" | 0.76484854 |
| cg02199947 | chr3 | 1.8E+08 | 180170319 | gene_id "L1FLnI_3q26.33a" | -6.369993368 |
| cg02041470 | chr3 | 1.82E+08 | 182160670 | gene_id "L1FLnI_3q26.33l" | -2.871868641 |
| cg02519925 | chr3 | 1.95E+08 | 195094193 | gene_id "L1FLnI_3q29g" | 3.254822906 |
| cg04371358 | chr3 | 1.95E+08 | 195094193 | gene_id "L1FLnI_3q29g" | -0.915022208 |
| cg03640807 | chr4 | 47916549 | 47926611 | gene_id "L1FLnI_4p12q" | 3.26208009 |
| cg24665592 | chr4 | 41345962 | 41356118 | gene_id "L1FLnI_4p13b" | -0.026435686 |
| cg10533705 | chr4 | 42695802 | 42705936 | gene_id "L1FLnI_4p13f" | -0.36572045 |
| cg19143671 | chr4 | 27995955 | 28005909 | gene_id "L1FLnI_4p15.1a" | 0.780043944 |
| cg21583438 | chr4 | 28163550 | 28172624 | gene_id "L1FLnI_4p15.1b" | 0.074733248 |
| cg24852605 | chr4 | 27375189 | 27385219 | gene_id "L1FLnI_4p15.2ca" | 0.038374237 |
| cg14102569 | chr4 | 12086483 | 12096625 | gene_id "L1FLnI_4p15.33g" | -0.011594154 |
| cg07298638 | chr4 | 13413266 | 13423417 | gene_id "L1FLnI_4p15.33p" | 1.657668125 |
| cg20631120 | chr4 | 13413266 | 13423417 | gene_id "L1FLnI_4p15.33p" | 1.316142627 |
| cg27283110 | chr4 | 10774895 | 10784920 | gene_id "L1FLnI_4p16.1e" | 0.634549476 |
| cg12977705 | chr4 | 51824403 | 51833165 | gene_id "L1FLnI_4q12a" | -0.312104786 |
| cg05441001 | chr4 | 55615655 | 55625681 | gene_id "L1FLnI_4q12ab" | 0.308468388 |
| cg05807665 | chr4 | 55163777 | 55173737 | gene_id "L1FLnI_4q12xa" | -7.099557825 |
| cg09092906 | chr4 | 55293663 | 55303786 | gene_id "L1FLnI_4q12za" | 0.550323724 |
| cg10335018 | chr4 | 75073744 | 75083889 | gene_id "L1FLnI_4q13.3ab" | -0.103782724 |
| cg05419625 | chr4 | 74208714 | 74219159 | gene_id "L1FLnI_4q13.3sa" | 1.271907418 |
| cg15555527 | chr4 | 79963409 | 79973433 | gene_id "L1FLnI_4q21.21k" | 4.035925096 |
| cg06467317 | chr4 | 80355125 | 80363711 | gene_id "L1FLnI_4q21.21n" | -1.596777971 |
| cg15749469 | chr4 | 80355125 | 80363711 | gene_id "L1FLnI_4q21.21n" | -0.314124018 |
| cg07757454 | chr4 | 81151699 | 81160621 | gene_id "L1FLnI_4q21.21u" | -0.709697955 |
| cg11561282 | chr4 | 83238126 | 83247452 | gene_id "L1FLnI_4q21.23a" | -1.944199036 |
| cg02898821 | chr4 | 86449238 | 86458379 | gene_id "L1FLnI_4q21.3g" | 0.572609198 |
| cg02518222 | chr4 | 87346625 | 87356646 | gene_id "L1FLnI_4q22.1a" | -0.553635933 |
| cg12132765 | chr4 | 87346625 | 87356646 | gene_id "L1FLnI_4q22.1a" | -0.421700892 |
| cg06064549 | chr4 | 87813150 | 87823790 | gene_id "L1FLnI_4q22.1e" | -0.053879573 |
| cg18884413 | chr4 | 89057381 | 89067210 | gene_id "L1FLnI_4q22.1m" | -1.205376277 |
| cg04539834 | chr4 | 99290001 | 99299901 | gene_id "L1FLnI_4q23k" | 0.686916304 |
| cg05890281 | chr4 | 1E+08 | 100010053 | gene_id "L1FLnI_4q23u" | 0.057446282 |
| cg10773082 | chr4 | 1E+08 | 100010053 | gene_id "L1FLnI_4q23u" | -0.003426534 |
| cg13442045 | chr4 | 1.01E+08 | 101415195 | gene_id "L1FLnI_4q24g" | 0.323985731 |
| cg27425532 | chr4 | 1.11E+08 | 111170681 | gene_id "L1FLnI_4q25ba" | -1.047866267 |
| ch.4.109582151R | chr4 | 1.08E+08 | 108445089 | gene_id "L1FLnI_4q25g" | -7.686083429 |
| cg03708153 | chr4 | 1.1E+08 | 109591097 | gene_id "L1FLnI_4q25l" | 3.167075845 |
| cg19915620 | chr4 | 1.1E+08 | 109591097 | gene_id "L1FLnI_4q25l" | 0.030048443 |
| cg17274599 | chr4 | 1.18E+08 | 118410337 | gene_id "L1FLnI_4q26na" | 1.759953169 |
| cg03490300 | chr4 | 1.16E+08 | 115856359 | gene_id "L1FLnI_4q26t" | -1.849462248 |
| cg21447540 | chr4 | 1.19E+08 | 119488188 | gene_id "L1FLnI_4q26wa" | -0.918495189 |
| cg09542154 | chr4 | 1.28E+08 | 127866187 | gene_id "L1FLnI_4q28.1ma" | -1.404720784 |
| cg14235511 | chr4 | 1.39E+08 | 138789136 | gene_id "L1FLnI_4q31.1b" | 1.78352725 |
| cg01280906 | chr4 | 1.41E+08 | 140921688 | gene_id "L1FLnI_4q31.21c" | 0.021733623 |
| cg24270157 | chr4 | 1.42E+08 | 141712368 | gene_id "L1FLnI_4q31.21j" | -0.357537717 |
| cg03759773 | chr4 | 1.42E+08 | 142478407 | gene_id "L1FLnI_4q31.21s" | 1.592515551 |
| cg08368933 | chr4 | 1.42E+08 | 142478407 | gene_id "L1FLnI_4q31.21s" | 0.096142363 |
| cg15485154 | chr4 | 1.42E+08 | 142478407 | gene_id "L1FLnI_4q31.21s" | -11.04521673 |
| cg01095227 | chr4 | 1.49E+08 | 149072119 | gene_id "L1FLnI_4q31.23e" | 0.503332946 |
| cg18830282 | chr4 | 1.5E+08 | 150508314 | gene_id "L1FLnI_4q31.3b" | 0.025575455 |
| cg15123353 | chr4 | 1.65E+08 | 164629907 | gene_id "L1FLnI_4q32.3l" | 1.170115385 |
| cg22186586 | chr4 | 1.67E+08 | 167108404 | gene_id "L1FLnI_4q32.3y" | -0.117058944 |
| cg08195271 | chr4 | 1.74E+08 | 173612686 | gene_id "L1FLnI_4q34.1o" | 1.352986722 |
| cg01884240 | chr4 | 1.75E+08 | 175125933 | gene_id "L1FLnI_4q34.1x" | 0.298482379 |
| cg09745331 | chr4 | 1.84E+08 | 184214753 | gene_id "L1FLnI_4q35.1e" | 1.180214524 |
| cg26088632 | chr5 | 43399050 | 43409147 | gene_id "L1FLnI_5p12i" | 4.015823045 |
| cg24561507 | chr5 | 44059391 | 44068851 | gene_id "L1FLnI_5p12u" | 0.672934824 |
| cg18788642 | chr5 | 38625101 | 38635247 | gene_id "L1FLnI_5p13.1a" | 2.605853457 |
| cg05015628 | chr5 | 39030831 | 39040976 | gene_id "L1FLnI_5p13.1b" | -2.651426314 |
| cg10057884 | chr5 | 39275246 | 39285386 | gene_id "L1FLnI_5p13.1c" | -8.748768662 |
| cg01539206 | chr5 | 33855215 | 33864635 | gene_id "L1FLnI_5p13.2a" | 0.417185255 |
| cg14775730 | chr5 | 35741290 | 35751967 | gene_id "L1FLnI_5p13.2t" | 1.900085158 |
| cg00589791 | chr5 | 30422879 | 30433007 | gene_id "L1FLnI_5p13.3m" | 0.129732943 |
| cg05086553 | chr5 | 18648519 | 18657330 | gene_id "L1FLnI_5p14.3c" | 0.088657645 |
| cg17982058 | chr5 | 9768283 | 9780062 | gene_id "L1FLnI_5p15.31s" | 6.862102705 |
| cg06803706 | chr5 | 1706320 | 1716335 | gene_id "L1FLnI_5p15.33c" | -0.138892535 |
| cg13229520 | chr5 | 51345169 | 51355322 | gene_id "L1FLnI_5q11.1q" | -0.598810142 |
| cg14542504 | chr5 | 59007277 | 59016791 | gene_id "L1FLnI_5q11.2ab" | -1.167293013 |
| cg14413394 | chr5 | 58284563 | 58294668 | gene_id "L1FLnI_5q11.2qa" | 1.744063018 |
| cg13344386 | chr5 | 63399469 | 63408465 | gene_id "L1FLnI_5q12.1fa" | -3.567975654 |
| cg04679515 | chr5 | 62813794 | 62823872 | gene_id "L1FLnI_5q12.1w" | -2.787399532 |
| cg27527970 | chr5 | 67597838 | 67607971 | gene_id "L1FLnI_5q13.1a" | 0.406443892 |
| cg02093951 | chr5 | 67814446 | 67824459 | gene_id "L1FLnI_5q13.1i" | 0.703974245 |
| cg24727109 | chr5 | 73203267 | 73213715 | gene_id "L1FLnI_5q13.2h" | -0.250951034 |
| cg23129178 | chr5 | 75638737 | 75648765 | gene_id "L1FLnI_5q13.3g" | -0.151589479 |
| cg04269291 | chr5 | 75963710 | 75973835 | gene_id "L1FLnI_5q13.3p" | 0.304308957 |
| cg06671473 | chr5 | 77497406 | 77507549 | gene_id "L1FLnI_5q13.3u" | 0.231019338 |
| cg06001854 | chr5 | 78381931 | 78391524 | gene_id "L1FLnI_5q14.1e" | -0.456333466 |
| cg01827571 | chr5 | 78578843 | 78588495 | gene_id "L1FLnI_5q14.1i" | 1.638717552 |
| cg09796935 | chr5 | 82928945 | 82938960 | gene_id "L1FLnI_5q14.2h" | -2.087029672 |
| cg24192058 | chr5 | 90188576 | 90197247 | gene_id "L1FLnI_5q14.3va" | -4.397036274 |
| cg19674688 | chr5 | 93257537 | 93267565 | gene_id "L1FLnI_5q15c" | -2.36550836 |
| cg23965352 | chr5 | 93257537 | 93267565 | gene_id "L1FLnI_5q15c" | -3.890500628 |
| cg20952076 | chr5 | 97152790 | 97163699 | gene_id "L1FLnI_5q15z" | -3.385300501 |
| cg00120286 | chr5 | 1.09E+08 | 109451143 | gene_id "L1FLnI_5q21.3x" | -0.057154318 |
| cg10514770 | chr5 | 1.09E+08 | 109451143 | gene_id "L1FLnI_5q21.3x" | -1.939027964 |
| cg09023351 | chr5 | 1.09E+08 | 109463890 | gene_id "L1FLnI_5q21.3y" | -0.555702137 |
| cg07309238 | chr5 | 1.11E+08 | 110896387 | gene_id "L1FLnI_5q22.1f" | -0.041665202 |
| cg11809897 | chr5 | 1.11E+08 | 111094408 | gene_id "L1FLnI_5q22.1j" | -0.277069489 |
| cg13635023 | chr5 | 1.13E+08 | 113081325 | gene_id "L1FLnI_5q22.2i" | 8.774100646 |
| cg14670213 | chr5 | 1.21E+08 | 121070741 | gene_id "L1FLnI_5q23.1ba" | -0.438610795 |
| cg22106273 | chr5 | 1.18E+08 | 118295460 | gene_id "L1FLnI_5q23.1g" | -0.1586113 |
| cg11846226 | chr5 | 1.19E+08 | 119303763 | gene_id "L1FLnI_5q23.1k" | 0.677691908 |
| cg01777372 | chr5 | 1.21E+08 | 120609556 | gene_id "L1FLnI_5q23.1z" | 0.888093429 |
| cg20252099 | chr5 | 1.26E+08 | 125992620 | gene_id "L1FLnI_5q23.2fa" | -0.640787474 |
| cg03557835 | chr5 | 1.29E+08 | 128659929 | gene_id "L1FLnI_5q23.3e" | 0.85847182 |
| cg21253560 | chr5 | 1.29E+08 | 128853207 | gene_id "L1FLnI_5q23.3f" | -3.30328125 |
| cg07421806 | chr5 | 1.29E+08 | 128965993 | gene_id "L1FLnI_5q23.3h" | -9.567565073 |
| cg06516533 | chr5 | 1.32E+08 | 132403647 | gene_id "L1FLnI_5q31.1l" | -4.140125698 |
| cg07314206 | chr5 | 1.39E+08 | 138688327 | gene_id "L1FLnI_5q31.2f" | 1.765063628 |
| cg09929786 | chr5 | 1.44E+08 | 143858714 | gene_id "L1FLnI_5q31.3d" | -1.435271779 |
| cg16762925 | chr5 | 1.48E+08 | 147615140 | gene_id "L1FLnI_5q32n" | -5.521609342 |
| cg10504055 | chr5 | 1.49E+08 | 148677844 | gene_id "L1FLnI_5q32x" | -2.581864292 |
| cg18727618 | chr5 | 1.49E+08 | 148677844 | gene_id "L1FLnI_5q32x" | 0.371818469 |
| cg16192428 | chr5 | 1.49E+08 | 148693047 | gene_id "L1FLnI_5q32y" | 0.00796045 |
| cg02752133 | chr5 | 1.52E+08 | 152349552 | gene_id "L1FLnI_5q33.1l" | -3.391885834 |
| cg18433473 | chr5 | 1.53E+08 | 153154465 | gene_id "L1FLnI_5q33.1v" | 5.189042269 |
| cg25148632 | chr5 | 1.67E+08 | 166976315 | gene_id "L1FLnI_5q34na" | 6.316114632 |
| cg16170223 | chr5 | 1.63E+08 | 162632448 | gene_id "L1FLnI_5q34q" | 0.231910998 |
| cg05207048 | chr5 | 1.68E+08 | 168087078 | gene_id "L1FLnI_5q34qa" | -12.1217742 |
| cg20418172 | chr5 | 1.7E+08 | 170249295 | gene_id "L1FLnI_5q35.1c" | 0.001060891 |
| cg04561094 | chr5 | 1.78E+08 | 177795816 | gene_id "L1FLnI_5q35.3b" | 0.111636476 |
| cg05051799 | chr6 | 53941694 | 53952420 | gene_id "L1FLnI_6p12.1e" | 1.227579429 |
| cg12341018 | chr6 | 56301213 | 56311370 | gene_id "L1FLnI_6p12.1u" | 0.266643852 |
| cg06981276 | chr6 | 51848420 | 51857614 | gene_id "L1FLnI_6p12.2b" | -3.118580261 |
| cg26131114 | chr6 | 51117937 | 51128084 | gene_id "L1FLnI_6p12.3ka" | 0.731800066 |
| cg04124466 | chr6 | 51666059 | 51675471 | gene_id "L1FLnI_6p12.3qa" | 1.793870074 |
| cg16470491 | chr6 | 50026782 | 50036834 | gene_id "L1FLnI_6p12.3z" | 0.798904627 |
| cg25385020 | chr6 | 44516002 | 44526905 | gene_id "L1FLnI_6p21.1b" | -0.654220004 |
| cg08637101 | chr6 | 44583674 | 44593813 | gene_id "L1FLnI_6p21.1c" | 0.908954752 |
| cg02705792 | chr6 | 44896545 | 44906675 | gene_id "L1FLnI_6p21.1g" | 1.907010244 |
| cg22127299 | chr6 | 28017259 | 28027412 | gene_id "L1FLnI_6p22.1a" | -1.20308445 |
| cg12590769 | chr6 | 18353284 | 18362833 | gene_id "L1FLnI_6p22.3f" | 0.021274471 |
| cg17404640 | chr6 | 22459241 | 22468054 | gene_id "L1FLnI_6p22.3t" | 0.323558894 |
| cg12916580 | chr6 | 4401855 | 4411605 | gene_id "L1FLnI_6p25.1c" | -1.164800123 |
| cg14163722 | chr6 | 4401855 | 4411605 | gene_id "L1FLnI_6p25.1c" | -8.815544674 |
| cg02875740 | chr6 | 6664656 | 6673490 | gene_id "L1FLnI_6p25.1p" | 1.733355109 |
| cg20958467 | chr6 | 2873372 | 2883353 | gene_id "L1FLnI_6p25.2c" | -4.597125253 |
| cg23172545 | chr6 | 2873372 | 2883353 | gene_id "L1FLnI_6p25.2c" | -0.415681135 |
| cg22833428 | chr6 | 1428116 | 1438250 | gene_id "L1FLnI_6p25.3b" | -0.243305782 |
| cg13960067 | chr6 | 2074777 | 2084288 | gene_id "L1FLnI_6p25.3d" | 1.378225517 |
| cg21800170 | chr6 | 65768582 | 65778702 | gene_id "L1FLnI_6q12s" | 2.349702552 |
| cg05273639 | chr6 | 71267016 | 71277165 | gene_id "L1FLnI_6q13x" | 1.103177246 |
| cg02549516 | chr6 | 78802152 | 78811596 | gene_id "L1FLnI_6q14.1ha" | 0.272847145 |
| cg18292383 | chr6 | 78802152 | 78811596 | gene_id "L1FLnI_6q14.1ha" | 1.494219163 |
| cg12248480 | chr6 | 80567440 | 80577445 | gene_id "L1FLnI_6q14.1ya" | -0.195154324 |
| cg00033066 | chr6 | 83737353 | 83747953 | gene_id "L1FLnI_6q14.2g" | 0.564036553 |
| cg16541147 | chr6 | 83797244 | 83807270 | gene_id "L1FLnI_6q14.2h" | -0.066669365 |
| cg15620008 | chr6 | 84068470 | 84077112 | gene_id "L1FLnI_6q14.2k" | -0.336326723 |
| cg17847526 | chr6 | 84977966 | 84986517 | gene_id "L1FLnI_6q14.3j" | 0.355401169 |
| cg01156568 | chr6 | 87136187 | 87146258 | gene_id "L1FLnI_6q14.3wa" | -16.47512708 |
| cg11795893 | chr6 | 87136187 | 87146258 | gene_id "L1FLnI_6q14.3wa" | -6.651306492 |
| cg25865489 | chr6 | 99820099 | 99830094 | gene_id "L1FLnI_6q16.2g" | -0.518876686 |
| cg16685777 | chr6 | 1E+08 | 100361225 | gene_id "L1FLnI_6q16.3b" | 0.206146727 |
| cg05484606 | chr6 | 1.13E+08 | 113233015 | gene_id "L1FLnI_6q21da" | -0.721027485 |
| cg03482387 | chr6 | 1.06E+08 | 106411523 | gene_id "L1FLnI_6q21e" | 0.259685768 |
| cg24300962 | chr6 | 1.14E+08 | 113537647 | gene_id "L1FLnI_6q21fa" | -0.460609219 |
| cg08383171 | chr6 | 1.17E+08 | 117355325 | gene_id "L1FLnI_6q22.1ia" | 3.430441207 |
| cg01820962 | chr6 | 1.16E+08 | 116190817 | gene_id "L1FLnI_6q22.1x" | -11.27042992 |
| cg03348540 | chr6 | 1.25E+08 | 125402744 | gene_id "L1FLnI_6q22.31ua" | 2.542143257 |
| cg19906877 | chr6 | 1.25E+08 | 125402744 | gene_id "L1FLnI_6q22.31ua" | 0.497423241 |
| cg03838823 | chr6 | 1.28E+08 | 127599792 | gene_id "L1FLnI_6q22.33e" | -0.205072479 |
| cg14126093 | chr6 | 1.29E+08 | 128593840 | gene_id "L1FLnI_6q22.33j" | 2.399468215 |
| cg00078458 | chr6 | 1.31E+08 | 130708267 | gene_id "L1FLnI_6q23.1c" | -3.291358955 |
| cg05051876 | chr6 | 1.32E+08 | 132358908 | gene_id "L1FLnI_6q23.2h" | 2.793836641 |
| cg02004823 | chr6 | 1.44E+08 | 143674419 | gene_id "L1FLnI_6q24.2f" | 0.095387137 |
| cg22008851 | chr6 | 1.61E+08 | 160689251 | gene_id "L1FLnI_6q26a" | 9.478376184 |
| cg24029114 | chr6 | 1.61E+08 | 160689251 | gene_id "L1FLnI_6q26a" | 0.892990633 |
| cg12705408 | chr6 | 1.61E+08 | 160750393 | gene_id "L1FLnI_6q26b" | 0.975561271 |
| cg25115148 | chr6 | 1.61E+08 | 160750393 | gene_id "L1FLnI_6q26b" | 2.180728229 |
| cg18836146 | chr6 | 1.61E+08 | 160944338 | gene_id "L1FLnI_6q26c" | 0.14809915 |
| cg23219388 | chr6 | 1.68E+08 | 168150149 | gene_id "L1FLnI_6q27m" | -0.086239669 |
| cg26526883 | chr6 | 1.68E+08 | 168150149 | gene_id "L1FLnI_6q27m" | 1.211017575 |
| cg22624022 | chr7 | 54923017 | 54933525 | gene_id "L1FLnI_7p11.2h" | 9.094895605 |
| cg11266396 | chr7 | 55576690 | 55586813 | gene_id "L1FLnI_7p11.2j" | -0.004848807 |
| cg20170989 | chr7 | 53025823 | 53035836 | gene_id "L1FLnI_7p12.1r" | -3.823645218 |
| cg05511794 | chr7 | 53740167 | 53750862 | gene_id "L1FLnI_7p12.1v" | 0.065716553 |
| cg27069616 | chr7 | 49034804 | 49044922 | gene_id "L1FLnI_7p12.2a" | 0.796609383 |
| cg12361987 | chr7 | 50067577 | 50079044 | gene_id "L1FLnI_7p12.2e" | -3.643372832 |
| cg19685916 | chr7 | 46011733 | 46021599 | gene_id "L1FLnI_7p12.3d" | 1.016789321 |
| cg01976726 | chr7 | 46231371 | 46240609 | gene_id "L1FLnI_7p12.3e" | 0.280173908 |
| cg23151856 | chr7 | 46587237 | 46597446 | gene_id "L1FLnI_7p12.3g" | -2.745540432 |
| cg25191305 | chr7 | 48529422 | 48539483 | gene_id "L1FLnI_7p12.3k" | -0.247354885 |
| cg05481217 | chr7 | 37162844 | 37172991 | gene_id "L1FLnI_7p14.1d" | -0.601342246 |
| cg22829007 | chr7 | 37162844 | 37172991 | gene_id "L1FLnI_7p14.1d" | -9.136826632 |
| cg11233105 | chr7 | 39007029 | 39017052 | gene_id "L1FLnI_7p14.1j" | 3.539045162 |
| cg15990679 | chr7 | 34902741 | 34912768 | gene_id "L1FLnI_7p14.2b" | -2.993276994 |
| cg00460793 | chr7 | 36116457 | 36126650 | gene_id "L1FLnI_7p14.2g" | -0.217398776 |
| cg15352065 | chr7 | 34199733 | 34209777 | gene_id "L1FLnI_7p14.3x" | 0.26686785 |
| cg05535908 | chr7 | 34229063 | 34239398 | gene_id "L1FLnI_7p14.3y" | 0.638736147 |
| cg01187600 | chr7 | 16909104 | 16917647 | gene_id "L1FLnI_7p21.1b" | 5.835634456 |
| cg27120543 | chr7 | 15513874 | 15524345 | gene_id "L1FLnI_7p21.2i" | -0.03013974 |
| cg02162205 | chr7 | 12427248 | 12435989 | gene_id "L1FLnI_7p21.3la" | 1.560435558 |
| cg12514654 | chr7 | 12493713 | 12503738 | gene_id "L1FLnI_7p21.3ma" | -0.002892927 |
| cg16705561 | chr7 | 70193830 | 70203857 | gene_id "L1FLnI_7q11.22a" | -0.936067925 |
| cg17905009 | chr7 | 84058911 | 84068936 | gene_id "L1FLnI_7q21.11da" | 1.592433389 |
| cg20747774 | chr7 | 80560888 | 80569866 | gene_id "L1FLnI_7q21.11p" | 1.469396064 |
| cg00670637 | chr7 | 91503238 | 91513367 | gene_id "L1FLnI_7q21.2a" | -0.097227344 |
| cg16917609 | chr7 | 91755699 | 91765685 | gene_id "L1FLnI_7q21.2g" | 1.058661999 |
| cg12695360 | chr7 | 92918736 | 92928860 | gene_id "L1FLnI_7q21.2m" | 0.16869174 |
| cg01101472 | chr7 | 93118050 | 93128002 | gene_id "L1FLnI_7q21.2p" | -0.424165071 |
| cg17668728 | chr7 | 93118050 | 93128002 | gene_id "L1FLnI_7q21.2p" | -0.540135933 |
| cg14772398 | chr7 | 1.04E+08 | 104216389 | gene_id "L1FLnI_7q22.2a" | -0.526951016 |
| cg22840454 | chr7 | 1.04E+08 | 104216389 | gene_id "L1FLnI_7q22.2a" | 0.063052868 |
| cg05655307 | chr7 | 1.11E+08 | 111506480 | gene_id "L1FLnI_7q31.1ba" | 5.883689318 |
| cg00192468 | chr7 | 1.24E+08 | 123598623 | gene_id "L1FLnI_7q31.32j" | 0.52432394 |
| cg24073215 | chr7 | 1.24E+08 | 123848654 | gene_id "L1FLnI_7q31.32k" | 3.175813603 |
| cg23785462 | chr7 | 1.24E+08 | 124103710 | gene_id "L1FLnI_7q31.32p" | -0.236802514 |
| cg06024364 | chr7 | 1.36E+08 | 136118222 | gene_id "L1FLnI_7q33k" | -0.739363958 |
| cg24652379 | chr7 | 1.42E+08 | 141779586 | gene_id "L1FLnI_7q34f" | 0.718873346 |
| cg20052431 | chr7 | 1.46E+08 | 146045005 | gene_id "L1FLnI_7q35m" | 0.019205832 |
| cg01982516 | chr7 | 1.57E+08 | 156919042 | gene_id "L1FLnI_7q36.3d" | 3.003473977 |
| cg21481322 | chr8 | 41125927 | 41135181 | gene_id "L1FLnI_8p11.21h" | 0.043079597 |
| cg18934074 | chr8 | 41221510 | 41231611 | gene_id "L1FLnI_8p11.21i" | -0.409334922 |
| cg18101152 | chr8 | 31230766 | 31240918 | gene_id "L1FLnI_8p12b" | 7.604915886 |
| cg16678267 | chr8 | 32032759 | 32041929 | gene_id "L1FLnI_8p12i" | 0.930033703 |
| cg11497131 | chr8 | 36121490 | 36131848 | gene_id "L1FLnI_8p12ia" | 1.638540473 |
| cg21206021 | chr8 | 36121490 | 36131848 | gene_id "L1FLnI_8p12ia" | -1.402714002 |
| cg11809056 | chr8 | 27548894 | 27558935 | gene_id "L1FLnI_8p21.1" | 0.890983078 |
| cg25032722 | chr8 | 15555510 | 15565528 | gene_id "L1FLnI_8p22e" | -0.047657477 |
| cg02578584 | chr8 | 8467361 | 8477387 | gene_id "L1FLnI_8p23.1c" | 0.196772993 |
| cg04138721 | chr8 | 11402389 | 11411608 | gene_id "L1FLnI_8p23.1g" | 0.093088825 |
| cg26544317 | chr8 | 11402389 | 11411608 | gene_id "L1FLnI_8p23.1g" | -0.965862503 |
| cg00552087 | chr8 | 3449814 | 3459944 | gene_id "L1FLnI_8p23.2i" | -0.416228391 |
| cg22327666 | chr8 | 47296959 | 47307092 | gene_id "L1FLnI_8q11.21a" | -3.892581926 |
| cg17010309 | chr8 | 47343470 | 47353603 | gene_id "L1FLnI_8q11.21c" | -1.78081743 |
| cg03449315 | chr8 | 47499143 | 47509242 | gene_id "L1FLnI_8q11.21g" | -0.152060561 |
| cg13067992 | chr8 | 53365512 | 53375653 | gene_id "L1FLnI_8q11.23i" | 0.410179187 |
| cg03517476 | chr8 | 62237027 | 62246189 | gene_id "L1FLnI_8q12.3f" | -1.732348531 |
| cg10218002 | chr8 | 72839414 | 72849812 | gene_id "L1FLnI_8q21.11m" | 0.223950709 |
| cg04178156 | chr8 | 89334897 | 89344596 | gene_id "L1FLnI_8q21.3s" | 7.321118879 |
| cg16721359 | chr8 | 97589087 | 97599226 | gene_id "L1FLnI_8q22.1ma" | 6.852618494 |
| cg18569825 | chr8 | 98583599 | 98593622 | gene_id "L1FLnI_8q22.2c" | -1.425690918 |
| cg00090261 | chr8 | 1E+08 | 100114294 | gene_id "L1FLnI_8q22.2s" | 1.233204844 |
| cg06569139 | chr8 | 1E+08 | 100114294 | gene_id "L1FLnI_8q22.2s" | 0.88351337 |
| cg08590069 | chr8 | 1E+08 | 100114294 | gene_id "L1FLnI_8q22.2s" | -0.444763811 |
| cg12648537 | chr8 | 1E+08 | 100114294 | gene_id "L1FLnI_8q22.2s" | 9.313369755 |
| cg17168157 | chr8 | 1E+08 | 100114294 | gene_id "L1FLnI_8q22.2s" | -0.969087918 |
| cg20051292 | chr8 | 1E+08 | 100114294 | gene_id "L1FLnI_8q22.2s" | 1.598594733 |
| cg20219381 | chr8 | 1E+08 | 100114294 | gene_id "L1FLnI_8q22.2s" | 20.56077223 |
| cg20976526 | chr8 | 1E+08 | 100114294 | gene_id "L1FLnI_8q22.2s" | 0.770181249 |
| cg23208513 | chr8 | 1E+08 | 100114294 | gene_id "L1FLnI_8q22.2s" | 2.122056522 |
| cg24848035 | chr8 | 1E+08 | 100114294 | gene_id "L1FLnI_8q22.2s" | 1.276897357 |
| cg01950192 | chr8 | 1.01E+08 | 100858965 | gene_id "L1FLnI_8q22.3a" | 0.057897671 |
| cg21957720 | chr8 | 1.04E+08 | 103712110 | gene_id "L1FLnI_8q22.3o" | -1.909825439 |
| cg14390884 | chr8 | 1.15E+08 | 114640815 | gene_id "L1FLnI_8q23.3x" | 0.624161747 |
| cg16332413 | chr8 | 1.24E+08 | 124135971 | gene_id "L1FLnI_8q24.13l" | 2.037214051 |
| cg20364222 | chr8 | 1.24E+08 | 124249502 | gene_id "L1FLnI_8q24.13m" | 1.285857513 |
| cg27599856 | chr8 | 1.3E+08 | 129524364 | gene_id "L1FLnI_8q24.21s" | -1.846744453 |
| cg15200648 | chr8 | 1.38E+08 | 138122724 | gene_id "L1FLnI_8q24.23k" | 1.442957266 |
| cg04988869 | chr9 | 41257616 | 41267636 | gene_id "L1FLnI_9p11.2f" | 0.50757345 |
| cg13731106 | chr9 | 39783194 | 39791701 | gene_id "L1FLnI_9p12c" | 2.334149577 |
| cg12288452 | chr9 | 34922557 | 34931381 | gene_id "L1FLnI_9p13.3e" | -0.339624934 |
| cg21961472 | chr9 | 33081951 | 33092067 | gene_id "L1FLnI_9p21.1ma" | 1.182113465 |
| cg14029791 | chr9 | 31850014 | 31860495 | gene_id "L1FLnI_9p21.1v" | 1.901354825 |
| cg13913108 | chr9 | 27247686 | 27256312 | gene_id "L1FLnI_9p21.2l" | 1.080994172 |
| cg03946645 | chr9 | 22173603 | 22183645 | gene_id "L1FLnI_9p21.3k" | 2.767495851 |
| cg19186428 | chr9 | 22219840 | 22229985 | gene_id "L1FLnI_9p21.3m" | -1.891371191 |
| cg01187279 | chr9 | 18557526 | 18567971 | gene_id "L1FLnI_9p22.1a" | 0.418097701 |
| cg02539082 | chr9 | 16219585 | 16229954 | gene_id "L1FLnI_9p22.3r" | 0.483861948 |
| cg15866162 | chr9 | 4602301 | 4612311 | gene_id "L1FLnI_9p24.1a" | -0.972642741 |
| cg15274195 | chr9 | 4914575 | 4924690 | gene_id "L1FLnI_9p24.1c" | 4.621538293 |
| cg20534846 | chr9 | 8186094 | 8197200 | gene_id "L1FLnI_9p24.1ca" | 4.972772326 |
| cg04796432 | chr9 | 5497966 | 5508013 | gene_id "L1FLnI_9p24.1k" | -0.856661804 |
| cg04900438 | chr9 | 6132349 | 6142350 | gene_id "L1FLnI_9p24.1n" | 1.54228463 |
| cg21901459 | chr9 | 6132349 | 6142350 | gene_id "L1FLnI_9p24.1n" | 1.690877404 |
| cg13856127 | chr9 | 4527573 | 4537700 | gene_id "L1FLnI_9p24.2p" | 0.013933796 |
| cg05316290 | chr9 | 69413232 | 69423290 | gene_id "L1FLnI_9q21.12a" | 0.487291653 |
| cg15274823 | chr9 | 69413232 | 69423290 | gene_id "L1FLnI_9q21.12a" | -0.105762077 |
| cg07964356 | chr9 | 72068697 | 72077257 | gene_id "L1FLnI_9q21.13d" | -0.215232024 |
| cg13127973 | chr9 | 85071167 | 85081178 | gene_id "L1FLnI_9q21.33b" | 1.83252424 |
| cg04154380 | chr9 | 87688840 | 87698516 | gene_id "L1FLnI_9q21.33q" | 1.031348846 |
| cg04730691 | chr9 | 94113037 | 94123065 | gene_id "L1FLnI_9q22.32d" | 3.45332251 |
| cg15399577 | chr9 | 98504779 | 98514899 | gene_id "L1FLnI_9q22.33i" | 0.196598572 |
| ch.9.1518492F | chr9 | 1E+08 | 100263050 | gene_id "L1FLnI_9q31.1d" | -0.447854952 |
| cg21177713 | chr9 | 1.03E+08 | 102604467 | gene_id "L1FLnI_9q31.1t" | 0.614674758 |
| cg13113147 | chr9 | 1.06E+08 | 105903949 | gene_id "L1FLnI_9q31.2f" | -0.227721486 |
| cg07709224 | chrX | 56057842 | 56067413 | gene_id "L1FLnI_Xp11.21ca" | 0.056241502 |
| cg13011976 | chrX | 55066562 | 55077014 | gene_id "L1FLnI_Xp11.21h" | 0.051634297 |
| cg18983126 | chrX | 55066562 | 55077014 | gene_id "L1FLnI_Xp11.21h" | 0.01966982 |
| cg26239772 | chrX | 55066562 | 55077014 | gene_id "L1FLnI_Xp11.21h" | 0.03631178 |
| cg05598541 | chrX | 52842974 | 52853171 | gene_id "L1FLnI_Xp11.22ga" | 0.383357081 |
| cg04188486 | chrX | 46651859 | 46661885 | gene_id "L1FLnI_Xp11.3ja" | 0.816575523 |
| cg05549246 | chrX | 23422813 | 23432945 | gene_id "L1FLnI_Xp22.11h" | 0.334065544 |
| cg01498090 | chrX | 12857808 | 12867941 | gene_id "L1FLnI_Xp22.2h" | 1.212246663 |
| cg08029608 | chrX | 12857808 | 12867941 | gene_id "L1FLnI_Xp22.2h" | 0.632966677 |
| cg08840366 | chrX | 13721128 | 13730126 | gene_id "L1FLnI_Xp22.2j" | 2.413730278 |
| cg18412780 | chrX | 65214681 | 65223762 | gene_id "L1FLnI_Xq11.2oa" | 0.616914168 |
| cg16046226 | chrX | 66400319 | 66409145 | gene_id "L1FLnI_Xq12m" | -0.266281234 |
| cg09093493 | chrX | 76254872 | 76265136 | gene_id "L1FLnI_Xq13.3ta" | 2.023489246 |
| cg08772684 | chrX | 76975113 | 76985281 | gene_id "L1FLnI_Xq21.1e" | -1.400611517 |
| cg04204138 | chrX | 82212640 | 82224337 | gene_id "L1FLnI_Xq21.1kd" | -3.411837008 |
| cg15533646 | chrX | 83747002 | 83757148 | gene_id "L1FLnI_Xq21.1ne" | 0.139936328 |
| cg04479192 | chrX | 94397217 | 94405724 | gene_id "L1FLnI_Xq21.33a" | 0.929067257 |
| cg26112687 | chrX | 1.16E+08 | 115967266 | gene_id "L1FLnI_Xq23bb" | 0.386178281 |
| cg01095867 | chrX | 1.21E+08 | 120599361 | gene_id "L1FLnI_Xq24u" | 0.339215352 |
| cg24634676 | chrX | 1.22E+08 | 122369178 | gene_id "L1FLnI_Xq25f" | 0.007908863 |
| cg09968214 | chrX | 1.27E+08 | 126777207 | gene_id "L1FLnI_Xq25ua" | -0.465214962 |
| cg05302779 | chrX | 1.45E+08 | 145398739 | gene_id "L1FLnI_Xq27.3o" | 0.046586607 |
| cg05608794 | chrY | 12534289 | 12544485 | gene_id "L1FLnI_Yq11.221a" | -0.418778954 |
| cg09552983 | chr3 | 1.79E+08 | 178866993 | gene_id "L1ORF2_3q26.32b" | 0.017276037 |
| cg20445932 | chr4 | 1.23E+08 | 122659695 | gene_id "L1ORF2_4q27b" | 4.829930232 |
| cg11789321 | chr5 | 57470575 | 57478624 | gene_id "L1ORF2_5q11.2" | 6.33195019 |
| cg04879818 | chr7 | 29578934 | 29586977 | gene_id "L1ORF2_7p14.3" | 1.781246539 |
| cg00241482 | chr8 | 72478433 | 72486477 | gene_id "L1ORF2_8q21.11" | 0.096588222 |
| cg07732827 | chr10 | 18006456 | 18008712 | gene_id ERV316A3_10p12.33 | 1.733796255 |
| cg17310173 | chr10 | 7813000 | 7816802 | gene_id ERV316A3_10p14a | 0.538395646 |
| cg25382740 | chr10 | 47288922 | 47291726 | gene_id ERV316A3_10q11.22a | -0.99503064 |
| cg27477883 | chr10 | 58415047 | 58416306 | gene_id ERV316A3_10q21.1g | 0.156526597 |
| cg02688359 | chr11 | 2072427 | 2073468 | gene_id ERV316A3_11p15.5b | -0.795295861 |
| cg09342225 | chr11 | 1.02E+08 | 101798452 | gene_id ERV316A3_11q22.1h | -0.891413583 |
| cg12833422 | chr11 | 1.18E+08 | 118302403 | gene_id ERV316A3_11q23.3c | -0.210548785 |
| cg14076394 | chr11 | 1.33E+08 | 132588924 | gene_id ERV316A3_11q25b | -4.64413294 |
| cg21019638 | chr12 | 45546734 | 45548929 | gene_id ERV316A3_12q12e | 1.162416459 |
| cg03041038 | chr12 | 90278138 | 90284377 | gene_id ERV316A3_12q21.33c | 3.523563319 |
| cg21733098 | chr12 | 1.27E+08 | 127448062 | gene_id ERV316A3_12q24.32c | -1.465432953 |
| cg00834519 | chr13 | 26628531 | 26629975 | gene_id ERV316A3_13q12.13b | 1.708740894 |
| cg09011602 | chr13 | 26628531 | 26629975 | gene_id ERV316A3_13q12.13b | 0.154031023 |
| cg14558712 | chr13 | 26628531 | 26629975 | gene_id ERV316A3_13q12.13b | -0.643459305 |
| cg11270449 | chr13 | 46094507 | 46098944 | gene_id ERV316A3_13q14.13 | -6.39191442 |
| cg15366524 | chr14 | 22266973 | 22269032 | gene_id ERV316A3_14q11.2g | -0.029270413 |
| cg11363745 | chr14 | 39483890 | 39486026 | gene_id ERV316A3_14q21.1b | 1.792596871 |
| cg25030604 | chr14 | 39483890 | 39486026 | gene_id ERV316A3_14q21.1b | 0.227459066 |
| cg22799691 | chr14 | 1.03E+08 | 103355857 | gene_id ERV316A3_14q32.32 | 0.230155703 |
| cg27229520 | chr15 | 51995047 | 51996386 | gene_id ERV316A3_15q21.2b | -4.072478862 |
| cg13990618 | chr15 | 79540213 | 79543477 | gene_id ERV316A3_15q25.1c | -1.316537902 |
| cg05504820 | chr15 | 99963111 | 99968055 | gene_id ERV316A3_15q26.3a | 4.033711812 |
| cg04000927 | chr16 | 58247275 | 58248778 | gene_id ERV316A3_16q21a | 0.916841788 |
| cg15535507 | chr17 | 79904359 | 79906563 | gene_id ERV316A3_17q25.3b | -5.507407949 |
| cg20202132 | chr17 | 79904359 | 79906563 | gene_id ERV316A3_17q25.3b | 0.958482708 |
| cg25447290 | chr18 | 11527168 | 11529434 | gene_id ERV316A3_18p11.21b | -0.544394998 |
| cg19443833 | chr1 | 1.02E+08 | 102176687 | gene_id ERV316A3_1p21.1a | 0.028133 |
| cg22652144 | chr1 | 49626047 | 49632050 | gene_id ERV316A3_1p33 | -3.413081428 |
| cg11790706 | chr1 | 26145639 | 26147087 | gene_id ERV316A3_1p36.11b | -0.61067082 |
| cg24937877 | chr1 | 11383219 | 11385505 | gene_id ERV316A3_1p36.22b | -1.192138667 |
| cg02021559 | chr1 | 1.51E+08 | 151503350 | gene_id ERV316A3_1q21.3a | -11.29019761 |
| cg16341115 | chr1 | 1.54E+08 | 154306031 | gene_id ERV316A3_1q21.3e | 0.752771007 |
| cg03174876 | chr1 | 2.16E+08 | 216195860 | gene_id ERV316A3_1q41c | -2.695464323 |
| cg11841031 | chr20 | 46432835 | 46435517 | gene_id ERV316A3_20q13.12 | -1.481803068 |
| cg17066785 | chr20 | 46432835 | 46435517 | gene_id ERV316A3_20q13.12 | 0.866015315 |
| cg13813474 | chr21 | 39710767 | 39715002 | gene_id ERV316A3_21q22.2c | -2.267330442 |
| cg10889196 | chr21 | 41653581 | 41657285 | gene_id ERV316A3_21q22.3a | -0.574335329 |
| cg09387528 | chr21 | 46451755 | 46458103 | gene_id ERV316A3_21q22.3e | -1.216669719 |
| cg19921642 | chr22 | 37287909 | 37290999 | gene_id ERV316A3_22q13.1 | 0.008285534 |
| cg19246944 | chr2 | 57695895 | 57699454 | gene_id ERV316A3_2p16.1e | 7.276383918 |
| cg18303589 | chr2 | 8296771 | 8302648 | gene_id ERV316A3_2p25.1a | -6.245730185 |
| cg11184392 | chr2 | 1.8E+08 | 180014127 | gene_id ERV316A3_2q31.3b | -0.391237097 |
| cg18604678 | chr2 | 1.94E+08 | 193845885 | gene_id ERV316A3_2q32.3b | 1.133388676 |
| cg04833392 | chr2 | 2.16E+08 | 216036830 | gene_id ERV316A3_2q35f | -0.366026476 |
| cg00038760 | chr3 | 1.59E+08 | 159193972 | gene_id ERV316A3_3q25.32b | -0.532603597 |
| cg03024690 | chr3 | 1.73E+08 | 172552486 | gene_id ERV316A3_3q26.31 | -0.021651053 |
| cg06287945 | chr3 | 1.85E+08 | 184542173 | gene_id ERV316A3_3q27.1c | -5.116824985 |
| cg09485313 | chr4 | 1.17E+08 | 116704058 | gene_id ERV316A3_4q26g | -0.521029161 |
| cg20003377 | chr4 | 1.49E+08 | 149031932 | gene_id ERV316A3_4q31.23c | 0.922652664 |
| cg16180117 | chr4 | 1.88E+08 | 187650752 | gene_id ERV316A3_4q35.2c | 2.759539583 |
| cg08596532 | chr5 | 5032844 | 5034464 | gene_id ERV316A3_5p15.32c | 0.228429145 |
| cg16550849 | chr5 | 53259162 | 53265410 | gene_id ERV316A3_5q11.2d | -0.501077837 |
| cg22006408 | chr5 | 65120559 | 65123334 | gene_id ERV316A3_5q12.3c | 2.552152294 |
| cg27032781 | chr5 | 79228212 | 79231720 | gene_id ERV316A3_5q14.1 | -1.756359312 |
| cg20409328 | chr5 | 1.05E+08 | 104774091 | gene_id ERV316A3_5q21.2c | 1.107403888 |
| cg12188634 | chr5 | 1.41E+08 | 141274064 | gene_id ERV316A3_5q31.3a | 0.511151549 |
| cg16261259 | chr5 | 1.53E+08 | 152506682 | gene_id ERV316A3_5q33.1 | -0.241039542 |
| cg18798702 | chr5 | 1.78E+08 | 177936750 | gene_id ERV316A3_5q35.3a | 0.400905049 |
| cg00218406 | chr6 | 31463420 | 31469043 | gene_id ERV316A3_6p21.33c | 0.138023185 |
| cg18808777 | chr6 | 31463420 | 31469043 | gene_id ERV316A3_6p21.33c | 1.286216662 |
| cg10139151 | chr6 | 29579631 | 29584117 | gene_id ERV316A3_6p22.1f | 0.152327639 |
| cg05973840 | chr6 | 29793128 | 29794351 | gene_id ERV316A3_6p22.1i | -0.287861254 |
| cg20912517 | chr6 | 29793128 | 29794351 | gene_id ERV316A3_6p22.1i | 0.426220168 |
| cg23961091 | chr6 | 29793128 | 29794351 | gene_id ERV316A3_6p22.1i | 0.189451461 |
| cg06596503 | chr6 | 29868623 | 29874102 | gene_id ERV316A3_6p22.1k | -0.028789383 |
| cg06922212 | chr6 | 29868623 | 29874102 | gene_id ERV316A3_6p22.1k | 0.089297396 |
| cg10197332 | chr6 | 17265730 | 17270638 | gene_id ERV316A3_6p22.3a | -0.697694801 |
| cg01157718 | chr6 | 3252869 | 3256853 | gene_id ERV316A3_6p25.2c | 0.005744329 |
| cg11464312 | chr6 | 86487105 | 86490539 | gene_id ERV316A3_6q14.3d | 0.283349575 |
| cg02266736 | chr6 | 93837870 | 93842822 | gene_id ERV316A3_6q16.1a | 0.57005401 |
| cg21887487 | chr6 | 1.32E+08 | 132164412 | gene_id ERV316A3_6q23.2b | -0.600335045 |
| cg22910439 | chr6 | 1.5E+08 | 150003708 | gene_id ERV316A3_6q25.1g | -2.520037962 |
| cg14614117 | chr7 | 22656846 | 22661312 | gene_id ERV316A3_7p15.3b | 0.126332252 |
| cg18904170 | chr7 | 22656846 | 22661312 | gene_id ERV316A3_7p15.3b | 0.815993924 |
| cg08167638 | chr7 | 79652420 | 79654628 | gene_id ERV316A3_7q21.11a | -1.747136202 |
| cg23498494 | chr7 | 90462841 | 90467565 | gene_id ERV316A3_7q21.13 | 1.061728436 |
| cg14206855 | chr7 | 1.39E+08 | 139113489 | gene_id ERV316A3_7q34a | -3.25456331 |
| cg22787151 | chr7 | 1.56E+08 | 156194382 | gene_id ERV316A3_7q36.3b | 0.155580228 |
| cg01075715 | chr8 | 32951528 | 32953770 | gene_id ERV316A3_8p12f | 0.455382741 |
| cg21290162 | chr8 | 9882724 | 9884677 | gene_id ERV316A3_8p23.1c | 0.470185374 |
| cg12377591 | chr8 | 96735582 | 96736748 | gene_id ERV316A3_8q22.1d | 6.153001757 |
| cg18515940 | chr8 | 1.06E+08 | 106104556 | gene_id ERV316A3_8q23.1a | -0.797185919 |
| cg14611488 | chr8 | 1.36E+08 | 136118334 | gene_id ERV316A3_8q24.23c | 5.120556518 |
| cg03739470 | chr8 | 1.44E+08 | 143665455 | gene_id ERV316A3_8q24.3j | 0.483228441 |
| cg07615237 | chr9 | 1526655 | 1530079 | gene_id ERV316A3_9p24.3d | 1.829210849 |
| cg11328558 | chr9 | 1.26E+08 | 125613700 | gene_id ERV316A3_9q33.3 | -0.325217702 |
| cg05548968 | chrX | 30910129 | 30913573 | gene_id ERV316A3_Xp21.2 | -5.818635955 |
| cg02418648 | chrX | 1.5E+08 | 149524641 | gene_id ERV316A3_Xq28b | 0.159734066 |
| cg00508950 | chr12 | 1.32E+08 | 132340999 | gene_id ERVL_12q24.33 | -0.280352347 |
| cg11854777 | chr2 | 1.31E+08 | 130840847 | gene_id ERVL_2q21.1 | -0.361729645 |
| cg09476073 | chr11 | 2292785 | 2296646 | gene_id ERVLB4_11p15.5c | 3.17682134 |
| cg12500951 | chr11 | 2292785 | 2296646 | gene_id ERVLB4_11p15.5c | -2.005919561 |
| cg05369707 | chr11 | 2354004 | 2362091 | gene_id ERVLB4_11p15.5e | 0.844389496 |
| cg12121166 | chr11 | 2354004 | 2362091 | gene_id ERVLB4_11p15.5e | -2.156486556 |
| cg23951380 | chr11 | 96697533 | 96700987 | gene_id ERVLB4_11q21c | 1.166605214 |
| cg20680308 | chr12 | 45584263 | 45588528 | gene_id ERVLB4_12q12d | 0.365302216 |
| cg21399301 | chr15 | 31448070 | 31450841 | gene_id ERVLB4_15q13.3a | 3.891263147 |
| cg21814142 | chr16 | 59590524 | 59591669 | gene_id ERVLB4_16q21a | 1.223636002 |
| cg16936867 | chr17 | 986091 | 987370 | gene_id ERVLB4_17p13.3a | -0.303338501 |
| cg07803836 | chr17 | 987594 | 988954 | gene_id ERVLB4_17p13.3b | -0.477875139 |
| cg14143385 | chr17 | 74540590 | 74541302 | gene_id ERVLB4_17q25.1 | -0.930057953 |
| cg08868257 | chr1 | 1.73E+08 | 173340404 | gene_id ERVLB4_1q25.1b | 4.604076886 |
| cg12038299 | chr1 | 2.39E+08 | 238630665 | gene_id ERVLB4_1q43a | -2.256004397 |
| cg04512780 | chr20 | 54817566 | 54819424 | gene_id ERVLB4_20q13.2 | 2.797096499 |
| cg21054147 | chr22 | 49097803 | 49099489 | gene_id ERVLB4_22q13.32a | -2.976633516 |
| cg05632274 | chr2 | 58005706 | 58007331 | gene_id ERVLB4_2p16.1d | 3.394495459 |
| cg23229329 | chr2 | 3267103 | 3270169 | gene_id ERVLB4_2p25.3 | 0.064824701 |
| cg12709335 | chr2 | 1E+08 | 100179071 | gene_id ERVLB4_2q11.2 | -0.952000641 |
| cg05167606 | chr2 | 1.27E+08 | 126528169 | gene_id ERVLB4_2q14.3h | -2.786952116 |
| cg12451887 | chr3 | 1.83E+08 | 183439428 | gene_id ERVLB4_3q27.1a | 2.048868987 |
| cg03131527 | chr3 | 1.98E+08 | 197574968 | gene_id ERVLB4_3q29b | 0.604013352 |
| cg22813783 | chr4 | 35252787 | 35254223 | gene_id ERVLB4_4p15.1e | 0.958176575 |
| cg15909724 | chr4 | 77428273 | 77433435 | gene_id ERVLB4_4q21.1a | 1.334192404 |
| cg15551284 | chr4 | 1.6E+08 | 160320164 | gene_id ERVLB4_4q32.1d | 1.973291937 |
| cg21481321 | chr5 | 14660212 | 14663052 | gene_id ERVLB4_5p15.2b | -3.555515178 |
| cg26517012 | chr5 | 1860328 | 1865142 | gene_id ERVLB4_5p15.33a | -0.069074605 |
| cg11305401 | chr6 | 1.5E+08 | 150334582 | gene_id ERVLB4_6q25.1 | 1.480618915 |
| cg08412584 | chr6 | 1.66E+08 | 165585503 | gene_id ERVLB4_6q27c | -0.736228008 |
| cg26967792 | chr7 | 53188928 | 53189794 | gene_id ERVLB4_7p12.1f | -0.106042241 |
| cg00954167 | chr7 | 46653888 | 46660477 | gene_id ERVLB4_7p12.3d | 0.752055606 |
| cg11096309 | chr7 | 46653888 | 46660477 | gene_id ERVLB4_7p12.3d | -0.09471432 |
| cg20575288 | chr8 | 1.39E+08 | 139129844 | gene_id ERVLB4_8q24.3a | 2.167776168 |
| cg04134193 | chr9 | 84643747 | 84648345 | gene_id ERVLB4_9q21.33a | -0.112993972 |
| cg10192933 | chr9 | 1.24E+08 | 124434712 | gene_id ERVLB4_9q33.3a | -4.666443533 |
| cg06567996 | chr9 | 1.27E+08 | 126573829 | gene_id ERVLB4_9q33.3b | -2.710351094 |
| cg12190517 | chrX | 74678921 | 74684880 | gene_id ERVLB4_Xq13.2b | 0.088772232 |
| cg05371273 | chr10 | 37784211 | 37786335 | gene_id ERVLE_10p11.21 | 4.668394178 |
| cg03162820 | chr10 | 23337763 | 23339497 | gene_id ERVLE_10p12.2a | 1.993874384 |
| cg22965561 | chr10 | 13003971 | 13007597 | gene_id ERVLE_10p13b | 0.199062577 |
| cg19556123 | chr10 | 62421204 | 62423037 | gene_id ERVLE_10q21.2 | 0.113016824 |
| cg27433764 | chr10 | 85510511 | 85511904 | gene_id ERVLE_10q23.1g | -0.172598033 |
| cg11534335 | chr11 | 43874661 | 43878914 | gene_id ERVLE_11p11.2b | 1.44853553 |
| cg02014334 | chr11 | 359363 | 366821 | gene_id ERVLE_11p15.5a | -0.87619362 |
| cg16222491 | chr11 | 359363 | 366821 | gene_id ERVLE_11p15.5a | -0.086022703 |
| cg18398175 | chr11 | 359363 | 366821 | gene_id ERVLE_11p15.5a | -6.619496257 |
| cg21547628 | chr11 | 359363 | 366821 | gene_id ERVLE_11p15.5a | -0.824406439 |
| cg14994916 | chr11 | 1360002 | 1364808 | gene_id ERVLE_11p15.5b | -0.20159914 |
| cg12278739 | chr11 | 69021006 | 69028229 | gene_id ERVLE_11q13.3a | 2.47300429 |
| cg13545097 | chr11 | 70524189 | 70525264 | gene_id ERVLE_11q13.4a | 2.533395096 |
| cg11677453 | chr11 | 1.25E+08 | 124717588 | gene_id ERVLE_11q24.2a | 3.365245528 |
| cg09586300 | chr12 | 17974961 | 17976508 | gene_id ERVLE_12p12.3f | -1.519177569 |
| cg03994477 | chr12 | 2718594 | 2722529 | gene_id ERVLE_12p13.33 | -0.198524976 |
| cg15397233 | chr12 | 67218572 | 67222948 | gene_id ERVLE_12q14.3b | 2.095193643 |
| cg07121221 | chr12 | 99916729 | 99918296 | gene_id ERVLE_12q23.1c | -0.049889681 |
| cg03212451 | chr13 | 67493040 | 67494354 | gene_id ERVLE_13q21.32c | 0.315305709 |
| cg11446736 | chr13 | 88748389 | 88752248 | gene_id ERVLE_13q31.2d | 1.593936216 |
| cg15239532 | chr14 | 50539759 | 50544229 | gene_id ERVLE_14q22.1 | -3.874502541 |
| cg00007540 | chr14 | 1.03E+08 | 103143466 | gene_id ERVLE_14q32.32b | -0.473779643 |
| cg09164913 | chr17 | 80989236 | 80990718 | gene_id ERVLE_17q25.3b | -4.995529877 |
| cg10070101 | chr17 | 80989236 | 80990718 | gene_id ERVLE_17q25.3b | -0.294833605 |
| cg01395858 | chr18 | 12766939 | 12770774 | gene_id ERVLE_18p11.21b | -0.334993473 |
| cg05882685 | chr18 | 68656500 | 68664413 | gene_id ERVLE_18q22.1f | -4.574625685 |
| cg19491653 | chr19 | 21792238 | 21794236 | gene_id ERVLE_19p12 | -0.005232385 |
| cg10021364 | chr19 | 53864254 | 53865774 | gene_id ERVLE_19q13.42 | -5.137184351 |
| cg17990711 | chr19 | 53864254 | 53865774 | gene_id ERVLE_19q13.42 | -1.668038894 |
| cg26339862 | chr1 | 51165011 | 51169107 | gene_id ERVLE_1p32.3b | 0.206412291 |
| cg09653659 | chr1 | 53477198 | 53481817 | gene_id ERVLE_1p32.3c | -0.41361869 |
| cg07529461 | chr1 | 15982682 | 15983526 | gene_id ERVLE_1p36.13a | -0.014480781 |
| cg22106265 | chr1 | 1.45E+08 | 144594077 | gene_id ERVLE_1q21.1c | 0.11729653 |
| cg18714225 | chr1 | 1.82E+08 | 182411144 | gene_id ERVLE_1q25.3d | 5.153868499 |
| cg13893488 | chr1 | 1.84E+08 | 184259708 | gene_id ERVLE_1q25.3f | -1.738828505 |
| cg12940759 | chr1 | 1.89E+08 | 189431426 | gene_id ERVLE_1q31.1d | -2.575004099 |
| cg07092121 | chr1 | 2.36E+08 | 236139173 | gene_id ERVLE_1q42.3 | -0.468065459 |
| cg08541930 | chr1 | 2.36E+08 | 236139173 | gene_id ERVLE_1q42.3 | 5.966592202 |
| cg09067344 | chr20 | 44281659 | 44289339 | gene_id ERVLE_20q13.12b | 0.713957826 |
| cg18815398 | chr20 | 62873312 | 62876267 | gene_id ERVLE_20q13.33b | 0.024021143 |
| cg09248356 | chr21 | 10330456 | 10332299 | gene_id ERVLE_21p11.2 | 4.433432534 |
| cg00211944 | chr21 | 31064922 | 31067593 | gene_id ERVLE_21q22.11a | -4.380831021 |
| cg01848115 | chr21 | 45630362 | 45633131 | gene_id ERVLE_21q22.3b | 0.453632839 |
| cg15489003 | chr22 | 47859718 | 47861696 | gene_id ERVLE_22q13.31d | -0.280120226 |
| cg20833495 | chr22 | 50583743 | 50587782 | gene_id ERVLE_22q13.33 | 1.329391935 |
| cg07955677 | chr2 | 1.12E+08 | 112262683 | gene_id ERVLE_2q14.1a | -3.201037773 |
| cg14779065 | chr2 | 1.62E+08 | 162076401 | gene_id ERVLE_2q24.2c | 0.960927145 |
| cg06833228 | chr2 | 1.73E+08 | 173124224 | gene_id ERVLE_2q31.1b | -3.873395271 |
| cg17114983 | chr2 | 1.77E+08 | 177339616 | gene_id ERVLE_2q31.2 | 0.16718938 |
| cg16652349 | chr2 | 1.82E+08 | 181582395 | gene_id ERVLE_2q31.3b | 0.676748538 |
| cg11969526 | chr2 | 2.08E+08 | 208282358 | gene_id ERVLE_2q34a | 6.495051143 |
| cg16915426 | chr2 | 2.29E+08 | 228601553 | gene_id ERVLE_2q36.3e | -2.498695451 |
| cg16404144 | chr2 | 2.4E+08 | 240142329 | gene_id ERVLE_2q37.3g | -0.216854846 |
| cg16905434 | chr2 | 2.41E+08 | 240885761 | gene_id ERVLE_2q37.3h | 0.65887835 |
| cg15387636 | chr3 | 81734506 | 81736552 | gene_id ERVLE_3p12.2h | 1.876324893 |
| cg22377480 | chr3 | 35891137 | 35892785 | gene_id ERVLE_3p22.3d | -1.24058892 |
| cg15544868 | chr3 | 31368918 | 31370095 | gene_id ERVLE_3p23 | 2.226123813 |
| cg26153425 | chr3 | 1.09E+08 | 108538368 | gene_id ERVLE_3q13.13b | -0.873251657 |
| cg24134430 | chr3 | 1.1E+08 | 110175415 | gene_id ERVLE_3q13.13d | 1.962389542 |
| cg08825673 | chr3 | 1.22E+08 | 121976641 | gene_id ERVLE_3q13.33d | -5.170042009 |
| cg03927472 | chr3 | 1.48E+08 | 148281792 | gene_id ERVLE_3q24d | -0.240613083 |
| cg26854360 | chr3 | 1.67E+08 | 167325120 | gene_id ERVLE_3q26.1i | 0.297094732 |
| cg02815692 | chr3 | 1.9E+08 | 190218206 | gene_id ERVLE_3q28a | 1.449721242 |
| cg21955864 | chr3 | 1.92E+08 | 191557587 | gene_id ERVLE_3q28b | -1.62611048 |
| cg02413776 | chr4 | 39952513 | 39953383 | gene_id ERVLE_4p14d | 1.285350749 |
| ch.4.658374F | chr4 | 26455516 | 26456067 | gene_id ERVLE_4p15.2d | -33.50699784 |
| cg04152294 | chr4 | 7850687 | 7853092 | gene_id ERVLE_4p16.1c | 1.839567558 |
| cg14207326 | chr4 | 99103433 | 99104536 | gene_id ERVLE_4q23c | -0.580815726 |
| cg14966190 | chr4 | 1.02E+08 | 101820290 | gene_id ERVLE_4q24e | 1.130656065 |
| cg08374659 | chr4 | 1.48E+08 | 147569445 | gene_id ERVLE_4q31.23a | 0.053346903 |
| cg06655097 | chr4 | 1.77E+08 | 176535531 | gene_id ERVLE_4q34.2b | 0.11408211 |
| cg11553575 | chr5 | 24058326 | 24059658 | gene_id ERVLE_5p14.2e | 0.309750298 |
| cg14809891 | chr5 | 5738978 | 5740902 | gene_id ERVLE_5p15.32b | -1.266601945 |
| cg24423574 | chr5 | 99899489 | 99901357 | gene_id ERVLE_5q21.1b | 0.071402415 |
| cg23642407 | chr5 | 1.31E+08 | 131226891 | gene_id ERVLE_5q31.1a | -1.067245379 |
| cg14846627 | chr5 | 1.48E+08 | 147808303 | gene_id ERVLE_5q32 | -0.020205407 |
| cg07548202 | chr6 | 53416949 | 53420757 | gene_id ERVLE_6p12.1a | -0.420008772 |
| cg15729809 | chr6 | 33940363 | 33944337 | gene_id ERVLE_6p21.31a | 0.278237326 |
| ch.6.11766035R | chr6 | 11657132 | 11658462 | gene_id ERVLE_6p24.1a | -19.13460573 |
| cg12725810 | chr6 | 5876874 | 5880937 | gene_id ERVLE_6p25.1a | -0.383283285 |
| cg08714041 | chr6 | 67210631 | 67213729 | gene_id ERVLE_6q12f | 0.650191463 |
| cg08160801 | chr6 | 76684905 | 76689159 | gene_id ERVLE_6q14.1e | 1.560697702 |
| cg02936931 | chr6 | 1.35E+08 | 134536159 | gene_id ERVLE_6q23.2d | 3.657297862 |
| cg11960867 | chr7 | 37655667 | 37657425 | gene_id ERVLE_7p14.1a | -1.907670182 |
| cg11134774 | chr7 | 11612963 | 11614190 | gene_id ERVLE_7p21.3h | 0.444503032 |
| cg03641134 | chr7 | 67231 | 68014 | gene_id ERVLE_7p22.3 | 0.868632442 |
| cg04638650 | chr7 | 66304013 | 66309489 | gene_id ERVLE_7q11.21 | 0.487608501 |
| cg12856369 | chr7 | 66304013 | 66309489 | gene_id ERVLE_7q11.21 | 1.56352772 |
| cg04986922 | chr7 | 1.23E+08 | 123439051 | gene_id ERVLE_7q31.32f | 1.435395058 |
| cg15083851 | chr7 | 1.23E+08 | 123442393 | gene_id ERVLE_7q31.32g | -2.198431331 |
| cg17769731 | chr8 | 12976266 | 12979702 | gene_id ERVLE_8p22a | 0.240877342 |
| cg03805769 | chr8 | 11338021 | 11340451 | gene_id ERVLE_8p23.1e | -1.490228619 |
| cg02968265 | chr8 | 49483636 | 49486584 | gene_id ERVLE_8q11.21b | -3.062223927 |
| cg12786023 | chr8 | 1.02E+08 | 102034477 | gene_id ERVLE_8q22.3a | -0.197819484 |
| cg10892093 | chr8 | 1.36E+08 | 135545070 | gene_id ERVLE_8q24.23a | -3.09443429 |
| cg18397885 | chr9 | 16111273 | 16112772 | gene_id ERVLE_9p22.3b | -1.258290787 |
| cg16243898 | chr9 | 69768513 | 69772563 | gene_id ERVLE_9q21.12 | 1.908119587 |
| cg19591402 | chr9 | 71794050 | 71795363 | gene_id ERVLE_9q21.13b | -2.398509347 |
| cg07872945 | chr9 | 1.34E+08 | 134011797 | gene_id ERVLE_9q34.2 | -7.475576861 |
| cg14295611 | chr9 | 1.34E+08 | 134011797 | gene_id ERVLE_9q34.2 | -9.771082513 |
| cg16214670 | chr9 | 1.35E+08 | 134959257 | gene_id ERVLE_9q34.3a | 0.073063346 |
| cg17403393 | chrX | 1.55E+08 | 154753704 | gene_id ERVLE_Xq28f | -2.339179087 |
| cg13292984 | chr13 | 22883560 | 22889847 | gene_id HARLEQUIN_13q12.12 | 0.031975693 |
| cg06302751 | chr1 | 1.68E+08 | 167869903 | gene_id HARLEQUIN_1q24.2 | 0.105072189 |
| cg24373618 | chr4 | 1.16E+08 | 115956525 | gene_id HARLEQUIN_4q26 | 0.171761179 |
| cg19523937 | chr7 | 5020879 | 5028716 | gene_id HARLEQUIN_7p22.1 | -1.187611173 |
| cg19889856 | chr7 | 1.35E+08 | 135175958 | gene_id HARLEQUIN_7q33a | -0.502413674 |
| cg12670123 | chr10 | 43591986 | 43597077 | gene_id HERV3_10q11.21b | -2.177049297 |
| cg16136467 | chr10 | 43591986 | 43597077 | gene_id HERV3_10q11.21b | -4.55922201 |
| cg12267155 | chr11 | 29518506 | 29527987 | gene_id HERV3_11p14.1 | -3.478337298 |
| cg22787186 | chr11 | 322542 | 324821 | gene_id HERV3_11p15.5 | -0.012576451 |
| cg25490693 | chr11 | 58917283 | 58923130 | gene_id HERV3_11q12.1 | 2.521238026 |
| cg12456014 | chr14 | 1.07E+08 | 106845981 | gene_id HERV3_14q32.33 | -9.351096085 |
| cg13743052 | chr14 | 1.07E+08 | 106845981 | gene_id HERV3_14q32.33 | -7.014603311 |
| cg19901523 | chr14 | 1.07E+08 | 106845981 | gene_id HERV3_14q32.33 | -13.49418533 |
| cg25078225 | chr19 | 21430674 | 21441176 | gene_id HERV3_19p12b | 0.572437846 |
| cg16992340 | chr19 | 22524807 | 22531806 | gene_id HERV3_19p12d | 1.033214139 |
| cg06595206 | chr19 | 49952495 | 49957720 | gene_id HERV3_19q13.33 | -0.060959164 |
| cg18682615 | chr19 | 53433104 | 53438199 | gene_id HERV3_19q13.42b | -1.409372362 |
| cg11022525 | chr21 | 38224043 | 38229805 | gene_id HERV3_21q22.13 | -0.403148752 |
| cg00813264 | chr3 | 1.12E+08 | 112308531 | gene_id HERV3_3q13.2 | 2.827601607 |
| cg03858152 | chr3 | 1.12E+08 | 112308531 | gene_id HERV3_3q13.2 | 0.281840344 |
| cg07633701 | chr3 | 1.12E+08 | 112308531 | gene_id HERV3_3q13.2 | 0.963436801 |
| cg23198707 | chr3 | 1.12E+08 | 112308531 | gene_id HERV3_3q13.2 | 5.564707501 |
| cg23236169 | chr3 | 1.12E+08 | 112308531 | gene_id HERV3_3q13.2 | -3.405351345 |
| cg23799515 | chr4 | 8434382 | 8439749 | gene_id HERV3_4p16.1 | 0.190086057 |
| cg22953682 | chr4 | 68901045 | 68907797 | gene_id HERV3_4q13.2a | -2.908767824 |
| cg26737305 | chr6 | 85863519 | 85867947 | gene_id HERV3_6q14.3b | -0.239987354 |
| cg06513015 | chr7 | 64990324 | 64999936 | gene_id HERV3_7q11.21 | 0.480462731 |
| cg11548184 | chr7 | 1.41E+08 | 140662300 | gene_id HERV3_7q34b | -0.33625466 |
| cg06177459 | chr8 | 53520479 | 53524714 | gene_id HERV3_8q11.23 | 0.1623716 |
| cg19096327 | chr10 | 53025174 | 53030661 | gene_id HERV30_10q21.1 | 0.115773309 |
| cg18173014 | chr3 | 1.62E+08 | 161854038 | gene_id HERV30_3q26.1 | -2.106955394 |
| cg03929825 | chr11 | 94650011 | 94657535 | gene_id HERV4_11q21 | 0.968297817 |
| cg05715688 | chr13 | 99486866 | 99497078 | gene_id HERV4_13q32.3 | -8.657234599 |
| cg14778437 | chr13 | 99486866 | 99497078 | gene_id HERV4_13q32.3 | -1.251122439 |
| cg04188018 | chr17 | 6936343 | 6940666 | gene_id HERV4_17p13.1 | 0.283026215 |
| cg05728951 | chr19 | 58251074 | 58253959 | gene_id HERV4_19q13.43b | -0.336934987 |
| cg26542660 | chr4 | 55938235 | 55948186 | gene_id HERV4_4q12 | -0.217321235 |
| cg15268456 | chr8 | 1.43E+08 | 143180015 | gene_id HERV4_8q24.3 | -0.435384332 |
| cg18790774 | chr12 | 1.31E+08 | 131344796 | gene_id HERV9_12q24.33b | 0.154561863 |
| cg16015139 | chr21 | 17296932 | 17306851 | gene_id HERV9_21q21.1 | 0.795777387 |
| cg25945841 | chr3 | 17774262 | 17782406 | gene_id HERV9_3p24.3 | 1.189873644 |
| cg23365513 | chr10 | 14990821 | 14994887 | gene_id HERVE_10p13b | -0.364995232 |
| cg10312190 | chr11 | 67978497 | 67982552 | gene_id HERVE_11q13.2c | -0.368947596 |
| cg22511706 | chr12 | 8419057 | 8423114 | gene_id HERVE_12p13.31b | 1.236099356 |
| cg04220230 | chr14 | 51748587 | 51752556 | gene_id HERVE_14q22.1 | -0.147897523 |
| cg16940316 | chr14 | 51748587 | 51752556 | gene_id HERVE_14q22.1 | 1.718610342 |
| cg21067079 | chr17 | 28230519 | 28239339 | gene_id HERVE_17q11.2 | -0.101044355 |
| cg05582035 | chr19 | 28606436 | 28615273 | gene_id HERVE_19q12 | 1.122429714 |
| cg00596438 | chr20 | 24927945 | 24933027 | gene_id HERVE_20p11.21b | -3.55253119 |
| cg19217735 | chr2 | 71020400 | 71024471 | gene_id HERVE_2p13.3a | -0.563719173 |
| cg02588277 | chr2 | 64887412 | 64891023 | gene_id HERVE_2p14a | -0.30197046 |
| cg10669266 | chr2 | 64897102 | 64899872 | gene_id HERVE_2p14b | -6.322190956 |
| cg06564069 | chr2 | 2.31E+08 | 231408843 | gene_id HERVE_2q37.1 | -0.507112567 |
| cg13895280 | chr2 | 2.31E+08 | 231408843 | gene_id HERVE_2q37.1 | -4.900398061 |
| cg00995689 | chr3 | 75604058 | 75608125 | gene_id HERVE_3p12.3c | 0.065280791 |
| cg03556366 | chr3 | 8679702 | 8683824 | gene_id HERVE_3p25.3b | -0.235412091 |
| cg27100163 | chr3 | 1.26E+08 | 125743769 | gene_id HERVE_3q21.2c | 0.802161605 |
| cg01394167 | chr4 | 9476315 | 9480369 | gene_id HERVE_4p16.1e | -0.091444576 |
| cg19556341 | chr4 | 4160757 | 4170756 | gene_id HERVE_4p16.3c | 0.346620237 |
| cg24666178 | chr4 | 4160757 | 4170756 | gene_id HERVE_4p16.3c | -1.415317121 |
| cg06857049 | chr5 | 86565179 | 86571036 | gene_id HERVE_5q14.3b | -1.957757986 |
| cg11005571 | chr8 | 30728581 | 30733679 | gene_id HERVE_8p12a | 0.977247789 |
| cg15177668 | chr9 | 90755224 | 90759266 | gene_id HERVE_9q22.2b | 0.309606839 |
| cg22992904 | chrX | 49139563 | 49149008 | gene_id HERVE_Xp11.23 | 0.996687436 |
| cg00333698 | chr19 | 20243740 | 20248488 | gene_id HERVEA_19p12a | -1.730553104 |
| cg10960147 | chr19 | 9725353 | 9730899 | gene_id HERVEA_19p13.2 | 1.167095467 |
| cg01206756 | chr1 | 1.04E+08 | 103704285 | gene_id HERVEA_1p21.1b | 1.056903617 |
| cg26695076 | chr5 | 1.79E+08 | 179055098 | gene_id HERVEA_5q35.3 | 2.167631418 |
| cg05508099 | chr9 | 1.3E+08 | 129594670 | gene_id HERVFH19_9q34.11 | -1.295214216 |
| cg15766011 | chr13 | 54937197 | 54942758 | gene_id HERVFH21_13q21.1 | 0.022490369 |
| cg06604447 | chr14 | 1.02E+08 | 102239533 | gene_id HERVFH21_14q32.31a | -0.375956626 |
| cg19611300 | chr14 | 1.02E+08 | 102239533 | gene_id HERVFH21_14q32.31a | 0.041678688 |
| cg05676261 | chr16 | 30541765 | 30548758 | gene_id HERVFH21_16p11.2 | 2.545115071 |
| cg01002223 | chr1 | 8921052 | 8927668 | gene_id HERVFH21_1p36.23 | -6.035734474 |
| cg03470597 | chr1 | 6560297 | 6570283 | gene_id HERVFH21_1p36.31 | -1.400021617 |
| cg11186386 | chr5 | 29389989 | 29396428 | gene_id HERVFH21_5p13.3 | 0.245362902 |
| cg26516287 | chr7 | 12588442 | 12595509 | gene_id HERVFH21_7p21.3 | 0.60485561 |
| cg20545694 | chr8 | 47988989 | 47999250 | gene_id HERVFH21_8q11.21 | 0.246812326 |
| cg03729128 | chr12 | 56830706 | 56837966 | gene_id HERVFRD_12q13.3 | 3.626838375 |
| cg09278098 | chr12 | 84502043 | 84509596 | gene_id HERVFRD_12q21.31b | -7.750308634 |
| cg16912512 | chr12 | 84502043 | 84509596 | gene_id HERVFRD_12q21.31b | -1.016872279 |
| cg17064825 | chr12 | 84502043 | 84509596 | gene_id HERVFRD_12q21.31b | 0.273297833 |
| cg25164623 | chr12 | 84502043 | 84509596 | gene_id HERVFRD_12q21.31b | -0.326988491 |
| cg04132263 | chr13 | 79393292 | 79394632 | gene_id HERVFRD_13q31.1 | 1.692975445 |
| cg19211853 | chr13 | 79393292 | 79394632 | gene_id HERVFRD_13q31.1 | 0.458435096 |
| cg14883772 | chr14 | 96112157 | 96118463 | gene_id HERVFRD_14q32.2 | 1.709378817 |
| cg16202996 | chr21 | 27721679 | 27727559 | gene_id HERVFRD_21q21.3 | 0.449079281 |
| cg00847387 | chr4 | 1.46E+08 | 145710863 | gene_id HERVFRD_4q31.21 | -1.062435225 |
| cg00966482 | chr6 | 11102745 | 11112217 | gene_id HERVFRD_6p24.2 | 1.836245796 |
| cg01695620 | chr8 | 57209256 | 57210965 | gene_id HERVFRD_8q12.1b | 0.269356809 |
| cg11848882 | chr8 | 61899275 | 61903380 | gene_id HERVFRD_8q12.3 | -0.279462743 |
| cg06679692 | chr10 | 17630037 | 17632161 | gene_id HERVH_10p12.33 | 0.311987706 |
| cg14058291 | chr10 | 1349016 | 1354570 | gene_id HERVH_10p15.3a | 0.020193139 |
| cg26391077 | chr12 | 11609767 | 11615294 | gene_id HERVH_12p13.2c | -0.254830516 |
| cg14086828 | chr19 | 15828738 | 15834725 | gene_id HERVH_19p13.12 | 0.593779852 |
| cg25373246 | chr1 | 2.32E+08 | 232123945 | gene_id HERVH_1q42.2c | 0.452052303 |
| cg07416187 | chr2 | 1.93E+08 | 192513182 | gene_id HERVH_2q32.3b | -3.927119678 |
| cg07077221 | chr3 | 54634484 | 54640204 | gene_id HERVH_3p14.3a | 1.490141778 |
| cg08530824 | chr3 | 32460939 | 32466915 | gene_id HERVH_3p22.3b | 0.75084099 |
| cg11259152 | chr5 | 1.22E+08 | 122478846 | gene_id HERVH_5q23.2a | 1.989107622 |
| cg23010062 | chr5 | 1.36E+08 | 136307022 | gene_id HERVH_5q31.1c | 0.094144697 |
| cg24944500 | chr6 | 67880996 | 67889473 | gene_id HERVH_6q12f | 0.778909733 |
| cg16996746 | chr6 | 1.64E+08 | 164344779 | gene_id HERVH_6q27a | -3.392218797 |
| cg06059332 | chr7 | 1.46E+08 | 146249225 | gene_id HERVH_7q35c | -0.253802589 |
| cg07520860 | chrX | 73801458 | 73809385 | gene_id HERVH_Xq13.2b | 1.402284805 |
| cg26142094 | chr14 | 20830771 | 20836724 | gene_id HERVH48_14q11.2 | 0.023808033 |
| cg07853501 | chr14 | 70344652 | 70350485 | gene_id HERVH48_14q24.2 | -1.725537644 |
| cg16086098 | chr1 | 2.47E+08 | 246606819 | gene_id HERVH48_1q44a | -0.640413621 |
| cg07362365 | chr21 | 42916742 | 42925876 | gene_id HERVH48_21q22.3 | -1.486125203 |
| cg01146990 | chr5 | 43072721 | 43075673 | gene_id HERVH48_5p12 | 1.680879942 |
| cg06620665 | chr3 | 51737952 | 51742260 | gene_id HERVI_3p21.2 | 3.13572063 |
| cg09436355 | chr7 | 1.35E+08 | 134548478 | gene_id HERVI_7q33a | 2.326090272 |
| cg14588352 | chr11 | 67689713 | 67696211 | gene_id HERVIP10F_11q13.2 | -0.283686047 |
| cg25641855 | chr2 | 1.08E+08 | 108405283 | gene_id HERVIP10F_2q12.3 | 0.693574188 |
| cg00652158 | chr3 | 48272779 | 48278831 | gene_id HERVIP10F_3p21.31 | -1.199821735 |
| cg18074165 | chr3 | 48272779 | 48278831 | gene_id HERVIP10F_3p21.31 | -0.904013642 |
| cg26433232 | chr9 | 26972599 | 26976760 | gene_id HERVIP10F_9p21.2b | 0.072845113 |
| cg10938866 | chrY | 19809366 | 19817999 | gene_id HERVIP10F_Yq11.223 | -0.008674042 |
| cg27593341 | chr12 | 10362141 | 10363332 | gene_id HERVIP10FH_12p13.2 | -2.229081818 |
| cg21315874 | chr15 | 79918724 | 79920814 | gene_id HERVIP10FH_15q25.1 | 0.717552312 |
| cg03726693 | chr19 | 57799186 | 57805431 | gene_id HERVIP10FH_19q13.43 | 3.863412952 |
| cg03688854 | chr20 | 61191606 | 61192917 | gene_id HERVIP10FH_20q13.33b | 0.585061782 |
| cg22540572 | chr21 | 36104268 | 36109248 | gene_id HERVIP10FH_21q22.12 | 1.150803361 |
| cg02827977 | chr2 | 1.22E+08 | 121683452 | gene_id HERVIP10FH_2q14.3b | -0.339601909 |
| cg21761171 | chr3 | 1.21E+08 | 120623770 | gene_id HERVIP10FH_3q13.33 | 1.201201387 |
| cg01306261 | chr5 | 1.43E+08 | 143095011 | gene_id HERVIP10FH_5q31.3 | -0.253034469 |
| cg09223975 | chr6 | 1.59E+08 | 158733323 | gene_id HERVIP10FH_6q25.3 | -0.483143599 |
| cg12796916 | chr11 | 67698550 | 67705411 | gene_id HERVK11_11q13.2 | 1.359113948 |
| cg25855162 | chr19 | 27703454 | 27713310 | gene_id HERVK11_19q11b | 1.774294443 |
| cg20285723 | chr4 | 4042123 | 4049160 | gene_id HERVK11_4p16.3 | 0.173675881 |
| cg24634009 | chr4 | 1.4E+08 | 139639045 | gene_id HERVK11_4q31.1 | -5.241517986 |
| cg25422346 | chr4 | 1.65E+08 | 164753249 | gene_id HERVK11_4q32.3 | 0.633866751 |
| cg26177807 | chr8 | 88670470 | 88676597 | gene_id HERVK11_8q21.3 | 0.0757689 |
| cg25293675 | chr3 | 94647179 | 94651193 | gene_id HERVK11D_3q11.2 | 0.542571571 |
| cg19654323 | chr10 | 1.34E+08 | 133609346 | gene_id HERVK14C_10q26.3 | -0.232768931 |
| cg00300523 | chr19 | 40954173 | 40959190 | gene_id HERVK14C_19q13.2 | -0.816236388 |
| cg17975299 | chr1 | 2.47E+08 | 247111180 | gene_id HERVK14C_1q44 | 0.035613867 |
| cg03545643 | chr8 | 43697571 | 43702581 | gene_id HERVK14C_8p11.1b | -0.380932831 |
| cg12775359 | chr19 | 7796061 | 7801046 | gene_id HERVKC4_19p13.2 | 8.279664598 |
| cg11795728 | chr10 | 87634539 | 87641235 | gene_id HERVL_10q23.2 | -0.693736017 |
| ch.12.602695F | chr12 | 26775547 | 26782199 | gene_id HERVL_12p11.23 | -11.91029043 |
| cg10121097 | chr12 | 11070735 | 11073773 | gene_id HERVL_12p13.2b | 0.210599734 |
| cg22775873 | chr12 | 74034877 | 74040505 | gene_id HERVL_12q21.1c | 0.199887223 |
| cg23838503 | chr22 | 22384866 | 22389982 | gene_id HERVL_22q11.22a | -0.271893754 |
| ch.2.147017622F | chr2 | 1.47E+08 | 146549807 | gene_id HERVL_2q22.3b | -5.5969923 |
| cg04434356 | chr3 | 1.13E+08 | 112700907 | gene_id HERVL_3q13.2 | 0.666180628 |
| cg12503912 | chr4 | 31501329 | 31507892 | gene_id HERVL_4p15.1c | 1.222108805 |
| cg07938676 | chr4 | 62161515 | 62167059 | gene_id HERVL_4q13.1c | 1.335046867 |
| cg20061931 | chr4 | 94152117 | 94159527 | gene_id HERVL_4q22.2 | 1.354492466 |
| cg15160963 | chr5 | 26398325 | 26405152 | gene_id HERVL_5p14.1c | 1.335299975 |
| cg09531389 | chr6 | 68133929 | 68141347 | gene_id HERVL_6q12c | 0.007318962 |
| cg25792710 | chr11 | 21810797 | 21814752 | gene_id HERVL18_11p15.1b | -0.715477383 |
| cg08402708 | chr11 | 85940994 | 85945741 | gene_id HERVL18_11q14.2a | 0.111299006 |
| cg24871877 | chr17 | 60558502 | 60561264 | gene_id HERVL18_17q23.2 | -0.830430043 |
| cg17862961 | chr2 | 60743759 | 60746878 | gene_id HERVL18_2p16.1 | 0.594955939 |
| cg08043779 | chr2 | 1.28E+08 | 127833378 | gene_id HERVL18_2q14.3b | 1.042888316 |
| cg19233088 | chr2 | 1.28E+08 | 127833378 | gene_id HERVL18_2q14.3b | 2.111449261 |
| cg09955928 | chr2 | 1.48E+08 | 148259882 | gene_id HERVL18_2q23.1 | 5.774180609 |
| cg20119370 | chr2 | 1.99E+08 | 199230424 | gene_id HERVL18_2q33.1 | -0.637240611 |
| cg08960706 | chr3 | 1.57E+08 | 156626577 | gene_id HERVL18_3q25.31 | 3.557512953 |
| cg14883930 | chr4 | 1.04E+08 | 103755259 | gene_id HERVL18_4q24b | -7.264815581 |
| cg12319281 | chr4 | 1.16E+08 | 116362242 | gene_id HERVL18_4q26 | 1.869995619 |
| cg11499696 | chr5 | 1.07E+08 | 106876978 | gene_id HERVL18_5q21.3 | 2.841524113 |
| cg15926234 | chr5 | 1.07E+08 | 106876978 | gene_id HERVL18_5q21.3 | 1.65388018 |
| cg01817897 | chr5 | 1.61E+08 | 160938828 | gene_id HERVL18_5q34 | 0.269185762 |
| cg04390328 | chr5 | 1.61E+08 | 160938828 | gene_id HERVL18_5q34 | -0.061104531 |
| cg12212060 | chr6 | 28353865 | 28357249 | gene_id HERVL18_6p22.1c | -0.700986943 |
| cg26655361 | chr6 | 28353865 | 28357249 | gene_id HERVL18_6p22.1c | -0.072348788 |
| cg05882646 | chr6 | 79307594 | 79313548 | gene_id HERVL18_6q14.1 | 0.274701751 |
| cg17715419 | chr6 | 79307594 | 79313548 | gene_id HERVL18_6q14.1 | 4.357133337 |
| cg22311066 | chr6 | 79307594 | 79313548 | gene_id HERVL18_6q14.1 | 3.0895093 |
| cg05239976 | chr6 | 1.22E+08 | 122366684 | gene_id HERVL18_6q22.31a | 1.959942729 |
| cg14048475 | chr6 | 1.22E+08 | 122366684 | gene_id HERVL18_6q22.31a | 1.075117994 |
| cg15483437 | chr9 | 75235558 | 75239484 | gene_id HERVL18_9q21.13 | 1.69560239 |
| cg00729876 | chr9 | 93391121 | 93393828 | gene_id HERVL18_9q22.31 | -1.189492498 |
| cg15401363 | chrX | 76796466 | 76803137 | gene_id HERVL18_Xq13.3 | 0.969483366 |
| cg08516846 | chr14 | 1.06E+08 | 105985336 | gene_id HERVL32_14q32.33a | 0.593803127 |
| cg10187902 | chr14 | 1.06E+08 | 105985336 | gene_id HERVL32_14q32.33a | 0.055990079 |
| cg12754658 | chr4 | 1.23E+08 | 122955311 | gene_id HERVL32_4q28.1b | -2.563292968 |
| cg25106914 | chr10 | 26778654 | 26786076 | gene_id HERVL40_10p12.1 | -0.604801994 |
| cg03631065 | chr13 | 96019147 | 96021285 | gene_id HERVL40_13q32.1 | -2.603082407 |
| cg14519880 | chr14 | 34423073 | 34426443 | gene_id HERVL40_14q13.1a | 0.793707679 |
| cg10136168 | chr14 | 77314097 | 77319837 | gene_id HERVL40_14q24.3 | 3.605106228 |
| cg14372433 | chr14 | 77314097 | 77319837 | gene_id HERVL40_14q24.3 | 1.34224047 |
| cg09488450 | chr20 | 2672035 | 2679386 | gene_id HERVL40_20p13 | 1.711622427 |
| cg08984772 | chr3 | 1.11E+08 | 110870700 | gene_id HERVL40_3q13.13b | -1.169971596 |
| cg02505117 | chr5 | 43149894 | 43159343 | gene_id HERVL40_5p12 | 3.254947605 |
| cg02978414 | chr5 | 18391469 | 18395845 | gene_id HERVL40_5p15.1b | -3.30122031 |
| cg02346475 | chr5 | 86879051 | 86882634 | gene_id HERVL40_5q14.3c | 2.439975583 |
| cg08415137 | chr5 | 86879051 | 86882634 | gene_id HERVL40_5q14.3c | 0.758481621 |
| cg16419764 | chr6 | 4881840 | 4884887 | gene_id HERVL40_6p25.1a | -2.126405288 |
| cg05770389 | chr6 | 88012261 | 88017679 | gene_id HERVL40_6q15 | -6.707633963 |
| cg19892578 | chr6 | 88012261 | 88017679 | gene_id HERVL40_6q15 | 0.086683567 |
| cg22812133 | chr7 | 25249899 | 25252738 | gene_id HERVL40_7p15.3 | -0.567869587 |
| cg03444800 | chr7 | 92403115 | 92404941 | gene_id HERVL40_7q21.2 | 2.983382821 |
| cg26826927 | chr7 | 1.07E+08 | 107465091 | gene_id HERVL40_7q22.3 | -2.576806541 |
| cg18043426 | chr9 | 1E+08 | 100216444 | gene_id HERVL40_9q31.1a | -0.48217186 |
| cg16917713 | chr4 | 22334844 | 22339971 | gene_id HERVL66_4p15.2 | -1.511771911 |
| cg02181349 | chr4 | 1.86E+08 | 186059149 | gene_id HERVL74_4q35.1 | 1.389042249 |
| cg02137970 | chr6 | 3928387 | 3931934 | gene_id HERVL74_6p25.2 | 3.37825363 |
| cg19426609 | chr10 | 1.04E+08 | 104070395 | gene_id HERVP71A_10q25.1 | 1.459051234 |
| cg17585910 | chr12 | 7588093 | 7593696 | gene_id HERVP71A_12p13.31a | -0.014951849 |
| cg18808702 | chr12 | 107124 | 110749 | gene_id HERVP71A_12p13.33a | -0.644071671 |
| cg17127722 | chr15 | 75063487 | 75067944 | gene_id HERVP71A_15q24.2 | -6.70776541 |
| cg13319396 | chr17 | 32969771 | 32974333 | gene_id HERVP71A_17q11.2b | 0.271271267 |
| cg18368431 | chr3 | 46111061 | 46118672 | gene_id HERVP71A_3p21.31 | 1.350148755 |
| cg16550555 | chr3 | 5140823 | 5149820 | gene_id HERVP71A_3p26.1 | 0.326210566 |
| cg19867445 | chr3 | 1.46E+08 | 146339816 | gene_id HERVP71A_3q24 | -6.417696416 |
| cg23363602 | chr6 | 29962489 | 29970366 | gene_id HERVP71A_6p22.1 | 0.287867924 |
| cg22282161 | chr7 | 21547317 | 21556347 | gene_id HERVP71A_7p15.3b | -1.215411001 |
| cg04114754 | chr7 | 1.46E+08 | 146058901 | gene_id HERVP71A_7q35 | -2.184700582 |
| cg06471816 | chr10 | 52946389 | 52956110 | gene_id HERVS71_10q21.1 | -0.158814424 |
| cg20110284 | chr11 | 67882066 | 67889515 | gene_id HERVS71_11q13.2 | 2.822262652 |
| cg11361409 | chr11 | 71705986 | 71713426 | gene_id HERVS71_11q13.4 | 0.152887042 |
| cg03548085 | chr12 | 8317488 | 8324925 | gene_id HERVS71_12p13.31a | 0.202453473 |
| cg04517416 | chr12 | 8317488 | 8324925 | gene_id HERVS71_12p13.31a | 0.297259913 |
| cg05308904 | chr14 | 1.06E+08 | 106207472 | gene_id HERVS71_14q32.33 | 6.851740372 |
| cg10163998 | chr14 | 1.06E+08 | 106207472 | gene_id HERVS71_14q32.33 | 12.53544654 |
| cg13619891 | chr14 | 1.06E+08 | 106207472 | gene_id HERVS71_14q32.33 | 0.513679755 |
| cg19968790 | chr14 | 1.06E+08 | 106207472 | gene_id HERVS71_14q32.33 | -1.976698507 |
| cg07539564 | chr15 | 88538134 | 88546230 | gene_id HERVS71_15q26.1 | 1.409645847 |
| cg07901797 | chr16 | 35897003 | 35902579 | gene_id HERVS71_16p11.1 | 0.616859091 |
| cg16229626 | chr16 | 35897003 | 35902579 | gene_id HERVS71_16p11.1 | -2.293610998 |
| cg11544647 | chr19 | 6656135 | 6661723 | gene_id HERVS71_19p13.3 | 0.190851987 |
| cg18340445 | chr19 | 6656135 | 6661723 | gene_id HERVS71_19p13.3 | 3.291211824 |
| cg06551476 | chr19 | 34552716 | 34561339 | gene_id HERVS71_19q13.11a | 5.306791314 |
| cg11682263 | chr19 | 34721933 | 34727787 | gene_id HERVS71_19q13.11b | 1.874706131 |
| cg26266099 | chr1 | 32923851 | 32926914 | gene_id HERVS71_1p35.1 | -1.544802627 |
| cg21905068 | chr2 | 1.24E+08 | 123683589 | gene_id HERVS71_2q14.3a | 0.649667521 |
| cg03145583 | chr3 | 75500237 | 75507467 | gene_id HERVS71_3p12.3 | -0.66326138 |
| cg22552237 | chr3 | 75500237 | 75507467 | gene_id HERVS71_3p12.3 | 1.321708818 |
| cg27135645 | chr3 | 1.64E+08 | 164341897 | gene_id HERVS71_3q26.1 | 0.708013813 |
| cg12145680 | chr4 | 4030241 | 4037574 | gene_id HERVS71_4p16.3 | 0.373628604 |
| cg01092133 | chr6 | 28766179 | 28776502 | gene_id HERVS71_6p22.1 | 2.660553606 |
| cg03349184 | chr6 | 28766179 | 28776502 | gene_id HERVS71_6p22.1 | 8.704471604 |
| cg12681040 | chr6 | 28766179 | 28776502 | gene_id HERVS71_6p22.1 | 7.59241583 |
| cg16961648 | chr6 | 28766179 | 28776502 | gene_id HERVS71_6p22.1 | 2.13415099 |
| cg23609713 | chr6 | 28766179 | 28776502 | gene_id HERVS71_6p22.1 | 0.456306247 |
| cg16221704 | chr6 | 86473027 | 86481664 | gene_id HERVS71_6q14.3 | 0.103848432 |
| cg12338731 | chr7 | 6988359 | 6995705 | gene_id HERVS71_7p22.1 | 2.763433849 |
| cg00644387 | chr7 | 1.35E+08 | 134557896 | gene_id HERVS71_7q33 | 0.464215259 |
| cg03284117 | chr12 | 14216760 | 14221108 | gene_id HERVW_12p13.1 | -0.022809441 |
| cg21771528 | chr5 | 18742472 | 18746014 | gene_id HERVW_5p14.3 | -1.259289766 |
| cg10669449 | chr6 | 1.06E+08 | 106235814 | gene_id HERVW_6q21a | -3.149947993 |
| cg21071075 | chr11 | 56327845 | 56333225 | gene_id HML1_11q12.1 | 1.514148414 |
| cg00367567 | chr11 | 62368491 | 62370999 | gene_id HML2_11q12.3a | -0.198265422 |
| cg21656108 | chr11 | 62375545 | 62383091 | gene_id HML2_11q12.3b | -0.761664964 |
| cg26483512 | chr14 | 24009696 | 24015776 | gene_id HML2_14q11.2 | 0.004352611 |
| cg08883146 | chr17 | 8056337 | 8063901 | gene_id HML2_17p13.1 | -5.297179259 |
| cg08960549 | chr17 | 8056337 | 8063901 | gene_id HML2_17p13.1 | -4.391844229 |
| cg11630939 | chr17 | 8056337 | 8063901 | gene_id HML2_17p13.1 | -6.146363302 |
| cg19786602 | chr17 | 8056337 | 8063901 | gene_id HML2_17p13.1 | -0.778177607 |
| cg08919930 | chr21 | 18561341 | 18569644 | gene_id HML2_21q21.1 | -0.541461826 |
| cg02098999 | chr22 | 23536062 | 23546900 | gene_id HML2_22q11.23 | -1.65076186 |
| cg27060510 | chr22 | 23536062 | 23546900 | gene_id HML2_22q11.23 | 2.423091586 |
| cg18195328 | chr3 | 75551314 | 75559999 | gene_id HML2_3p12.3 | 0.069086454 |
| cg20586911 | chr4 | 9657956 | 9667550 | gene_id HML2_4p16.1b | -0.03638763 |
| cg04674565 | chr4 | 241200 | 245565 | gene_id HML2_4p16.3a | -6.587020648 |
| cg14387280 | chr6 | 28682591 | 28692958 | gene_id HML2_6p22.1 | 1.863919153 |
| cg23676047 | chr6 | 28682591 | 28692958 | gene_id HML2_6p22.1 | -4.76068293 |
| cg14297546 | chr9 | 1.37E+08 | 136789776 | gene_id HML2_9q34.3 | -1.730499474 |
| cg01130322 | chr10 | 68861092 | 68868584 | gene_id HML3_10q22.1 | 0.549943971 |
| cg05786085 | chr12 | 47443339 | 47448766 | gene_id HML3_12q13.11 | -0.268040125 |
| cg22345048 | chr13 | 20678247 | 20685411 | gene_id HML3_13q12.11b | -1.30338929 |
| cg20517123 | chr16 | 3076839 | 3081509 | gene_id HML3_16p13.3 | 1.762196491 |
| cg22636191 | chr16 | 3076839 | 3081509 | gene_id HML3_16p13.3 | -0.777120178 |
| cg21482921 | chr10 | 5038312 | 5047640 | gene_id HML4_10p15.1 | -8.186565457 |
| cg10451844 | chr17 | 43323118 | 43351750 | gene_id HML4_17q21.31 | 0.523816369 |
| cg24580161 | chr17 | 43323118 | 43351750 | gene_id HML4_17q21.31 | -0.989065393 |
| cg08436120 | chr1 | 3801472 | 3806930 | gene_id HML4_1p36.32 | -0.090418813 |
| cg05697754 | chr7 | 99977146 | 99979562 | gene_id HML4_7q22.1 | -1.834343984 |
| cg17921026 | chr11 | 58925599 | 58929132 | gene_id HML5_11q12.1 | 0.004069428 |
| cg27139424 | chr11 | 58925599 | 58929132 | gene_id HML5_11q12.1 | 2.681247638 |
| cg12396344 | chr12 | 69328937 | 69332487 | gene_id HML5_12q15 | -1.597696815 |
| cg19124816 | chr12 | 69328937 | 69332487 | gene_id HML5_12q15 | -9.385255797 |
| cg22375663 | chr12 | 69328937 | 69332487 | gene_id HML5_12q15 | -1.17632889 |
| cg06159896 | chr12 | 95444445 | 95448153 | gene_id HML5_12q22 | 0.642315194 |
| cg10621809 | chr12 | 95444445 | 95448153 | gene_id HML5_12q22 | 0.489035891 |
| cg10815129 | chr12 | 95444445 | 95448153 | gene_id HML5_12q22 | -2.878032199 |
| cg07639259 | chr12 | 1E+08 | 100165461 | gene_id HML5_12q23.1 | 0.513418142 |
| cg14812628 | chr19 | 27745824 | 27750223 | gene_id HML5_19q11 | 6.520935849 |
| cg15817928 | chr19 | 27745824 | 27750223 | gene_id HML5_19q11 | -0.376739246 |
| cg15227051 | chr6 | 64299000 | 64304246 | gene_id HML5_6q12 | 0.904643717 |
| cg01795886 | chr7 | 1.07E+08 | 107391346 | gene_id HML5_7q22.3 | -1.485463163 |
| cg23362622 | chr7 | 1.07E+08 | 107391346 | gene_id HML5_7q22.3 | -3.84779055 |
| cg20677114 | chr11 | 62049779 | 62056355 | gene_id HML6_11q12.3a | 0.021459271 |
| cg08018888 | chr12 | 1.12E+08 | 111824274 | gene_id HML6_12q24.12a | -0.605370277 |
| cg18096902 | chr13 | 80397537 | 80400338 | gene_id HML6_13q31.1 | -3.65231459 |
| cg26230303 | chr14 | 69811463 | 69816023 | gene_id HML6_14q24.2 | 3.414995324 |
| cg18776840 | chr19 | 21627354 | 21628684 | gene_id HML6_19p12b | -0.178284626 |
| cg03476760 | chr19 | 11621351 | 11627046 | gene_id HML6_19p13.2c | 2.722289132 |
| cg00897744 | chr1 | 2.48E+08 | 248434463 | gene_id HML6_1q44a | 6.141892108 |
| cg20083297 | chr6 | 32559720 | 32567345 | gene_id HML6_6p21.32b | 0.03588769 |
| cg23333970 | chr2 | 96209232 | 96212733 | gene_id HUERSP1_2q11.2 | -0.87019781 |
| cg19378060 | chr3 | 9635796 | 9643306 | gene_id HUERSP1_3p25.3c | 0.971735081 |
| cg08917665 | chr12 | 9707423 | 9711259 | gene_id HUERSP2_12p13.31 | -4.256916323 |
| cg23047760 | chr13 | 58856204 | 58861398 | gene_id HUERSP2_13q21.1 | -0.523493525 |
| cg07895006 | chr1 | 1.99E+08 | 198831700 | gene_id HUERSP2_1q32.1 | 1.603771671 |
| cg12023726 | chr21 | 44485340 | 44491111 | gene_id HUERSP2_21q22.3 | 0.272054955 |
| cg14282096 | chr3 | 1.81E+08 | 180870678 | gene_id HUERSP2_3q26.33 | 0.333158252 |
| cg15367212 | chr3 | 1.81E+08 | 180870678 | gene_id HUERSP2_3q26.33 | 0.163079499 |
| cg14458627 | chr4 | 1.75E+08 | 175255566 | gene_id HUERSP2_4q34.1 | 0.046605552 |
| cg04395846 | chr10 | 37320772 | 37326042 | gene_id HUERSP3_10p11.21 | -0.260252297 |
| cg26004244 | chr11 | 1.26E+08 | 125875038 | gene_id HUERSP3_11q24.2 | 0.622408281 |
| cg21814996 | chr13 | 24995133 | 24998556 | gene_id HUERSP3_13q12.13 | -10.0976088 |
| cg20088835 | chr13 | 96966355 | 96971199 | gene_id HUERSP3_13q32.1 | 0.425217999 |
| cg00180711 | chr19 | 54672643 | 54677704 | gene_id HUERSP3_19q13.42 | -1.511472446 |
| cg20411127 | chr1 | 2.26E+08 | 226494787 | gene_id HUERSP3_1q42.12 | 2.901810019 |
| cg08528940 | chr1 | 2.47E+08 | 246538336 | gene_id HUERSP3_1q44 | -2.180066762 |
| cg26513378 | chr2 | 41493320 | 41502091 | gene_id HUERSP3_2p22.1 | -0.521660245 |
| cg08031509 | chr2 | 1.28E+08 | 127825835 | gene_id HUERSP3_2q14.3 | -0.406681405 |
| cg03067402 | chr2 | 2.28E+08 | 228309450 | gene_id HUERSP3_2q36.3 | -0.686719565 |
| cg19550890 | chr3 | 1.77E+08 | 176515706 | gene_id HUERSP3_3q26.32 | -0.695978164 |
| cg23771785 | chr3 | 1.97E+08 | 196525490 | gene_id HUERSP3_3q29 | 0.495493242 |
| cg24693227 | chr4 | 1.61E+08 | 161257841 | gene_id HUERSP3_4q32.2a | 2.997033978 |
| cg13904130 | chr6 | 33117739 | 33125003 | gene_id HUERSP3_6p21.32 | 0.576570254 |
| cg15737123 | chr6 | 33117739 | 33125003 | gene_id HUERSP3_6p21.32 | -0.037308749 |
| cg16230384 | chr6 | 33117739 | 33125003 | gene_id HUERSP3_6p21.32 | 0.373112365 |
| cg17217478 | chr6 | 33117739 | 33125003 | gene_id HUERSP3_6p21.32 | 0.018311282 |
| cg14109551 | chr6 | 1.19E+08 | 118588950 | gene_id HUERSP3_6q22.31 | -0.517297554 |
| cg26617298 | chr7 | 46463467 | 46471647 | gene_id HUERSP3_7p12.3 | -0.128279283 |
| cg22438656 | chr7 | 64618066 | 64626253 | gene_id HUERSP3_7q11.21a | -0.007008934 |
| cg23873662 | chr7 | 64618066 | 64626253 | gene_id HUERSP3_7q11.21a | 7.519372032 |
| cg24764114 | chr7 | 64951152 | 64953114 | gene_id HUERSP3_7q11.21b | -0.048275524 |
| cg14908202 | chr8 | 39326061 | 39330601 | gene_id HUERSP3_8p11.22 | -0.003432914 |
| cg24082681 | chr8 | 1.11E+08 | 110835968 | gene_id HUERSP3_8q23.2 | 1.045159762 |
| cg24099977 | chr8 | 1.11E+08 | 110835968 | gene_id HUERSP3_8q23.2 | -2.081737978 |
| cg09715925 | chrX | 57816574 | 57823244 | gene_id HUERSP3_Xp11.21 | -0.511072582 |
| cg02447257 | chr16 | 29708769 | 29715758 | gene_id HUERSP3B_16p11.2a | -26.31930977 |
| cg14253018 | chr16 | 29708769 | 29715758 | gene_id HUERSP3B_16p11.2a | -6.810061299 |
| cg20235857 | chr19 | 42790564 | 42794718 | gene_id HUERSP3B_19q13.2 | 0.048699558 |
| cg08121845 | chr3 | 1.04E+08 | 104280102 | gene_id HUERSP3B_3q13.11 | -4.812660951 |
| cg14246539 | chr5 | 28013557 | 28015406 | gene_id HUERSP3B_5p14.1b | 0.755447523 |
| cg18577953 | chr5 | 1.24E+08 | 123930099 | gene_id HUERSP3B_5q23.2a | 1.037560953 |
| cg03582691 | chr1 | 2.22E+08 | 221960186 | gene_id LTR23_1q41 | 1.089366194 |
| cg10676097 | chr19 | 21945796 | 21951669 | gene_id LTR25_19p12a | 0.299170397 |
| cg15736973 | chr1 | 84603880 | 84611675 | gene_id LTR25_1p22.3 | 1.446933924 |
| cg00380930 | chr3 | 1.83E+08 | 182726346 | gene_id LTR25_3q26.33b | 1.074835864 |
| cg14210337 | chr4 | 1.73E+08 | 173289284 | gene_id LTR25_4q34.1a | 2.294947445 |
| cg18805182 | chr5 | 98839880 | 98843293 | gene_id LTR25_5q15b | -0.365117576 |
| cg17323282 | chr11 | 96498781 | 96507527 | gene_id LTR46_11q21 | -4.978050042 |
| cg00042904 | chrX | 63426553 | 63434962 | gene_id LTR46_Xq11.1 | -7.784575133 |
| cg07059712 | chr22 | 36179880 | 36181488 | gene_id LTR57_22q12.3 | -0.755478569 |
| cg27562259 | chr6 | 28868273 | 28875869 | gene_id LTR57_6p22.1 | -0.110308286 |
| cg19551037 | chr11 | 67654639 | 67661572 | gene_id MER101_11q13.2 | -1.414528384 |
| cg07221444 | chr12 | 1.12E+08 | 111953828 | gene_id MER101_12q24.13b | 0.19523165 |
| cg23213327 | chr2 | 6869773 | 6876489 | gene_id MER101_2p25.2 | 0.531984579 |
| cg24955731 | chr7 | 1.49E+08 | 149324081 | gene_id MER101_7q36.1a | 1.776531756 |
| cg05774117 | chr19 | 40299587 | 40304817 | gene_id MER34B_19q13.2 | -3.596108563 |
| cg14709381 | chr7 | 1.59E+08 | 159162646 | gene_id MER34B_7q36.3d | 0.339846135 |
| cg18688299 | chr8 | 41812198 | 41817154 | gene_id MER34B_8p11.21 | 4.60486997 |
| cg17400014 | chr10 | 87796235 | 87803793 | gene_id MER4_10q23.31a | 6.493443366 |
| cg25811448 | chr10 | 90087053 | 90092706 | gene_id MER4_10q23.31b | 0.33162554 |
| cg03800252 | chr12 | 1.13E+08 | 113408713 | gene_id MER4_12q24.13b | 1.650054421 |
| cg25255025 | chr14 | 69745226 | 69747765 | gene_id MER4_14q24.1d | 2.354931366 |
| cg24698924 | chr14 | 1.06E+08 | 106416851 | gene_id MER4_14q32.33c | 5.467370079 |
| cg00476270 | chr16 | 89672252 | 89684187 | gene_id MER4_16q24.3 | 0.252080314 |
| cg20374441 | chr17 | 21133190 | 21142900 | gene_id MER4_17p11.2b | 0.939628697 |
| cg27234723 | chr1 | 45305213 | 45315797 | gene_id MER4_1p34.1c | 0.251095506 |
| cg15274684 | chr21 | 46631793 | 46635087 | gene_id MER4_21q22.3b | 1.917301525 |
| cg21592975 | chr21 | 46631793 | 46635087 | gene_id MER4_21q22.3b | -2.080239544 |
| cg03283134 | chr3 | 1.13E+08 | 112514942 | gene_id MER4_3q13.2b | -2.303847872 |
| cg25296465 | chr3 | 1.96E+08 | 195720473 | gene_id MER4_3q29c | 0.006905223 |
| cg09400963 | chr7 | 32515103 | 32516266 | gene_id MER4_7p14.3c | -2.018556357 |
| cg07865653 | chr9 | 35025135 | 35028325 | gene_id MER4_9p13.3b | 9.210983067 |
| cg00812655 | chrX | 16577951 | 16583715 | gene_id MER4_Xp22.2d | 0.008355447 |
| cg06578851 | chrX | 1.02E+08 | 102067080 | gene_id MER4_Xq22.1d | 0.658914448 |
| cg09284509 | chrX | 1.02E+08 | 102067080 | gene_id MER4_Xq22.1d | 0.789550176 |
| cg03955589 | chr11 | 349106 | 352592 | gene_id MER41_11p15.5b | 6.527169204 |
| cg16218276 | chr19 | 57275342 | 57278839 | gene_id MER41_19q13.43a | 4.632929031 |
| cg22350285 | chr1 | 2.45E+08 | 244818297 | gene_id MER41_1q44a | 0.833144826 |
| cg15803346 | chr20 | 45482970 | 45489460 | gene_id MER41_20q13.12 | 0.897039802 |
| cg05209515 | chr22 | 37188838 | 37196108 | gene_id MER41_22q12.3e | 2.440538022 |
| cg26440042 | chr22 | 37188838 | 37196108 | gene_id MER41_22q12.3e | -0.39202229 |
| cg03025176 | chr4 | 7084495 | 7091661 | gene_id MER41_4p16.1a | 0.508283667 |
| cg23780067 | chr4 | 99726123 | 99734925 | gene_id MER41_4q23d | 0.16464659 |
| cg17136442 | chr5 | 1.19E+08 | 119001695 | gene_id MER41_5q23.1b | 0.237747565 |
| cg19090097 | chr6 | 79257432 | 79258797 | gene_id MER41_6q14.1a | 0.93658012 |
| cg25483734 | chr11 | 67679749 | 67683767 | gene_id MER4B_11q13.2 | 2.249142619 |
| cg03834906 | chr13 | 24640319 | 24644172 | gene_id MER4B_13q12.12 | -0.644202258 |
| cg27356159 | chr14 | 23337068 | 23345236 | gene_id MER4B_14q11.2b | 0.284184518 |
| cg21465252 | chr14 | 90238596 | 90240078 | gene_id MER4B_14q32.11a | 1.070896908 |
| cg25157473 | chr15 | 50870443 | 50875773 | gene_id MER4B_15q21.2 | 1.887429507 |
| cg16626107 | chr1 | 1275713 | 1280788 | gene_id MER4B_1p36.33 | 2.592474418 |
| cg01934596 | chr1 | 1.91E+08 | 191155164 | gene_id MER4B_1q31.2 | 0.579592871 |
| cg12273394 | chr21 | 36095300 | 36098036 | gene_id MER4B_21q22.12 | 1.663394504 |
| cg27402928 | chr3 | 1.26E+08 | 126090891 | gene_id MER4B_3q21.2 | 4.773301093 |
| cg06729434 | chr3 | 1.6E+08 | 160054533 | gene_id MER4B_3q25.33a | -4.396124044 |
| cg25292140 | chr8 | 1.43E+08 | 143114047 | gene_id MER4B_8q24.3 | -6.196428612 |
| cg12348276 | chrX | 3040211 | 3051599 | gene_id MER4B_Xp22.33 | 0.03614592 |
| cg04877503 | chr10 | 13066618 | 13072809 | gene_id MER61_10p13 | 0.026585641 |
| cg19947501 | chr13 | 1E+08 | 100409632 | gene_id MER61_13q32.3 | -0.687223854 |
| cg03720617 | chr16 | 47489299 | 47493193 | gene_id MER61_16q12.1 | 2.157643632 |
| cg15612221 | chr18 | 39795158 | 39800435 | gene_id MER61_18q12.3a | 0.204765135 |
| cg22963343 | chr1 | 45573148 | 45575575 | gene_id MER61_1p34.1a | 0.253127156 |
| cg25231957 | chr1 | 1.88E+08 | 187643633 | gene_id MER61_1q31.1a | 1.286274558 |
| cg18815065 | chr2 | 78672431 | 78678070 | gene_id MER61_2p12 | -0.699538344 |
| cg26512771 | chr2 | 1.17E+08 | 116758985 | gene_id MER61_2q14.1b | 0.424487561 |
| cg26257265 | chr3 | 81986021 | 81991673 | gene_id MER61_3p12.2 | 0.197240899 |
| cg19102621 | chr3 | 21542639 | 21548245 | gene_id MER61_3p24.3 | 0.380881739 |
| cg16230626 | chr5 | 1.25E+08 | 124967101 | gene_id MER61_5q23.2a | 0.40565317 |
| cg03808925 | chr6 | 69890795 | 69895721 | gene_id MER61_6q13b | 3.758208651 |
| cg03284554 | chr8 | 43134411 | 43139620 | gene_id MER61_8p11.21 | -1.729758041 |
| cg09225933 | chr9 | 6382449 | 6385022 | gene_id MER61_9p24.1b | 0.477056005 |
| cg15869609 | chr18 | 80060215 | 80064591 | gene_id PABLA_18q23 | -1.289730392 |
| cg08446032 | chr8 | 53348448 | 53352761 | gene_id PABLA_8q11.23 | -1.707260063 |
| cg00813582 | chr3 | 16763827 | 16772528 | gene_id PABLB_3p24.3 | 0.83493957 |
| cg06563313 | chr8 | 72096360 | 72104776 | gene_id PABLB_8q21.11 | 0.61439765 |
| cg10219742 | chr8 | 1.43E+08 | 143065669 | gene_id PABLB_8q24.3b | 1.099708356 |
| cg05025211 | chr9 | 1.36E+08 | 135662919 | gene_id PABLB_9q34.3 | -0.634308005 |
| cg22600802 | chr12 | 31307571 | 31315051 | gene_id PRIMA4_12p11.21b | -1.370191655 |
| cg00845901 | chr10 | 49371 | 52548 | gene_id PRIMA41_10p15.3 | 1.783938707 |
| cg18158970 | chr14 | 91679851 | 91683021 | gene_id PRIMA41_14q32.12 | 3.916529257 |
| cg10433390 | chr17 | 43354075 | 43360454 | gene_id PRIMA41_17q21.31b | 0.079401695 |
| cg12432496 | chr18 | 49690 | 52854 | gene_id PRIMA41_18p11.32 | 0.861644672 |
| cg15851164 | chr18 | 49690 | 52854 | gene_id PRIMA41_18p11.32 | 0.109859019 |
| cg26905010 | chr18 | 49690 | 52854 | gene_id PRIMA41_18p11.32 | 2.323507509 |
| cg26491555 | chr1 | 2.16E+08 | 216484870 | gene_id PRIMA41_1q41 | -1.221858524 |
| cg07166084 | chr1 | 2.42E+08 | 242056948 | gene_id PRIMA41_1q43 | 1.045597755 |
| cg27466466 | chr22 | 23915159 | 23919868 | gene_id PRIMA41_22q11.23 | -0.060064014 |
| cg23387678 | chr4 | 1.75E+08 | 175456817 | gene_id PRIMA41_4q34.2 | 2.434780812 |
| cg13129313 | chr7 | 93148971 | 93154061 | gene_id PRIMA41_7q21.2 | -3.095350125 |
| cg27451843 | chr7 | 93148971 | 93154061 | gene_id PRIMA41_7q21.2 | 0.694715825 |

Table S2.

**Composite Retroelement-Age-450 Clock**

| **name** | **coefficient** |
| --- | --- |
| (Intercept) | 84.2097786 |
| cg11646485 | -0.3334785 |
| cg18515940 | -1.1744302 |
| cg20985847 | 0.51285902 |
| cg02688359 | -1.8025046 |
| cg02290350 | 1.48342987 |
| cg19539224 | 9.44319893 |
| cg22619910 | 0.41802207 |
| cg10621809 | 0.94322639 |
| cg15852841 | -0.8749933 |
| cg06655097 | 3.1178577 |
| cg15181676 | 3.68612092 |
| cg23015605 | 0.98157593 |
| cg26138202 | 1.50594173 |
| cg22596411 | 1.07289093 |
| cg16905434 | 1.25823084 |
| cg06192058 | -0.4070345 |
| cg27353054 | -1.889006 |
| cg09458956 | -0.5904639 |
| cg24634009 | -10.790215 |
| cg06159896 | 3.0570234 |
| cg12707353 | -0.9014883 |
| cg07416187 | -3.393147 |
| cg00995689 | 2.17533113 |
| cg03569377 | -1.1603567 |
| cg12121166 | -6.0120714 |
| cg18455249 | -1.3523467 |
| cg19815963 | 0.7262203 |
| cg11544647 | 1.39778389 |
| cg06426293 | 0.2683674 |
| cg03042396 | -0.9093125 |
| cg03020635 | 2.93574811 |
| cg02083109 | -2.1628984 |
| cg10136168 | 13.723657 |
| cg01665013 | 0.20883755 |
| cg03734002 | -2.9763659 |
| cg21177257 | 1.05073719 |
| ch.3.82259654F | 21.0549245 |
| cg09387528 | -1.3488149 |
| cg26391077 | -0.740924 |
| cg16230384 | 0.6201422 |
| cg05012523 | -0.5157438 |
| cg22511706 | 0.54981131 |
| cg00695558 | -1.1283257 |
| cg04167804 | 1.56629744 |
| cg16659657 | -4.6351881 |
| cg08596532 | 3.73765361 |
| cg26784412 | -0.9999887 |
| cg05025211 | -0.7310711 |
| cg02499236 | 0.71323469 |
| cg07268090 | 0.43446946 |
| cg23530031 | 7.65718207 |
| cg16923485 | 0.11703562 |
| cg16435580 | 0.08900882 |
| cg22652999 | 1.34478617 |
| cg09531389 | 0.83206355 |
| cg12678274 | -0.3744261 |
| cg14074366 | 1.19560486 |
| cg22352709 | 0.29332813 |
| cg01695620 | 2.34538904 |
| cg14255471 | 0.57481056 |
| cg16888367 | 2.65487665 |
| cg04012319 | 0.72615517 |
| cg01934638 | 1.9880481 |
| cg10707794 | -1.8279952 |
| cg19728936 | 0.60470298 |
| cg21556904 | 2.42241276 |
| cg27243686 | -2.2488183 |
| cg25497175 | 0.01741391 |
| cg03955589 | 14.8794041 |
| cg19983559 | -1.0309958 |
| cg19266945 | 0.4001721 |
| cg01359967 | -1.4580083 |
| cg21315874 | 3.62037495 |
| ch.9.98463211R | -2.2374994 |
| cg11537447 | 0.10529025 |
| cg06933036 | -3.1062217 |
| cg20120786 | 0.53096139 |
| cg16670889 | 0.41852176 |
| ch.15.37208706R | -0.67646 |
| cg24282829 | -4.0944434 |
| cg20658450 | -0.4703555 |
| cg26754510 | -2.1526338 |
| cg03891574 | -3.5826037 |
| ch.12.71498574F | 0.51466414 |
| cg16175737 | 1.0172845 |
| cg20545694 | 0.91226111 |
| cg07226718 | 0.08964229 |
| cg10163998 | 21.7984595 |
| cg25422346 | 2.97005974 |
| cg21191275 | 1.87752769 |
| cg24377340 | -1.881869 |
| cg16429208 | -1.205462 |
| cg15781397 | -0.5082615 |
| cg02593098 | 11.6813193 |
| cg08354320 | 0.13374795 |
| cg02279891 | -0.3654226 |
| cg26695076 | 6.80904018 |
| cg05973840 | -1.5628959 |
| ch.4.135914028F | 18.248008 |
| cg06832262 | -1.9645159 |
| cg25095589 | -3.2608107 |
| cg04268211 | 0.00131627 |
| cg11677705 | -0.6371553 |
| cg03131527 | 4.45427775 |
| cg01406647 | 5.92677504 |
| cg13904130 | 2.64069378 |
| cg14076394 | -5.4789229 |
| cg05706649 | -0.4983313 |
| cg14675109 | 0.45713727 |
| cg11481687 | 1.35597746 |
| cg26475819 | -0.4146211 |
| cg23498494 | 8.59768222 |
| ch.5.91394379F | -10.594688 |
| cg11277190 | 2.46698194 |
| cg02505117 | 2.70528398 |
| cg07000980 | -2.6639672 |
| ch.8.89640933F | -2.6728686 |
| cg26461477 | 0.34400685 |
| cg26177807 | 0.28847152 |
| cg14992760 | 0.40593562 |
| cg21771528 | -2.9148872 |
| cg27392286 | 1.81694342 |
| cg13999210 | -0.1742094 |
| cg18467358 | -0.3055083 |
| cg06634886 | -3.6805321 |
| cg20285723 | 1.04557815 |
| cg12927785 | 1.4720719 |
| cg21733098 | -1.9609821 |
| ch.4.76675046R | 6.82595907 |
| cg15542144 | 0.91820428 |
| cg15439110 | -0.8123678 |
| cg17608350 | 1.02300423 |
| cg23658362 | 0.7140242 |
| cg14207326 | -1.0413627 |
| cg19217735 | -7.9963473 |
| cg13039545 | 2.28878771 |
| cg19622415 | -3.5789916 |
| cg10312190 | -5.0395964 |
| cg07461215 | -0.6050994 |
| cg11800577 | 0.54600549 |
| cg24937877 | -7.9836567 |
| cg20003377 | 1.19180701 |
| cg15814980 | -2.2206018 |
| cg02526508 | -1.5022928 |
| cg09032314 | 0.76334631 |
| cg18074165 | -3.0442267 |
| cg11186593 | 1.11732212 |
| cg04685392 | -1.5025977 |
| cg24344787 | 1.7809667 |
| cg20257330 | 1.99067135 |
| cg08967584 | -0.0319972 |
| cg26516287 | 0.49240565 |
| cg05417140 | -0.140524 |
| cg01115380 | 0.98724766 |
| cg07177306 | 0.05787217 |
| cg01817897 | 7.62359967 |
| cg16662846 | -0.5279175 |
| cg10627583 | 0.0857773 |
| cg23453435 | -0.7348194 |
| cg01360457 | -4.3838091 |
| cg12796916 | 1.460946 |
| cg26905010 | 4.62792045 |
| cg03089994 | 0.17936837 |
| cg02014334 | -1.1655658 |
| cg12879853 | 3.46248003 |
| cg18805182 | -0.206353 |
| cg08374659 | 0.50832097 |
| cg02181287 | -5.5105638 |
| cg18682615 | -1.9318868 |
| cg17316564 | -0.5547581 |
| cg05995607 | 0.57734469 |
| cg14264793 | 1.93687619 |
| cg10324329 | 3.86431459 |
| cg23198707 | 8.79547211 |
| cg09933401 | -0.172604 |
| cg24169503 | 0.72572864 |
| ch.6.98280058F | 12.6242957 |
| cg05003973 | 9.083922 |
| cg11969526 | 15.0524409 |
| cg19523937 | -3.0152816 |
| cg01130322 | 0.6849541 |
| cg08355345 | -0.4263691 |
| cg00599812 | -0.1105997 |
| cg22016755 | -1.7540075 |
| cg04459585 | -0.1175225 |
| cg02082613 | -0.689558 |
| cg18163973 | -2.7461143 |
| cg21064337 | 1.6449502 |
| cg20991483 | 1.31366051 |
| cg05690360 | 0.6939264 |
| cg27371125 | 0.83856063 |
| cg22136013 | -0.4383245 |
| cg14083558 | -1.272568 |
| cg26196162 | 0.36332076 |
| cg10265952 | -1.8062413 |
| cg19786602 | -3.557829 |
| cg20680308 | 4.32187251 |
| cg16717781 | 0.48494918 |
| cg18927185 | 0.10127837 |
| cg24504014 | 0.08078625 |
| cg10667394 | 2.12188039 |
| cg26096646 | -2.3243794 |
| cg26142490 | 6.35463723 |
| cg16530208 | 0.2849039 |
| cg20349435 | -0.6327068 |
| cg09429216 | 0.00261291 |
| cg19102621 | 1.09405278 |
| cg04000927 | 6.62444946 |
| cg09374673 | -1.879204 |
| cg07028263 | -1.6471411 |
| cg00368844 | -2.2277941 |
| cg20502536 | 0.35847092 |
| cg22965561 | 0.22415037 |
| cg06346218 | 0.22921446 |
| cg21158075 | -1.55543 |
| cg13619891 | 2.22324495 |
| cg21293117 | -3.2552878 |
| cg16989714 | -0.4125832 |
| cg09180890 | 0.19545298 |
| cg25062533 | -0.0371583 |
| cg14714629 | 5.86382428 |
| cg04674267 | -1.5280943 |
| cg26305090 | 0.13698141 |
| cg02960716 | 0.14305922 |
| cg00380930 | 0.59597936 |
| cg04390328 | -2.4147836 |
| cg12144446 | 2.39029919 |
| cg20061931 | 7.82956995 |
| cg21054147 | -9.1317428 |
| cg01717482 | -0.1410097 |
| cg21481321 | -9.6484701 |
| cg08496664 | 0.12468676 |
| cg17975299 | 0.37907149 |
| cg23047397 | 0.12393984 |
| cg03603296 | -0.1487328 |
| ch.10.58771679R | -1.9609257 |
| cg20024577 | 1.26358098 |
| cg10021364 | -7.6580537 |
| cg25539589 | 4.03493816 |
| cg03720617 | 2.83045456 |
| cg06177459 | 0.75867171 |
| cg14295611 | -21.938655 |
| cg19901523 | -16.552303 |
| cg26257265 | 1.56457043 |
| cg11902554 | -1.0439367 |
| cg08412584 | -1.0890081 |
| cg12500951 | -3.00476 |
| cg14944696 | -2.0834467 |
| cg19564408 | 3.06236583 |
| cg12670123 | -5.9636443 |
| cg12360690 | -2.8774635 |
| cg00436420 | 1.82880378 |
| cg11361409 | 0.78472337 |
| cg20186995 | 3.72138451 |
| cg22877639 | -0.2140026 |
| cg00007540 | -1.4439135 |
| cg00987743 | -0.9351065 |
| cg00125002 | 3.48124638 |
| cg12967666 | 0.65727375 |
| cg07633701 | 3.41861683 |
| cg18232879 | -0.8264486 |
| cg27562259 | -5.2404509 |
| cg03073003 | -0.7811652 |
| cg21646025 | 3.46503964 |
| cg15274684 | 3.73687327 |
| cg12424716 | 3.87380553 |
| cg25515758 | 0.99773714 |
| cg10815129 | -5.0062316 |
| cg18004371 | -1.301474 |
| cg21547628 | -3.1237774 |
| cg15612221 | 0.12551084 |
| cg20746081 | 0.96223242 |
| cg06604447 | -0.017952 |
| cg16757896 | 4.19747426 |
| cg10070101 | -1.1989507 |
| cg18710738 | -1.3977465 |
| cg11022525 | -0.5809109 |
| cg11848882 | -6.8311907 |
| cg02140171 | -1.3233544 |
| cg00239171 | 0.81715798 |
| ch.2.147017622F | -17.010788 |
| cg04834966 | 1.66822542 |
| cg18815398 | 0.20048329 |
| cg05676261 | 2.68120986 |
| cg26341086 | 0.67363007 |
| cg12109747 | 0.29332166 |
| cg07448812 | 1.47881277 |
| cg05697754 | -8.8716988 |
| cg01971370 | 0.11292403 |
| cg05624396 | -0.9193827 |
| cg22264170 | -0.6871033 |
| cg15277108 | -3.5593739 |
| cg21214566 | -2.3685062 |
| cg09223975 | -2.2824758 |
| cg14353201 | 0.92215053 |
| cg03834906 | -0.0512222 |
| cg09591497 | -0.2146299 |
| cg14758163 | -3.5788942 |
| cg00047553 | 0.64166437 |
| cg00218406 | 0.38875903 |
| cg05407200 | -0.0132457 |
| cg22822395 | 0.14863518 |
| cg21071075 | 2.92653293 |
| cg01720520 | 0.96724153 |
| cg19947501 | -0.7629789 |
| cg26876466 | 0.59950869 |
| cg25570453 | 1.34699623 |
| cg13983922 | -0.466737 |
| cg24377495 | 0.17636605 |
| cg25853655 | 0.55544315 |
| cg14284541 | -0.6993418 |
| cg16230626 | 2.40598274 |
| cg24706839 | 0.65466678 |
| cg25257018 | -2.4498968 |
| cg00813264 | 10.7745412 |
| cg01361049 | -0.985762 |
| cg20110284 | 5.70859233 |
| cg01029934 | 2.17228401 |
| cg00652158 | -2.6466611 |
| cg10453777 | -3.0410972 |
| cg16321540 | 0.54925908 |
| cg15268456 | -1.0519303 |
| cg06710438 | 0.43212928 |
| cg19211853 | 2.90098714 |
| cg22165480 | -3.081733 |
| cg01394167 | -0.568791 |
| cg13556934 | 0.11086545 |
| cg05479618 | 2.4270929 |
| cg14775504 | 1.95389179 |
| cg13624406 | 0.51947654 |
| cg01374735 | -0.5306206 |
| cg15234816 | -0.0239575 |
| cg20675040 | -2.440578 |
| cg13093110 | 0.45591349 |
| cg16691557 | -0.2923012 |
| cg06578851 | 2.40207956 |
| cg06794038 | 2.24410923 |
| cg05785724 | 1.39238296 |
| cg21905068 | 4.73967461 |
| cg23333970 | -3.0595738 |
| cg05308904 | 13.1181428 |
| cg11751719 | 1.09962835 |
| cg16202338 | 0.06174095 |
| cg01238639 | -0.6299759 |
| cg11001828 | -0.5302022 |
| cg16819051 | -1.1200447 |
| cg11630939 | -12.976565 |
| cg14064092 | 0.70975508 |
| cg21368154 | -2.7368707 |
| cg01848115 | 1.15623634 |
| cg11901929 | 1.6066733 |
| cg23612095 | 1.47323473 |
| cg03929825 | 3.8383448 |
| cg16996746 | -6.2961522 |
| cg03278407 | -1.3597393 |
| ch.4.658374F | -69.666392 |
| cg00824379 | 8.80324298 |
| cg07520860 | 3.18634976 |
| cg20586911 | -1.2973414 |
| cg26925025 | 0.01091578 |
| cg10487521 | 3.46387176 |
| cg18072778 | 1.92699533 |
| cg05613017 | 1.93809656 |
| cg06564069 | -2.7238696 |
| cg10669449 | -3.1003065 |
| cg00308770 | 0.32199916 |
| cg18751637 | -1.1400262 |
| cg08277815 | -0.5000431 |
| cg03410772 | -1.1743714 |
| cg06857049 | -11.449331 |
| cg03840694 | 1.49910977 |
| cg00642607 | 1.26169328 |
| cg27451843 | 7.40296583 |
| cg25843003 | -1.0975015 |
| cg26563242 | 0.87456678 |
| cg12805629 | 0.50794241 |
| cg06377220 | -1.0924368 |
| cg12417549 | 0.52207593 |
| cg02022488 | -2.1967409 |
| cg10052504 | 0.86428485 |
| cg21533683 | 0.1863555 |
| cg11523960 | 0.52735619 |
| cg25215835 | -2.7129188 |
| cg19188207 | -0.0504706 |
| cg23860269 | 0.10746552 |
| cg09520498 | -1.9787197 |
| cg04909259 | -0.5413854 |
| cg17765025 | 0.76781985 |
| cg13633941 | -0.0686142 |
| cg08960549 | -8.9944737 |
| cg14716323 | -1.8108292 |
| ch.4.146002696F | 4.85993684 |
| cg04289078 | -3.4110435 |
| cg18398175 | -13.95717 |
| cg09278098 | -19.219234 |
| cg06596503 | -1.3480508 |
| ch.6.94606978F | 1.20364213 |
| cg12212060 | -8.3266985 |
| cg24245773 | -0.3584717 |
| cg14297546 | -4.4502761 |
| cg18237203 | -0.9795621 |
| cg16245716 | -3.7804936 |
| cg19556341 | 1.3328314 |
| cg22787186 | -0.1271806 |
| cg06683148 | 2.08181535 |
| cg27203309 | -1.198425 |
| cg24140801 | -4.658053 |
| cg01432405 | -5.2174121 |
| cg08686011 | 0.7691598 |
| cg15905124 | 2.68542705 |
| cg26914004 | 0.40562817 |
| cg16876162 | -4.5060009 |
| cg13028481 | 0.51521362 |
| cg15367212 | 1.4908969 |
| cg23376071 | 0.21280001 |
| cg03284554 | -8.4007311 |
| cg06857473 | -1.2573159 |
| cg00966482 | 2.31931256 |
| cg19282411 | -0.0559959 |
| cg07334926 | -5.6454742 |
| cg02532030 | -1.1913271 |
| cg14710850 | 0.0127547 |
| cg22993358 | -0.729723 |
| cg14897569 | 1.72199899 |
| cg06551476 | 9.48400728 |
| cg02160611 | -2.1866774 |
| cg26655361 | -0.6405415 |
| cg04379642 | 0.09507814 |
| cg19353377 | 1.35934239 |
| cg09284509 | 1.65448325 |
| cg20494196 | 1.1476635 |
| cg01002223 | -10.784437 |
| cg18531351 | -0.6280921 |
| cg20781526 | -3.340435 |
| cg07950964 | 0.77415023 |
| cg23213327 | 6.38245417 |
| cg22166728 | -0.5552188 |
| cg03784956 | -0.3028397 |
| cg26647265 | 1.53190745 |
| cg20679403 | -0.4422926 |
| cg16173234 | -1.678041 |
| cg21149357 | -0.1241785 |
| cg00954167 | 0.99685449 |
| cg25142416 | -0.0780113 |
| cg16961648 | 3.28068275 |
| cg18810033 | 1.93919333 |
| cg04484136 | 1.84115081 |
| cg17715419 | 8.28881472 |
| cg02055547 | 0.91429416 |
| cg00219792 | -1.9485982 |
| cg24666178 | -2.6498688 |
| cg11881003 | 0.98021231 |
| cg03334473 | -0.349595 |
| ch.6.11766035R | -4.5681991 |
| cg19968790 | -3.4026726 |
| cg13143120 | 2.14112824 |
| cg08121925 | -1.3346356 |
| cg05209515 | 3.59614544 |
| cg24095864 | -2.8534873 |
| cg27139424 | 6.57908851 |
| cg03349184 | 14.398962 |
| cg00344422 | -0.1787625 |
| cg14109822 | -1.1890658 |
| cg20411127 | 1.62299719 |
| cg10869641 | -1.543386 |
| cg26967792 | -1.3771278 |
| cg26826927 | -5.9729654 |
| cg26538224 | -0.2748764 |
| cg11072637 | 0.71361537 |
| cg21593148 | -2.2910204 |
| cg08803790 | 3.37529216 |
| cg26369133 | -4.9618722 |
| cg04688024 | -0.6316551 |
| cg00422311 | 3.68660587 |
| cg14270590 | -1.2466711 |
| cg04827593 | 2.41066837 |
| cg17952939 | -1.7705094 |
| cg04885343 | 0.36856267 |
| cg14086828 | 4.52375428 |
| cg15988287 | 5.01208495 |
| cg14372234 | 1.45965058 |
| cg21684411 | -0.2249724 |
| cg23590472 | -0.6683603 |
| cg01455398 | -1.8000575 |
| cg21290162 | 1.15002802 |
| cg24944500 | 0.82007578 |
| cg05546741 | -5.6166979 |
| cg15983605 | 1.28149178 |
| cg00353337 | -1.7082311 |
| cg21070222 | 1.29383663 |
| cg06641336 | -3.6993983 |
| cg12828313 | 0.82706488 |
| cg22160110 | 2.22448515 |
| cg14146669 | -1.1797067 |
| cg13578306 | 0.58847506 |
| cg17874485 | -0.1202576 |
| cg14778437 | -10.033303 |
| cg08137014 | 0.80017982 |
| cg24247132 | 0.05177589 |
| cg15914485 | -0.6579395 |
| cg17217478 | 0.32595145 |
| cg10193420 | 1.39096286 |
| cg11369485 | -1.4164535 |
| cg09880847 | -0.8390707 |
| ch.9.10784434R | 3.17103985 |
| cg22423821 | -0.8689762 |
| cg00919806 | -1.6080873 |
| cg23232773 | -1.3233385 |
| cg09164913 | -10.429501 |
| cg07548668 | 0.54066407 |
| cg02137970 | 7.50500714 |
| cg08646442 | -0.7066142 |
| cg12270717 | -3.0852439 |
| cg15737123 | -1.1482506 |
| cg23961091 | 2.4692874 |
| cg15128347 | 1.17257571 |
| cg10139151 | 2.41045527 |
| cg11041994 | 1.37959812 |
| cg00621559 | 0.52756051 |
| cg08883146 | -9.7189061 |
| cg07077221 | 4.58250142 |
| cg00845901 | 4.47646885 |
| cg18340445 | 9.52482614 |
| cg24408706 | -5.6992618 |
| cg14083720 | 0.81527798 |
| cg22552237 | 1.20838061 |
| cg19791627 | -0.4583098 |
| cg02026801 | 0.08786927 |
| cg13698991 | -2.5435763 |
| cg05221326 | 3.15754664 |
| cg26266099 | -4.0907996 |
| cg14619716 | 0.57295938 |
| cg02936931 | 7.32600583 |
| cg09191776 | -2.7802843 |
| cg25102653 | 1.00750048 |
| cg09828411 | 0.66960174 |
| cg03520254 | -1.0198925 |
| cg23363602 | 0.92602315 |
| cg06707139 | -2.2643522 |
| cg17410603 | -4.928856 |
| ch.4.133661909R | 1.6219399 |
| cg26562263 | -0.8310461 |
| cg10256304 | -0.4287672 |
| cg12183549 | 1.27413448 |
| cg06014506 | -3.4320629 |
| cg11854777 | -1.405656 |
| cg08415137 | 0.53080496 |
| cg07803836 | -1.5792427 |
| cg15083851 | -5.142878 |
| cg20684174 | 1.91610631 |
| cg15988983 | -2.414564 |
| cg13643180 | -2.6975891 |
| cg18808777 | 2.77613575 |
| cg05621759 | 1.9517815 |
| cg12892666 | 0.05450122 |
| cg12949927 | 0.0037053 |
| cg08541930 | 7.94958082 |
| cg25601713 | 0.08639137 |
| cg00418219 | 1.07708044 |
| cg24004238 | -0.068475 |
| cg23987336 | 3.88536533 |
| cg12502751 | -0.4254008 |
| cg09342225 | -9.7767124 |
| cg12338731 | 6.65764811 |
| cg25035200 | -1.871501 |
| cg17429887 | 2.01744448 |
| cg00224417 | 0.19285811 |
| cg03631065 | -7.426292 |
| cg14109551 | -2.9570847 |
| cg10503542 | 0.02258147 |
| cg23917817 | 1.45022654 |
| cg07355088 | 0.29055954 |
| cg02358456 | -1.3397547 |
| cg25727029 | 2.19917269 |
| cg24073023 | 0.02861416 |
| cg00813582 | 1.72014321 |
| cg17588096 | -0.5860517 |
| cg04838191 | 0.75602561 |
| cg08550748 | 0.26519104 |
| cg09787236 | 0.16414286 |
| cg23236169 | -10.255783 |
| cg22106265 | 4.54402436 |
| cg20510833 | -0.0480361 |
| cg13743052 | -21.08033 |
| cg03189479 | 3.85198627 |
| cg18186914 | 1.02487374 |
| cg10687139 | -0.2970918 |
| cg24764114 | -0.7335644 |
| cg22375663 | -3.7959825 |
| cg02423471 | -0.0263675 |
| cg11270998 | -0.6607302 |
| cg08597142 | 0.60885632 |
| cg04326117 | -2.9684589 |
| cg03739470 | 1.46679121 |
| cg03805769 | -0.3592915 |
| cg02431089 | 4.74572021 |
| cg22371963 | -0.7100172 |
| cg10103906 | 0.01521436 |
| cg26542660 | -1.4942983 |
| cg08475244 | 2.33573625 |
| cg09322278 | 2.63753652 |
| cg03982016 | 1.98994378 |
| cg13526915 | 1.27160898 |
| cg17989736 | -1.1353939 |
| cg06152845 | -0.5932492 |
| cg07493704 | 1.4285577 |
| cg24141724 | 1.31650824 |
| cg22799691 | 1.09913658 |
| ch.12.602695F | -27.320233 |
| cg00913288 | 0.71228402 |
| cg17326994 | -0.4033799 |
| ch.8.869729R | -3.6284234 |
| cg19508985 | -0.493607 |
| cg06834035 | 1.82102828 |
| cg10619129 | 1.48129807 |
| cg13836687 | 0.63348029 |
| cg09476073 | 9.22805416 |
| cg11802781 | 0.71507156 |
| cg05003175 | 0.79717993 |
| cg26583584 | 3.86848814 |
| cg14709381 | 1.91719447 |
| ch.8.72742858R | -0.0294869 |
| cg03978169 | -5.7357103 |
| cg00508950 | -3.2073322 |
| cg01397223 | 0.9598013 |
| cg16818145 | -0.1425221 |
| cg18181787 | -0.6104862 |
| cg07096305 | 2.12251144 |
| cg09453685 | -3.7830328 |
| cg08321815 | 0.68977488 |
| cg07757454 | -0.9177274 |
| cg12221729 | 0.52872416 |
| cg02345638 | -1.6706123 |
| cg02020528 | 1.3575668 |
| cg02531193 | 2.62868088 |
| cg23153661 | -4.3884003 |
| cg14178937 | -0.5707914 |
| cg05643494 | -0.1189499 |
| cg02730303 | 0.09491627 |
| cg00812839 | -0.2320904 |
| cg12475092 | 0.13756624 |
| cg03290530 | 0.46971211 |
| cg19521610 | -0.5738869 |
| cg26526883 | 1.28187458 |
| cg14631761 | 0.14764823 |
| cg18030084 | 1.8817896 |
| cg09459548 | 4.85354703 |
| cg25265769 | 2.07861369 |
| cg07277465 | 0.77853398 |
| cg02604095 | 0.98940163 |
| cg11827743 | 1.08977974 |
| cg14416248 | 6.58745494 |
| cg07855322 | 1.62473651 |
| cg27492220 | 0.74234057 |
| cg05891292 | -0.7072539 |
| cg26352073 | 0.43684115 |
| cg09542154 | -7.6312475 |
| cg24980995 | 1.40580712 |
| cg26592560 | -0.188319 |
| cg08599672 | 0.24062445 |
| cg14492061 | -4.35854 |
| cg06705361 | -2.4418945 |
| cg17035109 | 2.97384713 |
| cg12882999 | -1.9871646 |
| cg14580297 | 2.19616017 |
| cg25456633 | -2.0530933 |
| cg15632673 | 1.09427949 |
| cg04461867 | -0.9690265 |
| cg12006733 | -16.398753 |
| cg20976526 | 1.22016309 |
| cg24848035 | 2.13732225 |
| cg16721359 | 12.3723443 |
| cg09526343 | -4.2282072 |
| cg02703695 | -1.3745127 |
| cg12248480 | -0.8664611 |
| cg12022630 | -0.6263627 |
| cg13488137 | 0.03583193 |
| cg02915530 | 0.92013436 |
| cg26834680 | 5.67136986 |
| cg09597666 | 0.40913702 |
| cg14126093 | 3.05626708 |
| cg01154525 | -0.172521 |
| cg03646329 | 6.65983532 |
| cg00186462 | 0.08458167 |
| cg12103802 | 1.07627483 |
| cg18569825 | -1.4339459 |
| cg02471166 | -1.087529 |
| cg04799493 | -2.7836722 |
| cg16535779 | -0.1985967 |
| cg15015426 | -0.4958712 |
| cg13947513 | -9.6551872 |
| cg15847775 | -1.7562905 |
| cg06659867 | 1.50857306 |
| cg14195992 | -0.004583 |
| cg22624022 | 12.6208981 |
| cg27073142 | 5.66356878 |
| cg00264298 | -0.3387527 |
| cg02262167 | -0.0894292 |
| cg01982516 | 2.94215387 |
| cg21206021 | -3.324276 |
| cg11147878 | -5.109311 |
| cg21909533 | 0.16294172 |
| cg26763368 | -0.2145293 |
| cg22833428 | -0.1575886 |
| cg17063780 | -0.0868106 |
| cg24048338 | 2.34096967 |
| cg20529334 | -0.8469838 |
| cg05464291 | 1.50357684 |
| cg11497131 | 4.32459884 |
| cg23180171 | -0.2585689 |
| cg15555527 | 5.65077927 |
| cg00271873 | -0.0119224 |
| cg03284839 | -0.8307769 |
| cg02629586 | 0.97463611 |
| cg26495473 | 1.51764965 |
| cg24674269 | 3.70225114 |
| cg07563409 | -0.9549158 |
| cg25756647 | 0.33169089 |
| cg13731106 | 8.93672531 |
| cg13913108 | 0.2981733 |
| cg27574287 | 0.29496878 |
| cg05943574 | 11.2719283 |
| cg24640561 | 0.72280227 |
| cg25120459 | -2.8224113 |
| cg05608794 | -0.9917197 |
| cg22983641 | 1.14321832 |
| cg05256276 | -0.690119 |
| cg26329047 | 1.46668053 |
| cg19432688 | 0.15660164 |
| cg26612736 | 0.09332657 |
| cg18254183 | -3.7609542 |
| cg10386292 | 0.30334435 |
| cg16601146 | 0.02950325 |
| cg05128056 | -0.0296113 |
| cg14741811 | -0.9992544 |
| cg08184936 | 1.53384072 |
| cg11809897 | -0.5622839 |
| cg07743730 | -0.9933343 |
| cg25991569 | 1.976221 |
| cg03779499 | -0.3514851 |
| cg04189295 | 0.29499246 |
| cg14260377 | -0.2832101 |
| cg23192749 | 1.36884824 |
| cg03346692 | -1.375336 |
| cg17168157 | -2.350856 |
| cg13011976 | 1.37497564 |
| cg23726945 | -4.6085758 |
| cg25405719 | 0.44742851 |
| cg08212545 | -6.0299942 |
| cg14055195 | -3.4607139 |
| cg27111461 | 3.29002584 |
| cg24699985 | 0.08951318 |
| cg06569139 | 4.29303671 |
| cg20492230 | -0.0330861 |
| cg00318766 | 1.02383274 |
| cg00010242 | -0.1057661 |
| cg19071460 | -1.1190304 |
| cg16134501 | 1.12432008 |
| cg00954536 | -2.461849 |
| cg03020006 | -2.2650294 |
| cg12668649 | -0.8437792 |
| cg03562350 | -0.6224106 |
| cg06001854 | -2.1007551 |
| cg04675342 | -1.8728802 |
| cg03671431 | 4.42588299 |
| cg23228779 | -0.5203797 |
| cg27528032 | -12.706267 |
| cg04411541 | 10.5411472 |
| cg07348854 | -1.2408709 |
| cg20364222 | 4.5957496 |
| cg13781819 | 4.88145036 |
| cg21599794 | -0.1119718 |
| cg01267908 | 1.10702468 |
| cg18009322 | -0.6858552 |
| cg05398036 | 0.05305321 |
| cg20860642 | 0.12796506 |
| cg00861295 | -8.3898538 |
| cg03838823 | -3.0178721 |
| cg09041389 | -0.9960301 |
| cg20064008 | 3.22190619 |
| cg27069616 | 2.91032575 |
| cg14985830 | -5.5035902 |
| cg18205081 | 9.40526688 |
| cg08200691 | 0.60329028 |
| cg14345215 | 0.53865976 |
| cg01187600 | 7.40171532 |
| cg04061225 | 0.81113003 |
| cg09690561 | -2.3116132 |
| cg07298638 | 4.95656626 |
| cg15611936 | -0.2694882 |
| cg08284207 | 0.07898299 |
| cg08772684 | -3.5175293 |
| cg13292487 | 0.51324001 |
| cg04548815 | 15.9883443 |
| ch.2.154349706F | 13.0656966 |
| cg04392293 | -0.6594413 |
| cg10561983 | 3.03462715 |
| cg27198040 | 0.46423833 |
| cg18920490 | -0.6163893 |
| cg13666005 | -0.5446042 |
| cg16966139 | -0.8625472 |
| cg05051799 | 1.88184376 |
| cg15553710 | 0.2205259 |
| cg21481322 | 1.55176962 |
| cg03006531 | 2.595871 |
| cg07538406 | 7.83308568 |
| cg16397176 | -1.0100189 |
| cg02645058 | -0.9631048 |
| cg08186548 | -4.3425906 |
| cg11507895 | -0.3673688 |
| cg16497431 | 1.79609348 |
| cg17063731 | -0.2075538 |
| cg24170212 | 0.28644242 |
| cg15293759 | 2.38646916 |
| cg11900328 | 0.86201868 |
| cg26064634 | 3.68746862 |
| cg06842946 | -1.1631653 |
| cg11296715 | 1.05995985 |
| cg03026420 | -0.6904202 |
| cg08169950 | -0.7742996 |
| cg19183395 | -0.1983226 |
| cg26103331 | 0.77762629 |
| cg09690215 | -0.60632 |
| cg06073147 | 0.02078478 |
| cg00745855 | 0.2190912 |
| cg18167921 | -4.8686628 |
| cg13127591 | -3.8956297 |
| cg04879818 | 5.15290863 |
| cg26063904 | -6.1374355 |
| cg01080483 | 0.40194869 |
| cg17826428 | 1.05565253 |
| cg08170119 | -3.8334993 |
| cg01366941 | -0.6102709 |
| cg00834234 | 0.5045076 |
| cg20068510 | -2.3468793 |
| cg00983697 | 2.08621111 |
| cg01262133 | 5.0665794 |
| cg24300962 | -1.972678 |
| cg26872780 | 0.2628797 |
| cg14420000 | -8.7466231 |
| cg18912530 | -1.3145978 |
| cg18635896 | -14.72236 |
| cg09894096 | -2.809679 |
| cg22127299 | -1.515898 |
| cg01389383 | -0.3889855 |
| cg11028622 | 1.96017134 |
| cg20503652 | -0.1687002 |
| cg09538587 | -2.3483007 |
| cg25540004 | 0.41829823 |
| cg08694858 | -1.7771339 |
| cg17094718 | 4.07215626 |
| cg23956317 | -1.5884153 |
| ch.4.109582151R | -17.624046 |
| cg21509889 | -0.0863915 |
| cg08479629 | 4.74776191 |
| cg00995220 | 1.9026474 |
| cg20219381 | 36.2192977 |
| cg26881591 | 0.52288391 |
| cg02656525 | -10.044742 |
| cg18874294 | 1.26830222 |
| cg13420422 | -0.2835511 |
| cg16778451 | -0.3762363 |
| cg06186228 | 0.89248755 |
| cg04400072 | -2.6787849 |
| cg11178030 | -1.573209 |
| cg10300464 | -0.0778358 |
| cg14143323 | 3.57511373 |
| cg04859241 | 0.21587293 |
| cg26106149 | 1.50458336 |
| cg03878722 | -2.5614788 |
| cg04262505 | -5.0448955 |
| cg00589791 | 1.14118824 |
| cg20331613 | -0.3555238 |
| cg01473507 | 0.38018424 |
| cg20952076 | -8.2084129 |
| cg01021682 | -0.5701287 |
| cg09915950 | -0.9654028 |
| cg19304273 | -0.069367 |
| cg13342370 | 0.14156808 |
| cg00394261 | 1.38908801 |
| cg08645879 | 0.09023592 |
| cg14319700 | 0.43702972 |
| cg14115325 | -1.5909457 |
| cg10486655 | -2.5791487 |
| cg16450683 | 0.66221249 |
| cg07989373 | 2.8458493 |
| cg21358429 | -0.3102854 |
| cg20602628 | -0.6145933 |
| cg10428982 | -0.6611826 |
| cg20165732 | -3.6509035 |
| cg04561094 | 1.94251992 |
| cg04818594 | -0.5020039 |
| cg26899591 | -5.6865929 |
| cg04227141 | -4.6916354 |
| cg11359737 | 0.19477837 |
| cg00346523 | -2.1242321 |
| cg22806076 | -1.5918148 |
| cg10011062 | 1.25438485 |
| cg07455865 | -2.1184978 |
| cg03699663 | 3.75550182 |
| cg07357445 | -1.3928318 |
| cg23172545 | -3.3933301 |
| cg25169733 | 0.60865865 |
| cg14997702 | -1.5665028 |
| cg20603162 | -5.4149052 |
| cg13642800 | 2.2959598 |
| cg06535186 | 2.37548875 |
| cg12361987 | -8.5311205 |
| cg08837192 | 0.83524475 |
| cg16892443 | -12.835534 |
| cg10477471 | 0.02001943 |
| cg02993013 | 0.44585913 |
| cg09220563 | -0.4526437 |
| cg26529044 | -8.3866754 |
| cg25555059 | 3.6424407 |
| cg00459119 | 14.0413665 |
| cg06679649 | -2.6514998 |
| cg24348786 | 0.46818504 |
| cg20051292 | 1.81929314 |
| cg11131508 | -0.3706874 |
| cg15461699 | -1.2863705 |
| cg05331856 | -5.5929654 |
| cg18562198 | 0.74339517 |
| cg02041470 | -7.4826021 |
| cg17756860 | 1.83498693 |
| cg10417128 | 4.27554324 |
| cg01380321 | 0.22113336 |
| cg05339515 | 4.36953739 |
| cg05227989 | -0.7468688 |
| cg10369444 | -1.1984558 |
| cg15708382 | -0.2672843 |
| cg24739596 | 9.09988453 |
| cg18737971 | -0.3007265 |
| cg21078247 | -0.3136429 |
| cg00044501 | -1.7823931 |
| cg16635314 | 0.38527918 |
| cg00597555 | -11.423257 |
| cg13182522 | -4.2301168 |
| cg02067232 | 2.13714119 |
| ch.4.122145192R | -1.4434739 |
| cg13391607 | -0.748731 |
| cg14296235 | -0.784824 |
| cg04165188 | 2.09059031 |
| cg26413683 | 4.05806748 |
| cg01101472 | -1.4757159 |
| cg10791343 | -1.2067228 |
| cg18450054 | -0.6254158 |
| cg14569837 | 2.41678145 |
| cg24791562 | -0.4240556 |
| cg08975197 | 6.04650679 |
| cg09354692 | 0.5071646 |
| cg13574050 | -1.1046171 |
| cg07139533 | 1.00827162 |
| cg15491131 | -1.6216696 |
| cg09590778 | -2.1659849 |
| cg21871952 | -0.3635109 |
| cg14609721 | 0.99529997 |
| cg01498090 | 3.53495754 |
| cg14043702 | -0.2111933 |
| cg10131227 | -0.1771242 |
| cg25745201 | -0.2065428 |
| cg09226290 | 1.02585902 |
| cg24874998 | 3.42235334 |
| cg00038020 | -0.7327178 |
| cg07756562 | -0.521847 |
| cg23385847 | -0.270007 |
| cg10631544 | -6.1650621 |
| cg14857615 | -1.9095404 |
| cg23308414 | 0.48059237 |
| cg12160324 | 0.84181763 |
| cg09691862 | 0.06183717 |
| cg25148632 | 15.0388006 |
| cg04178131 | 0.46416029 |
| cg18494563 | 2.42029772 |
| cg21531775 | 0.57936401 |
| cg07974890 | -2.749686 |
| cg09292354 | -20.015814 |
| cg05484606 | -1.6630976 |
| cg06340382 | 0.74496724 |
| cg04831589 | 0.70427781 |
| cg21201272 | 2.88772332 |
| cg02132460 | -0.0716923 |
| cg24442760 | 1.76656689 |
| cg21399320 | 1.17610871 |
| cg17489282 | -1.2400864 |
| cg13182625 | 0.01743665 |
| cg13475915 | -0.5687867 |
| cg05207048 | -25.109329 |
| cg12049322 | 0.48374506 |
| cg00359421 | 11.3299254 |
| cg26821433 | -2.6519735 |
| cg06276653 | 3.33030858 |
| cg04361749 | 1.72528734 |
| cg16436164 | -1.5483001 |
| cg14346202 | -0.0297651 |
| cg18101152 | 14.3820706 |
| cg00573140 | 1.39495332 |
| cg04826176 | 2.29803617 |
| cg01990695 | 0.13290687 |
| cg19275309 | -0.1629162 |
| cg02841155 | -5.707851 |
| cg10932815 | -0.4472372 |
| cg07149179 | 1.70099419 |
| cg15097640 | 3.16846897 |
| cg25436634 | -0.3015639 |
| cg16543943 | 0.24269018 |
| cg13542042 | 1.46158486 |
| cg08590069 | -0.2105052 |
| cg05341115 | -0.6692054 |
| cg01820962 | -21.363478 |
| cg07457820 | 0.51266054 |
| cg07108581 | 1.98308938 |
| cg18526870 | 4.82258111 |
| cg25827670 | 1.87524711 |
| cg15448990 | -3.7774216 |
| cg13442045 | 5.13089198 |
| cg12809883 | -0.8213997 |
| cg16545749 | -2.7061823 |
| cg13570284 | 2.35399113 |
| cg01334229 | -1.4722925 |
| cg26043322 | -4.1575852 |
| cg06424894 | -3.8983031 |
| cg17316073 | -0.1214476 |
| cg07409200 | -1.4364627 |
| cg27120543 | -0.7137391 |
| cg03796122 | -0.1205412 |
| cg20705236 | -0.7128708 |
| cg03446062 | 0.36006194 |
| cg12916580 | -1.9535856 |
| ch.8.54126760R | -0.4621634 |
| cg08128444 | 0.00755844 |
| cg25197698 | 1.22103279 |
| cg22495322 | -0.7091518 |
| cg19634693 | 7.25270464 |
| cg07599972 | -1.5409889 |
| cg25013443 | 0.35105127 |
| cg00160902 | 0.49901807 |
| cg12514654 | -1.0016946 |
| cg05987172 | 1.02795992 |
| cg02762063 | 0.37814403 |
| cg16418183 | 0.59035211 |
| cg19212453 | -0.514091 |
| cg20054420 | -1.807771 |
| cg11247129 | 2.89320089 |
| cg14053595 | 2.23014675 |
| cg02407762 | 0.79503346 |
| cg16376484 | -1.0175941 |
| cg01049884 | 0.41067096 |
| cg02249233 | 3.77178618 |
| cg20413948 | -1.1593632 |
| cg27317524 | 0.99189867 |
| cg07902406 | -0.486385 |
| cg07773337 | -2.8318232 |
| cg06021910 | -4.6453787 |
| cg20751057 | 0.09056548 |
| cg15758480 | -0.9659967 |
| cg08474403 | 3.680383 |
| cg12283695 | -0.0309338 |
| cg24815638 | -5.5692528 |
| cg13089369 | -0.0146248 |
| cg25854178 | 1.73416922 |
| cg07483007 | 0.41030695 |
| cg09852918 | 2.18085446 |
| cg15929241 | -1.7819983 |
| cg21797973 | -2.8784847 |
| cg00388838 | 3.49457459 |
| cg05660197 | 0.77991511 |
| cg00355514 | -1.6447904 |
| cg02125400 | 3.89407352 |
| cg21028981 | 0.08847107 |
| ch.1.56581040R | -16.321034 |
| cg01802485 | 1.09030845 |
| cg25605360 | -2.7339347 |
| cg05174766 | 1.49962857 |
| cg13754621 | 1.45588603 |
| cg01331064 | -2.1229118 |
| cg04026763 | 0.95859608 |
| cg20395596 | 3.93997038 |
| cg16320788 | -0.061552 |
| cg20170989 | -13.430218 |
| cg00071575 | -4.8815497 |
| cg15990679 | -3.3258379 |
| cg21618529 | -1.0336967 |
| cg17092860 | 6.91535482 |
| cg15856829 | 0.0769859 |
| cg14051288 | -0.7674772 |
| cg24501758 | 1.85052362 |
| cg22585954 | -0.5148677 |
| cg13709487 | -17.97326 |
| cg25193867 | 5.70984602 |
| cg24425968 | 0.15450707 |
| cg01751961 | 1.31946798 |
| cg20255560 | -1.4732432 |
| cg11380503 | -2.0827561 |
| cg06064549 | -0.9493145 |
| cg26338947 | -0.4097106 |
| cg21192698 | 6.23745734 |
| cg22528076 | 4.19448323 |
| cg12845900 | 0.42125523 |
| cg23208513 | 2.59348781 |
| ch.5.30573736R | 0.40098326 |
| cg00460793 | -4.9675644 |
| cg27195421 | 2.51551065 |
| cg05399692 | -1.4471241 |
| cg16356630 | -0.0704587 |
| ch.10.1020819F | 1.07055103 |
| cg06843147 | -2.9339786 |
| cg26519930 | -0.9982946 |
| cg13848981 | -2.2326647 |
| cg01558040 | 4.0056795 |
| cg20465830 | 0.10691356 |
| cg04896534 | 0.83057423 |
| cg06867863 | -2.4983385 |
| cg02737458 | -2.9692059 |
| cg01950192 | 0.5764872 |
| cg18755531 | -0.5394447 |
| cg12081267 | -0.4650383 |
| cg27400353 | 1.11049692 |
| cg23948391 | 0.90632042 |
| cg03288168 | -1.8082461 |
| cg18560915 | -1.8760764 |
| cg02873885 | 0.71203344 |
| cg00132141 | -9.4781823 |
| cg18082362 | -0.3277785 |
| cg19252565 | 0.88360585 |
| cg02396891 | -3.2487658 |
| cg00900226 | 0.86569257 |
| cg09908001 | -4.50221 |
| cg03738936 | 2.34565875 |
| cg24980856 | 2.72020819 |
| cg21493768 | -1.6427144 |
| cg07421806 | -7.9588137 |
| cg13986884 | -0.2955094 |
| cg01112236 | 7.81918255 |
| cg01523029 | 0.02975875 |
| cg02143743 | 0.2227753 |
| cg12868015 | -0.1440582 |
| cg23965352 | -7.8310409 |
| cg20522598 | 4.55716605 |
| cg18617669 | -4.7529495 |
| cg16289848 | 1.81388955 |
| cg03667040 | -2.0598018 |
| cg01277177 | 1.07050274 |
| cg26257969 | -1.3177373 |
| cg19111238 | 0.39663841 |
| cg18456512 | -2.0854033 |
| cg05539841 | 0.58147741 |
| cg25015735 | -0.0767281 |
| cg10514770 | -2.9637656 |
| cg11789321 | 13.9306048 |
| cg18294158 | -2.2952936 |
| cg04141648 | -8.4570633 |
| cg04608190 | -0.5501828 |
| cg04587161 | -0.1194299 |
| cg00771553 | -1.301881 |
| cg13754569 | 0.21882202 |
| cg23343273 | 2.1692046 |
| cg15175091 | 2.1549371 |
| cg00301256 | -0.8385422 |
| cg06713076 | 1.5860693 |
| ch.9.84051654F | -6.5598356 |
| cg00498434 | 0.33387668 |
| cg10625686 | 0.29346968 |
| cg24407260 | -0.4110531 |
| cg26131114 | 1.0532063 |
| cg18770231 | 1.5881229 |
| cg15659599 | -0.8574019 |
| cg13412213 | 0.59583053 |
| cg22008851 | 16.3555587 |
| cg03526902 | 1.0551965 |
| cg00120286 | -3.4259592 |
| cg25510115 | -0.2595531 |
| cg13715827 | 0.58498152 |
| cg11807188 | 4.20285397 |
| cg25668878 | -0.4760929 |
| cg14283082 | -0.9997784 |
| cg07086224 | -1.5126912 |
| cg01579172 | 1.54709702 |
| cg24012683 | 2.26477778 |
| cg17504989 | 1.04818508 |
| cg09621031 | 0.85441395 |
| cg22862357 | 0.08021279 |
| cg22117193 | 0.62426001 |
| cg17571992 | -17.153283 |
| cg23921545 | -0.6925987 |
| ch.8.61159655R | -0.767557 |
| cg17283207 | 2.39191336 |
| cg00200074 | 0.69423184 |
| cg26450106 | 1.01517337 |
| cg08980382 | 0.61590736 |
| cg11498726 | 0.30294371 |
| cg16443014 | 0.08636214 |
| cg09272405 | -0.6277741 |
| cg14840439 | -0.1173508 |
| cg02791145 | 1.62731807 |
| cg24166814 | 2.11094483 |
| cg23822407 | 1.32555976 |
| cg02705792 | 1.88263817 |
| cg21767461 | 1.24816574 |
| cg23607309 | 0.34410307 |
| cg18860329 | -0.7178527 |
| cg02991474 | -3.93638 |
| cg10989634 | 9.30629497 |
| cg16639172 | -0.8950033 |
| cg15074709 | -0.4874268 |
| cg14206056 | -0.3377612 |
| ch.9.1518492F | -2.5973136 |
| cg05550339 | 1.63289351 |
| cg07955754 | -0.7124679 |
| cg08650132 | -0.7626134 |
| cg24387129 | 7.03443235 |
| cg26072561 | -0.2406227 |
| cg07872300 | -1.8244149 |
| cg15257565 | 2.95778584 |
| cg15016701 | -2.5605406 |
| cg03474461 | 2.70093787 |
| ch.4.1528651F | -0.6192816 |
| cg20889904 | -1.3234912 |
| cg20297873 | -3.0157776 |
| cg16710348 | 0.03804336 |
| cg00745543 | -0.7550259 |
| cg18720803 | 1.6584781 |
| cg26464882 | 2.54909341 |
| cg09552983 | 0.45314861 |
| cg08158805 | -0.6584524 |
| cg11491436 | -0.572198 |
| cg01065905 | 0.78714519 |
| cg12040750 | -0.6979536 |
| cg11239175 | 0.28296636 |
| cg25039837 | -1.1138069 |
| cg10557827 | -0.5213509 |
| cg00264634 | -0.8362145 |
| cg19744943 | -2.0007332 |
| cg04297660 | -0.4581396 |
| cg27347579 | -2.224472 |
| cg22448304 | 0.34747088 |
| cg03517476 | -5.9835501 |
| cg09699225 | -2.8199835 |
| cg24444810 | -3.8255205 |
| cg22403906 | 0.61343609 |
| cg00090261 | 9.62587244 |
| cg04988869 | 1.39515921 |
| cg00566297 | 0.11726787 |
| cg08982952 | 0.62601499 |
| cg17857642 | 0.63208268 |
| cg09694300 | -1.3701127 |
| cg25720625 | -0.3881702 |
| cg20386784 | -3.0301943 |
| cg10814736 | -8.1945056 |
| cg17216266 | 1.9442849 |
| cg19962424 | 3.6384011 |
| cg14390884 | 4.47395095 |
| cg01966974 | 5.71610377 |
| cg21244845 | -4.9849587 |
| cg13995453 | 1.15206214 |
| cg27421810 | 0.04917389 |
| cg19731276 | -0.5281757 |
| cg08327428 | -1.8742089 |
| cg09189601 | -0.0008697 |
| cg14449317 | -0.327793 |
| cg15007326 | 3.40048367 |
| cg12341018 | 0.32193985 |
| cg08707771 | 0.91477376 |
| cg18811621 | 1.42155208 |
| cg10596609 | -3.9983786 |
| cg08413469 | 15.5368293 |
| cg22106273 | -1.4040799 |
| ch.11.2015773F | 3.17883209 |
| cg23153481 | 3.4833987 |
| ch.13.654879R | -31.501671 |
| cg16598810 | 0.34208641 |
| cg12546793 | -4.1702804 |
| cg00241482 | 2.12414064 |
| ch.11.96117892F | 0.49742567 |
| cg23663942 | -0.0376164 |
| cg20087776 | 1.85161639 |
| cg13469595 | 2.72756046 |
| cg11848496 | 3.76911709 |
| cg14777702 | 1.03714503 |
| cg25032722 | -0.3388792 |
| cg27508055 | 6.58057051 |
| cg18061197 | 0.74997009 |
| cg21177713 | 3.24747085 |
| cg22154449 | 2.97947137 |
| cg00423291 | 0.69036139 |
| cg26053217 | 0.00095413 |
| cg22719314 | -1.6559569 |
| cg02401554 | 1.00143498 |
| cg14614296 | -0.9577182 |
| cg04297406 | 0.96865366 |
| cg14125530 | -11.325007 |
| ch.11.102874269F | -9.9119111 |
| cg20654619 | -3.7199667 |
| cg15533646 | 1.2935933 |
| cg13225272 | 1.91472719 |
| cg18489244 | -14.975081 |
| cg24652379 | 4.31805866 |
| cg19431244 | 3.24292998 |
| cg02098380 | 0.35768627 |
| cg06781853 | 2.01485823 |
| cg25876227 | -2.9582591 |
| cg24704151 | -1.8287316 |
| cg06301178 | 3.83960591 |
| cg19766763 | 5.78508739 |
| cg07274922 | -2.1100023 |
| cg23384017 | 2.11369671 |
| cg24286765 | 1.18420168 |
| cg02775404 | 0.87757442 |
| cg01307684 | -1.411156 |
| cg07745061 | -2.1245555 |
| cg07166084 | 2.71102131 |
| cg03376340 | 1.1442432 |
| cg17843665 | -3.5758326 |
| cg09884419 | 3.36605619 |
| cg19350224 | 0.66974978 |
| cg15442567 | 0.62174401 |
| cg14291693 | 2.53427319 |

Table S3.

**Composite Retroelement-Age V2 CpGs**

|  | **Name EPIC v1.0** | **Name EPIC v2.0** | **coefficient** |
| --- | --- | --- | --- |
| 1 | (Intercept) | (Intercept) | 62.0741981 |
| 2 | cg23728960 | cg23728960_TC21 | 0.14868104 |
| 3 | cg10366786 | cg10366786_BC11 | 0.43190631 |
| 4 | cg08398371 | cg08398371_TC21 | -3.2955737 |
| 5 | cg10973967 | cg10973967_TC21 | 2.74046222 |
| 6 | cg01721313 | cg01721313_TC21 | 0.82767473 |
| 7 | cg25231957 | cg25231957_BC21 | 2.51616862 |
| 8 | cg21992844 | cg21992844_BC21 | -1.4428095 |
| 9 | cg24646803 | cg24646803_BC21 | 0.74835065 |
| 10 | cg16037947 | cg16037947_BC21 | 0.18299151 |
| 11 | cg03468827 | cg03468827_BC21 | -0.69271 |
| 12 | cg18939097 | cg18939097_BC21 | 0.17969781 |
| 13 | cg01740695 | cg01740695_TC21 | -0.1044002 |
| 14 | cg25593520 | cg25593520_TC21 | -1.5772449 |
| 15 | cg11120551 | cg11120551_TC21 | -0.6229541 |
| 16 | cg01403320 | cg01403320_TC21 | 0.69047581 |
| 17 | cg07821808 | cg07821808_BC21 | 0.16339101 |
| 18 | cg19796584 | cg19796584_BC21 | 0.91917717 |
| 19 | cg27422507 | cg27422507_BC21 | 3.95791258 |
| 20 | cg16033766 | cg16033766_BC21 | 0.08636161 |
| 21 | cg03667422 | cg03667422_TC21 | 0.54123832 |
| 22 | cg18369325 | cg18369325_BC21 | 0.4545162 |
| 23 | cg09373192 | cg09373192_TC21 | 0.62374309 |
| 24 | cg00700455 | cg00700455_BC21 | 1.28431364 |
| 25 | cg20923885 | cg20923885_TC21 | 0.90838956 |
| 26 | cg07644039 | cg07644039_BC21 | -0.1291372 |
| 27 | cg08506585 | cg08506585_TC21 | -0.0337911 |
| 28 | cg12744300 | cg12744300_BC21 | 2.2048282 |
| 29 | cg20051117 | cg20051117_TC21 | 3.12638961 |
| 30 | cg04765439 | cg04765439_TC21 | -4.1670885 |
| 31 | cg03320087 | cg03320087_TC21 | 2.50305262 |
| 32 | cg20718469 | cg20718469_TC21 | -1.4822623 |
| 33 | cg00254470 | cg00254470_TC21 | -0.396523 |
| 34 | cg13776906 | cg13776906_TC21 | -0.8028991 |
| 35 | cg01978064 | cg01978064_BC21 | 0.39067131 |
| 36 | cg01594316 | cg01594316_BC21 | 3.90346891 |
| 37 | cg13774635 | cg13774635_BC21 | 1.4548149 |
| 38 | cg15339994 | cg15339994_BC11 | -1.9998012 |
| 39 | cg00485494 | cg00485494_BC21 | -2.0077433 |
| 40 | cg14526664 | cg14526664_TC21 | 1.62656221 |
| 41 | cg19322602 | cg19322602_BC21 | -0.3193698 |
| 42 | cg11139645 | cg11139645_TC21 | -0.4115234 |
| 43 | cg06566477 | cg06566477_BC21 | 0.12802569 |
| 44 | cg26502666 | cg26502666_BC11 | 0.12717186 |
| 45 | cg26133295 | cg26133295_TC21 | -0.0252496 |
| 46 | cg05318503 | cg05318503_TC21 | 1.51459197 |
| 47 | cg26266372 | cg26266372_BC21 | 0.78923889 |
| 48 | cg01724838 | cg01724838_BC21 | -2.1396127 |
| 49 | cg07521929 | cg07521929_BC21 | -0.6861258 |
| 50 | cg11329422 | cg11329422_TC21 | 0.12069017 |
| 51 | cg21869913 | cg21869913_BC21 | -2.3004957 |
| 52 | cg26567688 | cg26567688_TC21 | 0.56789357 |
| 53 | cg17519553 | cg17519553_TC21 | -2.1880007 |
| 54 | cg27080917 | cg27080917_TC21 | 1.1523088 |
| 55 | cg21291342 | cg21291342_TC21 | 0.69363028 |
| 56 | cg11128212 | cg11128212_TC21 | -1.3538109 |
| 57 | cg03193322 | cg03193322_TC21 | 0.20593851 |
| 58 | cg08975875 | cg08975875_TC21 | -3.6999623 |
| 59 | cg04258833 | cg04258833_TC21 | 0.43664105 |
| 60 | cg15111244 | cg15111244_BC21 | -0.3019132 |
| 61 | cg23235337 | cg23235337_BC21 | -0.0307941 |
| 62 | cg23364246 | cg23364246_BC21 | -0.3724054 |
| 63 | cg01414357 | cg01414357_TC21 | -1.8830335 |
| 64 | cg14258218 | cg14258218_BC21 | -0.5324316 |
| 65 | cg23658320 | cg23658320_TC21 | 1.43324194 |
| 66 | cg00335428 | cg00335428_TC21 | 1.13728467 |
| 67 | cg14480507 | cg14480507_TC21 | 1.82531835 |
| 68 | cg10160276 | cg10160276_TC21 | 0.24472693 |
| 69 | cg19805775 | cg19805775_TC21 | 1.32575132 |
| 70 | cg06474661 | cg06474661_BC21 | -4.8338735 |
| 71 | cg01349336 | cg01349336_TC21 | 0.11687444 |
| 72 | cg16284640 | cg16284640_TC21 | 2.66597769 |
| 73 | cg01202600 | cg01202600_BC21 | -0.0491843 |
| 74 | cg02467537 | cg02467537_BC21 | -1.0017445 |
| 75 | cg06087424 | cg06087424_TC21 | 2.08432373 |
| 76 | cg06215643 | cg06215643_TC21 | -0.0483848 |
| 77 | cg05554536 | cg05554536_BC21 | -0.9361594 |
| 78 | cg04606053 | cg04606053_TC11 | 0.67713544 |
| 79 | cg25743236 | cg25743236_BC21 | -1.3845165 |
| 80 | cg04990989 | cg04990989_TC11 | -3.8637648 |
| 81 | cg08172581 | cg08172581_BC21 | -4.0751442 |
| 82 | cg04593437 | cg04593437_BC21 | -1.8345389 |
| 83 | cg12712122 | cg12712122_BC21 | 1.20226139 |
| 84 | cg24120153 | cg24120153_TC21 | 0.12521582 |
| 85 | cg01415937 | cg01415937_BC21 | 1.35230804 |
| 86 | cg04932525 | cg04932525_TC21 | 0.51902437 |
| 87 | cg12217291 | cg12217291_TC21 | 0.90735691 |
| 88 | cg12977942 | cg12977942_TC21 | 0.04577517 |
| 89 | cg07106095 | cg07106095_BC21 | 0.28641352 |
| 90 | cg14396230 | cg14396230_TC21 | 0.17356839 |
| 91 | cg22079043 | cg22079043_TC21 | -1.462696 |
| 92 | cg12643970 | cg12643970_BC21 | 0.17109864 |
| 93 | cg08991794 | cg08991794_BC21 | 6.23687033 |
| 94 | cg07838270 | cg07838270_BC11 | 0.03705037 |
| 95 | cg12032603 | cg12032603_BC21 | -0.6451663 |
| 96 | cg21952614 | cg21952614_BC21 | 3.27323882 |
| 97 | cg08686580 | cg08686580_BC21 | -0.1652588 |
| 98 | cg10089656 | cg10089656_TC21 | 2.21635097 |
| 99 | cg20390245 | cg20390245_BC21 | -0.0008396 |
| 100 | cg20939696 | cg20939696_BC21 | 0.20906203 |
| 101 | cg06830006 | cg06830006_TC21 | 0.02416247 |
| 102 | cg05847388 | cg05847388_BC21 | 0.40108807 |
| 103 | cg02625749 | cg02625749_BC21 | -1.4383387 |
| 104 | cg22334681 | cg22334681_BC21 | -0.0030266 |
| 105 | cg18627729 | cg18627729_BC21 | -2.7944465 |
| 106 | cg08026464 | cg08026464_BC21 | 0.70472974 |
| 107 | cg01640660 | cg01640660_BC21 | -0.1953339 |
| 108 | cg02218090 | cg02218090_TC21 | 0.0101235 |
| 109 | cg18019618 | cg18019618_TC21 | 1.1581197 |
| 110 | cg17127722 | cg17127722_TC21 | -2.4394468 |
| 111 | cg02562900 | cg02562900_BC21 | 0.06795959 |
| 112 | cg05058758 | cg05058758_TC11 | 0.17260776 |
| 113 | cg20425731 | cg20425731_BC21 | 4.82712067 |
| 114 | cg16608498 | cg16608498_BC21 | 0.2854438 |
| 115 | cg19378060 | cg19378060_TC21 | 0.2249881 |
| 116 | cg07696673 | cg07696673_TC21 | 0.48111283 |
| 117 | cg15821498 | cg15821498_TC21 | 7.14752833 |
| 118 | cg06570734 | cg06570734_TC21 | -0.8166923 |
| 119 | cg02697654 | cg02697654_TC21 | -0.2080999 |
| 120 | cg02046881 | cg02046881_TC21 | 0.13466819 |
| 121 | cg00333755 | cg00333755_BC21 | -0.0962596 |
| 122 | cg23866612 | cg23866612_TC21 | 0.36121069 |
| 123 | cg08825673 | cg08825673_TC21 | -2.4652003 |
| 124 | cg27309179 | cg27309179_TC21 | 0.28445153 |
| 125 | cg06873980 | cg06873980_BC21 | 0.24830025 |
| 126 | cg13041597 | cg13041597_BC21 | 0.52893867 |
| 127 | cg02717567 | cg02717567_BC21 | 0.01438008 |
| 128 | cg08280787 | cg08280787_TC21 | -2.5935446 |
| 129 | cg15666659 | cg15666659_BC21 | -0.8517912 |
| 130 | cg04233669 | cg04233669_TC21 | 1.19044459 |
| 131 | cg06163904 | cg06163904_TC21 | -2.6753066 |
| 132 | cg15483437 | cg15483437_TC21 | 0.04278136 |
| 133 | cg09920605 | cg09920605_BC21 | 2.84083898 |
| 134 | cg18662507 | cg18662507_BC21 | -0.1876781 |
| 135 | cg10786503 | cg10786503_TC21 | -1.7956131 |
| 136 | cg06273398 | cg06273398_BC21 | 0.05041125 |
| 137 | cg04388666 | cg04388666_BC21 | -0.5128858 |
| 138 | cg09278021 | cg09278021_TC21 | -2.5047021 |
| 139 | cg10863857 | cg10863857_BC21 | 0.47510944 |
| 140 | cg08969925 | cg08969925_BC21 | 0.67410891 |
| 141 | cg00125768 | cg00125768_BC21 | 0.22003665 |
| 142 | cg06964816 | cg06964816_BC21 | -1.7043365 |
| 143 | cg23183121 | cg23183121_BC11 | -1.0282475 |
| 144 | cg12140760 | cg12140760_BC21 | 2.12847963 |
| 145 | cg15168132 | cg15168132_BC21 | 1.05828759 |
| 146 | cg05966241 | cg05966241_BC11 | -2.0698486 |
| 147 | cg26543112 | cg26543112_BC21 | -2.572941 |
| 148 | cg21426218 | cg21426218_BC11 | -0.6699589 |
| 149 | cg02780634 | cg02780634_TC21 | -0.9942581 |
| 150 | cg05193435 | cg05193435_BC21 | 0.54479472 |
| 151 | cg07766424 | cg07766424_TC21 | 0.1674337 |
| 152 | cg18161904 | cg18161904_BC21 | 2.76196314 |
| 153 | cg00108554 | cg00108554_BC21 | -1.0556439 |
| 154 | cg13576014 | cg13576014_TC21 | -1.4007695 |
| 155 | cg07286682 | cg07286682_BC21 | 1.11254338 |
| 156 | cg19956868 | cg19956868_TC21 | 1.484416 |
| 157 | cg09116560 | cg09116560_BC21 | 1.09509788 |
| 158 | cg12081443 | cg12081443_TC11 | -1.4960106 |
| 159 | cg01719210 | cg01719210_BC21 | 1.02705894 |
| 160 | cg01955153 | cg01955153_TC21 | -0.6525105 |
| 161 | cg13836708 | cg13836708_TC21 | 0.17933015 |
| 162 | cg17964080 | cg17964080_TC21 | 0.5687176 |
| 163 | cg22131612 | cg22131612_TC21 | 0.03948197 |
| 164 | cg23885431 | cg23885431_BC21 | -7.5189636 |
| 165 | cg14784405 | cg14784405_BC21 | 0.23838128 |
| 166 | cg02771214 | cg02771214_BC21 | 0.05918638 |
| 167 | cg17007574 | cg17007574_BC21 | 1.07679078 |
| 168 | cg25672774 | cg25672774_TC21 | 0.28987367 |
| 169 | cg03168508 | cg03168508_BC21 | -0.238696 |
| 170 | cg04742197 | cg04742197_TC21 | -0.7618232 |
| 171 | cg15145868 | cg15145868_TC21 | -2.1713877 |
| 172 | cg10716444 | cg10716444_TC21 | -1.9520256 |
| 173 | cg19662922 | cg19662922_TC21 | -0.4943549 |
| 174 | cg04362419 | cg04362419_TC21 | 0.13382316 |
| 175 | cg20928238 | cg20928238_BC21 | -1.7378305 |
| 176 | cg11235829 | cg11235829_TC21 | 0.90902902 |
| 177 | cg10676762 | cg10676762_BC21 | 0.27477033 |
| 178 | cg06181286 | cg06181286_TC21 | -3.0112292 |
| 179 | cg03822645 | cg03822645_BC21 | 0.51404021 |
| 180 | cg04523891 | cg04523891_BC21 | -0.4580664 |
| 181 | cg01949324 | cg01949324_BC21 | -5.517355 |
| 182 | cg09160344 | cg09160344_BC21 | -0.4685131 |
| 183 | cg16096180 | cg16096180_TC21 | 0.32125288 |
| 184 | cg12158483 | cg12158483_BC11 | 4.7617395 |
| 185 | cg00065944 | cg00065944_TC21 | -1.4472736 |
| 186 | cg03706376 | cg03706376_BC21 | 1.04827738 |
| 187 | cg27217933 | cg27217933_TC21 | 1.48762316 |
| 188 | cg20013586 | cg20013586_BC21 | 0.83133078 |
| 189 | cg03914820 | cg03914820_BC21 | 0.00525367 |
| 190 | cg11752152 | cg11752152_BC21 | 0.4303056 |
| 191 | cg19618374 | cg19618374_TC21 | 0.10984597 |
| 192 | cg23995459 | cg23995459_TC21 | -2.0325214 |
| 193 | cg00393047 | cg00393047_TC21 | 0.94801445 |
| 194 | cg12064504 | cg12064504_BC21 | 0.23962515 |
| 195 | cg09858677 | cg09858677_BC21 | 1.04424771 |
| 196 | cg13854896 | cg13854896_BC21 | -0.6465282 |
| 197 | cg12084036 | cg12084036_TC21 | 0.59749491 |
| 198 | cg21183455 | cg21183455_BC21 | 0.25879926 |
| 199 | cg09362608 | cg09362608_TC21 | 1.78800398 |
| 200 | cg23840079 | cg23840079_TC21 | -0.538143 |
| 201 | cg14179258 | cg14179258_TC21 | 0.89094206 |
| 202 | cg11499696 | cg11499696_BC21 | 2.18228155 |
| 203 | cg21325862 | cg21325862_BC21 | 0.0271921 |
| 204 | cg20742784 | cg20742784_BC21 | -0.7755805 |
| 205 | cg01138890 | cg01138890_BC21 | 1.97074905 |
| 206 | cg02050915 | cg02050915_TC21 | -0.0448531 |
| 207 | cg00549851 | cg00549851_TC21 | 0.24926475 |
| 208 | cg06838584 | cg06838584_TC21 | 0.69096832 |
| 209 | cg17440417 | cg17440417_BC21 | 0.59917822 |
| 210 | cg09561832 | cg09561832_TC21 | 1.83096673 |
| 211 | cg04761931 | cg04761931_BC21 | -1.7348024 |
| 212 | cg16096646 | cg16096646_TC21 | 0.03546119 |
| 213 | cg02167322 | cg02167322_BC21 | -0.1963626 |
| 214 | cg17400014 | cg17400014_TC21 | 0.07480938 |
| 215 | cg09231529 | cg09231529_BC21 | 1.33476889 |
| 216 | cg12641578 | cg12641578_TC11 | 14.2445117 |
| 217 | cg21500970 | cg21500970_BC21 | 1.08437491 |
| 218 | cg17182156 | cg17182156_BC21 | -0.8072162 |
| 219 | cg20959907 | cg20959907_TC11 | -0.2659821 |
| 220 | cg04179000 | cg04179000_TC21 | -1.0017341 |
| 221 | cg13672106 | cg13672106_BC21 | 0.41547188 |
| 222 | cg13256545 | cg13256545_BC21 | 0.10257848 |
| 223 | cg26665035 | cg26665035_TC11 | -1.5354946 |
| 224 | cg00743629 | cg00743629_TC21 | 0.09969755 |
| 225 | cg11971944 | cg11971944_BC21 | -1.2416269 |
| 226 | cg18392201 | cg18392201_TC21 | 0.48542094 |
| 227 | cg14479354 | cg14479354_TC21 | -1.318423 |
| 228 | ch.14.1488981R | ch.14.1488981R_BC21 | -3.5812296 |
| 229 | cg01190071 | cg01190071_TC21 | 1.14239354 |
| 230 | cg12600032 | cg12600032_BC21 | -2.0405229 |
| 231 | cg00101235 | cg00101235_BC21 | 1.22101839 |
| 232 | cg01082601 | cg01082601_BC21 | 1.45104616 |
| 233 | cg09229492 | cg09229492_BC21 | 0.69926379 |
| 234 | cg06646796 | cg06646796_BC21 | 1.74546791 |
| 235 | cg09243824 | cg09243824_BC11 | -0.6501693 |
| 236 | cg10366876 | cg10366876_BC21 | 0.25885317 |
| 237 | cg17438580 | cg17438580_TC21 | 1.08804226 |
| 238 | cg10946644 | cg10946644_BC21 | 0.43493419 |
| 239 | cg17316947 | cg17316947_BC21 | 0.01592296 |
| 240 | cg21735522 | cg21735522_BC21 | -1.7740914 |
| 241 | cg05882685 | cg05882685_BC21 | -0.8363921 |
| 242 | cg07465899 | cg07465899_TC21 | -0.0252378 |
| 243 | cg18268085 | cg18268085_BC21 | 0.32112879 |
| 244 | cg13018715 | cg13018715_TC21 | -6.5327866 |
| 245 | cg15969090 | cg15969090_TC21 | -0.0549494 |
| 246 | cg13640414 | cg13640414_BC21 | -6.0533186 |
| 247 | cg10046771 | cg10046771_BC21 | 0.02610185 |
| 248 | cg22406238 | cg22406238_TC21 | 1.35754723 |
| 249 | cg11670749 | cg11670749_BC21 | 1.09153701 |
| 250 | cg05064739 | cg05064739_TC21 | -0.0010492 |
| 251 | cg18831304 | cg18831304_BC21 | 0.30791679 |
| 252 | cg01393060 | cg01393060_BC21 | -1.5268679 |
| 253 | cg00621366 | cg00621366_BC21 | 0.2833476 |
| 254 | cg12072997 | cg12072997_BC21 | 0.21933059 |
| 255 | cg20749846 | cg20749846_TC21 | 0.63777316 |
| 256 | cg03604110 | cg03604110_TC21 | 1.11312299 |
| 257 | cg13021857 | cg13021857_TC21 | -0.0795231 |
| 258 | cg05026404 | cg05026404_BC21 | 0.006176 |
| 259 | cg06975256 | cg06975256_TC21 | 0.68543324 |
| 260 | cg16964198 | cg16964198_BC21 | 0.49533928 |
| 261 | cg02565196 | cg02565196_TC21 | -1.373788 |
| 262 | cg27185521 | cg27185521_BC21 | 2.32866879 |
| 263 | cg00906581 | cg00906581_BC21 | 2.31881856 |
| 264 | cg18407136 | cg18407136_TC21 | 0.00467386 |
| 265 | cg02505117 | cg02505117_TC21 | 0.87456183 |
| 266 | cg10494843 | cg10494843_BC21 | -0.1699496 |
| 267 | cg06967145 | cg06967145_TC21 | 0.01541606 |
| 268 | cg02688432 | cg02688432_BC21 | 0.87037454 |
| 269 | cg03400695 | cg03400695_BC21 | -0.4194804 |
| 270 | cg00582902 | cg00582902_TC21 | 2.42753231 |
| 271 | cg12964935 | cg12964935_BC21 | -2.8925227 |
| 272 | cg17093466 | cg17093466_TC21 | -0.0733928 |
| 273 | cg06700275 | cg06700275_BC21 | -0.0118161 |
| 274 | cg05220114 | cg05220114_BC21 | 0.76615153 |
| 275 | cg15160963 | cg15160963_TC21 | 0.11964472 |
| 276 | cg11466773 | cg11466773_BC21 | 4.89084723 |
| 277 | cg18206764 | cg18206764_BC21 | 0.29770609 |
| 278 | cg19486038 | cg19486038_BC21 | 1.87084481 |
| 279 | cg04055211 | cg04055211_BC21 | 0.15329061 |
| 280 | cg26222730 | cg26222730_TC21 | -2.3595048 |
| 281 | cg20967006 | cg20967006_TC21 | 0.57874237 |
| 282 | cg26064870 | cg26064870_TC21 | -1.9898802 |
| 283 | cg12753934 | cg12753934_BC21 | -0.2196033 |
| 284 | cg05980523 | cg05980523_BC21 | -1.0162077 |
| 285 | cg22140708 | cg22140708_TC21 | -1.7821657 |
| 286 | cg21733098 | cg21733098_TC21 | -0.2211784 |
| 287 | cg19805228 | cg19805228_TC21 | -1.300055 |
| 288 | cg16059665 | cg16059665_BC21 | 0.21684758 |
| 289 | cg03025176 | cg03025176_BC21 | 0.31146765 |
| 290 | cg03464371 | cg03464371_BC21 | 0.2567447 |
| 291 | cg12075011 | cg12075011_TC21 | 1.42405055 |
| 292 | cg21192606 | cg21192606_TC21 | -1.0501194 |
| 293 | cg25547830 | cg25547830_BC21 | 0.90273115 |
| 294 | cg00011861 | cg00011861_BC21 | 0.42920639 |
| 295 | cg14288579 | cg14288579_BC21 | 0.69338456 |
| 296 | cg14671000 | cg14671000_BC21 | 0.21398908 |
| 297 | cg02711042 | cg02711042_BC21 | 0.81028616 |
| 298 | cg01188867 | cg01188867_BC21 | -1.2643602 |
| 299 | cg19638381 | cg19638381_BC21 | -0.7243194 |
| 300 | cg25627956 | cg25627956_TC21 | 0.22943728 |
| 301 | cg08826184 | cg08826184_TC21 | 0.52557086 |
| 302 | cg02101201 | cg02101201_BC21 | -3.33686 |
| 303 | cg18383660 | cg18383660_BC21 | 0.05440262 |
| 304 | cg25670171 | cg25670171_BC21 | -0.0409493 |
| 305 | cg16949001 | cg16949001_BC21 | 1.62775246 |
| 306 | cg21919596 | cg21919596_BC21 | 0.80131972 |
| 307 | cg20207443 | cg20207443_BC21 | 0.05454387 |
| 308 | cg10769507 | cg10769507_TC21 | -1.0022546 |
| 309 | cg13761004 | cg13761004_BC21 | -1.69215 |
| 310 | cg10139476 | cg10139476_BC21 | -4.798893 |
| 311 | cg14177258 | cg14177258_BC21 | -0.1190869 |
| 312 | cg24255121 | cg24255121_TC21 | -2.7905524 |
| 313 | cg19690792 | cg19690792_BC21 | -0.1565042 |
| 314 | cg25003531 | cg25003531_BC21 | 5.15199129 |
| 315 | cg21493290 | cg21493290_TC21 | -1.5503428 |
| 316 | cg04793632 | cg04793632_TC21 | -1.6446944 |
| 317 | cg14373685 | cg14373685_BC21 | 0.47504338 |
| 318 | cg27151362 | cg27151362_TC21 | 4.83218163 |
| 319 | cg23270113 | cg23270113_BC11 | -0.2063574 |
| 320 | cg19870401 | cg19870401_TC21 | 0.75809353 |
| 321 | cg21099238 | cg21099238_BC21 | 0.14561595 |
| 322 | cg01907837 | cg01907837_TC21 | 0.21724021 |
| 323 | cg08637691 | cg08637691_BC21 | 20.5496377 |
| 324 | cg01456831 | cg01456831_BC21 | -0.8708673 |
| 325 | cg23816136 | cg23816136_BC21 | -0.2516228 |
| 326 | cg05362029 | cg05362029_TC21 | 0.46809975 |
| 327 | cg00211944 | cg00211944_TC21 | -0.1386601 |
| 328 | cg11143399 | cg11143399_TC21 | -0.626617 |
| 329 | cg03611750 | cg03611750_BC21 | -0.4219002 |
| 330 | cg20024393 | cg20024393_BC21 | -1.466813 |
| 331 | cg14664341 | cg14664341_TC21 | -0.0366047 |
| 332 | cg11415498 | cg11415498_BC21 | -2.9452246 |
| 333 | cg24478949 | cg24478949_TC21 | 4.43168587 |
| 334 | cg13648527 | cg13648527_BC21 | 2.15120304 |
| 335 | cg26210302 | cg26210302_BC21 | -0.0972206 |
| 336 | cg13261390 | cg13261390_BC21 | 0.15651137 |
| 337 | cg03672117 | cg03672117_BC21 | 0.42003527 |
| 338 | cg26175846 | cg26175846_TC21 | -0.1829544 |
| 339 | cg24283375 | cg24283375_BC21 | 3.37409369 |
| 340 | cg15071067 | cg15071067_TC21 | 1.10607884 |
| 341 | cg20104055 | cg20104055_BC21 | -0.1455463 |
| 342 | cg25620430 | cg25620430_BC21 | 1.3502478 |
| 343 | cg12135457 | cg12135457_TC21 | -2.3198009 |
| 344 | cg14387280 | cg14387280_BC21 | 0.37409514 |
| 345 | cg26366958 | cg26366958_BC21 | 1.68493121 |
| 346 | cg11929780 | cg11929780_BC21 | -3.267122 |
| 347 | cg27214009 | cg27214009_TC21 | 0.00807863 |
| 348 | cg02340116 | cg02340116_BC21 | -1.6038252 |
| 349 | cg03580331 | cg03580331_BC21 | -1.0419238 |
| 350 | cg25880623 | cg25880623_TC21 | -5.1305369 |
| 351 | cg21664107 | cg21664107_TC21 | -1.6487305 |
| 352 | cg07330997 | cg07330997_TC21 | -0.3833209 |
| 353 | cg15197125 | cg15197125_TC21 | -0.0392487 |
| 354 | cg21043312 | cg21043312_TC21 | -0.0216097 |
| 355 | cg06663737 | cg06663737_BC21 | -0.0724975 |
| 356 | cg12524428 | cg12524428_TC21 | 0.18860086 |
| 357 | cg06929174 | cg06929174_BC21 | 0.53628316 |
| 358 | cg05910170 | cg05910170_BC21 | -0.5063215 |
| 359 | cg21126969 | cg21126969_BC21 | -0.0723215 |
| 360 | cg15047198 | cg15047198_BC21 | 0.07098668 |
| 361 | cg14215390 | cg14215390_BC21 | 0.20175113 |
| 362 | cg07393857 | cg07393857_BC11 | 0.94250003 |
| 363 | cg24832950 | cg24832950_TC21 | -1.2828788 |
| 364 | cg24297873 | cg24297873_BC11 | -0.2950436 |
| 365 | cg11553665 | cg11553665_BC21 | -0.2765943 |
| 366 | cg16857134 | cg16857134_TC21 | 0.20647744 |
| 367 | cg05306305 | cg05306305_BC21 | -1.3562407 |
| 368 | cg19893132 | cg19893132_TC21 | -0.0600171 |
| 369 | cg26801943 | cg26801943_BC21 | -1.9357158 |
| 370 | cg21836905 | cg21836905_BC21 | 1.83205813 |
| 371 | cg21774136 | cg21774136_BC21 | 1.01649534 |
| 372 | cg04087729 | cg04087729_BC21 | -0.0170682 |
| 373 | cg15300488 | cg15300488_BC21 | 0.43637837 |
| 374 | cg20010411 | cg20010411_BC21 | -0.1858643 |
| 375 | cg17754737 | cg17754737_BC21 | -0.6209382 |
| 376 | cg08154622 | cg08154622_BC21 | 0.35549731 |
| 377 | cg24790501 | cg24790501_TC21 | 0.22360209 |
| 378 | cg24659873 | cg24659873_TC21 | 0.0090974 |
| 379 | cg22844035 | cg22844035_TC21 | 1.54436247 |
| 380 | cg03518311 | cg03518311_BC21 | -0.0363659 |
| 381 | cg25745729 | cg25745729_BC21 | 0.32700894 |
| 382 | cg26698979 | cg26698979_TC21 | -0.0377186 |
| 383 | cg26708596 | cg26708596_TC21 | 1.36790278 |
| 384 | cg12484135 | cg12484135_BC21 | -2.5647691 |
| 385 | cg10451759 | cg10451759_BC21 | 0.24111379 |
| 386 | cg06166559 | cg06166559_TC21 | 1.06680396 |
| 387 | cg15115421 | cg15115421_BC21 | -0.0161834 |
| 388 | cg04567124 | cg04567124_BC21 | 0.15021121 |
| 389 | cg19551813 | cg19551813_BC21 | 0.22412183 |
| 390 | cg04811706 | cg04811706_BC21 | -0.0361932 |
| 391 | cg18715236 | cg18715236_BC21 | -1.5710701 |
| 392 | cg00224222 | cg00224222_BC21 | -0.6202337 |
| 393 | cg23198707 | cg23198707_BC11 | 1.37501486 |
| 394 | cg06114934 | cg06114934_TC21 | -0.0322837 |
| 395 | cg08006519 | cg08006519_BC21 | -0.0045493 |
| 396 | cg01816748 | cg01816748_TC21 | -0.6417543 |
| 397 | cg10331773 | cg10331773_TC21 | -0.4308137 |
| 398 | cg01907945 | cg01907945_TC21 | -0.4206214 |
| 399 | cg03763997 | cg03763997_BC21 | -0.9136409 |
| 400 | cg05116536 | cg05116536_BC21 | 0.64712269 |
| 401 | cg15350922 | cg15350922_TC21 | 2.83344889 |
| 402 | cg22968887 | cg22968887_BC21 | 2.99909685 |
| 403 | cg00518276 | cg00518276_BC21 | 0.76145688 |
| 404 | cg03352438 | cg03352438_TC21 | 0.47885524 |
| 405 | cg11001739 | cg11001739_TC11 | -1.4254608 |
| 406 | cg22649201 | cg22649201_BC21 | -0.6241682 |
| 407 | cg20736045 | cg20736045_BC21 | 0.51549112 |
| 408 | cg26800429 | cg26800429_BC21 | -0.1103572 |
| 409 | cg02113955 | cg02113955_BC21 | -2.1242777 |
| 410 | cg05891372 | cg05891372_BC21 | -1.7639278 |
| 411 | cg27468506 | cg27468506_TC21 | -0.1020667 |
| 412 | cg05819613 | cg05819613_BC21 | 0.24414154 |
| 413 | cg09416608 | cg09416608_BC21 | -0.0817109 |
| 414 | cg06330230 | cg06330230_BC21 | 0.93361669 |
| 415 | cg18391753 | cg18391753_BC21 | -0.4904851 |
| 416 | cg19422140 | cg19422140_BC21 | 0.79074342 |
| 417 | cg18772677 | cg18772677_TC21 | 0.25674901 |
| 418 | cg07791803 | cg07791803_TC21 | 1.14684636 |
| 419 | cg16049494 | cg16049494_BC21 | 0.10108152 |
| 420 | cg10556472 | cg10556472_TC11 | 0.53905006 |
| 421 | cg19852518 | cg19852518_BC21 | 0.76582984 |
| 422 | cg13714975 | cg13714975_BC21 | 0.82418914 |
| 423 | cg21195120 | cg21195120_BC21 | 0.1679174 |
| 424 | cg03905757 | cg03905757_BC21 | 0.48466315 |
| 425 | cg25354733 | cg25354733_BC21 | -0.0008883 |
| 426 | cg20022139 | cg20022139_BC21 | -0.2768791 |
| 427 | cg23318800 | cg23318800_BC21 | 0.06162362 |
| 428 | cg05365735 | cg05365735_TC21 | 0.67355917 |
| 429 | cg00700817 | cg00700817_BC21 | -0.2361515 |
| 430 | cg11403708 | cg11403708_BC11 | -1.4486439 |
| 431 | cg24389924 | cg24389924_TC21 | 1.37509348 |
| 432 | cg20451208 | cg20451208_TC21 | 0.20810153 |
| 433 | cg26886965 | cg26886965_BC21 | -0.6917199 |
| 434 | cg03142586 | cg03142586_TC11 | 4.0475601 |
| 435 | cg01606885 | cg01606885_BC21 | -5.8467741 |
| 436 | cg02493487 | cg02493487_TC21 | 3.75818699 |
| 437 | cg13714995 | cg13714995_BC21 | -1.5289607 |
| 438 | cg12815849 | cg12815849_TC21 | 0.17781413 |
| 439 | cg01779220 | cg01779220_BC21 | 0.02298 |
| 440 | cg17614403 | cg17614403_BC21 | 1.87958292 |
| 441 | cg24450548 | cg24450548_BC21 | 2.06343884 |
| 442 | cg26144246 | cg26144246_BC21 | -0.0574202 |
| 443 | cg22660609 | cg22660609_BC11 | 0.61932447 |
| 444 | cg04455646 | cg04455646_TC11 | -0.1864993 |
| 445 | cg01328467 | cg01328467_BC21 | 0.72288434 |
| 446 | cg03396826 | cg03396826_BC11 | -0.1619124 |
| 447 | cg21216024 | cg21216024_TC21 | 0.09649375 |
| 448 | cg09611599 | cg09611599_TC21 | -0.1911545 |
| 449 | cg11975053 | cg11975053_BC21 | -1.5141367 |
| 450 | cg26926221 | cg26926221_TC21 | 1.38473021 |
| 451 | cg27106643 | cg27106643_TC11 | -0.3035432 |
| 452 | cg23434203 | cg23434203_TC21 | 0.19055681 |
| 453 | cg04848107 | cg04848107_BC21 | 0.75163646 |
| 454 | cg00810441 | cg00810441_BC21 | 0.7673942 |
| 455 | cg03199366 | cg03199366_BC21 | 0.33632255 |
| 456 | cg22362956 | cg22362956_BC21 | 1.19655818 |
| 457 | cg03642431 | cg03642431_TC21 | 0.38409799 |
| 458 | cg13648741 | cg13648741_TC21 | 3.50127616 |
| 459 | cg17453778 | cg17453778_TC21 | 2.0661296 |
| 460 | cg24748078 | cg24748078_BC21 | -0.2755449 |
| 461 | cg11182093 | cg11182093_TC21 | 0.31650385 |
| 462 | cg01007323 | cg01007323_BC21 | 0.30283328 |
| 463 | cg15188547 | cg15188547_BC21 | -0.0004166 |
| 464 | cg12806822 | cg12806822_TC21 | 0.0344883 |
| 465 | cg15921319 | cg15921319_BC21 | 0.83757 |
| 466 | cg04444949 | cg04444949_TC21 | -2.4134684 |
| 467 | cg04655834 | cg04655834_BC21 | 0.4216153 |
| 468 | cg03470149 | cg03470149_BC21 | 1.19554472 |
| 469 | cg23913963 | cg23913963_BC21 | -0.5951623 |
| 470 | cg19309562 | cg19309562_TC21 | 0.63550038 |
| 471 | cg05655307 | cg05655307_BC21 | 0.41077642 |
| 472 | cg20902817 | cg20902817_TC21 | 3.66260435 |
| 473 | cg22650206 | cg22650206_BC21 | -2.6464615 |
| 474 | cg05504820 | cg05504820_BC21 | 1.36066848 |
| 475 | cg19513335 | cg19513335_TC21 | 1.27419609 |
| 476 | cg03112887 | cg03112887_BC21 | 1.56742921 |
| 477 | cg16358252 | cg16358252_BC21 | 2.29621415 |
| 478 | cg07452397 | cg07452397_TC21 | 0.36372228 |
| 479 | cg05107803 | cg05107803_BC21 | -0.0794903 |
| 480 | cg00096979 | cg00096979_TC11 | -2.4939341 |
| 481 | cg09490370 | cg09490370_TC21 | -0.2092508 |
| 482 | cg02041470 | cg02041470_TC21 | -0.4516546 |
| 483 | cg17756325 | cg17756325_BC21 | 2.14298966 |
| 484 | cg17035132 | cg17035132_BC11 | 1.1389588 |
| 485 | cg09025605 | cg09025605_BC21 | 1.58178157 |
| 486 | cg07360076 | cg07360076_TC11 | 0.90118862 |
| 487 | cg15654892 | cg15654892_BC21 | 0.14471016 |
| 488 | cg21074110 | cg21074110_BC21 | 0.17292412 |
| 489 | cg25088843 | cg25088843_TC21 | 0.7246755 |
| 490 | cg27656302 | cg27656302_TC21 | -1.302258 |
| 491 | cg01206874 | cg01206874_BC21 | 0.09320189 |
| 492 | cg26051165 | cg26051165_TC21 | 0.34336621 |
| 493 | cg06554081 | cg06554081_BC21 | 1.23573916 |
| 494 | cg04679515 | cg04679515_BC21 | -0.6382531 |
| 495 | cg18255101 | cg18255101_BC21 | 1.10729181 |
| 496 | cg10076783 | cg10076783_BC21 | -0.7303595 |
| 497 | cg24614722 | cg24614722_BC21 | 0.26916791 |
| 498 | cg14660449 | cg14660449_TC21 | 0.8917645 |
| 499 | cg10432942 | cg10432942_TC21 | -0.0262648 |
| 500 | cg20597575 | cg20597575_TC21 | -3.5123281 |
| 501 | cg07843181 | cg07843181_BC21 | -0.269222 |
| 502 | cg04122287 | cg04122287_BC21 | 0.24210733 |
| 503 | cg19379721 | cg19379721_BC21 | -0.6902487 |
| 504 | cg10228719 | cg10228719_TC21 | 0.23135454 |
| 505 | cg21794496 | cg21794496_TC21 | -0.1705185 |
| 506 | cg09161689 | cg09161689_TC21 | -2.0261294 |
| 507 | cg13375654 | cg13375654_BC21 | 1.77879735 |
| 508 | cg05593628 | cg05593628_BC21 | 3.22894949 |
| 509 | cg00457009 | cg00457009_TC21 | 2.02061762 |
| 510 | cg05266321 | cg05266321_BC21 | 0.08903254 |
| 511 | cg12456014 | cg12456014_TC21 | -3.418042 |
| 512 | cg06026473 | cg06026473_TC21 | 0.01708286 |
| 513 | cg13878276 | cg13878276_BC21 | -2.688676 |
| 514 | cg03271007 | cg03271007_BC21 | 1.24472959 |
| 515 | cg09118175 | cg09118175_TC21 | 1.43483047 |
| 516 | cg21652108 | cg21652108_TC21 | 0.31599698 |
| 517 | cg15251539 | cg15251539_TC21 | -0.8853366 |
| 518 | cg13704954 | cg13704954_BC21 | 0.41408202 |
| 519 | cg21803548 | cg21803548_TC21 | -0.8176862 |
| 520 | cg20173011 | cg20173011_BC21 | -0.4074067 |
| 521 | cg11399222 | cg11399222_BC21 | -1.1918113 |
| 522 | cg12050135 | cg12050135_TC21 | 0.04921171 |
| 523 | cg22512663 | cg22512663_BC21 | 0.51689478 |
| 524 | cg01560972 | cg01560972_BC11 | 0.04455053 |
| 525 | cg16714760 | cg16714760_BC21 | -4.0570461 |
| 526 | cg14819603 | cg14819603_BC21 | 0.67357282 |
| 527 | cg06293611 | cg06293611_BC21 | 1.94330943 |
| 528 | cg02643387 | cg02643387_TC21 | -0.2294503 |
| 529 | cg12542202 | cg12542202_BC21 | 1.24449014 |
| 530 | cg00420390 | cg00420390_BC21 | -0.0284379 |
| 531 | cg19901523 | cg19901523_TC11 | -6.1473214 |
| 532 | cg14622770 | cg14622770_TC21 | 0.50656079 |
| 533 | cg06488148 | cg06488148_TC21 | 0.01816993 |
| 534 | cg10813948 | cg10813948_BC21 | 0.13415961 |
| 535 | cg05305497 | cg05305497_TC21 | 0.62429338 |
| 536 | cg13545097 | cg13545097_BC21 | 1.24292294 |
| 537 | cg16756674 | cg16756674_TC21 | 0.61298653 |
| 538 | cg11429644 | cg11429644_BC21 | 0.00413317 |
| 539 | cg12500951 | cg12500951_TC11 | -0.0792559 |
| 540 | cg16848272 | cg16848272_TC21 | 0.08656064 |
| 541 | cg17057301 | cg17057301_TC21 | -0.6478899 |
| 542 | cg24160066 | cg24160066_TC21 | -0.7933751 |
| 543 | cg00339281 | cg00339281_BC21 | 0.52101974 |
| 544 | cg09463001 | cg09463001_BC21 | 0.08283019 |
| 545 | cg20978972 | cg20978972_BC21 | 0.06224826 |
| 546 | cg21732649 | cg21732649_TC21 | -1.1112319 |
| 547 | cg15778873 | cg15778873_TC21 | 0.34020677 |
| 548 | cg12080831 | cg12080831_BC21 | 1.3341749 |
| 549 | cg10955545 | cg10955545_TC21 | 0.1839785 |
| 550 | cg07695330 | cg07695330_TC21 | -0.1234921 |
| 551 | cg12371557 | cg12371557_BC21 | 0.05017012 |
| 552 | cg12970409 | cg12970409_BC21 | -0.2119263 |
| 553 | cg16307759 | cg16307759_TC21 | -0.6434582 |
| 554 | cg20744727 | cg20744727_BC21 | -0.3845509 |
| 555 | cg07396872 | cg07396872_BC21 | -0.5056655 |
| 556 | cg17072755 | cg17072755_TC21 | 0.32538259 |
| 557 | cg25809059 | cg25809059_BC21 | 0.11152325 |
| 558 | cg21959717 | cg21959717_BC21 | 0.34606242 |
| 559 | cg07571438 | cg07571438_TC21 | -0.013882 |
| 560 | cg22494026 | cg22494026_TC21 | 0.60636205 |
| 561 | cg08497455 | cg08497455_BC21 | 0.09681665 |
| 562 | cg13336326 | cg13336326_BC21 | 0.04721564 |
| 563 | cg09569166 | cg09569166_BC21 | 0.14463837 |
| 564 | cg27233850 | cg27233850_BC21 | 0.14545051 |
| 565 | cg17206645 | cg17206645_BC21 | -0.8007565 |
| 566 | cg04028616 | cg04028616_BC21 | 0.70784021 |
| 567 | cg06012872 | cg06012872_TC21 | 0.6566766 |
| 568 | cg00168783 | cg00168783_BC21 | -0.4815368 |
| 569 | cg21584226 | cg21584226_BC21 | -0.004152 |
| 570 | cg06347427 | cg06347427_BC21 | -0.9274072 |
| 571 | cg13393830 | cg13393830_BC21 | 1.63722327 |
| 572 | cg15787491 | cg15787491_BC21 | 0.46007411 |
| 573 | cg06358534 | cg06358534_BC21 | 0.11163208 |
| 574 | cg16614786 | cg16614786_TC21 | 0.2756168 |
| 575 | cg21153976 | cg21153976_TC21 | 0.3645974 |
| 576 | cg11315986 | cg11315986_BC21 | -0.3468881 |
| 577 | cg16856049 | cg16856049_TC21 | 0.89666973 |
| 578 | cg02857142 | cg02857142_TC21 | 0.13744976 |
| 579 | cg17758496 | cg17758496_BC21 | -0.2701709 |
| 580 | cg11513812 | cg11513812_BC21 | -0.2123523 |
| 581 | cg24344074 | cg24344074_TC21 | -0.1269092 |
| 582 | cg22388982 | cg22388982_TC11 | 2.03304832 |
| 583 | cg15299402 | cg15299402_BC21 | 0.62115207 |
| 584 | cg18986110 | cg18986110_BC21 | 0.39551211 |
| 585 | cg14499211 | cg14499211_BC21 | 7.93872707 |
| 586 | cg11155016 | cg11155016_BC21 | -0.1229628 |
| 587 | cg14349763 | cg14349763_TC11 | -0.2736904 |
| 588 | cg00088575 | cg00088575_TC21 | 0.19808375 |
| 589 | cg21714581 | cg21714581_BC21 | -1.4840989 |
| 590 | cg05094970 | cg05094970_BC21 | 0.36970507 |
| 591 | cg02660271 | cg02660271_TC21 | 0.88197531 |
| 592 | cg11123493 | cg11123493_TC21 | 1.17727597 |
| 593 | cg10888052 | cg10888052_TC21 | -0.2131584 |
| 594 | cg15234523 | cg15234523_BC21 | 0.97544146 |
| 595 | cg14343953 | cg14343953_TC21 | 0.90745333 |
| 596 | cg00961932 | cg00961932_BC21 | 0.98307751 |
| 597 | cg21636911 | cg21636911_TC21 | 0.80154946 |
| 598 | cg09308553 | cg09308553_TC21 | -2.5996934 |
| 599 | cg01798250 | cg01798250_BC21 | -0.5144326 |
| 600 | cg27240618 | cg27240618_TC21 | 0.11282838 |
| 601 | cg01676439 | cg01676439_BC21 | -1.8801293 |
| 602 | cg09107438 | cg09107438_TC21 | 0.056936 |
| 603 | cg00114478 | cg00114478_TC21 | 0.32096262 |
| 604 | cg15528441 | cg15528441_TC21 | -1.7287823 |
| 605 | cg05580734 | cg05580734_BC21 | 0.01470325 |
| 606 | cg06442089 | cg06442089_TC21 | 0.66615427 |
| 607 | cg13832333 | cg13832333_BC21 | 0.42355287 |
| 608 | cg04104329 | cg04104329_BC21 | 2.7066739 |
| 609 | cg20432824 | cg20432824_BC21 | -0.2155997 |
| 610 | cg21230675 | cg21230675_BC21 | -1.3844851 |
| 611 | cg24200825 | cg24200825_TC21 | -5.4333171 |
| 612 | cg26448931 | cg26448931_TC21 | 0.98437529 |
| 613 | cg02651683 | cg02651683_BC21 | -1.2212097 |
| 614 | cg18456755 | cg18456755_TC21 | 0.07183137 |
| 615 | cg00036045 | cg00036045_TC21 | 0.41707122 |
| 616 | cg07265477 | cg07265477_BC21 | 0.38660024 |
| 617 | cg16078124 | cg16078124_TC21 | -2.2850606 |
| 618 | cg05202858 | cg05202858_BC21 | -0.4814246 |
| 619 | cg18997137 | cg18997137_TC21 | 1.13901428 |
| 620 | cg00241638 | cg00241638_TC21 | -2.4557821 |
| 621 | cg07816205 | cg07816205_BC21 | -0.1883747 |
| 622 | cg11833003 | cg11833003_TC21 | -1.004785 |
| 623 | cg06837444 | cg06837444_BC21 | -0.1834143 |
| 624 | cg01837113 | cg01837113_BC21 | 0.36623211 |
| 625 | cg05973057 | cg05973057_BC21 | -5.6118386 |
| 626 | cg11135072 | cg11135072_TC21 | -3.6248462 |
| 627 | cg18582010 | cg18582010_TC21 | 1.78085393 |
| 628 | cg03738328 | cg03738328_BC21 | -0.8585183 |
| 629 | cg14283328 | cg14283328_BC21 | -0.374237 |
| 630 | cg08607001 | cg08607001_BC21 | 0.1160775 |
| 631 | cg15142312 | cg15142312_BC21 | -0.2462502 |
| 632 | cg15210447 | cg15210447_TC21 | 0.46869941 |
| 633 | cg23353113 | cg23353113_BC21 | -0.3378065 |
| 634 | cg03002271 | cg03002271_BC21 | -3.7248912 |
| 635 | cg11652765 | cg11652765_TC21 | -0.373647 |
| 636 | cg17982058 | cg17982058_TC21 | 2.57457134 |
| 637 | cg09654796 | cg09654796_BC21 | -0.4395494 |
| 638 | cg24170996 | cg24170996_BC21 | 0.58678904 |
| 639 | cg17876463 | cg17876463_TC21 | -0.2645858 |
| 640 | cg23196504 | cg23196504_BC21 | -2.8548648 |
| 641 | cg04178156 | cg04178156_TC21 | 0.84674511 |
| 642 | cg00843009 | cg00843009_TC21 | 1.0236518 |
| 643 | cg06530848 | cg06530848_TC21 | -0.3745473 |
| 644 | cg27485716 | cg27485716_BC11 | 7.4461356 |
| 645 | cg11124652 | cg11124652_TC21 | 0.83804808 |
| 646 | cg09837155 | cg09837155_TC21 | 1.98522965 |
| 647 | cg21393124 | cg21393124_BC21 | 1.78207767 |
| 648 | cg04905408 | cg04905408_BC21 | 1.40674438 |
| 649 | cg06094611 | cg06094611_BC21 | 1.02374623 |
| 650 | cg20833872 | cg20833872_TC21 | 0.16317142 |
| 651 | cg18586983 | cg18586983_TC11 | -0.064308 |
| 652 | cg04593531 | cg04593531_BC21 | 0.41251401 |
| 653 | cg03573446 | cg03573446_TC21 | 2.02348399 |
| 654 | cg12576709 | cg12576709_BC21 | 0.84366827 |
| 655 | cg22565251 | cg22565251_TC21 | -1.4788967 |
| 656 | cg10837229 | cg10837229_TC21 | 0.50255165 |
| 657 | cg19683551 | cg19683551_TC21 | -1.0127475 |
| 658 | cg13188283 | cg13188283_BC21 | -0.3983882 |
| 659 | cg12520319 | cg12520319_BC21 | 0.44423213 |
| 660 | cg15377610 | cg15377610_BC21 | -1.5114998 |
| 661 | cg13951450 | cg13951450_TC21 | 0.12160435 |
| 662 | cg10634115 | cg10634115_TC21 | 0.05622361 |
| 663 | cg22889455 | cg22889455_BC21 | -0.162164 |
| 664 | cg22047377 | cg22047377_TC21 | -0.8090509 |
| 665 | cg26515685 | cg26515685_BC21 | -0.7237877 |
| 666 | cg03959465 | cg03959465_TC21 | 0.72348328 |
| 667 | cg03550951 | cg03550951_BC21 | -0.2717212 |
| 668 | cg17994214 | cg17994214_TC21 | 0.23182949 |
| 669 | cg15687530 | cg15687530_BC11 | -0.0221762 |
| 670 | cg21986629 | cg21986629_BC21 | 0.21104159 |
| 671 | cg19679501 | cg19679501_TC21 | -1.8008686 |
| 672 | cg04858539 | cg04858539_BC21 | 1.0562897 |
| 673 | cg03652715 | cg03652715_TC11 | -1.1778056 |
| 674 | cg01212120 | cg01212120_TC21 | -0.0293719 |
| 675 | cg09772831 | cg09772831_BC21 | -0.6144849 |
| 676 | cg13130588 | cg13130588_BC11 | -0.4550635 |
| 677 | cg01669244 | cg01669244_BC21 | 0.04444198 |
| 678 | cg07698725 | cg07698725_BC21 | 3.17150064 |
| 679 | cg02458846 | cg02458846_TC21 | 0.1259603 |
| 680 | cg20317140 | cg20317140_TC21 | -1.0935413 |
| 681 | cg04669218 | cg04669218_BC21 | 0.6813682 |
| 682 | cg05402382 | cg05402382_BC21 | -1.734865 |
| 683 | cg25401874 | cg25401874_TC21 | 0.65115901 |
| 684 | cg12449295 | cg12449295_BC21 | 2.67162474 |
| 685 | cg24616450 | cg24616450_BC21 | 0.0231734 |
| 686 | cg11854772 | cg11854772_BC21 | 2.01248738 |
| 687 | cg26734064 | cg26734064_BC21 | -0.0421183 |
| 688 | cg06449058 | cg06449058_BC21 | 0.33727199 |
| 689 | cg27320439 | cg27320439_BC21 | 0.49846836 |
| 690 | cg22890765 | cg22890765_BC21 | -0.9945688 |
| 691 | cg07709065 | cg07709065_TC21 | -0.1041729 |
| 692 | cg14412891 | cg14412891_TC21 | 0.09646349 |
| 693 | cg16764008 | cg16764008_TC21 | 0.65492509 |
| 694 | cg16512376 | cg16512376_BC21 | 0.48805221 |
| 695 | cg23652734 | cg23652734_TC21 | -1.2403905 |
| 696 | cg08638989 | cg08638989_BC21 | -3.166762 |
| 697 | cg11122885 | cg11122885_TC21 | -1.4325197 |
| 698 | cg16810279 | cg16810279_TC11 | -0.0878969 |
| 699 | cg09049447 | cg09049447_BC21 | -0.2678818 |
| 700 | cg19966745 | cg19966745_TC21 | -0.4417672 |
| 701 | cg06833271 | cg06833271_BC21 | -1.6264028 |
| 702 | cg12671193 | cg12671193_BC21 | 0.36615931 |
| 703 | cg12899747 | cg12899747_TC21 | -3.2247285 |
| 704 | cg27212232 | cg27212232_BC21 | 1.14475965 |
| 705 | cg04193015 | cg04193015_TC21 | -2.5564571 |
| 706 | cg07721409 | cg07721409_TC21 | -0.1940563 |
| 707 | cg17175041 | cg17175041_BC21 | 0.8504536 |
| 708 | cg14282721 | cg14282721_BC21 | -0.495343 |
| 709 | cg23479360 | cg23479360_BC21 | 0.77883293 |
| 710 | cg24251135 | cg24251135_BC21 | 1.65220216 |
| 711 | cg24570302 | cg24570302_BC21 | 0.15846194 |
| 712 | cg07504739 | cg07504739_BC21 | 0.03515109 |
| 713 | cg07873320 | cg07873320_BC21 | -0.6319804 |
| 714 | cg19211853 | cg19211853_BC11 | 1.09189345 |
| 715 | cg03772108 | cg03772108_BC21 | 0.26973855 |
| 716 | cg24217140 | cg24217140_BC21 | 0.64358522 |
| 717 | cg21966910 | cg21966910_BC21 | -0.1864565 |
| 718 | cg16965794 | cg16965794_TC21 | 0.26743165 |
| 719 | cg11825941 | cg11825941_BC21 | -1.640379 |
| 720 | cg17110550 | cg17110550_BC21 | -0.7724788 |
| 721 | cg13723853 | cg13723853_BC11 | 2.4062304 |
| 722 | cg20971956 | cg20971956_BC21 | 1.07224163 |
| 723 | cg21614115 | cg21614115_TC21 | 0.49204071 |
| 724 | cg20204415 | cg20204415_TC11 | 0.63092847 |
| 725 | cg20107262 | cg20107262_BC21 | -0.0035366 |
| 726 | cg02059080 | cg02059080_BC21 | -0.0562817 |
| 727 | cg09530439 | cg09530439_BC21 | 1.05043797 |
| 728 | cg21844138 | cg21844138_BC21 | -0.8275987 |
| 729 | cg14768825 | cg14768825_BC21 | -1.9677629 |
| 730 | cg15228441 | cg15228441_BC21 | 0.72206674 |
| 731 | cg04789092 | cg04789092_BC21 | -0.0512845 |
| 732 | cg26185180 | cg26185180_BC21 | 0.67263513 |
| 733 | cg20205474 | cg20205474_BC21 | -0.0320602 |
| 734 | cg09224500 | cg09224500_TC21 | -2.089741 |
| 735 | cg05933240 | cg05933240_TC21 | -0.6618827 |
| 736 | cg15984175 | cg15984175_BC21 | 0.01523349 |
| 737 | cg25113157 | cg25113157_TC21 | 0.64847113 |
| 738 | cg21272956 | cg21272956_TC21 | 3.16602524 |
| 739 | cg07839071 | cg07839071_TC21 | 0.41535476 |
| 740 | cg06578851 | cg06578851_TC21 | 0.03796746 |
| 741 | cg04745278 | cg04745278_BC21 | 5.68820796 |
| 742 | cg18852137 | cg18852137_TC21 | 0.29000686 |
| 743 | cg19872010 | cg19872010_TC21 | -0.1903302 |
| 744 | cg00116658 | cg00116658_TC11 | 0.73085245 |
| 745 | cg00876449 | cg00876449_BC21 | -2.021506 |
| 746 | cg00216073 | cg00216073_BC21 | -0.392583 |
| 747 | cg00296348 | cg00296348_BC21 | -0.0324779 |
| 748 | cg23845380 | cg23845380_BC21 | 0.09290118 |
| 749 | cg00956987 | cg00956987_BC21 | 0.67906948 |
| 750 | cg27554535 | cg27554535_TC21 | 0.24121623 |
| 751 | cg07969684 | cg07969684_BC21 | 0.52820485 |
| 752 | cg16674566 | cg16674566_BC21 | -2.7068479 |
| 753 | cg20744229 | cg20744229_BC21 | -0.1971866 |
| 754 | cg12773508 | cg12773508_BC21 | -0.6891239 |
| 755 | cg05096492 | cg05096492_BC21 | -0.319901 |
| 756 | cg14587213 | cg14587213_TC21 | -1.559609 |
| 757 | cg23327859 | cg23327859_BC21 | 0.03105141 |
| 758 | cg11172565 | cg11172565_TC21 | -6.2869271 |
| 759 | cg08539737 | cg08539737_TC21 | 0.81122259 |
| 760 | cg23884551 | cg23884551_TC21 | 1.38048218 |
| 761 | cg04575953 | cg04575953_BC21 | 0.06473631 |
| 762 | cg11630939 | cg11630939_TC11 | -0.5257179 |
| 763 | cg16181537 | cg16181537_BC21 | -0.3108345 |
| 764 | cg04490037 | cg04490037_BC21 | 0.01439647 |
| 765 | cg22232492 | cg22232492_BC21 | 0.62990673 |
| 766 | cg03726640 | cg03726640_BC21 | -0.4921904 |
| 767 | cg00368338 | cg00368338_BC21 | -2.1622425 |
| 768 | cg02443129 | cg02443129_BC21 | 0.73545959 |
| 769 | cg03318469 | cg03318469_BC21 | 0.78067203 |
| 770 | cg24878059 | cg24878059_BC21 | 0.23302255 |
| 771 | cg16323641 | cg16323641_TC21 | -2.854388 |
| 772 | cg20071413 | cg20071413_TC21 | 0.06666439 |
| 773 | cg19541622 | cg19541622_TC21 | -0.6618612 |
| 774 | cg26263766 | cg26263766_BC11 | 0.70392601 |
| 775 | cg14131316 | cg14131316_BC21 | -3.6899193 |
| 776 | cg11197664 | cg11197664_BC21 | 1.22822051 |
| 777 | cg18082735 | cg18082735_BC21 | 0.32959032 |
| 778 | cg08487574 | cg08487574_TC21 | 1.16664273 |
| 779 | cg00769632 | cg00769632_BC21 | -0.1184212 |
| 780 | cg22986591 | cg22986591_BC21 | 0.59978617 |
| 781 | cg04410989 | cg04410989_TC21 | -1.0836212 |
| 782 | cg07949587 | cg07949587_TC21 | 0.63917292 |
| 783 | cg15401622 | cg15401622_TC21 | -3.3726685 |
| 784 | cg19501755 | cg19501755_TC21 | -3.8459052 |
| 785 | cg22354884 | cg22354884_TC21 | 0.3225978 |
| 786 | cg09586300 | cg09586300_TC21 | -1.0159161 |
| 787 | cg02580820 | cg02580820_TC21 | 0.02396197 |
| 788 | cg14807319 | cg14807319_BC21 | 0.28932379 |
| 789 | cg04502145 | cg04502145_BC21 | -0.2946659 |
| 790 | cg11049511 | cg11049511_BC21 | 1.35340589 |
| 791 | cg00595333 | cg00595333_TC21 | -0.1866377 |
| 792 | cg24512731 | cg24512731_BC11 | 1.16217711 |
| 793 | cg19288676 | cg19288676_BC21 | -0.0942078 |
| 794 | cg09126741 | cg09126741_BC21 | 0.9031416 |
| 795 | cg21993897 | cg21993897_BC21 | 0.20668127 |
| 796 | cg14921166 | cg14921166_BC21 | 0.40585853 |
| 797 | cg08914946 | cg08914946_BC21 | 0.92530387 |
| 798 | cg09903592 | cg09903592_TC21 | 2.07699883 |
| 799 | cg17091527 | cg17091527_BC21 | 1.47483057 |
| 800 | cg14254534 | cg14254534_BC21 | -2.9056119 |
| 801 | cg14195318 | cg14195318_TC21 | -0.8021713 |
| 802 | cg15210817 | cg15210817_BC21 | 0.01476117 |
| 803 | cg00792799 | cg00792799_BC21 | -0.922001 |
| 804 | cg16517790 | cg16517790_BC21 | -2.0493285 |
| 805 | cg08004926 | cg08004926_BC21 | 0.14385016 |
| 806 | cg16176432 | cg16176432_BC21 | 0.39239603 |
| 807 | cg27345757 | cg27345757_BC21 | 5.06202388 |
| 808 | cg06582394 | cg06582394_BC21 | 0.11623457 |
| 809 | cg27037944 | cg27037944_TC21 | 1.02541753 |
| 810 | cg09642347 | cg09642347_TC21 | 0.06309643 |
| 811 | cg00108108 | cg00108108_BC21 | -2.6768943 |
| 812 | cg16760899 | cg16760899_BC21 | 0.53131769 |
| 813 | cg25021026 | cg25021026_BC21 | -0.8166781 |
| 814 | cg14211332 | cg14211332_TC21 | 0.37736255 |
| 815 | cg11226932 | cg11226932_BC21 | 0.41753931 |
| 816 | cg17371184 | cg17371184_TC21 | -2.4967137 |
| 817 | cg27501458 | cg27501458_TC21 | 0.23547584 |
| 818 | cg02305856 | cg02305856_BC21 | -0.6781377 |
| 819 | cg22162164 | cg22162164_BC21 | -0.6965027 |
| 820 | cg01119957 | cg01119957_TC21 | 0.05098304 |
| 821 | cg02070677 | cg02070677_TC21 | -4.1295743 |
| 822 | cg18261667 | cg18261667_BC21 | -2.6457498 |
| 823 | cg20067719 | cg20067719_TC21 | -0.3504327 |
| 824 | cg20011601 | cg20011601_TC21 | -0.411536 |
| 825 | cg19124816 | cg19124816_BC21 | -2.1822964 |
| 826 | cg10611692 | cg10611692_BC21 | 2.51125448 |
| 827 | cg20457732 | cg20457732_BC11 | -0.3877409 |
| 828 | cg08097631 | cg08097631_BC21 | 0.22880692 |
| 829 | cg25909125 | cg25909125_TC21 | 1.96780844 |
| 830 | cg26631505 | cg26631505_TC21 | 0.02938426 |
| 831 | cg15643133 | cg15643133_TC21 | -0.4576512 |
| 832 | cg01964004 | cg01964004_BC21 | 0.93627974 |
| 833 | cg18537183 | cg18537183_BC21 | 0.35775269 |
| 834 | cg11423060 | cg11423060_TC21 | 0.60889808 |
| 835 | cg25511085 | cg25511085_BC21 | -0.4032861 |
| 836 | cg02540203 | cg02540203_BC21 | 0.01952425 |
| 837 | cg10765336 | cg10765336_TC21 | -0.0755363 |
| 838 | cg26829189 | cg26829189_TC21 | 0.01178929 |
| 839 | cg24641352 | cg24641352_BC21 | 1.83245254 |
| 840 | cg22460148 | cg22460148_BC21 | 0.09466761 |
| 841 | cg20672524 | cg20672524_TC21 | 0.12093493 |
| 842 | cg22421019 | cg22421019_BC21 | 0.05398908 |
| 843 | cg12596273 | cg12596273_TC21 | 0.07247242 |
| 844 | cg24438172 | cg24438172_BC21 | -0.6491451 |
| 845 | cg04176047 | cg04176047_TC21 | 1.08392613 |
| 846 | cg06594940 | cg06594940_TC21 | -1.0588425 |
| 847 | cg04071487 | cg04071487_BC21 | 0.01451278 |
| 848 | cg25471715 | cg25471715_BC11 | -0.2249886 |
| 849 | cg22769748 | cg22769748_TC21 | 1.76888157 |
| 850 | cg14523221 | cg14523221_BC21 | 0.28074067 |
| 851 | cg00085430 | cg00085430_TC21 | -0.0873867 |
| 852 | cg08255714 | cg08255714_BC21 | -1.347926 |
| 853 | cg07783477 | cg07783477_BC21 | -0.8895754 |
| 854 | cg25572037 | cg25572037_TC21 | -0.1467726 |
| 855 | cg22188885 | cg22188885_TC21 | 0.31639813 |
| 856 | cg00474031 | cg00474031_TC21 | 0.1475775 |
| 857 | cg25360231 | cg25360231_TC21 | 0.44199645 |
| 858 | cg16052327 | cg16052327_TC21 | 0.47300826 |
| 859 | cg27061049 | cg27061049_BC11 | -1.3730105 |
| 860 | cg19821846 | cg19821846_BC21 | 3.1119986 |
| 861 | cg02921845 | cg02921845_BC21 | 0.1819642 |
| 862 | cg18210656 | cg18210656_BC21 | -0.0102501 |
| 863 | cg22663098 | cg22663098_BC21 | 2.22735261 |
| 864 | cg09583780 | cg09583780_TC21 | 0.11246493 |
| 865 | cg06539497 | cg06539497_BC21 | 0.30686638 |
| 866 | cg01898222 | cg01898222_BC21 | 0.14528203 |
| 867 | cg24849910 | cg24849910_BC21 | -0.4901751 |
| 868 | cg22688447 | cg22688447_BC21 | -0.6990915 |
| 869 | cg23651117 | cg23651117_TC21 | -0.6172292 |
| 870 | cg14293223 | cg14293223_TC21 | -1.7139924 |
| 871 | cg12893271 | cg12893271_TC21 | -0.5749511 |
| 872 | cg08374617 | cg08374617_TC21 | -0.4979147 |
| 873 | cg12772987 | cg12772987_TC21 | 2.89963772 |
| 874 | cg15391813 | cg15391813_BC21 | 0.31007687 |
| 875 | cg17389400 | cg17389400_BC21 | 0.3295197 |
| 876 | cg15521034 | cg15521034_BC21 | 0.2560133 |
| 877 | cg01889025 | cg01889025_BC21 | 0.33398701 |
| 878 | cg11732853 | cg11732853_BC21 | 4.86386248 |
| 879 | cg25484306 | cg25484306_TC21 | -0.3012458 |
| 880 | cg13929658 | cg13929658_TC21 | -1.9670593 |
| 881 | cg26408927 | cg26408927_BC21 | 0.36388299 |
| 882 | cg14481315 | cg14481315_TC21 | -0.2657656 |
| 883 | cg03865524 | cg03865524_TC21 | -1.9402928 |
| 884 | cg07643616 | cg07643616_BC21 | 0.37826683 |
| 885 | cg04830416 | cg04830416_BC21 | -0.3340129 |
| 886 | cg21158280 | cg21158280_BC21 | 1.28505578 |
| 887 | cg15140236 | cg15140236_BC21 | 1.56482907 |
| 888 | cg20784841 | cg20784841_TC21 | -0.0907789 |
| 889 | cg13119578 | cg13119578_BC21 | -0.7603819 |
| 890 | cg14647543 | cg14647543_BC21 | 0.70607335 |
| 891 | cg22598841 | cg22598841_TC21 | 6.26988631 |
| 892 | cg07138104 | cg07138104_TC21 | 0.10886799 |
| 893 | cg12286415 | cg12286415_BC21 | 0.11188876 |
| 894 | cg11036058 | cg11036058_TC21 | -0.6366255 |
| 895 | cg21524899 | cg21524899_TC21 | -5.2585081 |
| 896 | cg07707056 | cg07707056_TC21 | 0.56862436 |
| 897 | cg22257107 | cg22257107_BC21 | -2.2680633 |
| 898 | cg13183732 | cg13183732_TC21 | 0.5089705 |
| 899 | cg22277154 | cg22277154_TC21 | -1.8001407 |
| 900 | cg22602379 | cg22602379_BC21 | 0.96698593 |
| 901 | cg11177698 | cg11177698_BC21 | 0.87147321 |
| 902 | cg05357229 | cg05357229_BC21 | -0.2073148 |
| 903 | cg19821872 | cg19821872_BC21 | 2.24346071 |
| 904 | cg16740586 | cg16740586_TC21 | 2.75319849 |
| 905 | cg20711299 | cg20711299_TC21 | -0.2943497 |
| 906 | cg10312464 | cg10312464_TC21 | 0.5284604 |
| 907 | cg01506627 | cg01506627_TC21 | 0.23488261 |
| 908 | cg02555241 | cg02555241_TC21 | -0.4717287 |
| 909 | cg01306003 | cg01306003_TC21 | 1.41763743 |
| 910 | cg06292004 | cg06292004_BC21 | 0.44030656 |
| 911 | cg10933529 | cg10933529_BC21 | -0.0994099 |
| 912 | cg18087395 | cg18087395_BC21 | 0.19885151 |
| 913 | cg10514640 | cg10514640_TC21 | 0.340587 |
| 914 | cg03631509 | cg03631509_BC21 | 1.50496376 |
| 915 | cg02724496 | cg02724496_TC21 | -2.336927 |
| 916 | cg01705888 | cg01705888_BC21 | 0.51057915 |
| 917 | cg04781978 | cg04781978_BC21 | 0.80559459 |
| 918 | cg15102821 | cg15102821_TC21 | -0.5748061 |
| 919 | cg01606057 | cg01606057_BC21 | 0.34290227 |
| 920 | cg21141726 | cg21141726_BC21 | -2.9643852 |
| 921 | cg11823142 | cg11823142_TC11 | -0.3076466 |
| 922 | cg16688434 | cg16688434_BC11 | 6.78620343 |
| 923 | cg05421487 | cg05421487_TC21 | -0.2743326 |
| 924 | cg09317437 | cg09317437_BC21 | 0.5968243 |
| 925 | cg13774304 | cg13774304_BC21 | 1.58145588 |
| 926 | cg27211071 | cg27211071_BC21 | 0.02256004 |
| 927 | cg07154414 | cg07154414_BC21 | -0.5207927 |
| 928 | cg17065725 | cg17065725_BC21 | 0.75143455 |
| 929 | cg17389212 | cg17389212_BC21 | 0.09331676 |
| 930 | cg24504388 | cg24504388_BC21 | 0.08297737 |
| 931 | cg10860275 | cg10860275_TC21 | -0.3524727 |
| 932 | cg19402756 | cg19402756_TC21 | 0.02584485 |
| 933 | cg14253018 | cg14253018_BC21 | -0.3204067 |
| 934 | cg04788392 | cg04788392_BC21 | 0.00679138 |
| 935 | cg15547413 | cg15547413_TC21 | 0.01088185 |
| 936 | cg10324245 | cg10324245_TC21 | -0.0241681 |
| 937 | cg10395850 | cg10395850_BC21 | -0.0149931 |
| 938 | cg17855598 | cg17855598_BC21 | -1.4457262 |
| 939 | cg11491284 | cg11491284_BC21 | -4.4183592 |
| 940 | cg24738628 | cg24738628_BC21 | 0.68544443 |
| 941 | cg04395846 | cg04395846_BC21 | -0.3205092 |
| 942 | cg10710176 | cg10710176_BC21 | 0.02704985 |
| 943 | cg19566482 | cg19566482_TC21 | 0.02061507 |
| 944 | cg21565937 | cg21565937_TC21 | 0.01696649 |
| 945 | cg19151852 | cg19151852_BC21 | 3.41304259 |
| 946 | cg21009965 | cg21009965_TC21 | 0.46933478 |
| 947 | cg22455892 | cg22455892_TC11 | -2.8512172 |
| 948 | cg05009902 | cg05009902_BC21 | 1.27764804 |
| 949 | cg18867200 | cg18867200_BC11 | 2.24452789 |
| 950 | cg05178839 | cg05178839_BC21 | -1.0261473 |
| 951 | cg25882544 | cg25882544_TC21 | 0.28073028 |
| 952 | cg26695190 | cg26695190_TC21 | -3.9181443 |
| 953 | cg19509830 | cg19509830_TC21 | -0.3295517 |
| 954 | cg03967756 | cg03967756_BC21 | -0.0850384 |
| 955 | cg26505330 | cg26505330_BC21 | -0.3755524 |
| 956 | cg14087697 | cg14087697_TC21 | -0.3662985 |
| 957 | cg22026677 | cg22026677_BC21 | 1.25546901 |
| 958 | cg06775471 | cg06775471_BC21 | 1.8665693 |
| 959 | cg22512239 | cg22512239_TC21 | -0.1430669 |
| 960 | cg18963953 | cg18963953_TC21 | 1.49733991 |
| 961 | cg01095227 | cg01095227_BC21 | 0.31335295 |
| 962 | cg03810165 | cg03810165_BC21 | 2.35054924 |
| 963 | cg20979800 | cg20979800_TC21 | 0.36892047 |
| 964 | cg02889942 | cg02889942_BC21 | 0.35481645 |
| 965 | cg20385959 | cg20385959_TC21 | 1.45951881 |
| 966 | cg10680608 | cg10680608_BC21 | -0.021659 |
| 967 | cg10700683 | cg10700683_TC21 | 0.55742872 |
| 968 | cg16043669 | cg16043669_BC21 | 1.18299458 |
| 969 | cg17481116 | cg17481116_BC21 | 1.05298909 |
| 970 | cg18487916 | cg18487916_TC21 | 0.3088641 |
| 971 | cg14172375 | cg14172375_TC21 | -1.0460386 |
| 972 | cg17550929 | cg17550929_TC21 | -1.1850322 |
| 973 | cg26994023 | cg26994023_TC21 | 0.23097411 |
| 974 | cg15915241 | cg15915241_TC21 | -0.1037323 |
| 975 | cg21660413 | cg21660413_BC21 | -0.0600065 |
| 976 | cg08334751 | cg08334751_BC21 | 0.18767342 |
| 977 | cg11422009 | cg11422009_TC21 | 2.17109946 |
| 978 | cg20542800 | cg20542800_BC21 | -1.6828882 |
| 979 | cg11251135 | cg11251135_BC21 | 0.00737029 |
| 980 | cg04464263 | cg04464263_BC21 | 1.22638332 |
| 981 | cg19193136 | cg19193136_TC21 | -0.4232088 |
| 982 | cg20361421 | cg20361421_TC21 | 1.6178611 |
| 983 | cg02489108 | cg02489108_BC21 | 0.69099471 |
| 984 | cg15545942 | cg15545942_TC21 | 1.10416122 |
| 985 | cg09677785 | cg09677785_BC21 | 0.11230312 |
| 986 | cg14118949 | cg14118949_BC21 | -0.1325604 |
| 987 | cg27426983 | cg27426983_TC21 | -1.9033675 |
| 988 | cg25490693 | cg25490693_TC21 | 0.36976219 |
| 989 | cg13785339 | cg13785339_BC21 | 1.11574293 |
| 990 | cg09589646 | cg09589646_BC21 | -0.2990955 |
| 991 | cg04085767 | cg04085767_TC21 | 0.10464594 |
| 992 | cg10427268 | cg10427268_BC21 | -1.5933097 |
| 993 | cg21066703 | cg21066703_BC21 | -1.4226605 |
| 994 | cg20062413 | cg20062413_TC21 | -1.8281182 |
| 995 | cg26632665 | cg26632665_BC21 | 0.07746744 |
| 996 | cg22526407 | cg22526407_TC21 | 1.0219264 |
| 997 | cg18007737 | cg18007737_TC21 | 1.46195164 |
| 998 | cg24455365 | cg24455365_BC11 | -0.6034419 |
| 999 | cg03421353 | cg03421353_BC21 | 0.71977335 |
| 1000 | cg19292712 | cg19292712_BC21 | 0.41379214 |
| 1001 | cg18513737 | cg18513737_BC21 | 0.00432439 |
| 1002 | cg08411376 | cg08411376_BC21 | 0.27880251 |
| 1003 | cg10523672 | cg10523672_BC21 | 0.48971749 |
| 1004 | cg13265524 | cg13265524_BC21 | 0.66147486 |
| 1005 | cg24465349 | cg24465349_TC21 | -0.9784435 |
| 1006 | cg06963130 | cg06963130_BC21 | -3.3699872 |
| 1007 | cg19767020 | cg19767020_TC21 | 0.74435849 |
| 1008 | cg00047050 | cg00047050_TC21 | -2.2324682 |
| 1009 | ch.1.839062R | ch.1.839062R_BC21 | -3.4149753 |
| 1010 | cg17081586 | cg17081586_BC21 | 3.29195275 |
| 1011 | cg08374428 | cg08374428_BC21 | 1.40698705 |
| 1012 | cg16867820 | cg16867820_TC21 | 0.18520497 |
| 1013 | cg21232822 | cg21232822_TC21 | 1.397183 |
| 1014 | cg11365584 | cg11365584_BC21 | -0.4851384 |
| 1015 | cg00753676 | cg00753676_TC21 | -0.2852844 |
| 1016 | cg00363845 | cg00363845_BC21 | 0.12592095 |
| 1017 | cg00741848 | cg00741848_BC21 | 0.1434836 |
| 1018 | cg17349007 | cg17349007_TC21 | 0.4311722 |
| 1019 | cg21244504 | cg21244504_BC21 | 0.23059952 |
| 1020 | cg00412782 | cg00412782_BC21 | 0.53618126 |
| 1021 | cg02159111 | cg02159111_BC21 | 1.20121516 |
| 1022 | cg00594335 | cg00594335_TC21 | 4.9545858 |
| 1023 | cg23289456 | cg23289456_TC21 | 0.05443114 |
| 1024 | cg00077044 | cg00077044_TC21 | 1.79036508 |
| 1025 | cg23062771 | cg23062771_BC21 | 0.25560158 |
| 1026 | cg06378981 | cg06378981_BC21 | 1.31496107 |
| 1027 | cg08578520 | cg08578520_BC21 | -0.9810839 |
| 1028 | cg25841008 | cg25841008_BC21 | 1.13156418 |
| 1029 | cg01060238 | cg01060238_BC21 | 0.24774618 |
| 1030 | cg20407119 | cg20407119_BC21 | 0.17421519 |
| 1031 | cg23788054 | cg23788054_BC21 | -2.3971731 |
| 1032 | cg10757964 | cg10757964_TC21 | 0.68282434 |
| 1033 | cg18810681 | cg18810681_BC21 | 0.17575247 |
| 1034 | cg14228710 | cg14228710_TC21 | 0.59017851 |
| 1035 | cg08822136 | cg08822136_TC21 | 0.03943159 |
| 1036 | cg12597822 | cg12597822_BC21 | -1.2522728 |
| 1037 | cg02922871 | cg02922871_TC21 | 0.3781063 |
| 1038 | cg03783393 | cg03783393_BC21 | 4.35252778 |
| 1039 | cg11584042 | cg11584042_BC21 | -4.8586083 |
| 1040 | cg13008698 | cg13008698_BC21 | -1.7839728 |
| 1041 | cg17847095 | cg17847095_BC21 | 3.54745939 |
| 1042 | cg21248322 | cg21248322_TC21 | 0.04017032 |
| 1043 | cg16732475 | cg16732475_TC21 | -0.8702322 |
| 1044 | cg23764836 | cg23764836_BC21 | -0.1176076 |
| 1045 | cg19980958 | cg19980958_TC21 | -2.3900426 |
| 1046 | cg00417068 | cg00417068_BC21 | 1.44853134 |
| 1047 | cg13469617 | cg13469617_BC21 | 0.73922334 |
| 1048 | cg20343278 | cg20343278_BC21 | -0.2860712 |
| 1049 | cg15926234 | cg15926234_BC21 | 0.02323916 |
| 1050 | cg03778809 | cg03778809_BC21 | 1.16270827 |
| 1051 | cg22729791 | cg22729791_BC21 | -0.4040014 |
| 1052 | cg00169030 | cg00169030_BC21 | 0.05081664 |
| 1053 | cg04214706 | cg04214706_BC21 | -0.9862854 |
| 1054 | cg27207809 | cg27207809_TC21 | -0.6203301 |
| 1055 | cg13062104 | cg13062104_BC21 | 0.34968186 |
| 1056 | cg26409826 | cg26409826_BC21 | 0.09788968 |
| 1057 | cg20158040 | cg20158040_BC21 | 0.54591425 |
| 1058 | cg14183865 | cg14183865_TC21 | -0.3532409 |
| 1059 | cg00042904 | cg00042904_BC21 | -2.0156602 |
| 1060 | cg20608259 | cg20608259_BC21 | -0.4541803 |
| 1061 | cg05780020 | cg05780020_TC21 | -0.8113989 |
| 1062 | cg19381797 | cg19381797_TC21 | 1.24509773 |
| 1063 | cg09116068 | cg09116068_BC21 | 1.39578309 |
| 1064 | cg05746865 | cg05746865_BC21 | 0.75388282 |
| 1065 | cg18346693 | cg18346693_TC21 | 1.73155472 |
| 1066 | cg16974046 | cg16974046_BC21 | -0.3859203 |
| 1067 | cg18360809 | cg18360809_TC21 | -0.2798203 |
| 1068 | cg15326741 | cg15326741_TC21 | 0.42804978 |
| 1069 | cg22513912 | cg22513912_TC21 | -2.4059028 |
| 1070 | cg08174179 | cg08174179_TC21 | 0.45322834 |
| 1071 | cg07128371 | cg07128371_BC21 | -1.4971968 |
| 1072 | cg05753416 | cg05753416_BC21 | -7.0306515 |
| 1073 | cg20786995 | cg20786995_BC11 | -0.1440179 |
| 1074 | cg16284279 | cg16284279_BC21 | 0.22358327 |
| 1075 | cg09164913 | cg09164913_BC21 | -0.8844003 |
| 1076 | cg15282616 | cg15282616_BC21 | 3.28661539 |
| 1077 | cg13308744 | cg13308744_TC21 | -0.3412317 |
| 1078 | cg11677147 | cg11677147_BC21 | 0.25819711 |
| 1079 | cg05431218 | cg05431218_TC21 | 0.20006801 |
| 1080 | cg03511588 | cg03511588_TC21 | -0.5717198 |
| 1081 | cg09016994 | cg09016994_TC21 | 0.00488683 |
| 1082 | cg13959371 | cg13959371_BC21 | -0.9094136 |
| 1083 | cg05773708 | cg05773708_TC21 | -0.4393644 |
| 1084 | cg21268653 | cg21268653_BC21 | -0.7863019 |
| 1085 | cg03078743 | cg03078743_BC21 | -2.0509267 |
| 1086 | cg23114828 | cg23114828_TC21 | 0.22679823 |
| 1087 | cg14214635 | cg14214635_TC11 | -2.2743896 |
| 1088 | cg09071007 | cg09071007_TC21 | 1.5024244 |
| 1089 | cg14129356 | cg14129356_TC21 | -1.406258 |
| 1090 | cg04452203 | cg04452203_BC21 | 1.30139614 |
| 1091 | cg16530946 | cg16530946_TC21 | 0.33985851 |
| 1092 | cg11176587 | cg11176587_BC21 | -3.1820533 |
| 1093 | cg07051684 | cg07051684_BC21 | 0.0127272 |
| 1094 | cg05865665 | cg05865665_BC21 | 0.16932059 |
| 1095 | cg19644580 | cg19644580_TC21 | -3.2987434 |
| 1096 | cg02624377 | cg02624377_TC21 | -3.7444094 |
| 1097 | cg18303589 | cg18303589_BC21 | -0.0844532 |
| 1098 | cg09241427 | cg09241427_BC21 | 0.08404693 |
| 1099 | cg13962846 | cg13962846_BC21 | 0.32086728 |
| 1100 | cg27613026 | cg27613026_BC21 | -0.4672159 |
| 1101 | cg04175911 | cg04175911_BC21 | -2.1394665 |
| 1102 | cg07459645 | cg07459645_TC21 | 0.41260175 |
| 1103 | cg26213240 | cg26213240_BC21 | 0.63395817 |
| 1104 | cg11116138 | cg11116138_TC21 | 0.19939139 |
| 1105 | cg11408259 | cg11408259_BC21 | 0.27320756 |
| 1106 | cg02290284 | cg02290284_BC21 | 1.03768949 |
| 1107 | cg14210337 | cg14210337_BC21 | 0.55470064 |
| 1108 | cg27024214 | cg27024214_TC21 | 0.89554597 |
| 1109 | cg14775730 | cg14775730_TC21 | 0.91316869 |
| 1110 | cg16992985 | cg16992985_BC21 | 0.05655531 |
| 1111 | cg12972086 | cg12972086_TC21 | 0.51821381 |
| 1112 | cg26142076 | cg26142076_BC21 | -0.1872882 |
| 1113 | cg11546131 | cg11546131_TC21 | 1.27976243 |
| 1114 | cg06159131 | cg06159131_BC21 | -0.7248486 |
| 1115 | cg09983746 | cg09983746_TC21 | -0.233241 |
| 1116 | cg23623781 | cg23623781_BC21 | 1.25829967 |
| 1117 | cg18993113 | cg18993113_TC21 | 0.5687755 |
| 1118 | cg13436782 | cg13436782_BC21 | 2.16052761 |
| 1119 | cg21449851 | cg21449851_BC21 | -0.0685268 |
| 1120 | cg00986928 | cg00986928_BC21 | -0.0138202 |
| 1121 | cg03454094 | cg03454094_BC21 | -6.8948689 |
| 1122 | cg13230187 | cg13230187_TC21 | -1.3021795 |
| 1123 | cg12595444 | cg12595444_TC21 | 0.06820647 |
| 1124 | cg04145937 | cg04145937_TC21 | -0.5806376 |
| 1125 | cg02737288 | cg02737288_BC21 | -0.2465312 |
| 1126 | cg06672696 | cg06672696_BC21 | 1.75160034 |
| 1127 | cg04700055 | cg04700055_TC21 | 0.72432346 |
| 1128 | cg20767370 | cg20767370_BC21 | -0.4793779 |
| 1129 | cg00565561 | cg00565561_BC21 | 0.06234025 |
| 1130 | cg18187918 | cg18187918_BC21 | 0.03987479 |
| 1131 | cg19847191 | cg19847191_TC21 | -2.3121 |
| 1132 | cg11841964 | cg11841964_BC21 | 0.03872086 |
| 1133 | cg21587777 | cg21587777_TC21 | -6.1275502 |
| 1134 | cg03275375 | cg03275375_TC21 | -0.0596552 |
| 1135 | cg15795081 | cg15795081_TC21 | 0.86872888 |
| 1136 | cg15300348 | cg15300348_TC21 | 0.90915284 |
| 1137 | cg25914821 | cg25914821_TC21 | -3.5297069 |
| 1138 | cg17744931 | cg17744931_TC21 | -1.2962686 |
| 1139 | cg12114963 | cg12114963_BC21 | 0.44497436 |
| 1140 | cg24485951 | cg24485951_BC21 | 0.96517803 |
| 1141 | cg13070212 | cg13070212_TC21 | 1.74676264 |
| 1142 | cg10798225 | cg10798225_BC21 | -0.5270375 |
| 1143 | cg25139740 | cg25139740_TC21 | -0.1827925 |
| 1144 | cg10953594 | cg10953594_BC21 | -0.3731219 |
| 1145 | cg23060323 | cg23060323_BC21 | -5.8641923 |
| 1146 | cg15931375 | cg15931375_BC21 | -3.3717819 |
| 1147 | cg00785980 | cg00785980_TC21 | 3.03072078 |
| 1148 | cg00264112 | cg00264112_TC21 | 0.08036265 |
| 1149 | cg14916175 | cg14916175_BC21 | -0.7149844 |
| 1150 | cg07465864 | cg07465864_TC21 | 1.61084753 |
| 1151 | cg05624388 | cg05624388_BC21 | 0.4873617 |
| 1152 | cg00941280 | cg00941280_TC21 | -0.7494588 |
| 1153 | cg00992268 | cg00992268_TC21 | -2.1607527 |
| 1154 | cg24187909 | cg24187909_BC21 | -0.5149982 |
| 1155 | cg01601198 | cg01601198_BC21 | 0.12554097 |
| 1156 | cg04384810 | cg04384810_BC21 | 1.4755365 |
| 1157 | cg16120485 | cg16120485_BC21 | 0.25967909 |
| 1158 | cg14659082 | cg14659082_TC21 | 0.24023903 |
| 1159 | cg22895815 | cg22895815_TC21 | 0.0925401 |
| 1160 | cg27154640 | cg27154640_TC21 | -0.0831577 |
| 1161 | cg06630416 | cg06630416_BC21 | -0.9665015 |
| 1162 | cg18230220 | cg18230220_TC21 | -2.4284932 |
| 1163 | cg27503801 | cg27503801_BC21 | -5.0733154 |
| 1164 | cg15994304 | cg15994304_TC21 | -0.0308713 |
| 1165 | cg04866800 | cg04866800_BC21 | -0.0077207 |
| 1166 | cg17239194 | cg17239194_TC21 | 1.21694625 |
| 1167 | cg21035990 | cg21035990_TC21 | 0.03008798 |
| 1168 | cg02683869 | cg02683869_BC21 | 0.24522749 |
| 1169 | cg11784435 | cg11784435_BC21 | 0.43520513 |
| 1170 | cg15916194 | cg15916194_BC21 | 0.68426701 |
| 1171 | cg11095714 | cg11095714_BC21 | -0.3455344 |
| 1172 | cg06978060 | cg06978060_TC21 | -0.6287911 |
| 1173 | cg23973576 | cg23973576_TC11 | -0.3297913 |
| 1174 | cg15170994 | cg15170994_BC21 | 0.00136884 |
| 1175 | cg17472686 | cg17472686_BC21 | -6.197249 |
| 1176 | cg03316614 | cg03316614_BC21 | -0.2034877 |
| 1177 | cg19323862 | cg19323862_TC21 | 0.12550537 |
| 1178 | cg16678452 | cg16678452_BC21 | 0.02110227 |
| 1179 | cg16936289 | cg16936289_BC21 | 0.46938151 |
| 1180 | cg13698411 | cg13698411_TC21 | -1.1748124 |
| 1181 | cg00541293 | cg00541293_BC21 | -0.5882252 |
| 1182 | cg09830575 | cg09830575_BC21 | 0.45584137 |
| 1183 | cg07762599 | cg07762599_BC21 | -0.7499605 |
| 1184 | cg14216263 | cg14216263_TC21 | 0.97261515 |
| 1185 | cg09497789 | cg09497789_BC21 | 0.5695292 |
| 1186 | cg25260543 | cg25260543_TC21 | 1.79188346 |
| 1187 | cg14217660 | cg14217660_TC21 | 1.097058 |
| 1188 | cg19204328 | cg19204328_TC21 | -0.7476467 |
| 1189 | cg20217978 | cg20217978_TC21 | 0.22075027 |
| 1190 | cg23883522 | cg23883522_TC21 | -0.5396111 |
| 1191 | cg23126977 | cg23126977_BC21 | -0.8722613 |
| 1192 | cg15240652 | cg15240652_TC21 | -0.4172544 |
| 1193 | cg01092133 | cg01092133_TC21 | 0.14321218 |
| 1194 | cg11651896 | cg11651896_BC21 | 2.98147419 |
| 1195 | cg27450557 | cg27450557_TC21 | -0.2147172 |
| 1196 | cg02372905 | cg02372905_BC21 | 0.11890226 |
| 1197 | cg08852244 | cg08852244_BC21 | 0.32938445 |
| 1198 | cg27112487 | cg27112487_BC21 | 1.08942855 |
| 1199 | cg09169591 | cg09169591_BC21 | -2.7005819 |
| 1200 | cg13511490 | cg13511490_BC21 | -0.0774316 |
| 1201 | cg10520964 | cg10520964_TC21 | 0.20742934 |
| 1202 | cg13366903 | cg13366903_TC21 | 0.13979251 |
| 1203 | cg22825261 | cg22825261_BC21 | 0.01679656 |
| 1204 | cg18845973 | cg18845973_TC21 | 0.7614251 |
| 1205 | cg02713563 | cg02713563_BC21 | 0.42773686 |
| 1206 | cg01999608 | cg01999608_TC21 | -4.814954 |
| 1207 | cg06478823 | cg06478823_TC21 | -1.4119773 |
| 1208 | cg17979719 | cg17979719_BC21 | 0.31343475 |
| 1209 | cg09149437 | cg09149437_TC21 | -0.5663892 |
| 1210 | cg06563667 | cg06563667_BC21 | -2.5506317 |
| 1211 | cg16080273 | cg16080273_BC21 | -1.1798922 |
| 1212 | cg23231456 | cg23231456_BC21 | -0.0937034 |
| 1213 | cg03800252 | cg03800252_TC21 | 0.04981959 |
| 1214 | cg27216853 | cg27216853_TC21 | 0.54769618 |
| 1215 | cg23902765 | cg23902765_BC21 | 0.04353702 |
| 1216 | cg07186767 | cg07186767_BC21 | -0.177459 |
| 1217 | cg00913794 | cg00913794_BC21 | 0.77846447 |
| 1218 | cg06128194 | cg06128194_BC21 | 0.0098902 |
| 1219 | cg08191886 | cg08191886_BC21 | -2.8303829 |
| 1220 | cg14317639 | cg14317639_BC21 | 1.62485555 |
| 1221 | cg16003004 | cg16003004_TC21 | 0.55511814 |
| 1222 | cg01744005 | cg01744005_TC21 | 0.40342377 |
| 1223 | cg08180188 | cg08180188_BC21 | 0.43319896 |
| 1224 | cg14320320 | cg14320320_BC21 | 0.46839465 |
| 1225 | cg05165556 | cg05165556_BC21 | 0.96243896 |
| 1226 | cg09780594 | cg09780594_TC21 | -0.0712148 |
| 1227 | cg08188889 | cg08188889_BC21 | 0.84517213 |
| 1228 | cg25227378 | cg25227378_TC21 | 1.72474947 |
| 1229 | cg25300780 | cg25300780_BC21 | -1.8310928 |
| 1230 | cg00650410 | cg00650410_BC21 | 0.05755245 |
| 1231 | cg04821933 | cg04821933_TC21 | -0.152773 |
| 1232 | cg02138813 | cg02138813_BC21 | -1.0737378 |
| 1233 | cg12040280 | cg12040280_BC21 | 0.06065451 |
| 1234 | cg22079827 | cg22079827_BC21 | -0.7293414 |
| 1235 | cg25433915 | cg25433915_TC21 | -0.6303026 |
| 1236 | cg01720533 | cg01720533_BC21 | 2.50929941 |
| 1237 | cg21733756 | cg21733756_BC21 | 0.35935963 |
| 1238 | cg22780540 | cg22780540_BC21 | 0.02888198 |
| 1239 | cg05883128 | cg05883128_BC21 | 2.57509285 |
| 1240 | cg16535390 | cg16535390_BC21 | 0.272553 |
| 1241 | cg09737019 | cg09737019_BC21 | 0.34130429 |
| 1242 | cg03231735 | cg03231735_BC21 | 2.31725992 |
| 1243 | cg23525360 | cg23525360_BC21 | 0.14989802 |
| 1244 | cg05288075 | cg05288075_BC21 | -0.3056008 |
| 1245 | cg15736994 | cg15736994_TC21 | -2.4371766 |
| 1246 | cg03604842 | cg03604842_BC21 | 0.38104125 |
| 1247 | cg22787530 | cg22787530_BC21 | 0.08971413 |
| 1248 | cg02859934 | cg02859934_TC21 | 0.81625889 |
| 1249 | cg12921720 | cg12921720_TC21 | 0.41043239 |
| 1250 | cg18902238 | cg18902238_BC21 | -2.0667376 |
| 1251 | cg17177602 | cg17177602_BC21 | 1.10222399 |
| 1252 | cg18248145 | cg18248145_BC21 | 0.05577138 |
| 1253 | cg23084415 | cg23084415_BC21 | -0.7626288 |
| 1254 | cg22529504 | cg22529504_BC21 | 0.35549867 |
| 1255 | cg24722577 | cg24722577_TC21 | 0.01380668 |
| 1256 | cg11410857 | cg11410857_BC21 | 2.50875759 |
| 1257 | cg02441694 | cg02441694_BC21 | 2.37044962 |
| 1258 | cg26828941 | cg26828941_BC21 | 0.23146712 |
| 1259 | cg16656322 | cg16656322_BC21 | -0.7726552 |
| 1260 | cg02517189 | cg02517189_TC21 | -2.7873265 |
| 1261 | cg12626242 | cg12626242_BC21 | -0.0624403 |
| 1262 | cg00701995 | cg00701995_TC21 | 0.87554185 |
| 1263 | cg13307649 | cg13307649_BC21 | 0.4973362 |
| 1264 | cg12318914 | cg12318914_BC21 | -4.2171563 |
| 1265 | cg18157642 | cg18157642_TC21 | 0.2937113 |
| 1266 | cg24601055 | cg24601055_BC21 | 0.00073652 |
| 1267 | cg20789819 | cg20789819_BC21 | -2.3425519 |
| 1268 | cg08679405 | cg08679405_BC21 | 2.33476215 |
| 1269 | cg01415148 | cg01415148_TC21 | -0.1952978 |
| 1270 | cg00776293 | cg00776293_TC21 | -1.0431947 |
| 1271 | cg18068929 | cg18068929_TC21 | 3.01135549 |
| 1272 | cg02447257 | cg02447257_BC21 | -4.9399345 |
| 1273 | cg09686990 | cg09686990_BC21 | -0.4737092 |
| 1274 | cg06073803 | cg06073803_TC21 | -0.0595977 |
| 1275 | cg17560073 | cg17560073_BC21 | 2.53409872 |
| 1276 | cg24330542 | cg24330542_BC21 | 1.95142972 |
| 1277 | cg15207883 | cg15207883_TC21 | -0.1592636 |
| 1278 | cg22706806 | cg22706806_BC21 | 1.12790861 |
| 1279 | cg10426318 | cg10426318_TC21 | -1.3597483 |
| 1280 | cg17086542 | cg17086542_BC21 | 0.65273433 |
| 1281 | cg22681246 | cg22681246_TC21 | -0.5036745 |
| 1282 | cg02987466 | cg02987466_TC21 | 0.96427213 |
| 1283 | cg22361181 | cg22361181_BC21 | -3.509968 |
| 1284 | cg05249016 | cg05249016_BC21 | -0.9763466 |
| 1285 | cg15833449 | cg15833449_BC21 | -0.0572071 |
| 1286 | cg20890687 | cg20890687_TC21 | 1.60071334 |
| 1287 | cg03069764 | cg03069764_BC21 | -0.1013782 |
| 1288 | cg17185710 | cg17185710_BC21 | -1.7893251 |
| 1289 | cg27489916 | cg27489916_BC21 | 0.14328202 |
| 1290 | cg03572680 | cg03572680_TC21 | -0.0262094 |
| 1291 | cg16182491 | cg16182491_TC21 | 0.65490238 |
| 1292 | cg18570126 | cg18570126_BC21 | -1.5331946 |
| 1293 | cg01947787 | cg01947787_BC21 | 1.34704998 |
| 1294 | cg00362690 | cg00362690_BC21 | 0.196067 |
| 1295 | cg18683711 | cg18683711_TC21 | -1.8476238 |
| 1296 | cg00688775 | cg00688775_BC21 | -0.2735099 |
| 1297 | cg09115158 | cg09115158_TC21 | 0.52373703 |
| 1298 | cg06005877 | cg06005877_TC21 | 0.88514216 |
| 1299 | cg22697223 | cg22697223_TC21 | 0.23330216 |
| 1300 | cg16750433 | cg16750433_BC21 | 0.28717117 |
| 1301 | cg05645141 | cg05645141_BC21 | 1.0485626 |
| 1302 | cg04081402 | cg04081402_TC21 | -3.4677449 |
| 1303 | cg09283959 | cg09283959_BC21 | 1.47944021 |
| 1304 | cg25506095 | cg25506095_BC21 | -0.6476242 |
| 1305 | cg26906737 | cg26906737_TC21 | 2.67150084 |
| 1306 | cg03646542 | cg03646542_BC21 | -0.4390093 |
| 1307 | cg26318926 | cg26318926_BC21 | -0.1247052 |
| 1308 | cg02441556 | cg02441556_BC21 | -0.3903418 |
| 1309 | cg27425655 | cg27425655_BC21 | -0.3792223 |
| 1310 | cg00846843 | cg00846843_BC21 | 0.34253973 |
| 1311 | cg09665331 | cg09665331_BC21 | 0.03837827 |
| 1312 | cg23990781 | cg23990781_TC21 | -0.0915236 |
| 1313 | cg24307703 | cg24307703_TC21 | -2.7967603 |
| 1314 | cg09623056 | cg09623056_BC21 | 0.29166333 |
| 1315 | cg20748132 | cg20748132_BC21 | -0.2839379 |
| 1316 | cg06986963 | cg06986963_TC21 | 0.73409788 |
| 1317 | cg21566071 | cg21566071_BC21 | -0.0403648 |
| 1318 | cg17274080 | cg17274080_BC21 | 0.0564653 |
| 1319 | cg00683285 | cg00683285_TC21 | 1.34558806 |
| 1320 | cg27578275 | cg27578275_BC21 | 0.08960962 |
| 1321 | cg03100063 | cg03100063_BC21 | 0.4201798 |
| 1322 | cg16359525 | cg16359525_BC21 | 0.34517061 |
| 1323 | cg24740322 | cg24740322_BC21 | 0.12758706 |
| 1324 | cg20912542 | cg20912542_BC21 | -0.8711312 |
| 1325 | cg15592828 | cg15592828_TC21 | -2.2863782 |
| 1326 | cg16040935 | cg16040935_BC21 | -0.541771 |
| 1327 | cg11664903 | cg11664903_BC21 | 0.3942641 |
| 1328 | cg27640528 | cg27640528_TC21 | -0.7891112 |
| 1329 | cg12883486 | cg12883486_BC21 | 2.11829328 |
| 1330 | cg27658402 | cg27658402_BC21 | 0.50538879 |
| 1331 | cg05517106 | cg05517106_BC21 | -0.0550511 |
| 1332 | cg12038352 | cg12038352_TC21 | 0.54215835 |
| 1333 | cg18810022 | cg18810022_BC21 | 1.84506913 |
| 1334 | cg11786320 | cg11786320_BC21 | 0.53065433 |
| 1335 | cg22006489 | cg22006489_TC21 | -1.9973252 |
| 1336 | cg07986773 | cg07986773_TC21 | 1.54800221 |
| 1337 | cg02375669 | cg02375669_TC21 | -0.2125485 |
| 1338 | cg04713102 | cg04713102_BC21 | 0.42719631 |
| 1339 | cg14742901 | cg14742901_BC21 | -0.7480404 |
| 1340 | cg19076536 | cg19076536_TC21 | -3.6830886 |
| 1341 | cg22798948 | cg22798948_BC21 | -4.4607443 |
| 1342 | cg25695116 | cg25695116_BC21 | -0.5383029 |
| 1343 | cg25866895 | cg25866895_TC21 | -0.4742007 |
| 1344 | cg08139264 | cg08139264_BC21 | 0.724367 |
| 1345 | cg05090375 | cg05090375_BC21 | -1.2144246 |
| 1346 | cg27312987 | cg27312987_TC21 | -3.47E-05 |
| 1347 | cg25361524 | cg25361524_TC21 | 1.14097207 |
| 1348 | cg04591799 | cg04591799_TC21 | 0.42828506 |
| 1349 | cg23664486 | cg23664486_BC21 | 1.92259578 |
| 1350 | cg09173939 | cg09173939_BC21 | -0.0373183 |
| 1351 | cg06976598 | cg06976598_TC21 | -0.7449498 |
| 1352 | cg10369049 | cg10369049_BC21 | -1.7528355 |
| 1353 | cg20631120 | cg20631120_TC21 | 0.02047126 |
| 1354 | cg16322747 | cg16322747_BC21 | 1.85148099 |
| 1355 | cg10499042 | cg10499042_BC21 | -1.1388688 |
| 1356 | cg20801476 | cg20801476_BC21 | 3.15953159 |
| 1357 | cg15999702 | cg15999702_TC21 | 0.59757282 |
| 1358 | cg19719042 | cg19719042_BC21 | -0.3669018 |
| 1359 | cg18833619 | cg18833619_BC21 | -0.1155581 |
| 1360 | cg06138239 | cg06138239_BC21 | 1.00599615 |
| 1361 | cg19750606 | cg19750606_BC21 | 1.39048498 |
| 1362 | cg03091982 | cg03091982_BC21 | 0.02569701 |
| 1363 | cg27656728 | cg27656728_BC21 | -1.2593838 |
| 1364 | cg06574789 | cg06574789_BC21 | -0.3495502 |
| 1365 | cg01799856 | cg01799856_BC21 | -0.2594186 |
| 1366 | cg14513685 | cg14513685_BC21 | 0.95408538 |
| 1367 | cg18614443 | cg18614443_BC21 | 0.32122468 |
| 1368 | cg27184055 | cg27184055_TC21 | 0.18792659 |
| 1369 | cg14029100 | cg14029100_TC21 | 0.16194082 |
| 1370 | cg22072332 | cg22072332_BC21 | 0.06874745 |
| 1371 | cg21031705 | cg21031705_TC21 | -0.1988118 |
| 1372 | cg03102881 | cg03102881_TC21 | -0.0924342 |
| 1373 | cg10113885 | cg10113885_BC21 | 0.42832843 |
| 1374 | cg08106532 | cg08106532_TC21 | -0.3844558 |
| 1375 | cg24170166 | cg24170166_BC21 | -2.4379056 |
| 1376 | cg13019173 | cg13019173_BC21 | 1.61703489 |
| 1377 | cg14441506 | cg14441506_TC21 | -1.5635237 |
| 1378 | cg13487127 | cg13487127_BC21 | -1.1334251 |
| 1379 | cg07867072 | cg07867072_BC21 | 2.06845918 |

Table S4.

**Immune Transcriptome Retroelement-Age**

| **Name** | **Coefficient** |
| --- | --- |
| (Intercept) | 47.2820312 |
| ERV316A3_10q22.3b | -0.5883676 |
| ERV316A3_13q32.1b | 0.21384207 |
| ERV316A3_2q32.2c | -0.1888955 |
| ERV316A3_2q34e | -0.7106562 |
| ERVLB4_11p15.5b | 1.34664716 |
| ERVLB4_13q12.11a | -0.8130629 |
| ERVLB4_15q23a | -0.3553158 |
| ERVLB4_6q15a | 0.0596267 |
| ERVLE_10p11.23 | -0.5766786 |
| ERVLE_1q25.3d | 0.7126799 |
| ERVLE_20p12.3a | 0.35016687 |
| HARLEQUIN_10q23.1 | -0.6741945 |
| HERV3_19p13.3 | -0.0361488 |
| HERV4_5q22.1 | -0.7792505 |
| HERV9_15q21.1b | 1.54005275 |
| HERV9_Yq11.221a | -0.3299653 |
| HERVE_8p23.1b | -0.0872594 |
| HERVEA_5q22.2 | -0.0395451 |
| HERVFRD_2p12a | -0.287477 |
| HERVH_13q13.3b | 0.00717553 |
| HERVH_2q34c | 0.17019075 |
| HERVH_3q22.1e | -0.0058787 |
| HERVH_5p15.32 | 0.3897048 |
| HERVH_5q22.1 | -0.1962073 |
| HERVH_6q22.31b | 0.03613837 |
| HERVH_Xq13.1b | -0.0821902 |
| HERVH_Xq27.3b | -0.3331063 |
| HERVIP10FH_11p15.5 | 0.02014944 |
| HERVIP10FH_Xp21.1 | 0.12195006 |
| HERVL_2q24.3b | 0.51459479 |
| HERVL_6q25.1 | -0.7135582 |
| HERVL_8q24.3a | -0.2156286 |
| HERVL_Xq28b | -0.2156816 |
| HERVS71_19q13.12a | -0.0330114 |
| HML5_2q32.1 | -0.005955 |
| HML6_19q13.41d | -0.2659298 |
| HUERSP2_Yq11.223 | -0.147641 |
| L1FLI_15q21.3 | -1.2055325 |
| L1FLnI_10p11.1c | 0.47853296 |
| L1FLnI_11p13a | -0.6506578 |
| L1FLnI_11p13w | 0.25647608 |
| L1FLnI_11q23.2d | -0.0436535 |
| L1FLnI_12q13.3b | -0.004746 |
| L1FLnI_12q23.1a | -0.0780051 |
| L1FLnI_13q32.1d | -0.192245 |
| L1FLnI_14q32.2f | -0.1763417 |
| L1FLnI_16p11.2e | 0.00649129 |
| L1FLnI_16q12.1g | 0.37730183 |
| L1FLnI_1p31.1oa | 0.64155304 |
| L1FLnI_1q25.3o | 0.33642401 |
| L1FLnI_1q31.3h | 0.08249789 |
| L1FLnI_1q41ma | -0.3447495 |
| L1FLnI_1q42.13f | -0.6817603 |
| L1FLnI_20p11.22g | -1.4936016 |
| L1FLnI_2q14.3p | -0.1813869 |
| L1FLnI_2q24.1o | -0.4906948 |
| L1FLnI_2q31.1n | -0.4918823 |
| L1FLnI_2q37.2b | -0.2110271 |
| L1FLnI_3p11.1o | 0.17567341 |
| L1FLnI_3p14.2y | -0.0534034 |
| L1FLnI_3p24.3aa | 0.07723939 |
| L1FLnI_3p24.3w | 0.01050955 |
| L1FLnI_3q26.1la | 0.05655957 |
| L1FLnI_3q26.2a | 0.04119995 |
| L1FLnI_4q31.1d | 0.00526961 |
| L1FLnI_4q31.21t | 0.12312257 |
| L1FLnI_4q31.3j | -0.7116064 |
| L1FLnI_4q35.1a | -0.5474089 |
| L1FLnI_5p13.1o | 0.063005 |
| L1FLnI_5q22.2a | 0.06726551 |
| L1FLnI_6p22.3h | -1.5058704 |
| L1FLnI_6q12d | 2.21720274 |
| L1FLnI_6q27o | 0.06040041 |
| L1FLnI_7p14.3e | 0.05568037 |
| L1FLnI_7q31.1b | -0.0319435 |
| L1FLnI_8p21.3f | 0.10483239 |
| L1FLnI_8q11.1b | -0.2918253 |
| L1FLnI_8q22.3c | -1.2838845 |
| L1FLnI_8q24.11b | -0.3146196 |
| L1FLnI_Xq13.1k | 0.46869392 |
| L1FLnI_Xq22.3z | 0.03808507 |
| L1FLnI_Xq27.1m | 0.29012196 |
| L1FLnI_Xq27.3b | -1.6556919 |
| LTR25_3p22.1 | -0.0141988 |
| MER101_15q21.2a | 1.59051184 |
| MER101_2q35b | 0.18289713 |
| MER4_12q12e | 0.10635722 |
| MER4_1p21.3 | -0.0883378 |
| MER41_11q21 | 0.31812974 |
| MER41_22q12.3d | 0.255033 |
| MER4B_14q32.33d | -0.0245824 |
| MER4B_Yq11.223b | -0.5874286 |
| MER61_7p21.3b | -0.1969914 |
| PABLA_11q13.4 | 0.31387025 |
| PRIMA41_19q13.2a | -0.0373635 |

Table S5.

**Human Multi-Tissue Retroelement clock CpGs**

|  | **ProbeID** | **Coefficient** |
| --- | --- | --- |
| 1 | (Intercept) | -97.247883 |
| 2 | cg00018667 | -1.415668 |
| 3 | cg00122653 | -25.834253 |
| 4 | cg00123704 | -0.5469348 |
| 5 | cg00156928 | 3.54799727 |
| 6 | cg00168389 | -2.3306546 |
| 7 | cg00195561 | -1.2649221 |
| 8 | cg00216340 | -2.0138083 |
| 9 | cg00231618 | 3.18095277 |
| 10 | cg00252803 | 1.51648595 |
| 11 | cg00270563 | 14.0662361 |
| 12 | cg00336986 | -0.6249703 |
| 13 | cg00346984 | -16.937817 |
| 14 | cg00376357 | -35.065913 |
| 15 | cg00387982 | 0.1095826 |
| 16 | cg00395291 | -0.0931865 |
| 17 | cg00420722 | -47.831997 |
| 18 | cg00428263 | -6.7088938 |
| 19 | cg00511107 | -1.1531654 |
| 20 | cg00527304 | 4.50242442 |
| 21 | cg00530252 | 1.19843419 |
| 22 | cg00587965 | 12.5719528 |
| 23 | cg00618078 | 2.52470727 |
| 24 | cg00622105 | 11.5535632 |
| 25 | cg00652027 | 78.5528598 |
| 26 | cg00682712 | -13.695629 |
| 27 | cg00697723 | -18.488337 |
| 28 | cg00702506 | -4.9251445 |
| 29 | cg00712356 | 6.6958907 |
| 30 | cg00741800 | -3.2722138 |
| 31 | cg00797286 | -0.0238185 |
| 32 | cg00807122 | 1.26742863 |
| 33 | cg00810473 | 8.56628514 |
| 34 | cg00873533 | 2.58898918 |
| 35 | cg00942188 | 24.0929394 |
| 36 | cg00976176 | -7.3165112 |
| 37 | cg01013732 | -2.4389603 |
| 38 | cg01025794 | -57.175336 |
| 39 | cg01025883 | -0.3149371 |
| 40 | cg01040649 | -1.9829354 |
| 41 | cg01079515 | 0.30786045 |
| 42 | cg01092133 | 8.76550207 |
| 43 | cg01134061 | -47.826807 |
| 44 | cg01164221 | -17.88747 |
| 45 | cg01185766 | 2.14507743 |
| 46 | cg01233449 | -1.8324194 |
| 47 | cg01240653 | 8.12668523 |
| 48 | cg01358199 | 0.76490463 |
| 49 | cg01448062 | -1.6053013 |
| 50 | cg01480911 | 7.66875762 |
| 51 | cg01492734 | -0.4047531 |
| 52 | cg01532487 | 1.80237431 |
| 53 | cg01661494 | 7.41850555 |
| 54 | cg01763851 | -0.9821231 |
| 55 | cg01772956 | 8.49448369 |
| 56 | cg01785096 | 2.88181729 |
| 57 | cg01793397 | -0.7671183 |
| 58 | cg01814671 | 1.56546524 |
| 59 | cg01940058 | -16.787139 |
| 60 | cg01972547 | -5.0214244 |
| 61 | cg01985465 | 5.18658771 |
| 62 | cg02046994 | -2.0519535 |
| 63 | cg02068164 | 2.37924004 |
| 64 | cg02113955 | -3.4750084 |
| 65 | cg02118776 | 72.0210541 |
| 66 | cg02200666 | 10.0473955 |
| 67 | cg02216595 | 0.30667549 |
| 68 | cg02226125 | 15.6342501 |
| 69 | cg02253978 | 2.60988445 |
| 70 | cg02278912 | 0.22454998 |
| 71 | cg02302816 | -0.2880811 |
| 72 | cg02327522 | -1.6888295 |
| 73 | cg02333875 | -4.8498141 |
| 74 | cg02376664 | -1.3024935 |
| 75 | cg02409364 | -0.4303547 |
| 76 | cg02412450 | 11.7097484 |
| 77 | cg02412628 | -2.3207062 |
| 78 | cg02461295 | 9.12519961 |
| 79 | cg02511570 | 0.99052283 |
| 80 | cg02636585 | 2.58811403 |
| 81 | cg02648101 | 17.7878772 |
| 82 | cg02663175 | -2.8934815 |
| 83 | cg02693996 | 10.3728378 |
| 84 | cg02724496 | -7.0287184 |
| 85 | cg02774855 | 11.3674139 |
| 86 | cg02786232 | 6.85765656 |
| 87 | cg02833379 | -1.5883872 |
| 88 | cg02861155 | -0.2441009 |
| 89 | cg02875664 | 7.40949813 |
| 90 | cg02893779 | -13.910044 |
| 91 | cg02904247 | -2.7131002 |
| 92 | cg02931100 | 4.31842428 |
| 93 | cg02950999 | 3.9316869 |
| 94 | cg02959351 | -2.4506634 |
| 95 | cg02995952 | -9.5937866 |
| 96 | cg03002086 | -4.7686656 |
| 97 | cg03030460 | -5.8445388 |
| 98 | cg03038016 | 14.3538398 |
| 99 | cg03042121 | 4.18559719 |
| 100 | cg03115743 | -1.1455075 |
| 101 | cg03251789 | -25.059079 |
| 102 | cg03263997 | -5.7486937 |
| 103 | cg03365037 | -2.3575822 |
| 104 | cg03396681 | -5.8843097 |
| 105 | cg03411918 | -7.8382848 |
| 106 | cg03418466 | -2.9104342 |
| 107 | cg03445802 | -21.369746 |
| 108 | cg03527426 | 0.16213082 |
| 109 | cg03578528 | -18.721906 |
| 110 | cg03710824 | 1.82969934 |
| 111 | cg03781727 | 4.40847486 |
| 112 | cg03791828 | 8.45619767 |
| 113 | cg03871327 | 3.65181784 |
| 114 | cg03877913 | 2.85451644 |
| 115 | cg03887721 | -1.8642962 |
| 116 | cg03904134 | -0.1465768 |
| 117 | cg03982487 | 2.62869048 |
| 118 | cg04022555 | 2.43658856 |
| 119 | cg04127823 | -2.1858359 |
| 120 | cg04260051 | -2.5166092 |
| 121 | cg04282186 | -2.6297297 |
| 122 | cg04289033 | 1.41775766 |
| 123 | cg04293662 | -6.5558929 |
| 124 | cg04305916 | -5.8006379 |
| 125 | cg04332448 | 17.1370592 |
| 126 | cg04407732 | 2.88926095 |
| 127 | cg04455646 | -0.0455564 |
| 128 | cg04455886 | 21.5509826 |
| 129 | cg04476692 | 14.3857594 |
| 130 | cg04489293 | -90.773507 |
| 131 | cg04517512 | -20.616981 |
| 132 | cg04606663 | -4.8307313 |
| 133 | cg04606867 | -10.028981 |
| 134 | cg04609959 | 5.95638405 |
| 135 | cg04666202 | -44.832569 |
| 136 | cg04749503 | -1.2097517 |
| 137 | cg04757799 | 5.13244893 |
| 138 | cg04767027 | 23.4428074 |
| 139 | cg04847817 | -12.280379 |
| 140 | cg04881646 | 8.10702797 |
| 141 | cg04897744 | -7.6233595 |
| 142 | cg04937388 | 0.34204644 |
| 143 | cg04948963 | 18.2941362 |
| 144 | cg05064509 | -3.7926673 |
| 145 | cg05096492 | -1.5713466 |
| 146 | cg05154694 | -6.2047148 |
| 147 | cg05186223 | 0.51206086 |
| 148 | cg05202858 | -5.2548397 |
| 149 | cg05308829 | 8.67587143 |
| 150 | cg05342157 | 4.51416655 |
| 151 | cg05429123 | -24.570038 |
| 152 | cg05433449 | 0.37204852 |
| 153 | cg05439724 | -0.2687667 |
| 154 | cg05521606 | -0.6106047 |
| 155 | cg05536132 | -12.079843 |
| 156 | cg05602479 | -11.12613 |
| 157 | cg05606767 | 0.27821276 |
| 158 | cg05627800 | 14.8474401 |
| 159 | cg05656876 | 1.5170763 |
| 160 | cg05665581 | 2.00805732 |
| 161 | cg05683630 | 0.15896366 |
| 162 | cg05754690 | 7.54321249 |
| 163 | cg05769946 | 8.90410994 |
| 164 | cg05772329 | -1.7921417 |
| 165 | cg05780100 | -1.558431 |
| 166 | cg05877899 | -4.3333269 |
| 167 | cg05892615 | 16.9933534 |
| 168 | cg05927830 | -38.209811 |
| 169 | cg05948994 | -5.4118207 |
| 170 | cg05980523 | -8.8027518 |
| 171 | cg06024951 | -1.0816531 |
| 172 | cg06062094 | 21.3411594 |
| 173 | cg06078588 | -2.0695551 |
| 174 | cg06086595 | -1.737479 |
| 175 | cg06166559 | -18.318178 |
| 176 | cg06166789 | 3.43263052 |
| 177 | cg06199438 | 0.30859499 |
| 178 | cg06262458 | -0.0017353 |
| 179 | cg06329185 | -1.5259083 |
| 180 | cg06341957 | 7.3704898 |
| 181 | cg06395490 | 0.46872453 |
| 182 | cg06412045 | 0.6439197 |
| 183 | cg06427621 | -4.1883047 |
| 184 | cg06428717 | -0.5781532 |
| 185 | cg06462760 | -0.3078847 |
| 186 | cg06475764 | 26.9263482 |
| 187 | cg06519883 | -1.1331797 |
| 188 | cg06570674 | 0.08154914 |
| 189 | cg06600444 | -1.2578919 |
| 190 | cg06632210 | 1.04956432 |
| 191 | cg06651172 | 3.60719408 |
| 192 | cg06663713 | -3.1980383 |
| 193 | cg06787151 | 0.44504337 |
| 194 | cg06801384 | -3.663845 |
| 195 | cg06833271 | -8.0618773 |
| 196 | cg06878166 | -1.4732632 |
| 197 | cg06906965 | 0.09748978 |
| 198 | cg06913517 | -12.188668 |
| 199 | cg06988310 | 68.2206814 |
| 200 | cg07020697 | 4.87182542 |
| 201 | cg07027713 | 0.7436203 |
| 202 | cg07054255 | 9.56032145 |
| 203 | cg07072281 | 0.01931322 |
| 204 | cg07085775 | -2.5005369 |
| 205 | cg07102783 | 1.42882728 |
| 206 | cg07128371 | -9.9467821 |
| 207 | cg07149935 | -11.531754 |
| 208 | cg07258788 | -12.075513 |
| 209 | cg07274340 | -0.0015941 |
| 210 | cg07313576 | 0.13012434 |
| 211 | cg07318113 | 0.0837745 |
| 212 | cg07335890 | -0.6780712 |
| 213 | cg07346020 | -4.1013278 |
| 214 | cg07355632 | 0.15732976 |
| 215 | cg07393857 | 0.76100759 |
| 216 | cg07474621 | -6.7965098 |
| 217 | cg07499026 | -2.3742037 |
| 218 | cg07514693 | -0.5134503 |
| 219 | cg07530325 | -5.8372921 |
| 220 | cg07544832 | -1.3401968 |
| 221 | cg07554030 | 2.91761167 |
| 222 | cg07588172 | 11.418095 |
| 223 | cg07599182 | 0.25309148 |
| 224 | cg07659168 | -4.3913035 |
| 225 | cg07705045 | -0.7356393 |
| 226 | cg07734052 | -9.6921174 |
| 227 | cg07745748 | 2.00603245 |
| 228 | cg07746282 | -16.437055 |
| 229 | cg07766382 | 15.3569481 |
| 230 | cg07938921 | -6.9725316 |
| 231 | cg08018188 | -0.4253432 |
| 232 | cg08043971 | -2.0061891 |
| 233 | cg08048252 | 6.17117599 |
| 234 | cg08052069 | 2.63728337 |
| 235 | cg08068640 | 4.83062278 |
| 236 | cg08104032 | 0.61069303 |
| 237 | cg08105005 | 6.30961128 |
| 238 | cg08121845 | -3.809873 |
| 239 | cg08144931 | 0.64097924 |
| 240 | cg08200809 | -5.5593321 |
| 241 | cg08219087 | 1.24614072 |
| 242 | cg08227926 | -0.059189 |
| 243 | cg08235477 | -3.8404707 |
| 244 | cg08248985 | -7.2390815 |
| 245 | cg08251203 | -1.3151248 |
| 246 | cg08256665 | -1.4902933 |
| 247 | cg08326089 | -4.8148344 |
| 248 | cg08337927 | -1.679927 |
| 249 | cg08353308 | -2.7242648 |
| 250 | cg08356333 | -0.0658725 |
| 251 | cg08368411 | 3.05943763 |
| 252 | cg08395793 | -1.0918833 |
| 253 | cg08414690 | 2.45174162 |
| 254 | cg08454687 | -7.9360061 |
| 255 | cg08466108 | -1.0396285 |
| 256 | cg08473249 | 8.36827506 |
| 257 | cg08521545 | -20.300117 |
| 258 | cg08530075 | 1.77193187 |
| 259 | cg08543976 | 4.96734332 |
| 260 | cg08554366 | -1.971103 |
| 261 | cg08576959 | 3.72427062 |
| 262 | cg08599433 | 12.2398016 |
| 263 | cg08637691 | 45.0660707 |
| 264 | cg08714076 | 4.2846185 |
| 265 | cg08770536 | -1.497809 |
| 266 | cg08786444 | 0.61802938 |
| 267 | cg08792791 | -3.7705833 |
| 268 | cg08819285 | 7.53929042 |
| 269 | cg08851837 | -0.019208 |
| 270 | cg08870845 | -1.2278122 |
| 271 | cg08903584 | 0.4573937 |
| 272 | cg08933775 | 10.8189212 |
| 273 | cg08955483 | 0.59615927 |
| 274 | cg09023160 | 0.51507954 |
| 275 | cg09024069 | 1.81283506 |
| 276 | cg09050126 | -2.6988793 |
| 277 | cg09080396 | 1.13147838 |
| 278 | cg09108044 | -2.7266019 |
| 279 | cg09156165 | 3.03937104 |
| 280 | cg09164709 | 3.17838986 |
| 281 | cg09207765 | -3.5053117 |
| 282 | cg09293319 | -0.2955993 |
| 283 | cg09332175 | -1.744223 |
| 284 | cg09332960 | 0.04599681 |
| 285 | cg09409957 | 1.94786147 |
| 286 | cg09447895 | 11.8130981 |
| 287 | cg09459471 | 9.00329824 |
| 288 | cg09563805 | -0.561592 |
| 289 | cg09658720 | 2.10261183 |
| 290 | cg09716878 | 3.29536204 |
| 291 | cg09732368 | 2.14373214 |
| 292 | cg09866910 | 5.32924583 |
| 293 | cg09905360 | -5.152539 |
| 294 | cg09913382 | 5.71492324 |
| 295 | cg10002742 | 1.7355577 |
| 296 | cg10070101 | -0.0141854 |
| 297 | cg10092241 | 9.55046153 |
| 298 | cg10105623 | -9.9904917 |
| 299 | cg10125636 | 6.81236925 |
| 300 | cg10148067 | 0.00180399 |
| 301 | cg10160988 | 0.6348613 |
| 302 | cg10202782 | -3.0307529 |
| 303 | cg10298992 | 1.45733527 |
| 304 | cg10318641 | 5.65817126 |
| 305 | cg10355041 | 1.37821804 |
| 306 | cg10375174 | 3.40877943 |
| 307 | cg10382675 | -1.3931853 |
| 308 | cg10422406 | 0.00371289 |
| 309 | cg10469522 | 4.05849896 |
| 310 | cg10557565 | 2.56607283 |
| 311 | cg10594913 | -0.2211484 |
| 312 | cg10617419 | 4.02085141 |
| 313 | cg10631097 | -2.2868257 |
| 314 | cg10634450 | 45.8766428 |
| 315 | cg10707823 | 30.2482633 |
| 316 | cg10738340 | -0.698458 |
| 317 | cg10759769 | -1.1676396 |
| 318 | cg10864941 | -14.639671 |
| 319 | cg10939112 | 2.83976728 |
| 320 | cg10969241 | -0.7903264 |
| 321 | cg11046421 | -1.5149443 |
| 322 | cg11070604 | 1.14625568 |
| 323 | cg11099463 | 2.24825334 |
| 324 | cg11107858 | -0.9659343 |
| 325 | cg11117381 | 2.10486238 |
| 326 | cg11117703 | -9.9368068 |
| 327 | cg11125823 | -46.820595 |
| 328 | cg11142525 | 1.74260374 |
| 329 | cg11155693 | -2.283824 |
| 330 | cg11268935 | 86.6126942 |
| 331 | cg11279918 | 41.8297073 |
| 332 | cg11280438 | 3.74952659 |
| 333 | cg11291747 | 15.1709086 |
| 334 | cg11300186 | 9.65553887 |
| 335 | cg11314827 | -1.0691594 |
| 336 | cg11325420 | -0.8182923 |
| 337 | cg11353593 | -0.9664965 |
| 338 | cg11365128 | -6.5527539 |
| 339 | cg11454513 | 9.52198568 |
| 340 | cg11480845 | -0.2143273 |
| 341 | cg11492856 | -11.703304 |
| 342 | cg11495626 | -9.3798601 |
| 343 | cg11574151 | -2.3092713 |
| 344 | cg11625382 | -2.8354245 |
| 345 | cg11651790 | 0.21221526 |
| 346 | cg11651896 | 2.83095767 |
| 347 | cg11658909 | 5.35606664 |
| 348 | cg11708780 | 11.6564939 |
| 349 | cg11776269 | 2.58238973 |
| 350 | cg11831043 | 8.72336921 |
| 351 | cg11835459 | -0.2864189 |
| 352 | cg11907607 | 17.9005852 |
| 353 | cg11909587 | -7.6186766 |
| 354 | cg11921473 | 25.9376436 |
| 355 | cg11975053 | -0.3299506 |
| 356 | cg12007048 | -6.4867778 |
| 357 | cg12013770 | 2.2093035 |
| 358 | cg12071799 | 3.66868534 |
| 359 | cg12101256 | 17.3967553 |
| 360 | cg12125006 | -0.3535471 |
| 361 | cg12184344 | 0.07795238 |
| 362 | cg12188903 | 0.25502028 |
| 363 | cg12217291 | 0.33407511 |
| 364 | cg12270633 | 0.5202413 |
| 365 | cg12298075 | -15.991701 |
| 366 | cg12419581 | -0.2134286 |
| 367 | cg12436498 | 4.53349546 |
| 368 | cg12585039 | 34.223121 |
| 369 | cg12641578 | 26.7666 |
| 370 | cg12784422 | -1.8352515 |
| 371 | cg12786012 | 9.96785293 |
| 372 | cg12836721 | -16.717928 |
| 373 | cg12871964 | -2.9221419 |
| 374 | cg12931853 | 7.75073093 |
| 375 | cg12947436 | 11.6418454 |
| 376 | cg13011204 | 0.88251733 |
| 377 | cg13011453 | -1.5613884 |
| 378 | cg13044223 | 1.26759268 |
| 379 | cg13060282 | -1.3071167 |
| 380 | cg13063704 | -0.3209753 |
| 381 | cg13072775 | 2.9790169 |
| 382 | cg13135437 | 1.71811956 |
| 383 | cg13136032 | 3.34995984 |
| 384 | cg13237509 | -3.2420491 |
| 385 | cg13254082 | 0.88868021 |
| 386 | cg13281762 | -6.6283152 |
| 387 | cg13286836 | -11.656636 |
| 388 | cg13321176 | -0.4328827 |
| 389 | cg13326063 | -0.8629577 |
| 390 | cg13397720 | -0.3763212 |
| 391 | cg13423535 | 0.31654334 |
| 392 | cg13430489 | 2.54192597 |
| 393 | cg13489590 | -0.1590209 |
| 394 | cg13490915 | -2.7961952 |
| 395 | cg13546639 | 2.87628726 |
| 396 | cg13546758 | -5.4925249 |
| 397 | cg13624321 | -2.1339302 |
| 398 | cg13640414 | -8.1251468 |
| 399 | cg13695309 | 8.85624239 |
| 400 | cg13712459 | -3.2855526 |
| 401 | cg13852833 | 30.8387263 |
| 402 | cg13925066 | -4.932884 |
| 403 | cg13928183 | 1.08146842 |
| 404 | cg13945778 | 2.09505284 |
| 405 | cg13959356 | -0.0585262 |
| 406 | cg13960015 | 9.58374834 |
| 407 | cg13968476 | 0.36540637 |
| 408 | cg14005725 | -16.316752 |
| 409 | cg14038693 | -17.480486 |
| 410 | cg14175766 | -1.8239512 |
| 411 | cg14247180 | 44.1861466 |
| 412 | cg14318455 | -13.857262 |
| 413 | cg14328273 | 9.53026771 |
| 414 | cg14331720 | 1.20430472 |
| 415 | cg14364661 | 1.36475728 |
| 416 | cg14392725 | 0.5479819 |
| 417 | cg14428590 | -15.273483 |
| 418 | cg14488658 | 2.81012387 |
| 419 | cg14535138 | -1.2422798 |
| 420 | cg14543770 | 1.36480906 |
| 421 | cg14584616 | -2.9745346 |
| 422 | cg14587213 | -0.0244182 |
| 423 | cg14593954 | 2.85923668 |
| 424 | cg14646998 | -1.357019 |
| 425 | cg14661773 | 8.74084459 |
| 426 | cg14688590 | 8.81122209 |
| 427 | cg14699057 | 4.76858006 |
| 428 | cg14729824 | 1.40443104 |
| 429 | cg14821564 | -4.463049 |
| 430 | cg14825889 | -5.4843035 |
| 431 | cg14839501 | 0.98315275 |
| 432 | cg14933812 | -4.8960473 |
| 433 | cg14990605 | 0.27241364 |
| 434 | cg15003393 | 0.75997761 |
| 435 | cg15028161 | 0.18318098 |
| 436 | cg15092213 | 4.65986968 |
| 437 | cg15124019 | 5.99577283 |
| 438 | cg15145868 | -0.2590099 |
| 439 | cg15229765 | -1.8082037 |
| 440 | cg15249411 | -3.1340521 |
| 441 | cg15302800 | -7.2098482 |
| 442 | cg15383195 | -0.0347184 |
| 443 | cg15390864 | 5.5632658 |
| 444 | cg15478441 | 14.0215378 |
| 445 | cg15486609 | 6.92150513 |
| 446 | cg15505543 | -2.8867494 |
| 447 | cg15512289 | -1.0367147 |
| 448 | cg15569228 | -7.4546749 |
| 449 | cg15574240 | -1.4769838 |
| 450 | cg15597846 | -0.6067956 |
| 451 | cg15601996 | -2.7270001 |
| 452 | cg15663872 | -0.727392 |
| 453 | cg15699243 | -1.7021604 |
| 454 | cg15799543 | -0.1642306 |
| 455 | cg15810640 | -2.4254807 |
| 456 | cg15830378 | -1.9701927 |
| 457 | cg15868751 | 9.0309393 |
| 458 | cg15941089 | -0.0280064 |
| 459 | cg16012294 | 1.15202147 |
| 460 | cg16034046 | -0.1561398 |
| 461 | cg16152600 | -2.7479487 |
| 462 | cg16282818 | 14.3332866 |
| 463 | cg16305753 | 0.14175955 |
| 464 | cg16358215 | 16.8705921 |
| 465 | cg16372928 | -10.896277 |
| 466 | cg16432469 | -3.6237146 |
| 467 | cg16471401 | -18.768436 |
| 468 | cg16591893 | 0.44828614 |
| 469 | cg16615280 | 8.21299195 |
| 470 | cg16640959 | 0.185881 |
| 471 | cg16688434 | 19.3876888 |
| 472 | cg16702999 | 7.00424564 |
| 473 | cg16750731 | -0.000571 |
| 474 | cg16769369 | 1.88860867 |
| 475 | cg16826333 | 29.3309348 |
| 476 | cg16849999 | -7.248337 |
| 477 | cg16865634 | 0.38175906 |
| 478 | cg16949898 | 0.38477922 |
| 479 | cg16968098 | 0.18207246 |
| 480 | cg17044074 | -4.2491901 |
| 481 | cg17061191 | -9.3505784 |
| 482 | cg17349025 | -23.878002 |
| 483 | cg17400177 | 4.03515506 |
| 484 | cg17402123 | -1.7062313 |
| 485 | cg17408686 | -3.350549 |
| 486 | cg17448156 | -12.007936 |
| 487 | cg17472686 | -0.0668396 |
| 488 | cg17488989 | -0.4182372 |
| 489 | cg17568131 | -1.0894775 |
| 490 | cg17581721 | -25.058685 |
| 491 | cg17611433 | -7.0033089 |
| 492 | cg17622401 | 5.74905941 |
| 493 | cg17656330 | -1.1378944 |
| 494 | cg17713488 | 0.31892942 |
| 495 | cg17853132 | -0.924948 |
| 496 | cg17893846 | 18.17538 |
| 497 | cg17936846 | 0.69438853 |
| 498 | cg17957543 | -0.4684583 |
| 499 | cg18007983 | -0.3739194 |
| 500 | cg18051740 | 0.32706059 |
| 501 | cg18059499 | 31.7656067 |
| 502 | cg18064360 | 0.57901173 |
| 503 | cg18097246 | 0.41511762 |
| 504 | cg18099237 | 4.32955088 |
| 505 | cg18137921 | 69.2408925 |
| 506 | cg18141396 | -1.2287494 |
| 507 | cg18224693 | 0.67084249 |
| 508 | cg18232235 | 0.11897724 |
| 509 | cg18280859 | -1.4315553 |
| 510 | cg18302737 | -7.4310648 |
| 511 | cg18309452 | -1.062341 |
| 512 | cg18383124 | 11.2187086 |
| 513 | cg18453143 | -0.2052449 |
| 514 | cg18470743 | 5.6659475 |
| 515 | cg18537410 | 16.5889889 |
| 516 | cg18546846 | -6.3062058 |
| 517 | cg18597415 | 3.27418096 |
| 518 | cg18651643 | 2.87760107 |
| 519 | cg18704658 | -42.039924 |
| 520 | cg18746137 | 17.6960045 |
| 521 | cg18844029 | -0.9932911 |
| 522 | cg18893176 | -2.0390911 |
| 523 | cg18910656 | -2.1081606 |
| 524 | cg18957228 | 0.71413796 |
| 525 | cg18973654 | -3.2239632 |
| 526 | cg19068238 | -6.2251303 |
| 527 | cg19073447 | -13.05439 |
| 528 | cg19089045 | 1.0667568 |
| 529 | cg19110725 | -0.3332918 |
| 530 | cg19125532 | 4.67740724 |
| 531 | cg19140834 | 2.99155222 |
| 532 | cg19230800 | 3.01240592 |
| 533 | cg19271098 | 4.42172118 |
| 534 | cg19357976 | 6.23020596 |
| 535 | cg19370234 | -1.4004715 |
| 536 | cg19396593 | -3.200777 |
| 537 | cg19471156 | 9.27050343 |
| 538 | cg19490374 | 2.00564121 |
| 539 | cg19493444 | -0.2301767 |
| 540 | cg19529484 | 1.33544914 |
| 541 | cg19541307 | -6.3126534 |
| 542 | cg19550294 | -0.3176734 |
| 543 | cg19587667 | -12.431312 |
| 544 | cg19602474 | 4.97618661 |
| 545 | cg19652860 | 7.47891064 |
| 546 | cg19683551 | -0.7580087 |
| 547 | cg19712447 | -0.9030534 |
| 548 | cg19733231 | 4.74845835 |
| 549 | cg19741675 | -2.2731998 |
| 550 | cg19837528 | -0.0610213 |
| 551 | cg19877656 | 7.28913049 |
| 552 | cg19888369 | -0.7123629 |
| 553 | cg19889330 | -3.2227928 |
| 554 | cg19918711 | 1.95006745 |
| 555 | cg19969349 | -22.121909 |
| 556 | cg19984320 | -2.1201876 |
| 557 | cg19995229 | 5.73286586 |
| 558 | cg20073451 | 12.6402539 |
| 559 | cg20087251 | -0.0537975 |
| 560 | cg20097209 | -7.6044172 |
| 561 | cg20110504 | -4.3696012 |
| 562 | cg20140023 | -2.8682001 |
| 563 | cg20179730 | -5.1150728 |
| 564 | cg20200412 | 1.6050327 |
| 565 | cg20224091 | -6.6379562 |
| 566 | cg20327913 | 31.3369285 |
| 567 | cg20368248 | -1.6334675 |
| 568 | cg20391883 | -4.6365735 |
| 569 | cg20452624 | 0.03214209 |
| 570 | cg20528007 | -0.0296507 |
| 571 | cg20568147 | 22.7406471 |
| 572 | cg20583816 | -5.15174 |
| 573 | cg20600034 | -1.9605972 |
| 574 | cg20631252 | -3.8802874 |
| 575 | cg20703392 | 2.78282888 |
| 576 | cg20713092 | 2.87333504 |
| 577 | cg20747619 | -2.0097768 |
| 578 | cg20757020 | 3.0698206 |
| 579 | cg20789138 | -1.4463037 |
| 580 | cg20882926 | -14.456057 |
| 581 | cg20883850 | 17.9332911 |
| 582 | cg20929826 | -1.6618461 |
| 583 | cg20989506 | 1.13629084 |
| 584 | cg21101308 | -1.5052697 |
| 585 | cg21142033 | -0.0226429 |
| 586 | cg21232341 | -5.4524513 |
| 587 | cg21240123 | 9.83557565 |
| 588 | cg21387653 | -0.8841788 |
| 589 | cg21388753 | 1.86623095 |
| 590 | cg21478928 | 10.7287999 |
| 591 | cg21504345 | -1.9199335 |
| 592 | cg21530449 | -14.181143 |
| 593 | cg21622568 | 1.80262227 |
| 594 | cg21645075 | 0.00670073 |
| 595 | cg21660413 | -17.601575 |
| 596 | cg21766902 | 19.7584843 |
| 597 | cg21785437 | -3.536415 |
| 598 | cg21803548 | -4.7302619 |
| 599 | cg21992844 | -18.842358 |
| 600 | cg21995430 | -13.54494 |
| 601 | cg22011361 | 0.66891185 |
| 602 | cg22055730 | -1.7568966 |
| 603 | cg22195268 | 8.5474638 |
| 604 | cg22220991 | 4.93719903 |
| 605 | cg22277154 | -0.0317646 |
| 606 | cg22290048 | -3.7826886 |
| 607 | cg22332770 | 11.601446 |
| 608 | cg22361181 | -0.3056832 |
| 609 | cg22458693 | 1.02457269 |
| 610 | cg22464692 | 0.36090973 |
| 611 | cg22493358 | -0.4653431 |
| 612 | cg22568590 | 15.5686159 |
| 613 | cg22593095 | 5.333394 |
| 614 | cg22599539 | 1.10877262 |
| 615 | cg22633057 | -5.2851485 |
| 616 | cg22688447 | -3.0041541 |
| 617 | cg22709609 | -9.7440851 |
| 618 | cg22723023 | 0.58363002 |
| 619 | cg22813290 | -11.537686 |
| 620 | cg22851864 | 0.0070786 |
| 621 | cg22857872 | -10.288432 |
| 622 | cg22897181 | -32.687463 |
| 623 | cg22996588 | 0.0197846 |
| 624 | cg23005405 | -1.3615646 |
| 625 | cg23026478 | 19.7763449 |
| 626 | cg23028072 | 26.6418412 |
| 627 | cg23117829 | -4.8373793 |
| 628 | cg23118151 | 0.36080611 |
| 629 | cg23251303 | -4.6363128 |
| 630 | cg23291271 | -5.6571557 |
| 631 | cg23307153 | -3.7477507 |
| 632 | cg23328777 | 1.11420883 |
| 633 | cg23332335 | 1.0798266 |
| 634 | cg23338945 | -0.4346207 |
| 635 | cg23387904 | 1.20544451 |
| 636 | cg23422998 | 14.5070528 |
| 637 | cg23494690 | 3.74167434 |
| 638 | cg23524699 | 6.38748403 |
| 639 | cg23528104 | 0.0042534 |
| 640 | cg23552369 | -4.0510548 |
| 641 | cg23601861 | 2.15893135 |
| 642 | cg23604699 | 0.49098043 |
| 643 | cg23624621 | -0.1662873 |
| 644 | cg23639784 | 0.63130139 |
| 645 | cg23716564 | 6.72918891 |
| 646 | cg23720931 | 12.9367198 |
| 647 | cg23755669 | -0.3856179 |
| 648 | cg23780067 | 0.33241803 |
| 649 | cg23838231 | 2.27369085 |
| 650 | cg23844115 | 2.4332555 |
| 651 | cg23855920 | 0.32475188 |
| 652 | cg23895001 | -4.6647355 |
| 653 | cg23937224 | 7.85710916 |
| 654 | cg24059765 | -3.2611464 |
| 655 | cg24098060 | -23.477357 |
| 656 | cg24140061 | -0.3717516 |
| 657 | cg24141298 | -45.54489 |
| 658 | cg24162150 | 4.31498947 |
| 659 | cg24254120 | 24.3128205 |
| 660 | cg24286200 | 1.1211841 |
| 661 | cg24303768 | -0.0317036 |
| 662 | cg24312918 | 0.49888703 |
| 663 | cg24341612 | 10.6281367 |
| 664 | cg24409440 | 0.24300903 |
| 665 | cg24508475 | -19.256895 |
| 666 | cg24508942 | 0.11717925 |
| 667 | cg24521351 | -0.4131202 |
| 668 | cg24523069 | -0.2249732 |
| 669 | cg24636867 | -38.368554 |
| 670 | cg24701257 | -14.375714 |
| 671 | cg24736114 | -1.2121264 |
| 672 | cg24739320 | -0.0028853 |
| 673 | cg24836679 | -4.4095286 |
| 674 | cg24846373 | -3.1408896 |
| 675 | cg24934408 | -4.7947364 |
| 676 | cg25021026 | -1.9487337 |
| 677 | cg25032603 | -7.6181168 |
| 678 | cg25039336 | 1.26367358 |
| 679 | cg25048816 | 2.05663505 |
| 680 | cg25073435 | 9.68512422 |
| 681 | cg25162456 | 8.53078777 |
| 682 | cg25237970 | 0.0425078 |
| 683 | cg25259432 | -3.225881 |
| 684 | cg25283432 | 7.65684106 |
| 685 | cg25311808 | -0.4709617 |
| 686 | cg25322106 | -8.4112245 |
| 687 | cg25360855 | 21.498081 |
| 688 | cg25382740 | 14.0095749 |
| 689 | cg25421647 | 6.4408543 |
| 690 | cg25447725 | 6.91440629 |
| 691 | cg25528758 | -13.957927 |
| 692 | cg25544434 | -2.0602831 |
| 693 | cg25595664 | 136.62119 |
| 694 | cg25611523 | -0.4605437 |
| 695 | cg25727206 | 0.00361091 |
| 696 | cg25749982 | 2.38155713 |
| 697 | cg25788006 | -1.4009957 |
| 698 | cg25863070 | 3.66771723 |
| 699 | cg25929202 | 0.38620237 |
| 700 | cg25982983 | -6.6930577 |
| 701 | cg25995599 | 8.23538902 |
| 702 | cg26012092 | 4.33927177 |
| 703 | cg26074539 | 4.42861109 |
| 704 | cg26175749 | 2.90810827 |
| 705 | cg26191723 | 2.99864603 |
| 706 | cg26299710 | -1.3521774 |
| 707 | cg26302238 | 0.78731961 |
| 708 | cg26332797 | 0.95492051 |
| 709 | cg26355596 | 1.10911715 |
| 710 | cg26373411 | 2.89598343 |
| 711 | cg26412531 | -0.5212173 |
| 712 | cg26440811 | 2.57147039 |
| 713 | cg26461537 | -0.5077366 |
| 714 | cg26500288 | -15.033523 |
| 715 | cg26529484 | 9.95179914 |
| 716 | cg26656977 | 8.89504336 |
| 717 | cg26745000 | -16.327166 |
| 718 | cg26814404 | 18.9951385 |
| 719 | cg26891247 | -0.9088488 |
| 720 | cg26979056 | 19.3315843 |
| 721 | cg26981311 | 18.3807983 |
| 722 | cg26999726 | 6.17201752 |
| 723 | cg27119551 | -3.6664817 |
| 724 | cg27136259 | 9.83268223 |
| 725 | cg27250927 | 30.4074494 |
| 726 | cg27279244 | 0.15689092 |
| 727 | cg27345757 | 1.10099175 |
| 728 | cg27390437 | 1.19750582 |
| 729 | cg27476481 | 0.61067473 |
| 730 | cg27484216 | 7.11263464 |
| 731 | cg27531982 | -1.6125372 |
| 732 | cg27583987 | 1.61201792 |
| 733 | cg27593320 | 1.42015422 |
| 734 | cg27602915 | -2.2200365 |
| 735 | cg27650180 | -8.3527363 |

Table S6.

**Pan-Mammalian Species Multi-Tissue Transposable Element Clock CpGs**

| ProbeID | coefficient |
| --- | --- |
| (Intercept) | 2.1620196 |
| cg00236396 | 0.11795164 |
| cg00271378 | 0.03792187 |
| cg00297600 | 0.04893819 |
| cg00325217 | 0.16040068 |
| cg00393367 | 0.04457867 |
| cg00399624 | 0.04603772 |
| cg00443833 | 0.03006427 |
| cg00445959 | -0.0410689 |
| cg00632889 | -0.0083374 |
| cg00658652 | 0.66616089 |
| cg00687674 | -0.0060876 |
| cg00702872 | -0.0291212 |
| cg00717629 | -0.085125 |
| cg00847072 | 0.04724182 |
| cg00851088 | 0.12825091 |
| cg00890651 | -0.0836402 |
| cg00976203 | 0.00190291 |
| cg00995686 | -0.0300654 |
| cg01002223 | -0.0290099 |
| cg01079563 | -0.0156005 |
| cg01241756 | -0.0249629 |
| cg01334830 | -0.0877132 |
| cg01391058 | -0.2679659 |
| cg01407797 | -0.0610594 |
| cg01523712 | 0.0767663 |
| cg01620430 | 1.13428331 |
| cg01635585 | 0.01580884 |
| cg01644993 | 0.27328393 |
| cg01668476 | -0.1095259 |
| cg01689963 | 0.20713138 |
| cg01810127 | 0.01156187 |
| cg01822313 | -0.2072382 |
| cg01837677 | 0.01052235 |
| cg02026974 | 0.19660705 |
| cg02033323 | -0.1902013 |
| cg02110337 | -0.0383163 |
| cg02123384 | 0.03780332 |
| cg02132051 | 0.00812749 |
| cg02151301 | -0.0801121 |
| cg02161890 | -0.0591849 |
| cg02315120 | -0.0213193 |
| cg02426735 | 0.15816761 |
| cg02574621 | 0.19376331 |
| cg02606840 | 0.0567952 |
| cg02607544 | -0.0570711 |
| cg02650131 | 0.07483089 |
| cg02714514 | 0.05125063 |
| cg02770318 | -0.1155025 |
| cg02771920 | 0.05115619 |
| cg02779913 | 0.14016384 |
| cg02821342 | -0.2641804 |
| cg03023496 | 0.05472362 |
| cg03031579 | -0.0564276 |
| cg03057744 | 0.03053956 |
| cg03077729 | 0.01012857 |
| cg03313271 | -0.0112212 |
| cg03429643 | 0.09851629 |
| cg03592537 | -0.0921009 |
| cg03603104 | 0.00023853 |
| cg03630442 | 0.0300993 |
| cg03674256 | -0.0347077 |
| cg03746976 | -0.0150423 |
| cg03779374 | -0.3585041 |
| cg03882802 | -0.179522 |
| cg03929330 | 0.11996077 |
| cg04126866 | -0.1225041 |
| cg04196926 | -0.0466684 |
| cg04305771 | -0.3195266 |
| cg04327785 | -0.1214844 |
| cg04347414 | 0.21911889 |
| cg04408104 | -0.0482439 |
| cg04416752 | 0.10590584 |
| cg04499392 | -0.0316247 |
| cg04576021 | -0.0478682 |
| cg04596060 | -0.1555869 |
| cg04631128 | -0.0367868 |
| cg04658357 | 0.03013481 |
| cg04832063 | -0.0135331 |
| cg04858164 | -0.0139537 |
| cg04907802 | -0.140074 |
| cg04964099 | -0.0225671 |
| cg05004394 | 0.04821342 |
| cg05096292 | 0.01974643 |
| cg05192044 | 0.03208369 |
| cg05295324 | 0.00640282 |
| cg05422352 | -0.0085007 |
| cg05442902 | 0.03708367 |
| cg05473648 | 0.09257263 |
| cg05517189 | -0.0453702 |
| cg05627557 | 0.01480628 |
| cg05801062 | 0.25108334 |
| cg05968748 | -0.0690271 |
| cg05985562 | -0.0260632 |
| cg06015525 | 0.07854934 |
| cg06245711 | -0.0568229 |
| cg06320218 | -0.0002967 |
| cg06343015 | 0.31290596 |
| cg06465076 | 0.17307489 |
| cg06489037 | 0.14769765 |
| cg06513706 | 0.04972539 |
| cg06515667 | -0.0003568 |
| cg06530919 | 0.3910096 |
| cg06600309 | -0.0179077 |
| cg06655030 | 0.12844058 |
| cg06764225 | -0.0431016 |
| cg06778417 | -0.221247 |
| cg06784992 | -0.0254312 |
| cg06926735 | -0.0127189 |
| cg06952412 | -0.1051874 |
| cg06975311 | -0.0253838 |
| cg07016730 | -0.1345036 |
| cg07026489 | -0.0807665 |
| cg07158339 | -0.4675163 |
| cg07160025 | 0.14680156 |
| cg07172885 | 0.29000816 |
| cg07180649 | -0.1484661 |
| cg07254551 | -0.0777596 |
| cg07266115 | 0.02764442 |
| cg07312601 | -0.1722237 |
| cg07429812 | 0.08041215 |
| cg07451598 | -0.1868342 |
| cg07597976 | 0.04235266 |
| cg07635837 | -0.0187418 |
| cg07646293 | -0.0441532 |
| cg07682621 | 0.06104802 |
| cg07702659 | 0.01461794 |
| cg07934123 | 0.06124146 |
| cg08029023 | -0.1331185 |
| cg08274301 | 0.02293014 |
| cg08284867 | -0.2045461 |
| cg08306076 | 0.04725273 |
| cg08331960 | -0.0116633 |
| cg08447038 | -0.0816875 |
| cg08480996 | -0.0116268 |
| cg08506127 | -0.0451941 |
| cg08645860 | -0.2059528 |
| cg08667099 | 0.12000861 |
| cg08682625 | -0.1132458 |
| cg08835797 | -0.088094 |
| cg08860750 | 0.01712814 |
| cg08981690 | -0.3413291 |
| cg09256063 | 0.07566084 |
| cg09318840 | -0.1912271 |
| cg09515041 | 0.09925926 |
| cg09545123 | 0.1184195 |
| cg09555176 | -0.0613157 |
| cg09606727 | -0.00248 |
| cg09616617 | 0.04483267 |
| cg09722826 | -0.2036391 |
| cg09736602 | 0.03287988 |
| cg09799873 | -0.148244 |
| cg09809793 | -0.0376725 |
| cg10045491 | 0.22533595 |
| cg10220472 | -0.1483562 |
| cg10243707 | 0.00618757 |
| cg10314406 | -0.058564 |
| cg10499974 | -0.0661892 |
| cg10512089 | 0.00759754 |
| cg10636246 | 0.1911046 |
| cg10695879 | 0.01896017 |
| cg10721215 | -0.0660615 |
| cg10779750 | 0.16331345 |
| cg10839301 | 0.02834544 |
| cg10856393 | 0.0292763 |
| cg10876821 | -0.0421113 |
| cg10983579 | -0.0542114 |
| cg11039901 | -0.1572152 |
| cg11120353 | 0.31620892 |
| cg11195951 | 0.03177488 |
| cg11197216 | -0.7702114 |
| cg11691077 | 0.07572611 |
| cg11864866 | -0.0250217 |
| cg11953805 | 0.27412879 |
| cg12126232 | 0.06809503 |
| cg12177001 | 0.03986163 |
| cg12240124 | -0.1920196 |
| cg12261786 | -0.1656898 |
| cg12324144 | -0.2157685 |
| cg12344862 | 0.0518649 |
| cg12406333 | -0.0193232 |
| cg12440372 | -0.210681 |
| cg12644222 | 0.05084048 |
| cg12655112 | -0.2139666 |
| cg12796724 | -0.0012364 |
| cg12813792 | -0.0650734 |
| cg12936001 | -0.0093203 |
| cg12941369 | -0.02795 |
| cg12991365 | 0.01227313 |
| cg13000827 | -0.0449442 |
| cg13029792 | 0.02329625 |
| cg13038560 | 0.33629708 |
| cg13040461 | -0.0265323 |
| cg13097175 | 0.01074669 |
| cg13149200 | -0.135147 |
| cg13642593 | 0.04625326 |
| cg13662954 | -0.1510063 |
| cg13756118 | -0.1123577 |
| cg13828047 | -0.2175229 |
| cg13842103 | -0.1478549 |
| cg13980705 | 0.18937455 |
| cg13985028 | 0.02892954 |
| cg14103663 | -0.052543 |
| cg14175847 | 0.14981524 |
| cg14229983 | 0.09975723 |
| cg14384491 | -0.0008593 |
| cg14437289 | 0.10241227 |
| cg14439745 | 0.05968099 |
| cg14477767 | 0.03375205 |
| cg14490946 | 0.19162123 |
| cg14518178 | -0.0257176 |
| cg14555759 | 0.31559628 |
| cg14574996 | -0.0144217 |
| cg14668986 | 0.0241284 |
| cg14747225 | -0.0634526 |
| cg14815794 | -0.1236301 |
| cg14878057 | -0.0383493 |
| cg15050015 | 0.00978006 |
| cg15061507 | -0.0748222 |
| cg15087459 | -0.1107492 |
| cg15109083 | -0.0065479 |
| cg15243979 | 0.09023678 |
| cg15411900 | 0.15355875 |
| cg15464531 | -0.0365333 |
| cg15469181 | -0.3850787 |
| cg15708373 | 0.08324733 |
| cg15772525 | 0.04045746 |
| cg15800651 | 9.82752785993189e-05 |
| cg15867698 | -0.041233 |
| cg15876728 | -0.0277608 |
| cg16012294 | 0.01431645 |
| cg16089221 | -0.0588176 |
| cg16156067 | 0.18351355 |
| cg16176526 | -0.000415 |
| cg16224928 | 0.07033572 |
| cg16454504 | -0.0323176 |
| cg16549957 | 0.0153298 |
| cg16587137 | 0.0742326 |
| cg16612562 | 0.01128067 |
| cg16630461 | -0.1154074 |
| cg16767270 | -0.1225447 |
| cg16810279 | -0.0146573 |
| cg16825428 | 0.07127472 |
| cg16878881 | 0.04291277 |
| cg16965309 | -0.1061893 |
| cg17020595 | 0.03486668 |
| cg17037231 | -0.9950612 |
| cg17099569 | 0.29369724 |
| cg17116828 | -0.12643 |
| cg17119557 | -0.0683582 |
| cg17162190 | -0.0709878 |
| cg17431739 | -0.0068832 |
| cg17536848 | -0.2416347 |
| cg17593187 | 0.04762717 |
| cg17673691 | -0.0058146 |
| cg17679560 | -0.176023 |
| cg17686885 | -0.5831393 |
| cg17696662 | -0.0334466 |
| cg17717866 | -0.0473834 |
| cg17744662 | -0.0622806 |
| cg17768768 | -0.1130281 |
| cg17850132 | 0.03736185 |
| cg17872229 | -0.0689286 |
| cg17900644 | -0.0281754 |
| cg17909166 | -0.0607874 |
| cg18166817 | -0.1851496 |
| cg18303397 | -0.1282276 |
| cg18315514 | 0.02538992 |
| cg18333000 | 0.12712459 |
| cg18368924 | -0.0109156 |
| cg18404996 | -0.0168392 |
| cg18527971 | 0.06258659 |
| cg18546384 | -0.0245403 |
| cg18699014 | 0.35048326 |
| cg18753452 | 0.49216499 |
| cg18753841 | -0.1570838 |
| cg18830527 | 0.05601543 |
| cg18857618 | 0.0621511 |
| cg18874294 | -0.003118 |
| cg18881501 | 0.02059281 |
| cg19062768 | -0.0551384 |
| cg19103271 | -0.0540599 |
| cg19274680 | 0.16751208 |
| cg19356189 | -0.1912928 |
| cg19408207 | 0.00117913 |
| cg19460836 | 0.04288419 |
| cg19500162 | -0.1135482 |
| cg19563106 | -0.0294403 |
| cg19686797 | -0.004233 |
| cg19860734 | -0.0558014 |
| cg19933595 | 0.15812262 |
| cg20031233 | -0.0451704 |
| cg20038100 | 0.00323657 |
| cg20138186 | -0.0347672 |
| cg20252363 | 0.14677396 |
| cg20346209 | -0.1491284 |
| cg20386303 | -0.0957754 |
| cg20417315 | -0.1722795 |
| cg20460771 | -0.0656855 |
| cg20512638 | 0.0499089 |
| cg20610950 | -0.0360857 |
| cg20659057 | -0.0191777 |
| cg20669012 | -0.10569 |
| cg20734673 | 0.02472505 |
| cg20755989 | 0.1555576 |
| cg20945853 | -0.1653247 |
| cg20959113 | 0.20299938 |
| cg20975542 | 0.09263567 |
| cg20983117 | 0.01022149 |
| cg21137706 | -0.0180784 |
| cg21221767 | -0.1789586 |
| cg21329721 | 0.04925064 |
| cg21335435 | -0.0367083 |
| cg21363706 | 0.00582749 |
| cg21524116 | -0.1024562 |
| cg21593149 | -0.0323966 |
| cg21648425 | -0.0504088 |
| cg21919939 | 0.02362889 |
| cg22033193 | 0.05712453 |
| cg22141415 | 0.10960831 |
| cg22213043 | -0.1737815 |
| cg22502858 | -0.1087894 |
| cg22566719 | -0.2013395 |
| cg22666439 | 0.10264481 |
| cg22719564 | 0.31364822 |
| cg22940717 | 0.00382313 |
| cg22947612 | 0.01837447 |
| cg23018285 | -0.0132119 |
| cg23025641 | 0.02373221 |
| cg23213217 | 0.04331265 |
| cg23223457 | 0.01714929 |
| cg23284252 | 0.07540482 |
| cg23300110 | -0.0134254 |
| cg23368743 | 0.03171427 |
| cg23490170 | 0.02247335 |
| cg23509770 | -0.123046 |
| cg23638400 | -0.0060742 |
| cg23661721 | 0.09318244 |
| cg23726036 | 0.00686321 |
| cg23740045 | -0.1029785 |
| cg23748504 | 0.08293733 |
| cg23798387 | -0.0092471 |
| cg24049629 | -0.0185709 |
| cg24107728 | -0.0461952 |
| cg24262469 | 0.17473777 |
| cg24267761 | -0.0414708 |
| cg24437280 | -0.0460096 |
| cg24445388 | -0.1987886 |
| cg24482288 | -0.1531647 |
| cg24491929 | -0.1948773 |
| cg24580001 | 0.07073439 |
| cg24620208 | -0.0505396 |
| cg24894712 | 0.07313065 |
| cg24901985 | -0.0460063 |
| cg25008538 | 0.04868076 |
| cg25032635 | -0.0419568 |
| cg25054515 | -0.1715532 |
| cg25098139 | -0.0003755 |
| cg25229964 | -0.1102217 |
| cg25235205 | -0.0374102 |
| cg25300034 | 0.03433667 |
| cg25410668 | 0.42564893 |
| cg25428494 | -0.0438061 |
| cg25483161 | -0.0692027 |
| cg25529140 | 0.03974781 |
| cg25771195 | 0.34772168 |
| cg25813765 | 0.03190977 |
| cg25854386 | -0.215788 |
| cg25963407 | 0.17792715 |
| cg25976696 | 0.03619049 |
| cg26066921 | -0.0539759 |
| cg26073614 | 0.09501266 |
| cg26076856 | -0.1680454 |
| cg26154047 | 0.03862623 |
| cg26178805 | -0.0599193 |
| cg26191916 | 0.07542741 |
| cg26501210 | 0.05992309 |
| cg26530885 | -0.2542885 |
| cg26552743 | -0.0952192 |
| cg26631126 | -0.1136915 |
| cg26692003 | 0.04556727 |
| cg26693978 | -0.0650395 |
| cg26749175 | -0.1513613 |
| cg26872866 | 0.16700979 |
| cg26933021 | -0.011585 |
| cg26934229 | 0.12200723 |
| cg27027151 | -0.0581248 |
| cg27059140 | 0.10002206 |
| cg27106643 | 0.18626487 |
| cg27171735 | -0.0134514 |
| cg27273520 | 0.15764833 |
| cg27359471 | 0.00963804 |
| cg27377450 | 0.21246868 |
| cg27423838 | 0.29200194 |
| cg27507284 | 0.02665231 |
| cg27563198 | 0.05725145 |
| ch.12.1107853R_II_R_O_37490 | 0.08393832 |
| ch.2.30415474F_II_F_O_37488 | -0.0035221 |

Table S7.

**Pan-Mammalian Species Multi-Tissue Retroelement Clock CpGs**

| ProbeID | coefficient |
| --- | --- |
| (Intercept) | 2.46378276 |
| cg00113096 | -0.080915 |
| cg00366435 | -0.0464587 |
| cg00443833 | -0.008698 |
| cg00658652 | 0.71230928 |
| cg00687674 | -0.3757965 |
| cg00702872 | -0.2240097 |
| cg00847072 | 0.09931785 |
| cg00851088 | 0.23816614 |
| cg01002223 | -0.2684983 |
| cg01040649 | 0.15898007 |
| cg01079563 | -0.063277 |
| cg01128603 | -0.1381551 |
| cg01167616 | -0.2819916 |
| cg01334830 | -0.1784901 |
| cg01423660 | -0.2596485 |
| cg01508445 | 0.02202168 |
| cg01620430 | 2.65758086 |
| cg01668476 | -0.2724877 |
| cg01689963 | 0.24347773 |
| cg01822313 | 0.02558918 |
| cg01881899 | -0.0223969 |
| cg01949403 | -0.0639084 |
| cg02071305 | -0.265757 |
| cg02110337 | -0.2405327 |
| cg02151301 | -0.177291 |
| cg02239453 | -0.3761202 |
| cg02315120 | -0.2157428 |
| cg02426735 | 0.09175557 |
| cg02650131 | -0.2238308 |
| cg02714514 | 0.02381045 |
| cg03077729 | -0.119638 |
| cg03535648 | 0.12336587 |
| cg03574244 | -0.0624198 |
| cg03592537 | -0.4834914 |
| cg03603104 | 0.04645381 |
| cg03637218 | 0.03210941 |
| cg04126866 | -0.0689475 |
| cg04162316 | -0.2330581 |
| cg04347414 | 0.62357187 |
| cg04408104 | -0.0895575 |
| cg04576021 | -0.1172712 |
| cg04631128 | -0.1305959 |
| cg04658357 | 0.23617346 |
| cg04858164 | -0.2412145 |
| cg05096292 | 0.15296451 |
| cg05192044 | -0.0113242 |
| cg05442902 | 0.28975561 |
| cg05627557 | 0.2534815 |
| cg05723552 | -0.2154455 |
| cg05902130 | 0.00423419 |
| cg06015525 | -0.0171278 |
| cg06245711 | -0.3574825 |
| cg06297318 | 0.19487698 |
| cg06343015 | 0.31142489 |
| cg06655030 | 0.89165587 |
| cg06952412 | -0.1892231 |
| cg07172885 | 0.50883904 |
| cg07266115 | 0.11352691 |
| cg07330072 | 0.07035597 |
| cg07429812 | 0.13092883 |
| cg07455279 | -0.0791804 |
| cg07597976 | 0.05651901 |
| cg07635837 | -0.1673706 |
| cg07702659 | 0.26991356 |
| cg08029023 | -0.1093661 |
| cg08318939 | -0.0421138 |
| cg08331960 | 0.01605331 |
| cg08420066 | -0.1296299 |
| cg08482359 | 0.07259461 |
| cg08506127 | -0.0547814 |
| cg08645860 | -0.0546165 |
| cg08860750 | 0.39692334 |
| cg09256063 | -0.0868117 |
| cg09318840 | -0.1444504 |
| cg09382517 | -0.0137173 |
| cg09515041 | 0.0784026 |
| cg10242074 | 0.10313389 |
| cg10314406 | 0.19282835 |
| cg10480329 | -0.1430186 |
| cg10512089 | -0.005333 |
| cg10636246 | 0.52930935 |
| cg10695879 | 0.15042093 |
| cg10779750 | 0.1094682 |
| cg10804966 | -0.4330141 |
| cg10856393 | -0.0662329 |
| cg10900550 | -0.047251 |
| cg11039901 | -0.1084446 |
| cg11120353 | 0.22416054 |
| cg11195951 | 0.14828007 |
| cg11197216 | -0.704485 |
| cg11335172 | 0.32997711 |
| cg11706849 | -0.0451663 |
| cg11860347 | 0.10736668 |
| cg12177001 | 0.10252551 |
| cg12324144 | -0.1458979 |
| cg12440372 | -0.3296246 |
| cg12480541 | -0.1537022 |
| cg12531611 | -0.0289777 |
| cg12655112 | -0.7345056 |
| cg12813792 | -0.1889554 |
| cg12830694 | -0.0362533 |
| cg12936001 | 0.00850495 |
| cg12941369 | -0.5176075 |
| cg13000827 | 0.04103384 |
| cg13029792 | 0.32062623 |
| cg13040461 | -0.1385936 |
| cg13296371 | -0.4977716 |
| cg13571802 | 0.06505408 |
| cg13828047 | -0.4879414 |
| cg13980705 | 0.02583176 |
| cg14109551 | 0.00015193 |
| cg14175847 | 0.15331307 |
| cg14229983 | 0.2831248 |
| cg14490946 | 0.41604786 |
| cg14518178 | -0.3007138 |
| cg14574996 | -0.091276 |
| cg14668986 | -0.0561152 |
| cg14747225 | -0.2602147 |
| cg14907310 | 0.17118967 |
| cg15035590 | -0.1194578 |
| cg15087459 | -0.02618 |
| cg15348679 | 0.19742903 |
| cg15411900 | 0.16506852 |
| cg15464531 | -0.1659045 |
| cg15657641 | -0.252091 |
| cg16012294 | -0.010638 |
| cg16089221 | -0.13621 |
| cg16247867 | -0.2857586 |
| cg16366997 | -0.0359824 |
| cg16549957 | 0.35348235 |
| cg16630461 | -0.1520496 |
| cg16767270 | -0.2603156 |
| cg16810279 | -0.0191145 |
| cg16965309 | -0.1676186 |
| cg17037231 | -1.5875365 |
| cg17116828 | -0.3474791 |
| cg17162190 | -0.1833479 |
| cg17673691 | -0.5613065 |
| cg17850132 | -0.0005966 |
| cg18303397 | -0.2898143 |
| cg18315514 | -0.0521213 |
| cg18333000 | -0.2274175 |
| cg18830527 | 0.05443444 |
| cg18874294 | -0.2609749 |
| cg18984151 | -0.0893322 |
| cg19274680 | 0.40002689 |
| cg19346193 | -0.1952118 |
| cg19408207 | 0.14809955 |
| cg19460836 | 0.19054233 |
| cg19573166 | 0.05257434 |
| cg19615265 | 0.17441468 |
| cg20211859 | -0.2429622 |
| cg20386303 | -0.2987999 |
| cg20610950 | -0.0855055 |
| cg20659057 | -0.1446862 |
| cg20891917 | 0.03296486 |
| cg20945853 | -0.0252757 |
| cg20983117 | 0.03521781 |
| cg21005240 | -0.155262 |
| cg21137706 | -0.6534762 |
| cg21363706 | -7.13E-05 |
| cg21524116 | -0.0011424 |
| cg21761853 | 0.02732057 |
| cg22141415 | 0.26812732 |
| cg22194129 | -0.0333122 |
| cg23028740 | 0.14826999 |
| cg23213217 | 0.13677109 |
| cg23368743 | 0.10932285 |
| cg23726036 | -0.042114 |
| cg23740045 | -0.2331018 |
| cg24049629 | -0.0240004 |
| cg24254120 | -0.0205399 |
| cg24262469 | 0.74202682 |
| cg24445388 | -0.9729429 |
| cg24482288 | 0.07210447 |
| cg24491929 | -0.287776 |
| cg24620208 | -0.1044243 |
| cg24894712 | 0.32131198 |
| cg25008538 | 0.12551494 |
| cg25334215 | 0.25838326 |
| cg25410668 | 0.70249567 |
| cg25428494 | -0.1272596 |
| cg25599673 | -0.3139609 |
| cg25771195 | 0.58993939 |
| cg26076856 | -0.1138498 |
| cg26150823 | 0.13248025 |
| cg26154047 | 0.11579716 |
| cg26178805 | -0.2380353 |
| cg26191916 | 0.14610996 |
| cg26251396 | -0.000646 |
| cg26316557 | 0.22792455 |
| cg26538782 | 0.12109588 |
| cg26749175 | -0.0350511 |
| cg26933021 | -0.1927365 |
| cg26934229 | 0.06831216 |
| cg27027151 | -0.3800803 |
| cg27059140 | 0.36497213 |
| cg27106643 | 0.33412116 |
| cg27423838 | 0.45810099 |
| ch.13.39564907R_II_R_O_37491 | 0.0010903 |
| ch.2.30415474F_II_F_O_37488 | 0.08980621 |
